# Operational tropical cyclone forecasting with AI

**DOI:** 10.1038/s41586-026-10953-2

**Published:** 2026-08-06

**Authors:** Ferran Alet, Tom R. Andersson, Ilan Price, Stratis Markou, Andrew El-Kadi, Dominic Masters, Amy Li, Samier Merchant, Natalie Williams, Gregory Thornton, Ken MacKay, Olivia Graham, Akib Uddin, Ben Gaiarin, Devaja Shah, Elinor Kruse, Wallace Hogsett, David Zelinsky, John Cangialosi, Jonathan Martinez, James Franklin, Mark DeMaria, Kate Musgrave, Caroline L. Bain, Helen Titley, Jacklynn Stott, Remi Lam, Aaron Bell, Paul Komarek, Matthew Willson, Alvaro Sanchez-Gonzalez, Peter Battaglia

**Affiliations:** 1 Google DeepMind, London, UK; 2 University of Waterloo, Waterloo, Ontario Canada; 3 Google Research, Mountain View, CA USA; 4 NOAA/NWS/NCEP National Hurricane Center, Miami, FL USA; 5 Cooperative Institute for Research in the Atmosphere, Colorado State University, Fort Collins, CO USA; 6 UK Met Office, Exeter, UK

**Keywords:** Natural hazards, Atmospheric dynamics

## Abstract

Tropical cyclones are among the most dangerous and costly weather phenomena, but forecasting them remains challenging. Here we introduce WeatherNext Cyclones (WN-C), an artificial intelligence (AI)-based operational weather model that produces state-of-the-art ensemble forecasts of the track, intensity and size of tropical cyclones worldwide. Trained on a combination of global analysis data^1^ and a global database of historical tropical cyclones^2,3^, WN-C generates large ensembles of possible global weather and cyclone scenarios extending 15 days into the future. When evaluated on tropical cyclones from 2023 to 2025, the track, intensity and wind-radius predictions from WN-C offer an average lead-time advantage of 1 day or more over leading operational models—an improvement in accuracy comparable to the progress seen in the last decade of operational development. We achieved these results using inputs that are orders of magnitude coarser than regional models, suggesting that high resolution is not a strict prerequisite for state-of-the-art intensity forecasting and that these coarse atmospheric data contain more intensity signal than has been previously recognized. Including predictions from WN-C in a weighted-average consensus ensemble improves its skill substantially. The scalability of WN-C enables ensembles of up to 1,000 members, which are better at capturing rare events than conventional 50-member ensembles. By providing advanced operational ensemble guidance to human forecasters, this work represents a step change towards more reliable and timely forecasts and warnings that can help to protect lives and to mitigate the devastating effects of tropical cyclones.

## Abstract (web summary)

An AI forecasting system called WeatherNext Cyclones can predict the track, intensity (maximum wind speed) and size of tropical cyclones with an accuracy gain over leading operational models comparable to a decade of progress in numerical forecasting.

## Main

Tropical cyclones, sometimes referred to as hurricanes or typhoons, are powerful rotating storms that form over warm ocean waters and can cause widespread devastation when they make landfall. In the past 50 years, they have been responsible for more than 700,000 deaths and US$1.4 trillion in economic losses globally^4^. Accurate and timely forecasts are crucial for issuing effective warnings, enabling evacuations and preparing emergency responses. Three principal factors influence the downstream effects of a tropical cyclone: its track (where a cyclone forms and where it goes), its intensity (its maximum wind speed) and its structure (its size and shape). For decades, public forecasting agencies such as the US National Hurricane Center (NHC) have provided forecasts of these parameters and associated tropical cyclone hazards several days into the future, to forewarn about incoming impacts.

Forecasting these complex systems is notoriously difficult. The track of a cyclone is governed mainly by large-scale atmospheric steering currents and cyclone–environment interactions, and its intensity is thought to also be strongly dictated by fine-scale thermodynamic processes within and around its core^5,6^. This has led to a long-standing scale dilemma in operational forecasting, because numerical weather prediction (NWP) models must simulate the atmosphere at high spatial resolutions to capture intensity changes, but this is too expensive to do over a very large domain^7,8^. Global NWP models, such as the European Centre for Medium-Range Weather Forecasts (ECMWF)’s ensemble system (ENS)^9^, excel at capturing this large-scale atmospheric flow and thus generally produce the most skilful track forecasts, but lack the resolution to accurately forecast intensity. Conversely, specialized and deterministic high-resolution regional models, such as the US National Oceanic and Atmospheric Administration (NOAA)’s Hurricane Analysis and Forecast System (HAFS)^10^, better capture intensity, but at the expense of track accuracy.

In recent years, AI has emerged as a powerful new paradigm for weather prediction, and models have achieved superior forecast accuracies and speeds, compared with NWP systems, for core atmospheric state variables^11–14^. These models show state-of-the-art track prediction for tropical cyclones^14^, but inherit the low-intensity bias of 0.25° global analysis owing to low resolution (around 28 km). Poor intensity skill has been noted as a major limitation of AI weather models^15^. Several works have combined AI with NWP^16,17^, and show improved track forecasts compared with NWP models and improved intensities compared with AI models, whereas other approaches have explored end-to-end approaches based on observational data^18–20^. However, these approaches have not achieved state-of-the-art results in operational tropical cyclone forecasting.

**Fig. 1:**
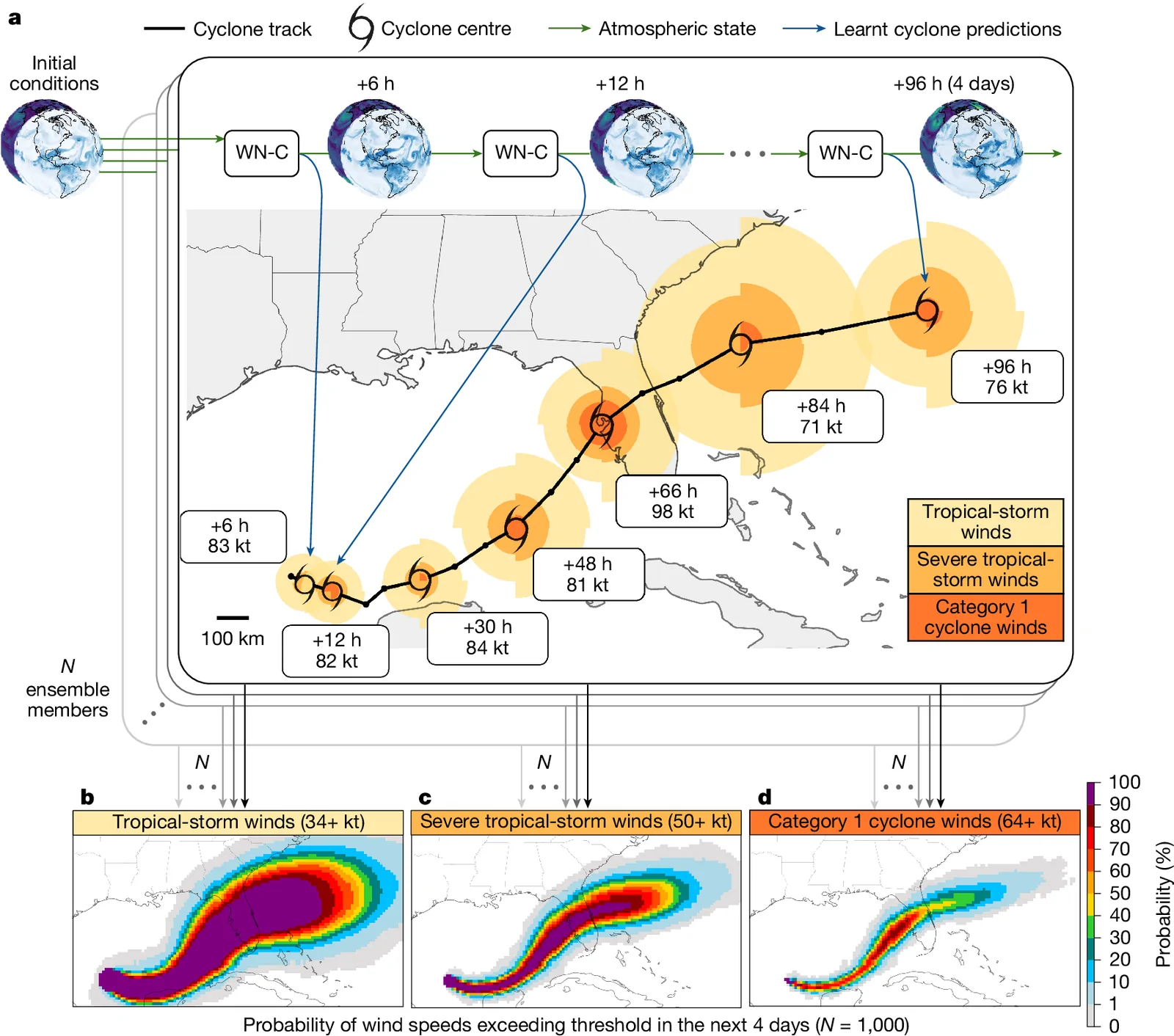
Schematic of a WN-C ensemble forecast. This forecast is initialized on 7 October 2024, 00:00 UTC, during Hurricane Milton. **a**, Each WN-C ensemble member is initialized with operational ECMWF analysis, and produces iterative 6-h forecasts of (1) the atmospheric state (comprising 84 weather variables) and (2) tropical cyclone trajectories out to 15 days, by autoregressively feeding its atmospheric state prediction as input to the next model step (green arrows). WN-C is trained to produce additional target-only gridded channels for cyclone trajectory variables, from which an inference-time tracking algorithm extracts tabular trajectories of tropical cyclone position, intensity and the greatest extents of 34 kt, 50 kt and 64 kt maximum 1-min sustained wind speeds in four quadrants (blue arrows; see ‘End-to-end cyclone forecasting’). A WN-C ensemble of size *N* is generated by a stochastic sampling process (see ‘Probabilistic joint modelling with FGNs’). The ensemble member in the panel was chosen for illustrative purposes only. **b**–**d**, Gridded fields showing the cumulative probability of tropical cyclone winds reaching 34 kt (**b**), 50 kt (**c**) and 64 kt (**d**) at any time within a 4-day forecast window, derived from the wind radii of an *N* *=* 1,000-member ensemble from WN-C, predicting the threat of cyclone-induced winds to specific geographical locations.

Here we introduce WeatherNext Cyclones (WN-C), an AI system that excels at both track and intensity forecasting. WN-C is a probabilistic system that generates an ensemble of 50 (or more) forecasts of possible scenarios to quantify uncertainty. It is jointly trained on a combination of two data sources (a process we refer to as co-training): decades of global atmospheric analysis data from the ECMWF^1^, capturing global weather dynamics, and cyclone data from the International Best Track Archive for Climate Stewardship (IBTrACS)^2,3^, a specialized, expert-curated database of nearly 5,000 observed tropical cyclones, which provides detailed information on their track, intensity and wind radii (Fig. 1).

We evaluated WN-C on historical data from 2023–2025, and have been releasing real-time predictions publicly on Google’s Weather Lab (https://deepmind.google.com/science/weatherlab) since June 2025. Our verification shows that WN-C’s ensemble mean is significantly more accurate than the ENS ensemble mean on track, and than the specialized HAFS model on intensity and quadrant wind radii. WN-C’s improvements in track and intensity accuracy represent a gain of 1 day or more in forecast lead-time advantage, an advancement comparable with the last decade’s worth of progress in NWP forecast accuracy (Extended Data Figs. 8 and 9 and Supplementary Information section 2.7). Furthermore, as an ensemble model, WN-C generates what are to our knowledge the most skilful, and best calibrated, intensity forecasts and wind-speed probabilities so far. This work shows that AI has the potential to be used to provide forecasters at weather agencies with more accurate and reliable guidance, ultimately leading to better-informed decisions that could save lives and protect communities.

## An ensemble weather and cyclone model

### Problem formulation

We formulate the forecasting task as joint prediction over two data modalities to capture the multi-scale nature of tropical cyclones: (1) the dense, global atmospheric state *X*, a gridded representation of large-scale weather; and (2) a sparse, tabular description of cyclone trajectories *C*, capturing properties that the coarse grid cannot resolve.

At any time step *t*, the state of all active cyclones is given by ${C}^{t}=\{({{\bf{p}}}_{i}^{t},{{\bf{s}}}_{i}^{t}){\}}_{i=1}^{{N}_{t}}$, where ${N}_{t}$ is the number of active storms, ${{\bf{p}}}_{i}^{t}=({\phi }_{i}^{t},{\lambda }_{i}^{t})$ denotes position (latitude, longitude) and ${{\bf{s}}}_{i}^{t}\in {{\mathbb{R}}}^{k}$ denotes *k* associated physical scalars.

Because atmospheric dynamics are inherently chaotic and observations imperfect, deterministic predictions cannot fully capture forecast uncertainty^21^. We therefore adopt a probabilistic framework: our goal is to draw samples from the joint probability distribution over *T-*step trajectories, $p(\{X,C{\}}^{1:T}|{X}^{\le 0})$. Following standard operational practice, we condition on initial analysis states ${X}^{\le 0}$ from a preceding data-assimilation process. We model the system as a second-order Markov process, so the multi-step distribution factorizes into a product of single-step conditional distributions:

$$\begin{array}{c}p(\{X,C{\}}^{1:T}|{X}^{0},{X}^{-1})=\mathop{\prod }\limits_{t=1}^{T}p(\{X,C{\}}^{t}|{X}^{t-2:t-1}).\end{array}$$

We train a single-step model to sample from $p(\{X,C{\}}^{t}|{X}^{t-2:t-1})$, with a time step of 6 h, using an autoregressive loss up to 3 days lead time (Supplementary Table 1). Applying the model autoregressively, we produce full ensemble forecasts out to 15 days. We follow standard practice in AI NWP^11,12,14^, and use the initial atmospheric variables *X*^0^, *X*^−1^ with no initial condition uncertainty.

In this work, *X* comprises 6 atmospheric variables at 13 vertical pressure levels and 6 surface variables on a 0.25° latitude–longitude grid, following previous studies^11,14^. For each cyclone in *C*, the scalar vector ${{\bf{s}}}_{i}^{t}$ comprises maximum sustained wind speed; minimum sea-level pressure; radius of maximum wind; and four quadrant radii for 34-kt, 50-kt, and 64-kt winds (Supplementary Table 2).

### End-to-end cyclone forecasting

Cyclone forecasting (both NWP and AI-based) conventionally relies on a two-step process: predict a sequence of future atmospheric states (${X}^{1:T}$), then apply a separate cyclone tracking algorithm to extract cyclone trajectories ${C}^{1:T}$. However, coarse analysis data systematically underestimate cyclone intensity, and training on atmospheric states alone provides no direct learning signal for cyclone-specific features.

To overcome this, we apply a deterministic ‘griddification’ mapping from the tabular cyclone state *C*^*t*^ into two tensors on the 0.25° grid (see ‘Griddification of cyclone best-track data’ in the Methods). The first tensor is the cyclone existence probability, *E*^*t*^, populated by placing a Gaussian kernel at each cyclone centre ${{\bf{p}}}_{i}^{t}$, providing a dense gradient field that encourages the model to predict the presence and precise location of true cyclone centres during training^22^. The second tensor contains conditional cyclone scalar properties, *S*^*t*^, constructed by spatially broadcasting the values of ${{\bf{s}}}_{i}^{t}$ within discs centred on the ${{\bf{p}}}_{i}^{t}$, and masked elsewhere so that the model receives training signal only at the times and locations of active cyclones (see ‘Model training for cyclone variables’ in the Methods).

During inference, after model training, the griddification mapping of C→(E,S) is inverted by a heuristic tracker (E,S)→C to extract predicted tabular trajectories $\hat{C}$ (see ‘Cyclone tracking algorithm’ in the Methods). This end-to-end framework enables training on the differentiable prediction of cyclone presence and properties, bypassing non-differentiable tracking, while still recovering tabular tracks at inference.

Although *C* is not provided as model input, jointly predicting *X* and *C* allows the system to infer fine-scale storm properties from the large-scale atmospheric state. Furthermore, it enables the sharing of latent representations learned from the approximately 20-TB gridded atmospheric analysis training data *X* and the approximately 10-MB tabular IBTrACS dataset *C*, mutually improving performance on both data modalities. By contrast, a two-step AI model that (1) predicts *X* alone and then (2) predicts *C* from *X*, would not have this transfer learning benefit, as shown by the comparison with GenCast and the ablation in Extended Data Fig. 4.

### Probabilistic joint modelling with FGNs

Weather and cyclone forecasting involve considerable epistemic uncertainty (model imperfection due to finite training data) and aleatoric uncertainty (unresolved atmospheric chaos). We train WN-C by optimizing the fair continuous ranked probability score (CRPS)^23^, a strictly proper scoring rule, to ensure that at each place and time, samples from our model capture the full probability distribution instead of just its mean, as happens with deterministic objectives such as mean squared error. In this sense, CRPS is only a marginal loss—it asks the model to fit the pointwise distributions, but does not necessarily constrain covariances and other aspects of the joint distribution. Cyclone variables, however, are examples of ‘joints’, in that they involve multiple variables and locations and are affected by their interdependencies. For instance, the cyclone centre is a local minimum in surface pressure, and the quadrant radii are defined by the entire region around the centre.

We enable the learning of joints via the CRPS loss in two ways: first, as explained in the previous section, we convert tabular cyclone joint variables to marginals and add them to the objective. Second, rather than per-pixel noise in input or output space, we express uncertainty in functional space, an approach we call functional generative networks (FGNs; details in ‘Functional generative networks’ in the Methods). To capture epistemic uncertainty, we use deep ensembles^24^, training multiple independent models from different randomly initialized weights. Aleatoric uncertainty is modelled by sampling neural network parameters ${\theta }_{i}^{t}$ from a learned distribution ${{\mathcal{P}}}_{{\mathcal{M}}}(\theta )$ (implemented via a low-dimensional Gaussian noise vector injected through conditional normalization layers; see ‘Model architecture’ in the Methods), perturbing the model at each time step to generate diverse, physically coherent trajectories.

Compared to previous diffusion-based models such as GenCast^14^, FGN requires only a single neural network call per step, making it eight times faster at inference and substantially easier to model the sparse cyclone targets.

## Evaluation methodology

We evaluate WN-C’s deterministic and probabilistic skill on gridded atmospheric states and cyclone track, intensity, structure, rapid intensification (RI; an increase of 30 kt or more in maximum sustained winds within 24 h; ref. ^25^) and cyclogenesis. For cyclone evaluations, we perform both paired evaluations (considering only already-formed cyclones) and unpaired evaluations, which also assess the model’s ability to forecast the formation and progression of new cyclones.

### Baselines

No single baseline excels across all prediction tasks^26,27^, so we compare against the state-of-the-art for each metric (Supplementary Information section 2.3 and Supplementary Figs. 5 and 6).

1. ECMWF ENS: the leading global ensemble NWP system, used as a baseline for weather variables and cyclone forecasts globally.
2. NOAA HAFS-A: NOAA’s next-generation high-resolution regional NWP model specialized for cyclones; its forecasts in the North Atlantic and East Pacific basins are used as a baseline for deterministic cyclone forecasts. We refer to it as HAFS. HAFS performed similarly to the Hurricane Weather Research and Forecast model (HWRF) and the Hurricanes in a Multi-scale Ocean-coupled Non-hydrostatic regional model (HMON) on intensities, and better on tracks and structure (Supplementary Information section 2.3).
3. GenCast with debiased intensities: a global AI weather model^14^, fine-tuned on operational ECMWF high-resolution (HRES)-fc0 data (the first step of the ECMWF HRES forecast, serving as an operational analysis). Because GenCast is trained on coarse 0.25° analysis data that cannot resolve a cyclone’s peak winds, its raw intensity forecasts are systematically low-biased. To present the strongest possible baseline, we apply an optimized pressure–wind-speed relation^28^ to correct GenCast’s large wind-speed bias and improve its intensity performance (see ‘GenCast intensity forecasts using a pressure–wind-speed relation’ in the Methods).

#### Operational latency adjustment

Like all analysis-dependent models, WN-C’s forecasts arrive around 6.5 h after the synoptic time. For operational realism, all paired evaluations use forecasts initialized 6 h before, corrected with the latest real-time cyclone observations from the TCVitals archive^29,30^, a standard practice in operational forecast verification (Extended Data Fig. 3 and ‘Early forecast construction via interpolation’ in the Methods). Unpaired evaluations use unadjusted forecasts.

#### Ground truth

We verify gridded atmospheric state against ECMWF’s HRES-fc0 analysis (the ECMWF forecast at time 0), also used as the operational input, and cyclone predictions against IBTrACS^2,3^ (International Best Track Archive for Climate Stewardship) (see Supplementary Information section 1.4). Note that IBTrACS captures tropical cyclones, and thus only contains extratropical cyclones if they are part of an extratropical transition.

#### Evaluation protocol

After freezing all design choices (validated on 2022), we evaluated atmospheric state predictions on 2023 and cyclone trajectory predictions on 2023–2024, and the North Atlantic and East Pacific basins for 2025, for which a version of this model ran operationally. For each test year, the model was retrained up to the end of the preceding calendar year. Results are pooled across test years.

#### Statistical tests

We assess significance using bootstrap tests (1,000 replicates; paired metrics) and heteroskedasticity and autocorrelation robust (HAR) inference^31^ (unpaired metrics). Almost all reported improvements are statistically significant (*P* < 0.05); see Supplementary Information section 2.6.

#### Consensus ensembles

Operational forecasters use consensus ensembles as primary guidance. These are averages (possibly weighted) of individual deterministic guidance models, which typically achieve improved performance over their individual constituents. In our evaluations, we treat WN-C as a single guidance model and compare it to other single guidance baselines. In ‘Valuable operational guidance’ below, we also assess the value WN-C adds to consensus ensembles.

## Global weather forecasting

A model’s ability to predict a tropical cyclone’s path and evolution is dependent on its skill in forecasting the surrounding large-scale atmospheric environment. Therefore, before evaluating cyclone-specific performance, we establish WN-C’s medium-range weather-forecasting skill through comparisons with leading AI and NWP systems, GenCast^14^ and the ECMWF’s ENS^9^.

As measured by CRPS, our model significantly outperforms GenCast, on 99.8% (*P* < 0.05) of target variable-level pairs (Extended Data Fig. 5), with an improvement of 12 h, 15 h, 16 h and 17 h at lead times of 1 day, 3 days, 5 days and 7 days, respectively (Supplementary Fig. 2). It also significantly outperforms ENS on 99.2% (*P* < 0.05) of all evaluated variables, levels and lead times out to 15 days (Supplementary Fig. 1), with an improvement of average performance of 16 h, 26 h, 27 h and 30 h at 1 day, 3 days, 5 days and 7 days respectively (Supplementary Fig. 3). Scorecard evaluations of WN-C’s univariate and spatial and cross-variable joint structure are provided in Supplementary Information section 2.2 and Extended Data Fig. 6.

WN-C advances the state-of-the-art in global probabilistic weather forecasting. This provides the robust foundation on which the model’s specialized cyclone prediction capabilities are built. The lead-time improvements seen in analysis forecasting directly translate to lead-time improvements in cyclone track performance.

## Cyclone track forecasting

Accurate track forecasting is crucial for effective warnings about which areas are at risk. We evaluated the track accuracy of WN-C against ENS, the world’s leading operational system for this task, and GenCast, a top AI-based global weather model. Following the evaluation protocol of the NHC^32^, we perform homogeneous evaluation, meaning that a forecast time step for a given cyclone is included in the evaluation only if all models produced a forecast for that cyclone at that lead time, and the target cyclone was active at that lead time. Furthermore, we consider only systems classified in IBTrACS as having a tropical or subtropical nature (excluding disturbances, extratropical and unclassified systems).

**Fig. 2:**
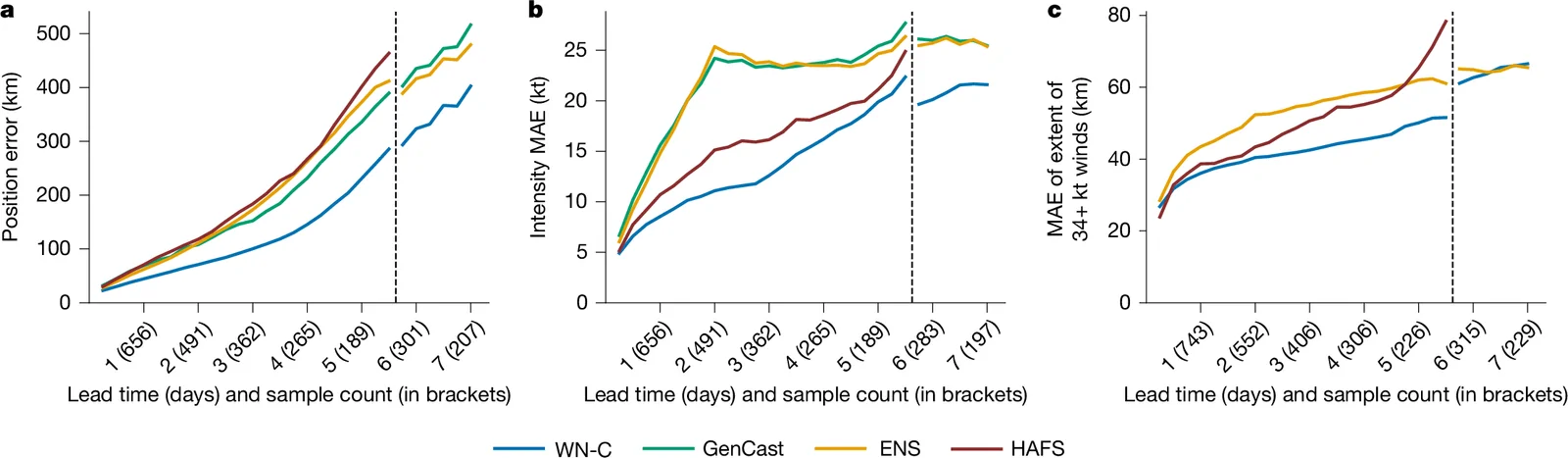
WN-C is skilful at forecasting deterministic cyclone track, intensity and wind radii. WN-C’s ensemble mean prediction obtains state-of-the-art performance for cyclone track, intensity and wind-radius forecasting in test years 2023–2024. We compare against the ENS ensemble mean, the GenCast ensemble mean and the HAFS model. Performance is evaluated only when all models make a prediction and the true system is classified as a hurricane, tropical storm, subtropical storm, tropical depression or subtropical depression. The vertical black dotted lines demarcate lead times beyond which HAFS stops producing forecasts, changing the homogeneous evaluation from the Atlantic & East Pacific to global. Sample counts are shown in brackets for each lead time (*x* axis). **a**, Mean track error. **b**, Intensity mean absolute error (MAE). Intensity is defined as the maximum 1-min sustained wind speed. **c**, MAE of 34-kt wind radius forecasts averaged over all quadrants. Note that GenCast does not appear because it does not predict quadrant radii. Ground-truth targets are IBTrACS best tracks^2,3^. Cyclone predictions from baselines were obtained from NHC operational a-decks^43^ (for HAFS) and from the ECMWF archive^9^ (for ENS).

**Extended Data Fig. 1:**
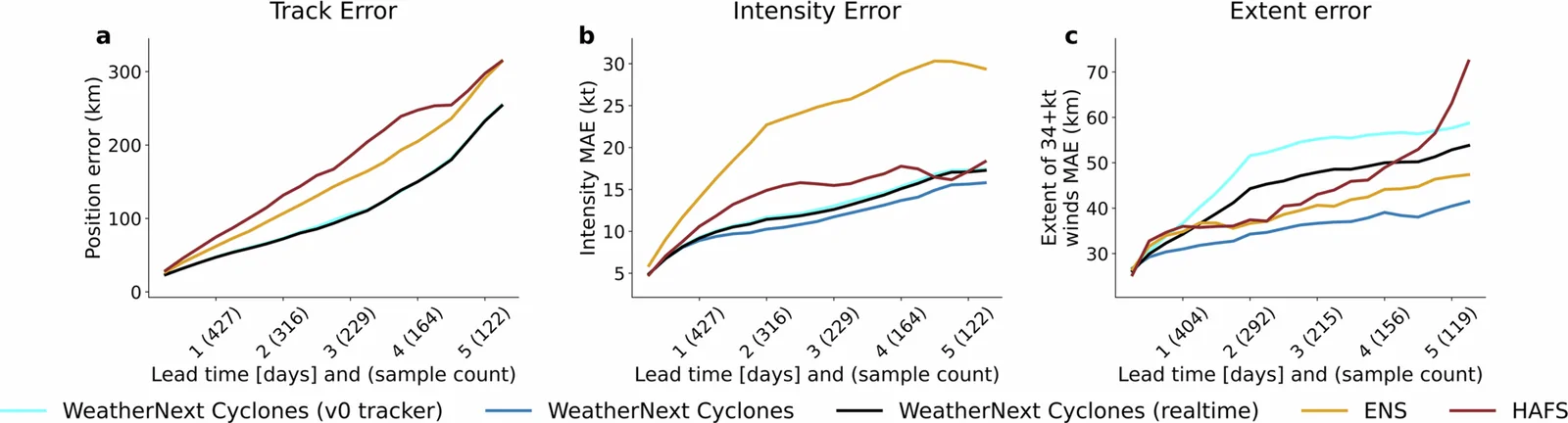
WN-C outperforms state-of-the-art guidance during the 2025 hurricane season. Deterministic paired evaluation on track, intensity and wind radii for 2025 NHC basins (North Atlantic and East Pacific), for which WN-C ran operationally in real time. Performance is evaluated only when all models make a prediction and the true system is classified as a hurricane, tropical storm, subtropical storm, tropical depression, or a subtropical depression. WN-C is evaluated with three tracker configurations: the v0 tracker, the present v1 tracker (WN-C), and the live predictions (switching to the v1 tracker in late September). **a**, Mean track error. **b**, Mean absolute intensity error. Intensity is defined as the maximum 1-minute sustained wind speed. **c**, MAE of 34-kt wind-radius forecasts averaged over all quadrants. Ground-truth targets are IBTrACS best-tracks^2,3^. Cyclone predictions from baselines were obtained from NHC operational a-decks^43^ (for HAFS) and the ECMWF archive^9^ (for ENS).

First we evaluate the position error of the ensemble mean track, which is dissipated when fewer than 30% of ensemble members maintain a cyclone (see Methods). WN-C achieves substantially lower track errors at all lead times across 2023–2024 (Fig. 2a), and 2025 NHC basins (Extended Data Fig. 1). These results are consistent across ocean basins and observed cyclones (Extended Data Fig. 10 and Supplementary Information section 2.8). At the 5-day forecast horizon, the ensemble mean track from WN-C has an average error of 230 km, a 140-km improvement over the 370-km error of ENS and a 105-km improvement over GenCast’s 335-km error at that lead time. ENS exceeds this 230-km error beyond approximately 3.75 days lead time, meaning that WN-C provides just over 30 h more advanced warning at that level of accuracy. Compared with GenCast, this improvement is approximately 24 h.

Beyond transitioning from diffusion models to FGNs, two other innovations brought improvements. As shown by ablation studies, co-training on cyclone data makes the model better at the physics associated with track forecasting, bringing gains of around 6 h at long lead times (Supplementary Fig. 7). Furthermore, the AI tracker can be more precise than the 0.25° grid, enabling better performance at the early lead times (Extended Data Fig. 4).

**Fig. 3:**
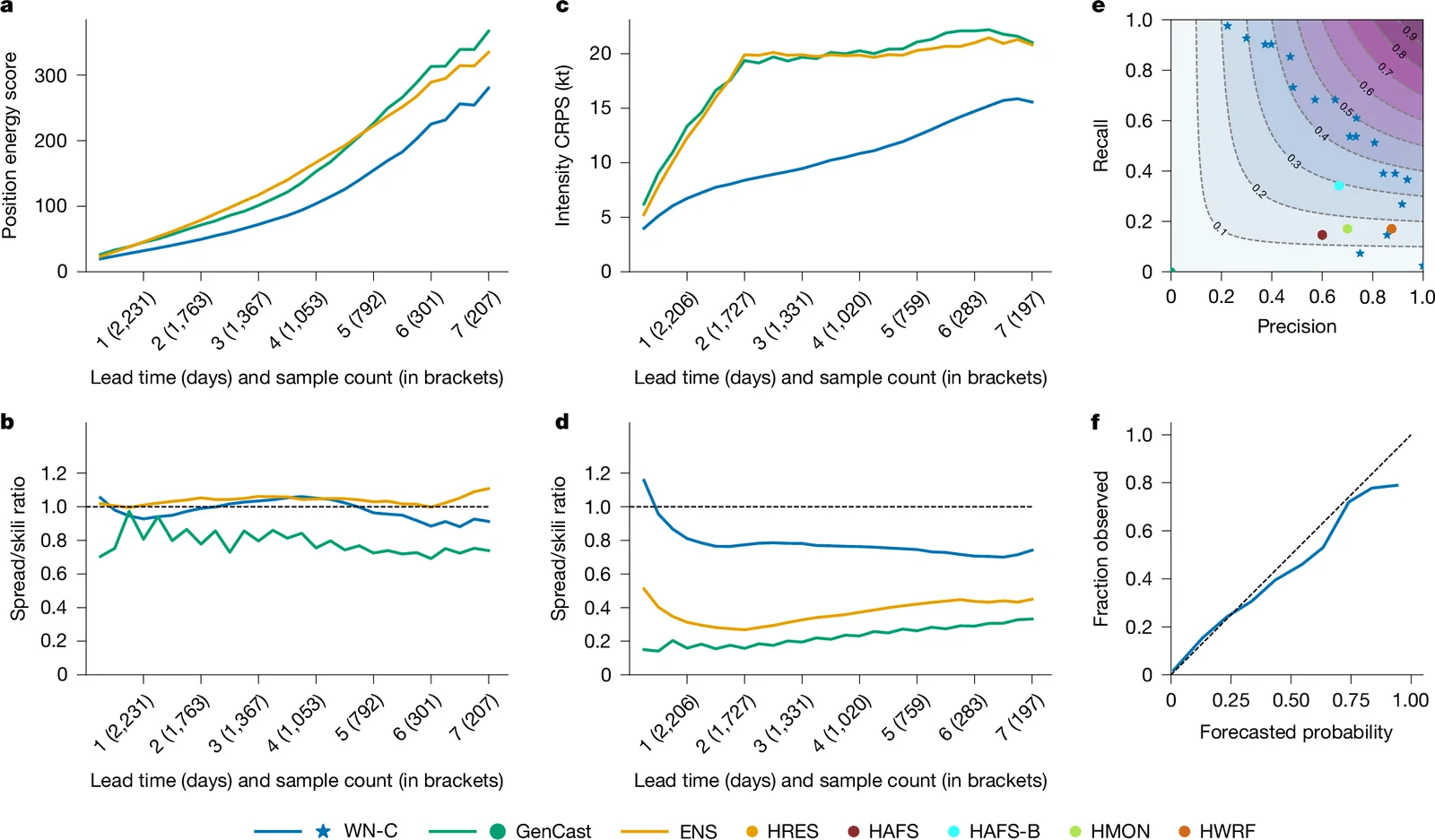
WN-C produces skilful and well-calibrated ensembles of cyclone track and intensity. Errors evaluated against IBTrACS best tracks^2,3^. Baseline data were obtained from NHC operational a-decks^43^. **a**, Position energy scores show that WN-C has an advantage of more than 1 day over previous probabilistic models. **b**, Track calibration is good, especially for WN-C and ENS. **c**, Intensity CRPS shows substantial probabilistic gains on intensities. **d**, Intensity calibration is generally quite poor, but WN-C’s is substantially better comparatively. In **a**–**d**, sample counts are shown in brackets for each lead time (*x* axis). **e**, When predicting rapid intensification (RI; intensity increase of at least 30 kt in 24 h), WN-C trades off precision and recall. No single model excels at RI, but WN-C is competitive with or better than other individual models across a range of precision–recall trade-offs, and substantially improves the critical success index, indicated by the background shading, from less than 0.3 to 0.5. Each star corresponds to a different probability threshold for classifying a prediction as RI; stars in the bottom right correspond to higher probability thresholds and stars in the top left to lower probability thresholds. The off-trend performance on the bottom right is due to the very low sample size for high-RI thresholds. **f**, WN-C’s predicted RI probabilities are generally reliable.

WN-C’s ensemble captures forecast uncertainty that is situation-dependent and well calibrated (Fig. 3b). This provides crucial information on forecast confidence. For example, the tight ensemble spread for Hurricane Milton correctly signalled high certainty about the landfall location days in advance (Fig. 1d), demonstrating the model’s utility for risk assessment.

To quantify the skill of these probabilistic cyclone track forecasts, we compute the position energy score, which is a generalization of CRPS for positions in three (or more) dimensions. It is defined in Supplementary Information section 1.3.3 and was found to be a strictly proper scoring rule, meaning that it is minimized in expectation when the predictive distribution being evaluated matches the ground-truth distribution. We observe in Fig. 3a that the probabilistic energy score results mainly follow those of the deterministic evaluations, with about a one-day advantage achieved by WN-C over ENS, and slightly less over GenCast.

Another crucial aspect is whether the forecasts exhibit the appropriate amount of uncertainty (ensemble spread), given the average size of their errors. A standard metric for assessing this property is the spread/skill ratio (see Supplementary Information section 1.3.4). A perfectly calibrated ensemble forecast would have a spread/skill ratio equal to 1; ratios less than or greater than one can indicate underdispersion (overconfidence) or overdispersion (underconfidence) on average, respectively. WN-C’s spread/skill ratio is close to 1 for most lead times, with a calibration that is similar to that of ENS and better than GenCast (Fig. 3b). We note that WN-C’s forecasts simultaneously achieve higher accuracy and tighter spread than those of ENS, resulting in a similar, well-calibrated spread/skill ratio.

## Intensity and wind radius forecasting

Although global models have improved steadily at track forecasting, predicting cyclone intensity—the maximum sustained wind speed—has remained a more challenging task^8^. Progress from conventional NWP models has been slow, and previous global AI models have struggled, often producing low-biased forecasts because they are trained on coarse analysis data that do not accurately match a cyclone’s true intensity^15^.

To assess intensity forecast skill, we perform a paired, homogeneous evaluation comparing WN-C against the best available deterministic and ensemble baselines. First, we compare to NOAA’s HAFS, a state-of-the-art, deterministic high-resolution regional model specifically designed to provide skilful intensity and structure forecasts. WN-C’s ensemble mean has a lower intensity error across all forecast horizons (Fig. 2b), with significant improvements from 0.5 to 3.25 days lead time. These results are consistent across ocean basins and observed cyclones (Extended Data Fig. 11 and Supplementary Information section 2.8). The 3-day mean intensity forecast of WN-C is 3.75 kt more accurate than that of HAFS—an improvement that matches the progress in intensity forecasting made by NWP models over the past 10 years (Extended Data Fig. 9 and Supplementary Information section 2.7.2). This result shows that a global AI model can achieve an intensity forecast skill comparable to that of specialized, limited-area models, without sacrificing the track accuracy inherent to global systems.

Second, we compare WN-C’s probabilistic intensity forecasts to those of ENS as well as to the debiased GenCast forecasts (see ‘GenCast intensity forecasts using a pressure–wind-speed relation’ in the Methods). We evaluate probabilistic skill using CRPS, and calibration using the spread/skill ratio. WN-C reduces the CRPS by more than 50% across many lead times, compared with ENS and GenCast (Fig. 3c). In terms of calibration (Fig. 3d), WN-C, while slightly under-spread, exhibits a spread/skill ratio substantially closer to 1 than the probabilistic baselines.

Closely related to cyclone intensity is its structure; that is, its size and shape. This is typically described by its wind quadrant radii. For a given wind-speed threshold, typically 34 kt, 50 kt or 64 kt, a quadrant radius is the maximum distance from the cyclone’s centre at which winds exceed the threshold, for the given quadrant. Predicting the extent of a cyclone is crucial for determining which areas far from the cyclone centre will be affected. Figure 2c shows that for 34-kt winds, WN-C attains significantly lower extent errors than do both ENS and HAFS.

### Rapid intensification

Among the greatest threats posed by tropical cyclones is the potential for rapid intensification (RI). Forecasting these explosive intensification events is a key unsolved problem in tropical cyclone forecasting.

We perform a homogeneous evaluation of RI occurring in the first 24-h period of a forecast. Predicting RI is a binary classification task, in which a model’s performance can be represented on a precision–recall curve that plots the fraction of correct RI predictions (precision; also referred to as success rate) against the fraction of true RI events captured (recall; also referred to as probability of detection). Whereas deterministic forecast models produce only a single prediction—a single point on the precision–recall plane—the probabilistic nature of WN-C allows us to sweep a decision threshold (minimum fraction of members predicting RI) to generate a full performance curve. The performance curve for WN-C improves on those of leading operational models, because it often achieves a better precision for the same level of recall. This indicates a better trade-off between correct detections and false positives in the Atlantic and East Pacific basins (Fig. 3e), improving the critical success index (CSI = TP/(TP + FP + FN), where TP, FP and FN denote true positive (correct RI predictions), false positive (false alarms), and false negative (missed RI events), respectively) from just below 0.3 to 0.5.

Because WN-C is a probabilistic model, it also enables decision makers to look at RI forecast probabilities themselves, rather than simply binary predictions that RI either will or will not occur. Figure 3f shows a reliability diagram, which plots the fraction of instances in which RI occurred out of all instances in which RI was forecasted with a given probability. A perfectly reliable forecast would generate a plot lying on the *y* = *x* diagonal. WN-C’s probabilistic RI forecasts are generally well calibrated, with the curve lying close to this diagonal.

This skill in predicting RI represents a crucial step towards providing forecasters with the reliable, early guidance needed to issue timely warnings ahead of a cyclone’s most dangerous phase.

## Wind probabilities and large ensembles

A crucial piece of information for decision makers is the probability of damaging winds at a specific location. Regional agencies such as the NHC and ECMWF provide wind-speed probability forecasts^33,34^ for meteorologists to issue hurricane or tropical-storm warnings in specific locations. We apply a deterministic mapping from ensembles of cyclone trajectories to gridded fields of wind-speed probabilities that a given location will experience winds exceeding 34 kt, 50 kt or 64 kt, either at a specific lead time, or cumulatively (at any time) within a lead-time window (Fig. 1b–d). This enables localized decision-making on a per-location basis.

Because each member of our ensemble represents a complete, physically plausible cyclone—with its own track, intensity and structure—we can directly compute the probability that a given location will experience winds exceeding a certain threshold (for example, higher than 65 kt), either at a specific lead time, or cumulatively (at any time) within a lead-time window. Note, though, that IBTrACS-derived wind radii are approximate, because actual winds are not strictly monotonic with distance from the centre; more precise assessments of per-location winds are left for future work.

**Fig. 4:**
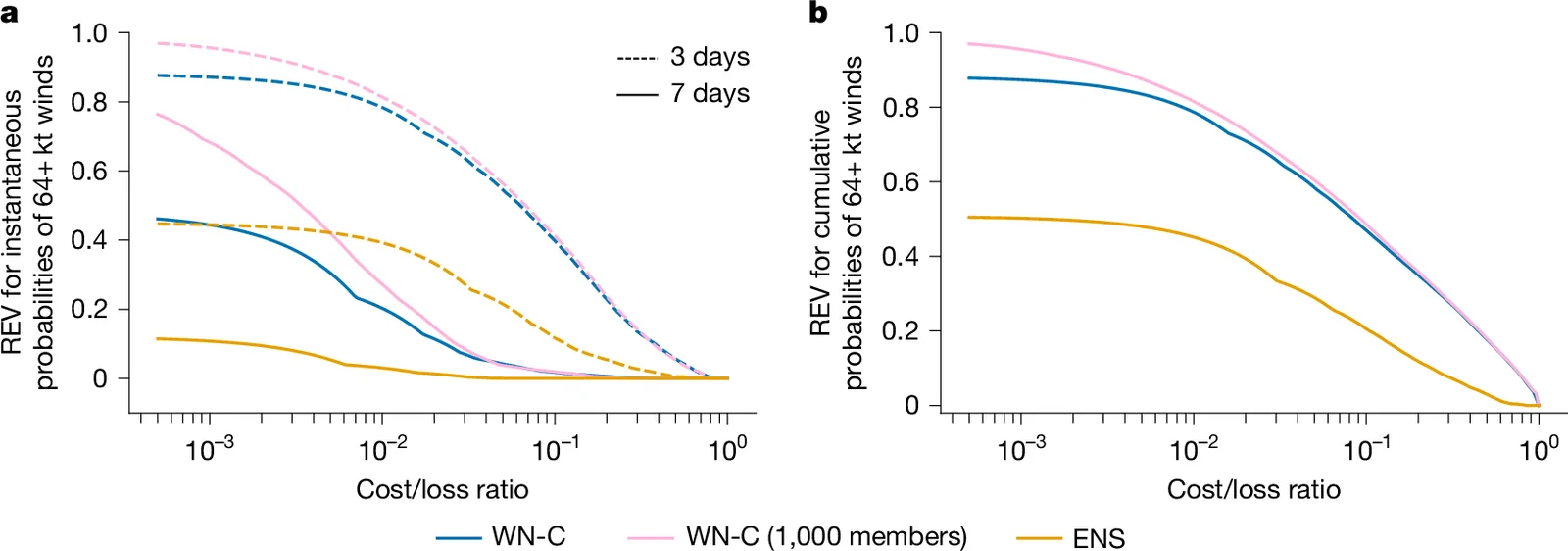
WN-C provides meaningful economic value and is improved by larger ensembles. We observe improvements in relative economic value (REV) over a wide range of cost/loss ratios for both instantaneous and cumulative wind-speed probabilities, as evaluated for 2023–2024 around the globe. **a**, WN-C improves the REV significantly across all cost/loss ratios for instantaneous probabilities of 64+ kt winds. Increasing the ensemble size from 50 to 1,000 members provides substantial value, particularly at longer lead times (7 days), where event probabilities require more samples to be accurately estimated. **b**, Similarly, WN-C improves the REV significantly over ENS for cumulative probabilities of 64+ kt winds (up to 10 days lead time), for all cost/loss ratios. Increasing the ensemble size from 50 to 1,000 members further increases REV, particularly for smaller cost/loss ratios. Predictions are evaluated against IBTrACS^2,3^. ENS tracks were obtained from the ECMWF archive^9^.

Faced with a forecast of some probability of extreme winds occurring at a given place and time, individuals, organizations and governments must decide whether to undertake potentially costly preparations to mitigate the potential losses if the extreme winds were to materialize. We quantify how well forecasts support such decisions using relative economic value^35^ (REV; Supplementary Information section 1.3.5). WN-C’s 50-member-ensemble forecasts of 64+ kt wind probabilities provide significantly more REV than do ENS forecasts across all cost/loss ratios, lead times and wind thresholds (Fig. 4, Extended Data Fig. 7 and Supplementary Fig. 9). WN-C’s raw probability forecasts are generally well calibrated (Supplementary Figs. 10 and 11), and substantially better calibrated than those of ENS, although slightly overconfident at higher thresholds.

Our AI-based approach is very computationally efficient. Generating a single forecast can be done roughly ten times faster than what has been achieved with previous AI models such as GenCast, and orders of magnitude faster than is possible with conventional NWP models, enabling very large ensembles in minutes that capture low-probability tail risks. Unlike previous large-ensemble cyclone outputs based on synthetic perturbations^36^, each WN-C member is an independent, physically complete 15-day weather trajectory.

Increasing the ensemble size from 50 to 1,000 members increases the REV substantially at low cost/loss ratios (Fig. 4). For instance, at a 0.001 cost/loss ratio, 1,000 members at 7 days provide more REV than do 50 members at 5 days.

## Valuable operational guidance

The ultimate test of a new forecasting system is its value in a live operational setting. During the 2025 Atlantic hurricane season, experimental forecasts from WN-C were provided to the NHC, and forecasters there have already begun incorporating its guidance into their official forecast process^37^. We have included an evaluation of WN-C forecasts for the 2025 season in Supplementary Information section 2.1.1. The rapid operational adoption of WN-C highlights its value as a complementary component in consensus ensembles—the diverse suite of models that forecasters use to build confidence and produce official forecasts and warnings.

**Fig. 5:**
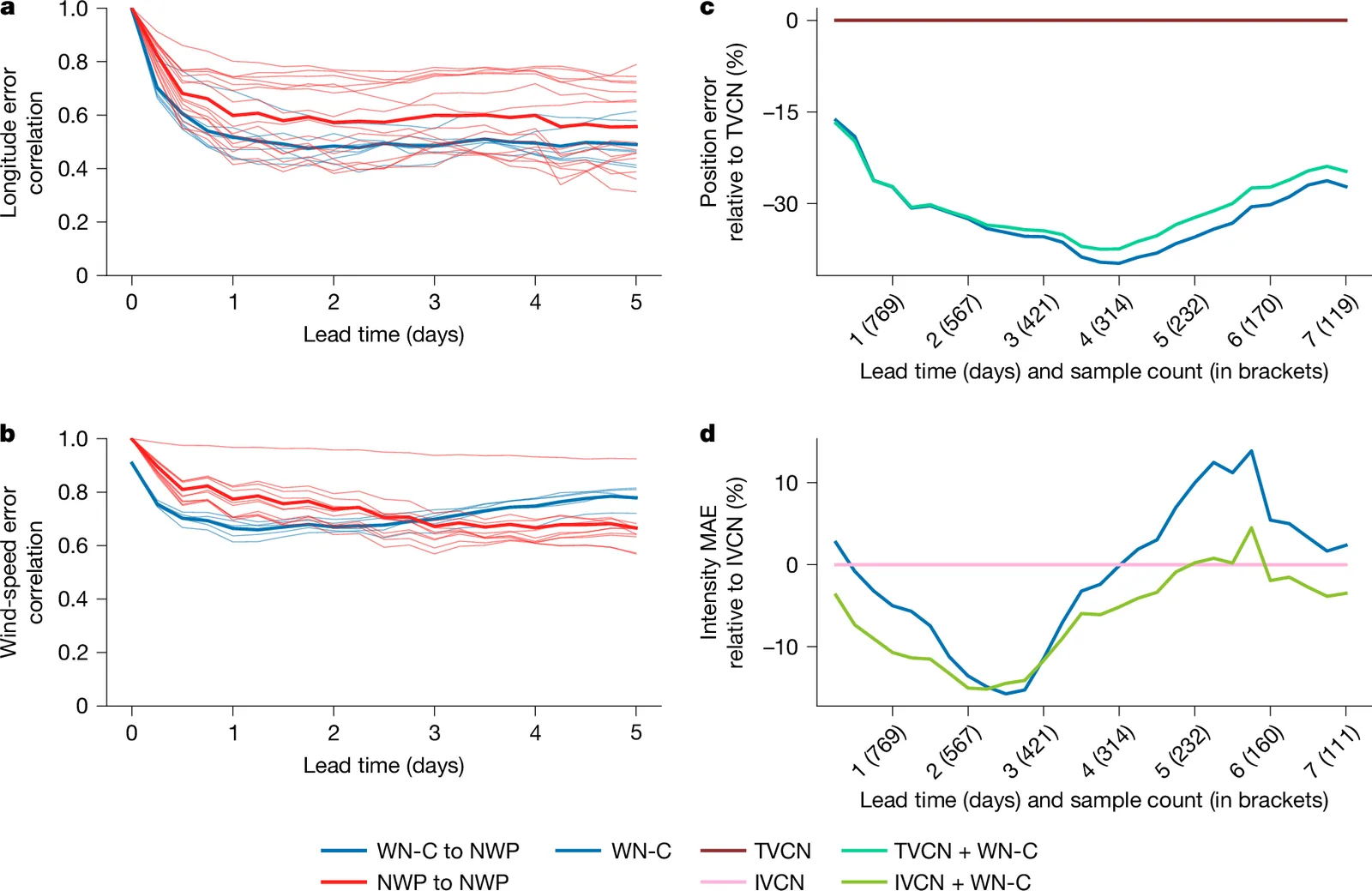
WN-C contributes substantial improvements to consensus ensembles. These improvements are a result of WN-C’s skill, as well as a low correlation with NWP models, evaluated against IBTrACS^2,3^. **a**, Correlation between longitude errors of WN-C with those of the constituent models of TVCN in blue, and the correlations of those constituent models with themselves in red (the average across all pairwise comparisons is in bold). **b**, Error correlations for maximum sustained wind speed, using the IVCN consensus. **c**,**d**, Homogeneous evaluation of WN-C on track error (**c**) and intensity error (**d**) against TVCN and IVCN, respectively, both with and without WN-C. Both WN-C on its own and TVCN + WN-C are substantially better at track forecasting than TVCN is. WN-C outperforms IVCN at early lead times and is worse at long lead times. IVCN + WN-C outperforms IVCN at lead times up to 5 days. In **c**,**d**, sample counts are shown in brackets for each lead time (*x* axis). TVCN and IVCN consensus data were obtained from the NHC operational a-decks^43^.

The value of adding a new model in a consensus is driven by two factors: its stand-alone skill and its low error correlation with existing models^38,39^. As established in the preceding sections, WN-C produces highly skilful track and intensity forecasts. Crucially, it also provides forecasts that are independent from existing guidance models. The error correlation between our model and operational NWP systems is comparable to and often lower than the high intercorrelation among the NWP models themselves (Fig. 5a,b). This low correlation means that our model typically has different failure modes to conventional models, making it a valuable additional source of guidance for forecasters.

To quantify this value, we simulated adding WN-C to a modified version of the track variable consensus (TVCN) and its intensity counterpart (IVCN), which are operational consensus ensembles produced by the NHC. Each of them comprises a set of early guidance models, which are averaged to produce a consensus mean^40,41^. Our key modification is to perform a weighted average of TVCN (or IVCN) with WN-C, with consensus weights optimized on our validation set of 2022 cyclone tracks on the North Atlantic and East Pacific basins. We tune the weights to minimize mean absolute error (MAE) either in latitude and longitude, or in intensity. This is important to maximize the benefit of WN-C to the consensus, because its optimal weight is often much bigger than the uniform weight (for full details, see ‘Details of the consensus ensemble method’ in the Methods. We keep weights constant across lead time and basins that is, in each of the two consensus models, we optimize one scalar weight for WN-C and another for TVCN or IVCN. For track forecasting (Fig. 5c), adding our model improves the consensus by about 18% to 38% depending on lead time, with an average improvement of 28%. On intensities (Fig. 5d), IVCN + WN-C shows appreciable improvements over IVCN on the order of 5% to 14%, depending on lead time, with an average improvement of 6%. We emphasize that this improvement indicates that conventional NWP models still provide substantial value when modelling tropical cyclone intensity. These results show that integrating our AI model into the existing operational suite leads to immediate and substantial gains in forecast accuracy, ultimately enhancing the tools available to protect lives and property.

## Conclusion

This paper introduces WeatherNext Cyclones (WN-C), an AI system that addresses a long-standing challenge in tropical cyclone forecasting. By training a probabilistic global model on both atmospheric analysis and a dedicated cyclone database, our system bridges the historical performance gap between the track skill of global models and the intensity skill of high-resolution regional models. Specifically, its track forecasts provide more than 1 day of lead-time advantage over the best global models, while simultaneously delivering intensity predictions superior to those of specialized models such as HAFS—a capability previously beyond the reach of global AI systems.

Our model uses an input resolution even coarser than that of global models, and orders of magnitude coarser than the fine meshes of regional models. This shows that high resolution is not essential for accurate forecasting of cyclone intensity. Instead, it suggests that large-scale atmospheric data contain more intensity signal than previously recognized, opening new challenges and opportunities for the field.

We ran WN-C live from June 2025 to the present, disseminating predictions publicly and to the NHC. Its forecasts have been rapidly adopted by forecasters at the NHC and other World Meteorological Organization tropical cyclone forecast centres^42^.

Despite these advances, the chaotic nature of the atmosphere ensures that tropical cyclone forecasting remains a challenging and fertile field for AI research. Expanding these models to predict cyclone-induced precipitation, storm surge and precise wind gusts offers clear avenues for future investigation. Moreover, although a reliance on high-quality analysis has been key to our success, future progress will also require moving towards end-to-end systems that are capable of directly assimilating raw observations, such as those collected in real time by operational centres.

The success of this research also precipitates questions about the use of machine learning for predicting extreme weather. Our results challenge the conventional view that machine learning performs poorly on extreme events. By using probabilistic training, we avoid the spatial smoothing problems that plague deterministic models^15^, and co-training on extreme and analysis weather data effectively bypasses the data scarcity of extreme events. These findings underscore the value of long-term investments in high-quality datasets such as IBTrACS. As AI methods rise to the challenge of forecasting Earth’s most extreme weather, they unlock opportunities to use a wider range of observations and impact data. This framework is not limited to tropical cyclones; it creates a blueprint for modelling other high-impact phenomena—from localized heat extremes to extreme precipitation—heralding a shift to AI-driven environmental predictions that will help build safer, more resilient societies.

## Methods

### Generation of cyclone data and tracking algorithm

**Extended Data Fig. 2:**
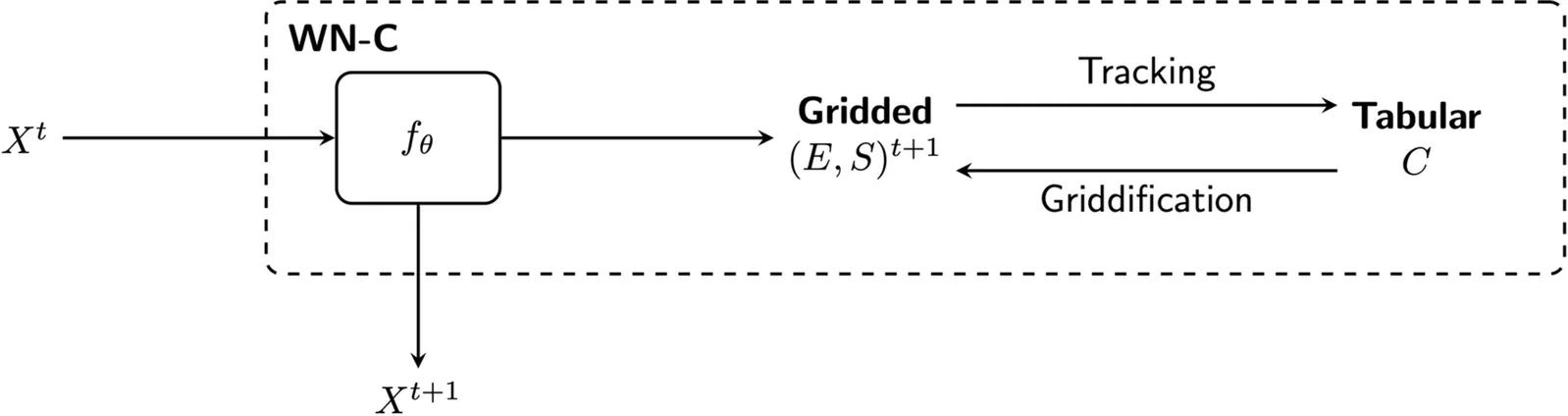
Autoregressive state transition and mapping between prediction spaces. The WN-C neural network *f*_θ_ receives atmospheric state data *X*^*t*^ as input and jointly predicts* X*^*t*+1^ and gridded cyclone targets (*E*, *S*)^*t*+1^. WN-C encapsulates the bidirectional transformations between these grids and the discrete tabular trajectories *C*.

To train WN-C end to end on sparse, tabular IBTrACS data, we apply a deterministic map from the cyclone trajectory data into gridded tensors on the 0.25° grid. We apply this map to IBTrACS to obtain gridded training data, which the neural network *f*_*θ*_ learns to predict. When the trained model generates gridded cyclone field predictions, we apply a tracking algorithm to map back to tabular cyclone trajectory space. This framework separates the differentiable prediction of cyclone presence and properties from their (non-differentiable) tracking, and ensures that a predicted cyclone’s existence, location and physical properties are all governed by the same learned representations. The autoregressive state transitions and bidirectional mappings between these prediction spaces are summarized in Extended Data Fig. 2. We describe the cyclone griddification, training and tracking algorithms in this section.

### Griddification of cyclone best-track data

The griddification process creates two main types of target fields at 0.25° resolution. If at time *t* there are *N*_*t*_ active cyclones with latitude–longitude coordinate pairs ${r}_{t1},\ldots ,{r}_{t{N}_{t}}$ and *K* corresponding scalar variables ${s}_{{kt}1},\ldots ,{s}_{{kt}{N}_{t}}$, the fields are defined as:

1. Existence probability (*E*): for each cyclone in the IBTrACS data, we generate a two-dimensional field representing the probability of the cyclone’s centre being at each grid cell by placing Gaussian kernels at the true cyclone locations:
  $${E}_{{tij}}=\mathop{\sum }\limits_{n=1}^{{N}_{t}}\exp \left(-\frac{g{({r}_{{ij}}^{{\prime} },{r}_{{tn}})}^{2}}{2{{\ell }}^{2}}\right)$$
  where ${r}_{{ij}}^{{\prime} }$ is the coordinate pair at grid point (*i*, *j*) and $g(\cdot ,\cdot )$ is the geodesic distance. We set ${\ell }$ = 120 km, scaled to a peak value of 1.0. This method, inspired by computer vision^22^, encourages the model to learn precise locations.
2. Conditional scalars (*S*): we create separate gridded fields for each scalar property (for example, maximum sustained wind speed, wind radii). The target value is assigned to all grid cells within the 120-km radius (${\ell }$) of the true centre, and is otherwise masked (NaN):

$${S}_{tij}=\left\{\begin{array}{cc}{s}_{tn} & \,{\rm{i}}{\rm{f}}\,g({r}_{{ij}}^{{\prime} },{r}_{{tn}}) < {\ell }\,\mathrm{for}\,\mathrm{some}\,n\\ {\rm{N}}{\rm{a}}{\rm{N}} & \,{\rm{otherwise}}\end{array}\right.$$

Occasionally, IBTrACS contains missing values for the scalar cyclone properties (for example, when the best-track data curators at operational centres were unable to determine a maximum sustained wind speed from available observations of a cyclone). For maximum sustained wind speed, minimum pressure and radius of maximum winds, we leave the corresponding *s*_*ktn*_ entries as NaN. This implies that the corresponding scalar channel is also set to NaN in the vicinity of the corresponding cyclone location *r*_*tn*_, so the model is not trained on these targets. For quadrant radii, we adopted the following imputation strategy. Before gridding the quadrant radius data, we imputed the quadrant radius scalars *s*_*tn*_ for each cyclone and quadrant threshold. If at least one of the four quadrants is non-NaN, we set any NaN quadrants equal to the mean of the non-NaN quadrants. If all four quadrants for a given threshold are NaN, we set all of them to 0.

### Model training for cyclone variables

The IBTrACS-derived gridded cyclone fields (*E*, *S*) are concatenated with the core atmospheric state variables (*X*; Supplementary Table 2) and incorporated into the model’s multi-stage training process (Supplementary Table 1). The same fair CRPS (fCRPS) loss function used for atmospheric variables (see ‘Reparameterization trick and CRPS marginal training’) is applied to the IBTrACS output channels.

A key aspect of the training is the use of masking for the conditional scalar losses. The fCRPS loss for each conditional scalar field is only computed and backpropagated for grid points located within the 120-km-radius discs centred on the ground-truth cyclone locations. The loss is masked to zero for all grid points outside these discs. Note that we do not normalize by the number of targets, so a target date with two cyclones in it will contribute twice as much as a date with no cyclones in it; that is, every cyclone is assigned the same weight during training. The existence probability field, however, is trained over the entire globe, because we use its predictions everywhere. These modelling choices encourage the model to learn to predict cyclone properties such as intensity and structure conditionally on the presence of a predicted cyclone at that location, as indicated by the existence probability field, and to ignore predicting cyclone scalar properties in cyclone-free regions. We include all storms in the training data, regardless of the storm stage of development (for example, subtropical, tropical, extratropical and so on).

### Cyclone tracking algorithm

At inference time, the model outputs predictions for the dense gridded fields, including the cyclone existence probability (*E*) and the conditional scalar fields (*S*). To extract meaningful cyclone tracks in the familiar tabular format, we apply a custom greedy tracking algorithm (the ‘direct tracker’). We use ‘learned tracker’ to refer to the application of the heuristic direct tracking algorithm to the model’s gridded cyclone outputs. The direct tracker operates as follows:

1. Initialization: cyclone tracks can be initialized either from known cyclone centres at the analysis time (*t* = 0)—which are sourced from IBTrACS in our historical evaluations—or through a cyclogenesis process (see below).
2. Propagation: the tracker advances all active cyclones in 6-h increments; that is, all tracks are propagated by an increment in the same step rather than propagating a single track to completion before starting the next track. For each active cyclone centre, we repeat the following steps.

- Momentum-based initial guess: to advance the cyclone’s position at the next time step, we first use a momentum-based update to make an initial guess. The momentum update extrapolates the position of the cyclone along the great circle that joins the current and previous positions of the cyclone, approximating Earth as a perfect sphere, and using an extrapolation factor of 0.9. This factor was roughly tuned on historical IBTrACS data to optimize the accuracy of this initial guess. If no previous position is available, that is, if this is the first time step of the cyclone, no momentum is applied.
- Mode-then-mean refinement: the prediction is then refined by analysing the predicted existence probability field in the vicinity. We use a ‘mode-then-mean’ refinement strategy: first, the grid point with the highest probability within a search radius of 250 km is identified (mode), and then a probability-weighted average position is calculated within a radius of 250 km around this mode to determine the new centre. The probability-weighted average is computed in three-dimensional Cartesian coordinates and the resulting point is projected back to Earth’s surface. The performance is not very sensitive to the exact values of these hyperparameters. Intuitively, their values have to be comparable to the 120 km used for training, but they also can’t be too large, to avoid merging two cyclones.
- Dissipation: after obtaining refined estimates for the next centres, we apply a dissipation step, in which a track is terminated if the mean existence probability within a disc of radius 500 km around the cyclone centre falls below a dissipation threshold of 10^−5^. This threshold was tuned from a coarse grid search on the 2022 validation set.
- Pruning: when advancing existing cyclone centres, one undesirable edge case that can occur is that distinct centres can coincide if during the mode-estimation step the tracker happened to advance two centres to the same maximum. To handle this, we apply a pruning operation that searches for cyclone centres that are within 500 km of each other, and remove the one that moved the largest geodesic distance in this step.

3. Cyclogenesis: after pruning, the tracker has evolved all existing centres but it must also handle the genesis of new cyclones at this time step; that is, cyclogenesis. To do this, the tracker first identifies candidate cyclogenesis centres by splitting up the global predicted existence probability field into 5° × 5° latitude–longitude squares and filtering for those in which the maximum probability of existence exceeds a threshold of 0.3. We set this threshold based on the fact that the ground-existence kernels are unit-mode Gaussians, so this heuristically selected threshold quickly rules out candidates with low existence probabilities. The purpose of this step is to quickly filter out a large number of regions in which cyclone activity is low, and speed up the search. The locations of the existence probability maxima from the surviving latitude–longitude squares are looped over in order of decreasing maximum probability of existence. Each candidate centre is refined by computing a weighted average of the existence probability field, within a disc of radius 250 km centred at the candidate. Similar to the propagation step, the refined candidate is kept only if it is at least 500 km away from any already-existing cyclone centres, and if the mean existence probability within a 500 km radius is above 10^−5^, and is discarded otherwise. This procedure is repeated until all initial cyclogenesis candidates have been exhausted. This treatment means that we are effectively treating the existence Gaussian kernels as a proxy for cyclone activity and genesis.
4. Scalar extraction: once the new active centres have been identified, including new cyclogenesis centres, the scalar properties (intensity, radii and so on) are extracted from the corresponding conditional scalar fields (*S*) predicted by the model. For this, we apply bilinear interpolation to the gridded predictions, at the predicted cyclone centres.
5. Repeat propagation, cyclogenesis and scalar extraction: the propagation and cyclogenesis steps above are repeated until the final lead time of the forecast has been reached.
6. Cyclogenesis minimum duration criterion: cyclone tracks produced via cyclogenesis by the tracker are filtered out if they last 2.5 days or less. This is to remove spurious short-lived tracks that are not sustained for long enough to be a physically plausible tropical cyclone.
7. Enforcing physical consistency: once all tracks have been produced by the tracker, a final step is applied to ensure the scalar variables respect certain physical constraints (non-negativity of all variables, monotonicity of quadrant radii across thresholds, and that quadrant radii are at least as large as the radius of maximum winds when the maximum wind speed exceeds the quadrant threshold). These constraints are enforced by post-hoc clipping.

We note that, unlike the TempestExtremes algorithm^44^, the greedy operation of the direct tracker means that there is no need for a separate ‘stitching’ step to join the centre of a given cyclone across different lead times. This is automatically handled because the advancing step only ever advances the centre of an already-existing cyclone.

We also note that, in principle, the direct tracker can produce jumps in a given track. For example, if the tracker is following a kernel associated with a given cyclone (A), and there is another kernel nearby associated with a different cyclone (B), the tracker could in principle jump from one to the other. The main failure mode of this kind that we observed at first was merging: occasionally while tracking A and B, and if B is at the end of its lifetime, instead of dissipating the associated track, the tracker jumps from B onto A, and continues to track both tracks as active even though after the jump they coincide exactly. This issue is addressed by the pruning operation in the propagation step.

To test that the direct tracker robustly inverts the griddification step (see ‘Griddification of cyclone best-track data’), we applied the tracker to the ground-truth griddified IBTrACS data (instead of the predictions of the model). We found that it produced tracks that closely matched the ground truth to position errors of up to a few tens of kilometres for the vast majority of cases. Some relatively small errors in position occur even when using ground-truth data, which can be attributed to the mean-refinement step’s probability-weighted average computation approximating Earth as a perfect sphere instead of an ellipsoid, which introduces a small error. We found that tracker jumps were rare, around 1% of cases, and that the contribution of this source in the average position error was negligible. Otherwise, we have found that the aforementioned steps are sufficient for accurate tracking.

#### Tuning the hyperparameters of the direct tracker

We tuned the direct tracker’s hyperparameters through a grid search. We ran the tracker on gridded forecasts on the 2022 validation set and evaluated paired deterministic and probabilistic metrics on tracks, intensity and quadrant radii, as a function of different tracker parameters. The parameters we optimized were the mode-then-mean refinement radius, the dissipation radius and whether or not to perform pruning, as well as the radii for pruning and cyclogenesis. For scalar extraction, we also investigated using integrals of the scalar fields as weighted by the existence field, but found that bilinear interpolation worked better, particularly in reducing under-spreading in the scalar predictions. Although there is the potential for competition between the lead times and the metrics considered, we found that the optimized hyperparameters brought consistent benefits among the grid search candidates without the need for a trade-off. Finally, the momentum factor was roughly tuned on historical IBTrACS data to optimize the accuracy of this initial guess.

### Functional generative networks

Our prediction problem is probabilistic owing to multiple uncertainties associated with weather and cyclone forecasting. First, there is the epistemic uncertainty about the model, which arises due to finite training data and an imperfect learning algorithm. To model this, we adopt a common strategy known as deep ensembles^24^, in which we train multiple models from independently initialized parameters, and then later combine their predictions into an ensemble. Second, there is aleatoric uncertainty because of the imperfect information in the initial state provided to the model: weather analysis states comprise estimates of the true weather state on a finite grid, which do not exactly reflect the true weather state. Although this uncertainty is small at early lead times, it grows because of the chaotic nature of the weather system, causing larger uncertainty at longer lead times.

To capture both uncertainties, we propose FGNs, a simple, scalable probabilistic modelling approach that expresses uncertainty through functional variability. FGNs learn to model the joint distribution over the outputs while training on a loss that applies only to the marginal distributions, in a suitably constrained setting.

Concretely, we model different forecast samples ${X}_{i}^{t}$ of $p({X}^{t}|{X}^{t-2:t-1})$ as coming from neural networks ${f}_{{\theta }_{i}^{t}}$ whose parameters ${\theta }_{i}^{t}$ are sampled from a learnt distribution. The model is trained using the fCRPS as our training objective^23^. At each training step, a two-member ensemble is generated, and the fCRPS is computed as the loss on this small ensemble.

At first, WN-C is trained on a ‘single-step’ loss predicting only the first (6 h) lead time. The following training stages add autoregressive rollouts as part of each training step, in which the model produces a number of forecast steps autoregressively, being fed its output as input, predicting lead times up to 48 h. There, the loss is averaged over all rollout steps, with gradients propagated back through the rollout.

Each of the constituent models in the WN-C model ensemble is implemented with a very similar neural network architecture to that of the denoiser in GenCast^14^ (see ‘Model architecture’ for details). Because the new model requires only one neural-network function call, compared with the 40 of GenCast, we also increase its size, from around 57 million to around 180 million parameters, while still being 8 times faster to sample. Furthermore, the new probabilistic approach easily handles sparse targets simply by not computing the loss when the targets are not present. This would have been harder in frameworks like autoencoders^45^ or diffusion^14^ that use the target as input during training.

### Modelling epistemic uncertainty with multi-model ensembling

One can account for epistemic uncertainty by drawing samples from the posterior predictive distribution,

$$\begin{array}{c}p({X}^{1:T}|{X}^{\le 0},{\mathcal{D}})=\int p({X}^{1:T}|{X}^{\le 0},{\mathcal{M}})p({\mathcal{M}}|{\mathcal{D}}){\rm{d}}{\mathcal{M}}\end{array},$$

which marginalizes over some posterior uncertainty about the model ${\mathcal{M}}$ conditional on our training dataset ${\mathcal{D}}$. We adopt a common approximation known as deep ensembles^24^, in which we train multiple models ${{\mathcal{M}}}_{1},\ldots ,{{\mathcal{M}}}_{J}$ from independently initialized parameters, and then later combine their predictions into an ensemble. Its connection to Bayesian inference has been studied elsewhere^46,47^, but here we apply it informally to approximate Bayesian inference.

In this work we train *J* = 4 models, a trade-off between performance and training cost. To combine their predictions into an ensemble, we generate an equal number of ensemble member trajectories from each model, using the same model for all time steps of a trajectory.

### Modelling aleatoric uncertainty with learned functional perturbations

The weather states we model from ECMWF Reanalysis v.5 (ERA5) and operational HRES-fc0 do not fully capture the state of the weather system. More precisely, the coarse 0.25° spatial representation leaves sub-grid processes unresolved, and the resulting forecast uncertainty grows over time through chaotic error amplification. It is common to use stochastic dynamics to model this uncertainty due to unresolved phenomena^48,49^, and from the perspective of the model this can be viewed as aleatoric uncertainty.

We incorporate this aleatoric uncertainty in our model by sampling functions. Concretely, we model different forecast samples ${X}_{i}^{t}$ of $p({X}^{t}|{X}^{t-2:t-1})$ as coming from neural networks ${f}_{{\theta }_{i}^{t}}$ whose parameters ${\theta }_{i}^{t}$ are sampled from a learned distribution: ${X}_{i}^{t}={f}_{{\theta }_{i}^{t}}({X}^{t-2:t-1})$, where ${\theta }_{i}^{t} \sim {{\mathcal{P}}}_{{\mathcal{M}}}(\theta )$ independently for each ensemble member *i* and time step *t*.

A previous study^50^ justifies why modelling aleatoric uncertainty in parameter space can lead to more structured variations than can modelling it in input or output space. This structure is due to the reuse of sampled parameters across spatial (or other relevant) dimensions in architectures with parameter-sharing layers. Similar insights have been leveraged in reinforcement-learning exploration^51^, generative adversarial networks^52^ and test-time adaptation^53–56^.

Modelling uncertainty through parameter perturbations has a long history in NWP stochastic physics^48,49,57^ and multi-model superensembles^58^. Other AI weather models have explored spatially dependent perturbations trained with CRPS-like objectives^13,59^, although these can introduce high-frequency artefacts that affect performance and require mitigation by modifying the fCRPS loss or convolutional filters.

### Reparameterization trick and CRPS marginal training

To learn the distribution ${{\mathcal{P}}}_{{\mathcal{M}}}(\theta )$, we use the well-known reparameterization trick^60–63^: $\theta ={\theta }^{* }+\Delta \cdot {\epsilon }$, where Δ is a potentially structured matrix used to map Gaussian noise ${\epsilon }$ to a perturbation in *θ* space. ${\mathcal{M}}:= \{{\theta }^{* },\Delta \}$ thus consists of ${\theta }^{* }$, acting as the mean θ and describing a deterministic function, and a matrix Δ controlling the covariance. At inference time, we make the modelling assumption that we can compose both types of uncertainty by sampling one of multiple ${{\mathcal{M}}}_{j}$ trained independently, and then sampling ${{\epsilon }}_{i}^{t}$ to get ${\theta }_{i}^{t}={\theta }_{j}^{* }+{\Delta }_{j}\cdot {{\epsilon }}_{i}^{t}$.

We use the CRPS as our training objective. For a cumulative density function (CDF) *F* and target $y\in {\mathbb{R}}$,

$$\begin{array}{c}{\rm{CRPS}}(F,y)={\int }_{-\infty }^{\infty }{(F(z)-{\bf{1}}[z\ge y])}^{2}{dz}.\end{array}$$

For an empirical distribution coming from samples ${x}^{1:N}$ this is equal to: $\frac{1}{N}\sum _{n}|{x}^{n}-{y|}-\frac{1}{2{N}^{2}}\sum _{{n,n}^{{\prime} }}|{x}^{n}-{x}^{{n}^{{\prime} }}|$. However, this is a biased estimator of the CRPS of the underlying distribution *F*. For an unbiased CRPS estimate we can use the ‘fair’ CRPS estimator^64^,

$${\rm{fCRPS}}({x}^{1:N},y)\frac{1}{N}\sum _{n}|{x}^{n}-{y|}-\frac{1}{2N(N-1)}\sum _{{n,m}^{{\prime} }}|{x}^{n}-{x}^{{n}^{{\prime} }}|.$$

In practice, during training we use *N* = 2 ensemble members, which is the minimum required for an unbiased estimate of the fCRPS^65^. Using more members would increase memory cost linearly with negligible benefit to the loss gradient. At inference time, we generate 50 or more members to fully characterize forecast uncertainty.

We average the training loss over all locations, variables and levels,

$$\begin{array}{c}{\mathcal{L}}:= \frac{1}{|D|}\sum _{d}\frac{1}{G}\sum _{i}{a}_{i}{\rm{fCRPS}}({x}_{i,d}^{1:2},{y}_{i,d})\end{array},$$

where *d* indexes into the dataset batch *D*, *i* indexes into the *G* variable-level-location tuples and *a*_*i*_ is a per-variable loss weight. For standard weather variables, these weights mainly follow a previous study^11^: 1.0 for most pressure-level fields and 2-metre temperature, and 0.1 for other surface fields. We only half geopotential to 0.5 because we observed overfitting. The cyclone-specific fields, existence probability, maximum wind speed and minimum pressure receive a weight of 10, whereas the radius of maximum winds and quadrant wind radii are weighted at 1. Note that cyclone weights are not directly comparable to regular fields because the variables are quite sparse and enter during fine-tuning.

A number of other works have also included or modified the CRPS objective when training AI-based weather models^13,59,66,67^. Our approach is also closely related to work on scoring rule minimization^68^, DISCO Nets^69^ and MMD nets^70^, where ensemble models are also trained with CRPS from noisy inputs. However, these latter works typically do not yield state-of-the-art joint distributions in high-dimensional problems. In this work, we show that modelling the noise in parameter space rather than the original inputs or outputs can be a simple, effective method to learn skilful joint distributions in a very high-dimensional setting, given a suitably strong algorithmic inductive bias.

### Architecture and training

#### Model architecture

Each of the constituent models in the WN-C model ensemble is implemented with a very similar neural network architecture to that of the denoiser in GenCast^14^, with a graph neural network (GNN) encoder/decoder mapping from the latitude/longitude grid to a latent space defined on a spherical six-times-refined icosahedral mesh, and a graph-transformer processor that operates on the nodes of this mesh.

There are key differences, however, between the WN-C and GenCast architectures. WN-C is a larger model, with around 180 million parameters per model seed, compared with GenCast’s around 57 million in total. WN-C has a latent dimension of 768 and 24 layers in its graph-transformer processor, compared with the latent dimension of 512 and 16 layers in GenCast, and WN-C produces forecasts with a 6-h time step, whereas GenCast has a 12-h time step. A number of other minor differences in the encoder architecture are outlined in Supplementary Information section 1.1. We can ‘afford’ increasing the size and resolution of our model thanks to predictions that require only a single neural-network function call instead of 40.

One key modelling choice was to use conditional normalization—which in GenCast is used to condition on the corresponding diffusion noise level *σ*—as the means of perturbing the model. To be precise, in WN-C a global noise vector $z \sim {\mathcal{N}}{(0,1)}^{32}$ is passed into all of the network’s conditional layer-norm layers, such that sampling different vectors *z* for each ensemble member is what generates the variance across the ensemble.

This approach has two important features: (1) a low-dimensional noise source, which (2) is globally applied across all layers, and with learned conditional normalization parameters that are shared across the spatial dimensions of the model (that is, mesh and grid node dimensions). Together, these constrain the output distribution in a way that encourages the model to generate globally coherent variability despite being trained on a marginals-only loss.

We also note that conditional normalization has had success in other contexts for semantically coherent functional modulation; for example, in diffusion to condition on noise levels, as noted above, or in generative models of images and audio to condition on style or voice^71,72^. One group^59^ also uses conditional normalization to inject stochasticity into their weather model, but does so with location-specific conditioning, which adds a high spatial frequency component to the noise injection.

### Training methodology

WN-C is trained in a multi-stage process to optimize the CRPS.

#### Training data

We use two primary data sources: First, the global analysis: 40 years (1979–2018) of ECMWF’s ERA5 reanalysis data^1^ as well as ECMWF’s operational HRES-fc0 analysis from 2016 onwards (see Supplementary Information section 1.4 for more details). This provides a comprehensive dataset of global atmospheric states for learning fundamental weather dynamics. Second, the cyclone database: a specialized dataset of nearly 5,000 observed tropical cyclones from the IBTrACS database^2,3^, spanning 45 years. This provides targeted information on the track, intensity (maximum wind speed, minimum sea-level pressure) and size (radius of 34, 50, and 64-kt winds) of historical storms.

#### Evaluation protocol

During development, we trained WN-C on data from 1979 to 2021 and used 2022 as a validation year for all design choices. After freezing the model design and training recipe, we evaluated weather predictions on 2023 data and cyclone predictions on 2023–2025 data. For each calendar year of evaluation, the model was retrained using the frozen protocol on all data up to the end of the preceding calendar year, ensuring strict temporal separation between training and test sets.

#### Training schedule

The training proceeds in several stages, starting with pre-training on the large ERA5 dataset to learn general atmospheric patterns, followed by fine-tuning on a mix of ERA5, HRES-fc0 and the dense cyclone-specific IBTrACS data, which we refer to as direct cyclone targets (DCT; see ‘End-to-end cyclone forecasting’ in the main text), to specialize the model for cyclone forecasting. The final stages involve autoregressive training, in which the model is unrolled for multiple steps and gradients are backpropagated through time to improve long-range stability and accuracy. The necessity of this co-training for overcoming the intensity limitations of pure-analysis models is quantified in our ablation studies (Extended Data Fig. 4 and Supplementary Figs. 7 and 8).

Stage 1: 400,000 steps with a 1° input and ground-truth ERA5 dataset at a 12-h temporal resolution.

Stage 2: 100,000 steps with a 1° input and ground-truth ERA5 dataset at a 6-h temporal resolution.

Stage 3: 32,000 steps with a 0.25° input and ground-truth ERA5 dataset at a 6-h temporal resolution.

Stage 4: 5,000 steps with a 0.25° input and ground-truth ERA5 dataset, as well as DCT at a 6-h temporal resolution. In this stage, only weights in the final linear layer of the model corresponding to the dense cyclone targets are optimized. The rest of the model weights remain frozen.

Stage 5: 20,000 steps with a 0.25° input and ground-truth ERA5 dataset, as well as DCT at a 6-h temporal resolution. In this stage, the model is trained end to end.

Stage 6: 18,000 autoregressive steps with a 0.25° input and ground-truth HRES-fc0 dataset, as well as DCT at a 6-h temporal resolution. This consists of 8,000 steps at 1AR, 4,000 steps at 2AR and 1,000 steps at 3AR–8AR, where the model is trained end to end in all cases.

In all cases, the model is trained with a batch size of 64 using the AdamW optimizer^73^ with weight decay 0.1 and a cosine learning rate schedule with linear warmup. A full list of hyperparameters used for the stages is provided in Supplementary Table 1.

We subsample the 6-hourly data with a stride of two to generate the 12-hourly dataset. Accumulated variables, such as total precipitation, are accumulated over the longer interval accordingly. Similarly, we downscale ERA5 from 0.25° to 1° by subsampling the spatial dimensions with a stride of four.

When training on 1° data, the graph-transformer processor operates on a five-times-refined icosahedral mesh. This is further refined a sixth time in the stages training on 0.25° data, in which the architecture is updated to input and output the higher-resolution data, but using the same model weights. To preserve the scale of the incoming signal at the start of stage 3, we account for the increase in the number of messages being received by each mesh node and divide the sum of the message vectors by 4 in the encoder GNN.

#### Computational resources

Training WN-C takes approximately 4 wall-clock days per model seed, using a combined total of 560 tensor processing unit (TPU) v5p and TPU v6e days of compute. For inference, generating a single 15-day forecast trajectory takes just under 1 min on a single TPU v5p, and every ensemble member can be generated in parallel.

### GenCast intensity forecasts using a pressure–wind-speed relation

One common challenge with previous machine-learning-based cyclone-forecasting models is that they produce low-biased intensity forecasts^15^. This is because, typically, they estimate the cyclone intensity on the basis of the gridded 10-m wind speeds produced by the model, which are coarse across both space and time. To remedy this effect and provide a strong global machine-learning baseline to compare against in our evaluations, we apply a post-processing step to the predictions made by GenCast. Specifically, instead of using the 10-m wind fields directly, we rely on a well-known relationship between the minimum pressure at a cyclone’s centre and its maximum sustained wind speed^28^. This relationship takes the form:

$$\begin{array}{c}{v}_{{\rm{\mathrm{max}}}}=\alpha ({\rm{\mathrm{max}}}{(0,{p}_{0}-{p}_{{\rm{\mathrm{min}}}}))}^{\beta },\end{array}$$

where *v*_max_ is the 1-min maximum sustained wind speed, *p*_min_ is the minimum sea-level pressure at the cyclone centre and *α*, *β* and *p*_0_ are constants. We found that, instead of using the constant values proposed previously^28^, optimizing them directly on historical data from ERA5 and IBTrACS improved performance. Our optimized constants were were *α* = 6.70, *p*_0_ = 1.01 × 10^3^ and *β *= 6.44 × 10^−1^. At inference time, we first apply the TempestExtremes tracker^44^ to the gridded forecasts produced by GenCast, to produce cyclone tracks that include an estimate of the minimum sea-level pressure *p*_min_, and then we apply the pressure–wind-speed relation above to convert this to a prediction of the wind speed *v*_max_. This substantially improves the accuracy of the intensity forecasts of GenCast. We present both raw and debiased GenCast intensity results in Supplementary Fig. 4 to demonstrate the magnitude of this correction.

### Early forecast construction via interpolation

**Extended Data Fig. 3:**
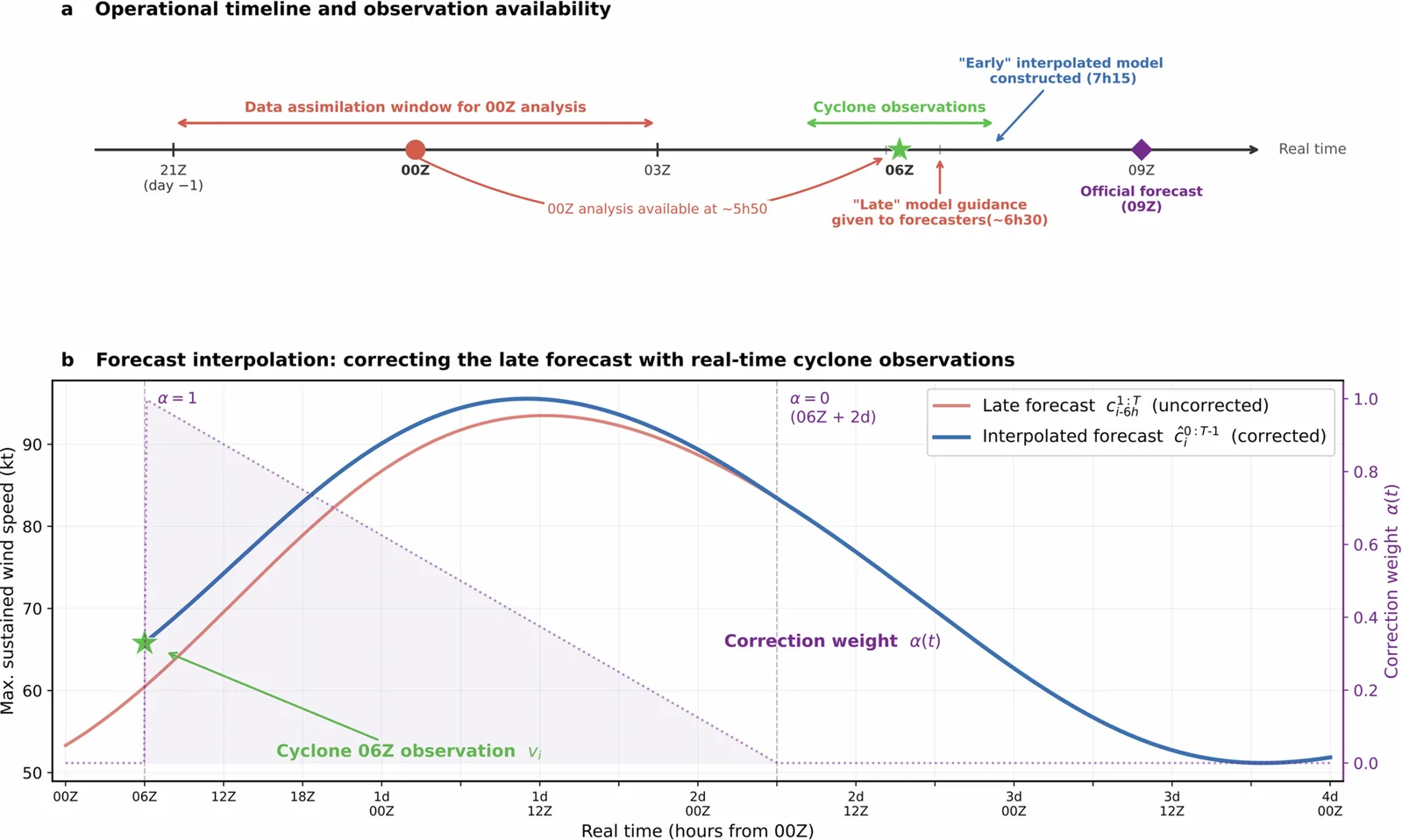
Schematic of operational timings and construction of the ‘early’ interpolated forecast. **a**, Global weather analyses have a latency of approximately 6 h due to the data-assimilation observation window and subsequent computation. Once the analysis becomes available, we produce a ‘late’ forecast and provide it as model guidance to forecasters. Using the most recent cyclone observations (available with negligible delay), we then construct an ‘early’ interpolated forecast by correcting the late forecast’s initial cyclone state. **b**, The correction weight *α*(*t*) decays linearly from 1 to 0 over the first 48 h of lead time (06Z to 06Z + 2d). Exact operational timings shown are those of the NHC; other agencies may differ slightly.

**Extended Data Fig. 4:**
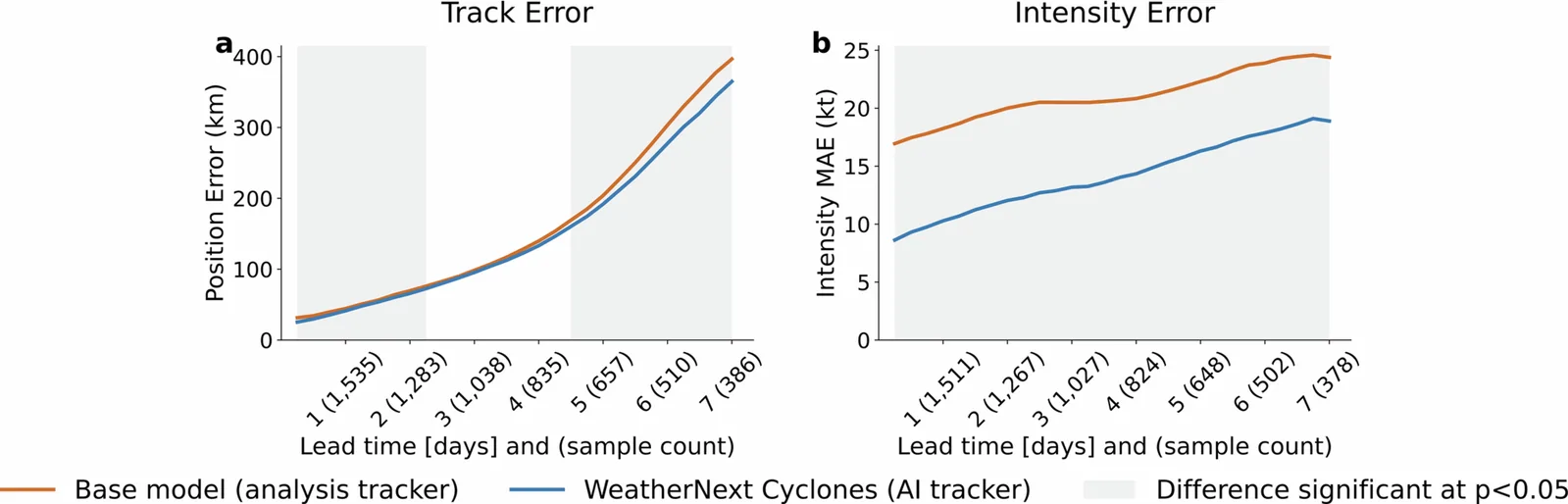
Co-training on IBTrACS and the direct tracker improve track and intensity errors. **a**,**b**, Mean track error (**a**) and intensity MAE (**b**) comparing the full WN-C to FGN (analysis only). Grey shading denotes lead times where the difference is statistically significant (*P* *<* 0.05). Together, co-training and the direct tracker yield large, statistically significant reductions in both track and intensity error. Evaluated on 2023 against IBTrACS^2,3^ without operational latency adjustment (i.e. without interpolation; Extended Data Fig. 3).

**Extended Data Fig. 5:**
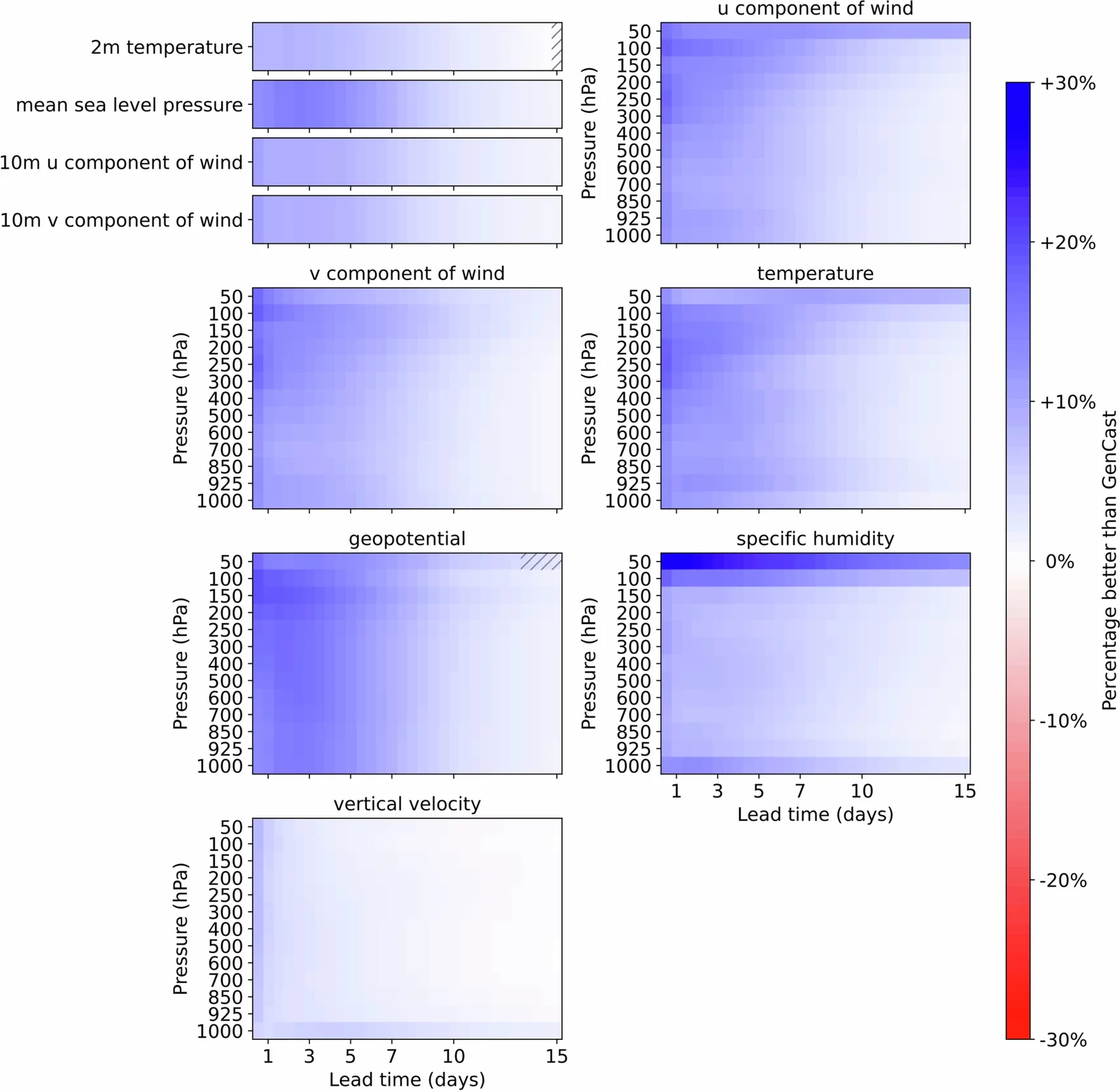
CRPS comparison of WN-C and GenCast. CRPS scorecard comparing WN-C to the currently operational version of GenCast on all initialization times in 2023, against HRES-fc0 ground truth, from ECMWF. WN-C significantly outperforms (*P* *<* 0.05) GenCast on 99.8% of targets, with an average improvement of 7.3% and a maximum improvement of 29.6%. Hatching indicates cells where the difference is not statistically significant (*P* *<* 0.05).

**Extended Data Fig. 6:**
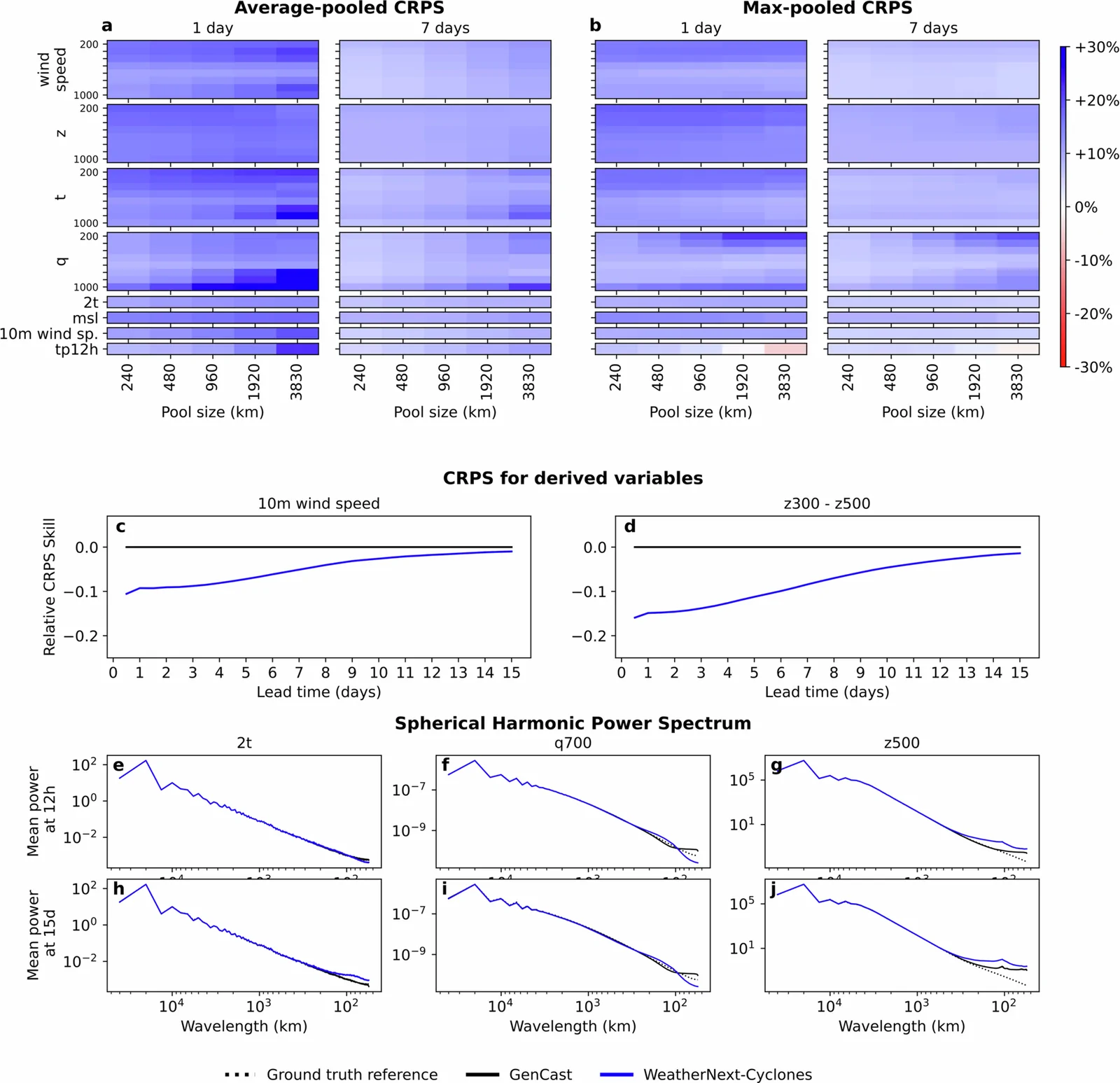
WN-C produces skilful joint forecast distributions. **a**,**b**, CRPS scorecards against GenCast evaluating average-pooled (**a**) and max-pooled (**b**) fields, for different pool sizes, at 1- and 7-day lead times. **c**,**d**, Evaluations of skill on two quantities of interest (10-m wind speed (**c**) and z300–z500 (**d**)) derived from predicted fields. **e**–**j**, Spectra of WN-C compared to GenCast, and the ground truth. **e**–**g**, Mean power at 12 h (2t (**e**), q700 (**f**) and z500 (**g**)).** h**–**j**, Mean power at 15 days (2t (**h**), q700 (**i**) and z500 (**j**)). Ground truth is HRES-fc0 from ECMWF^9^. 2t, 2-metre temperature; q700, specific humidity at 700 hPa; z500, geopotential at 500 hPa; z300–z500, 300–500 hPa geopotential thickness.

**Extended Data Fig. 7:**
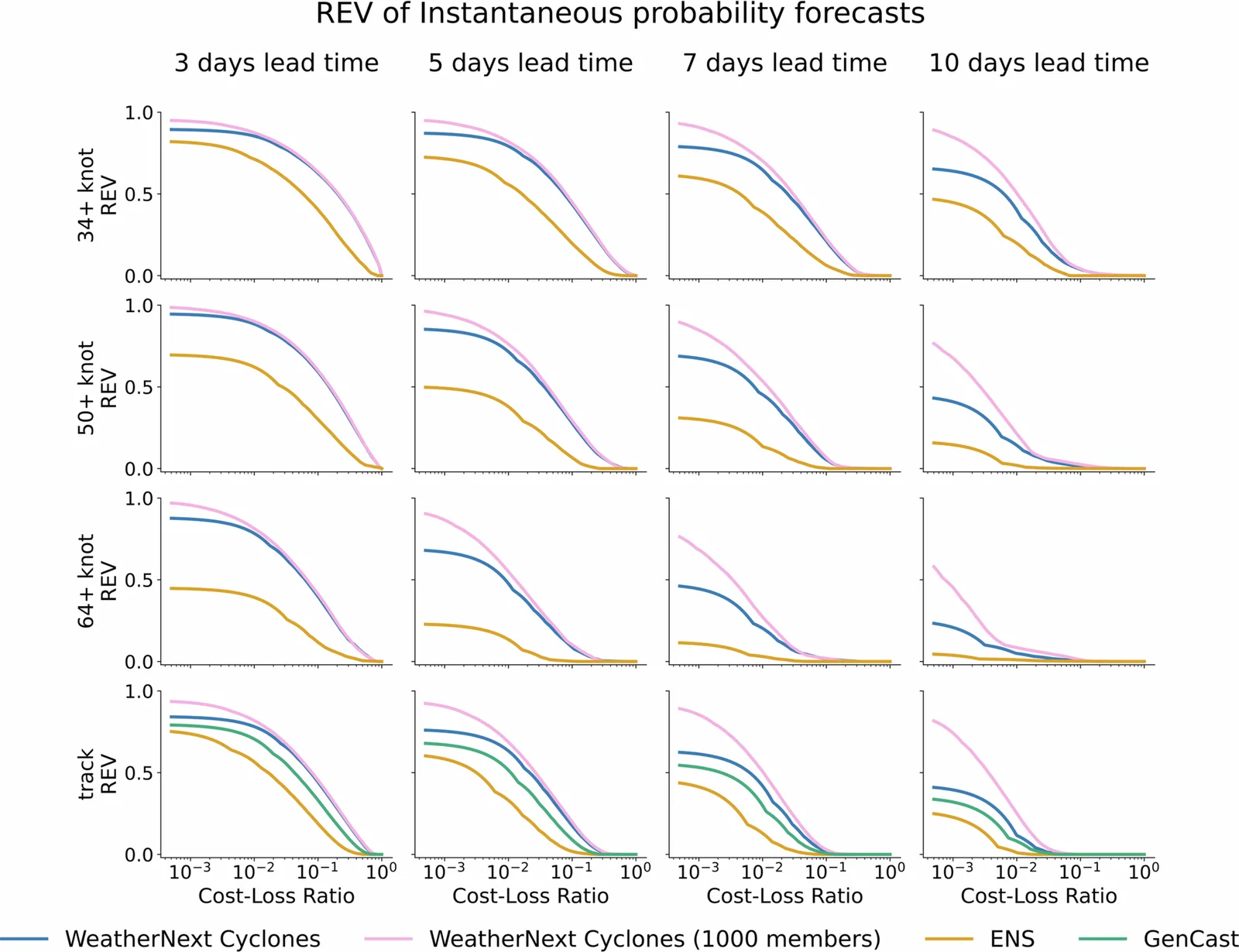
Relative economic value of WN-C instantaneous probability forecasts. REV across multiple lead times for instantaneous track probability and 34+ kt, 50+ kt and 64+ kt instantaneous wind-speed probabilities. The 7-day and 10-day lead times show results only for 00 UTC and 12 UTC initializations because ENS’s 06 UTC and 18 UTC initializations stop at a 6.25-day lead time. GenCast appears only in the bottom row, because it does not predict wind radii. Ground truth is IBTrACS^2,3^. ENS tracks were obtained from the ECMWF archive^9^.

**Extended Data Fig. 8:**
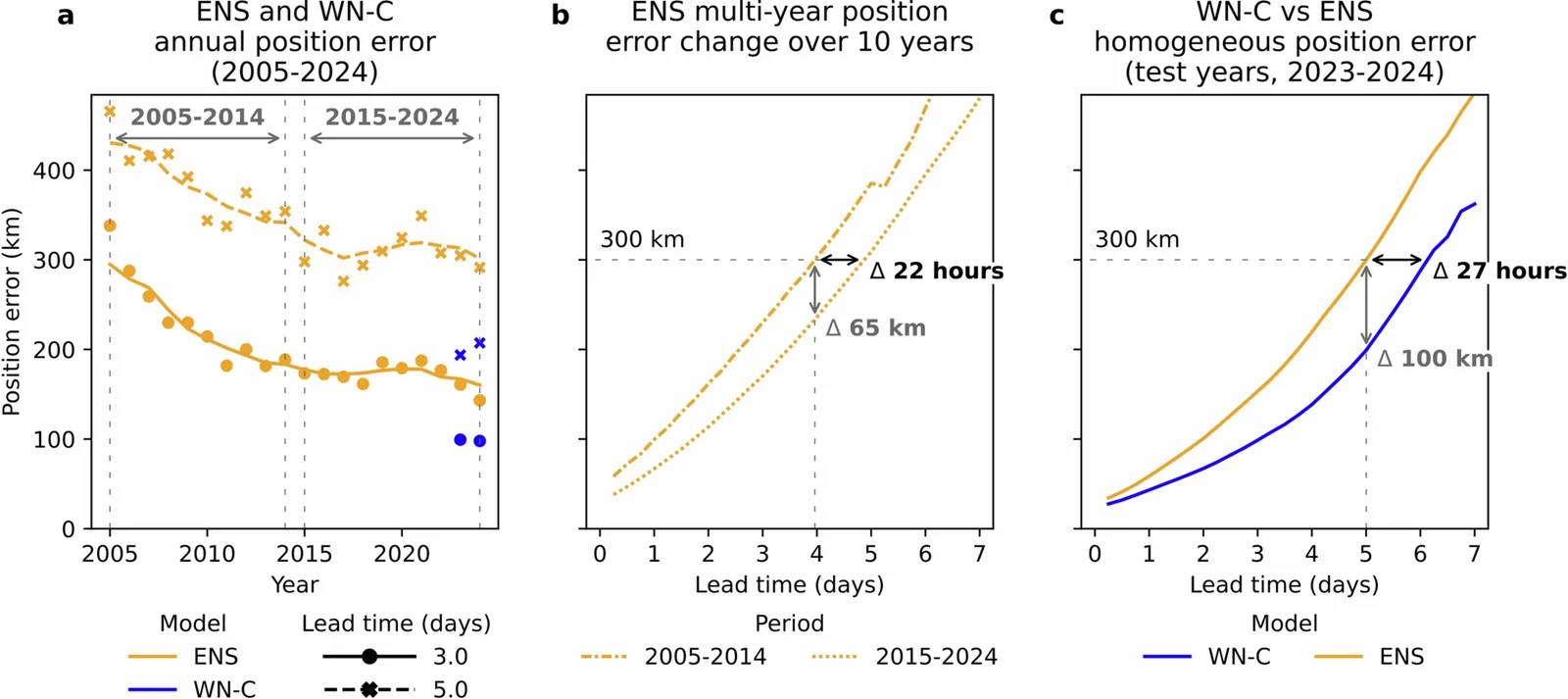
Benchmarking WN-C against the historical rate of progress of ECMWF ENS’s track skill. **a**, Annual mean tropical cyclone position error for ECMWF ENS and WN-C at forecast lead times of 3 days and 5 days. A five-year rolling average is overlaid to remove interannual variability. The ENS and WN-C results in 2023 and 2024 are homogeneous (Supplementary Information section 1.5.1). **b**, Growth of position error with lead time for ENS averaged over 2005–2014 versus 2015–2024. At a reference error threshold of 300 km, ENS gained approximately 22 h of predictive skill. **c**, Homogeneous comparison of position error growth for WN-C and ENS on the 2023–2024 test set. WN-C extends the useful forecast range by 27 h relative to ENS at the same 300 km threshold. Tropical cyclone ground-truth data from IBTrACS^2,3^. ENS tracks were obtained from the ECMWF archive^9^. See Supplementary Information section 2.7.1 for details.

**Extended Data Fig. 9:**
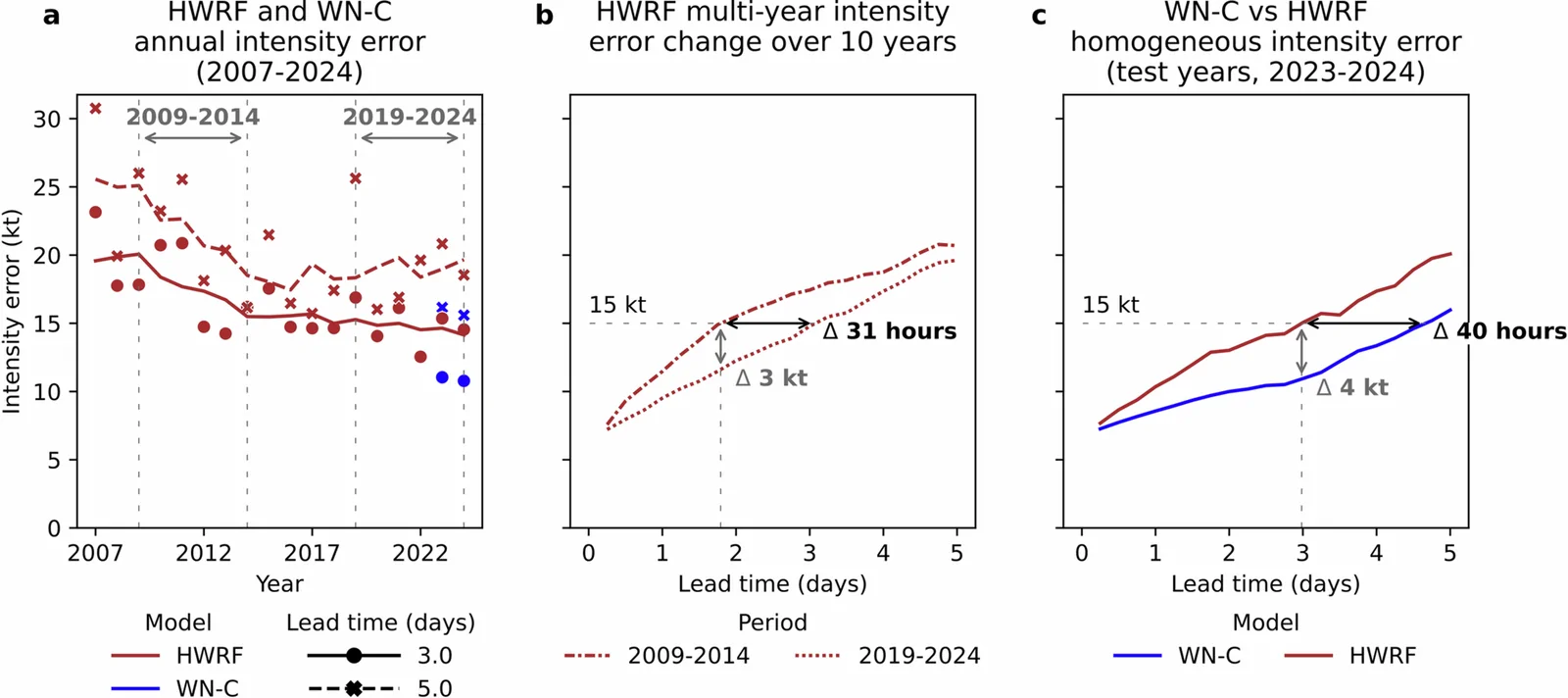
Benchmarking WN-C against the historical rate of progress of HWRF’s intensity skill. **a**, Annual mean tropical cyclone intensity error for HWRF and WN-C in the North Atlantic and Eastern North Pacific basins at forecast lead times of 3 days and 5 days. A five-year rolling average is overlaid to remove interannual variability. **b**, Growth of intensity error with lead time for HWRF averaged over 2009–2014 versus 2019–2024. At a reference error threshold of 15 kt, HWRF gained approximately 31 h of predictive skill. **c**, Homogeneous comparison of intensity error growth for WN-C and HWRF on the 2023–2024 test set. WN-C extends the forecast range by 40 h relative to HWRF at the same 15 kt threshold. Ground-truth data from IBTrACS^2,3^. HWRF tracks were obtained from NHC operational a-decks^43^. See Supplementary Information section 2.7.2 for details.

**Extended Data Fig. 10:**
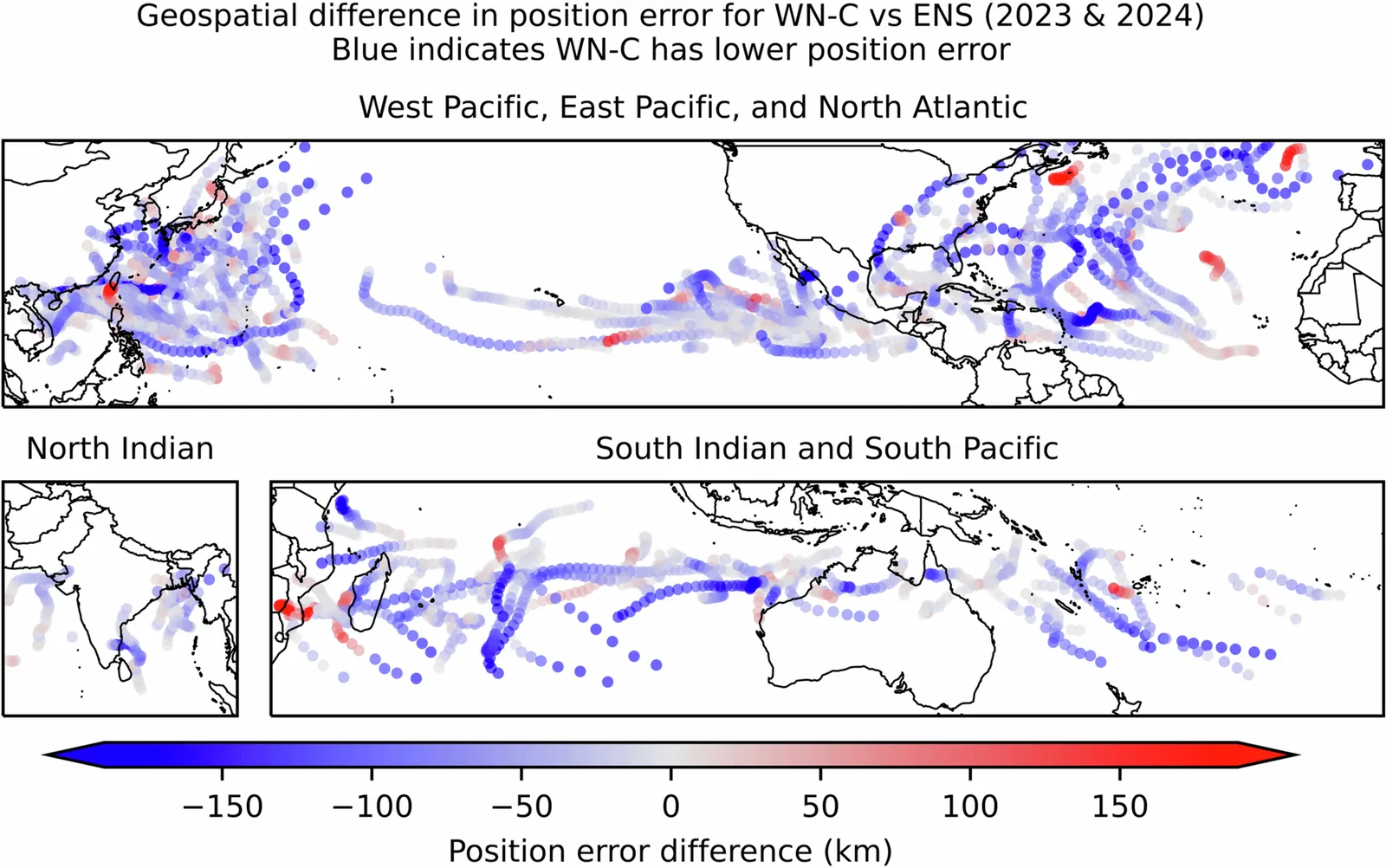
Geospatial illustration of WN-C versus ENS position error. Geospatial difference in ensemble mean position error for the interpolated homogeneous comparison, averaged over all lead times. The colour bar saturates at 95 % of the maximum absolute difference. Base map data provided by Natural Earth. Tropical cyclone ground-truth data from IBTrACS^2,3^, and baseline forecast data from NHC operational a-decks^10^.

**Extended Data Fig. 11:**
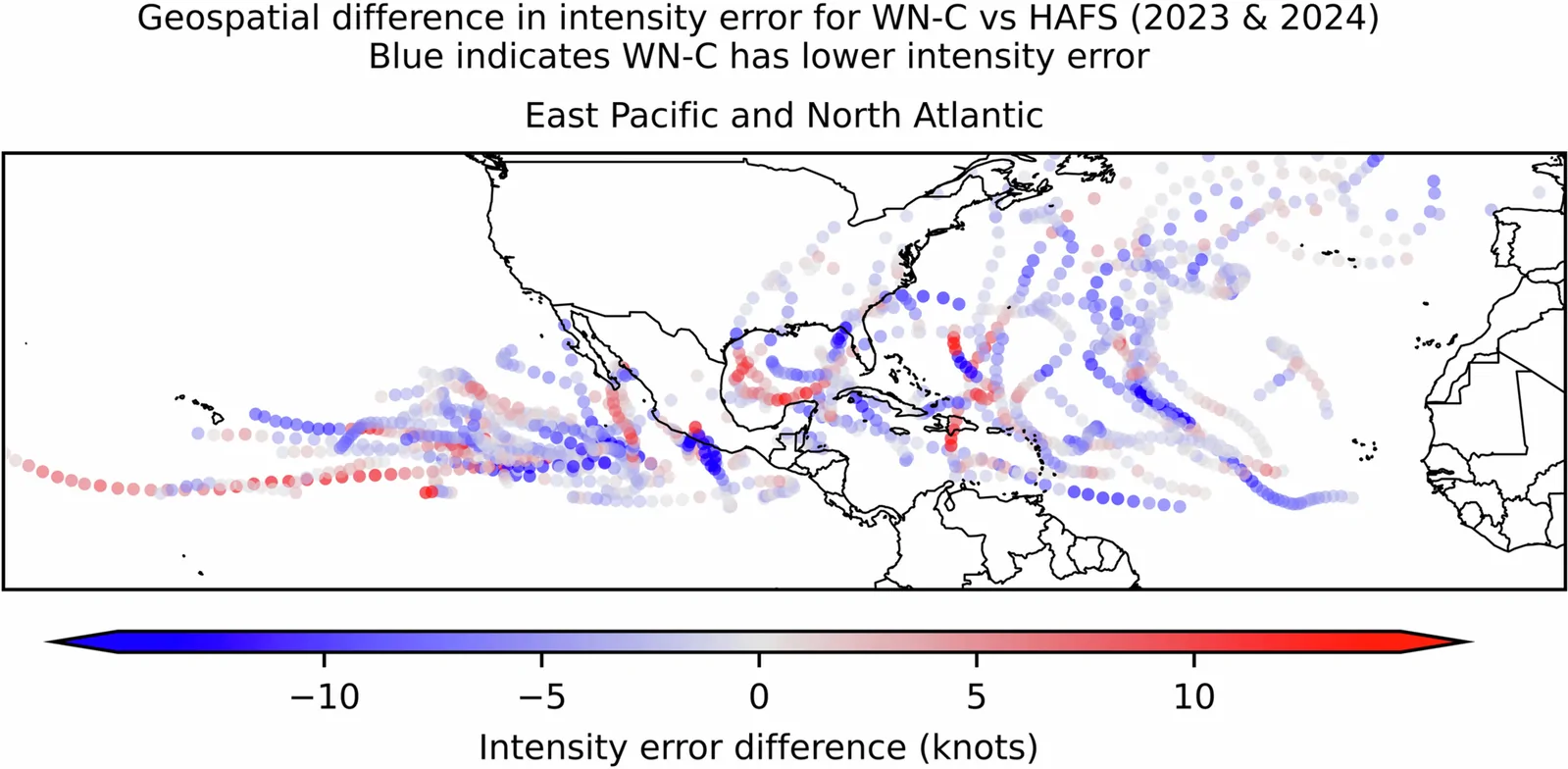
Geospatial illustration of WN-C versus HAFS intensity error. Geospatial difference in ensemble mean intensity error for the interpolated homogeneous comparison, averaged over all lead times. The colour bar saturates at 95% of the maximum absolute difference. Note that the HAFS data from the NHC a-decks are available only in the East Pacific and North Atlantic. Base map data provided by Natural Earth. Tropical cyclone ground-truth data from IBTrACS^2,3^, and baseline forecast data from NHC operational a-decks^10^.

To explain how interpolated (‘early’) forecasts are constructed, it is useful to distinguish between synoptic time and what we will call real time. Synoptic time is the time to which a given analysis atmospheric state corresponds, whereas real time is the time on one’s watch. Data assimilation, the process that produces an analysis state, uses observations from a window that includes a few hours before and after the synoptic time of that analysis state, and it then takes a few more hours to generate the analysis. In practice, this means that at a given point in real time *i*, one has access to the analysis only from synoptic time *i* − 6 h (the previous data-assimilation cycle). Consider a cyclone forecast ${c}_{i-6\mathrm{h}}^{0:T}$, which is initialized at synoptic timestep *i* − 6 h, and provides forecasts at 6-h intervals, such that ${c}_{i-6\mathrm{h}}^{1}$ is the forecast for real time *i*, ${c}_{i-6\mathrm{h}}^{2}$ is the forecast for real time *i *+ 6 h, and so on. To obtain an ‘early’ forecast made at real time *i*, we take the forecast ${c}_{i-6\mathrm{h}}$ for real time *i* onwards; that is, we define the early forecast ${\hat{c}}_{i}^{0:T-1}:= {c}_{i-6\mathrm{h}}^{1:T}$. We then apply a correction term based on TCVitals data for the current time *i*, *v*_*i*_. Specifically, we compute ${e}_{i}={c}_{i-6\mathrm{h}}^{1}-{v}_{i}={\hat{c}}_{i}^{0}-{v}_{i}$ and then compute the early (interpolated) forecast as ${\hat{c}}_{i}^{t}+\alpha (t){e}_{i}$, where *α*(*t*) is 1 at *t* = 0 and decays linearly to 0 when *t* corresponds to a 48-h lead time, remaining 0 thereafter. See Extended Data Fig. 3 for a schematic visualization of this correction process.

### Details of the consensus ensemble method

To estimate the value of WN-C cyclone forecasts in a setting that more closely resembles operational deployment, in the main-text section ‘Valuable operational guidance’ we incorporated its predictions within consensus ensembles for forecasting cyclone position and maximum sustained wind speed. A consensus ensemble is a model that combines multiple guidance predictions; for example, by averaging them. The resulting predictions are often substantially more accurate than is any one of the individual guidance predictions.

In our case, we combined WN-C within the track variable consensus ensemble (TVCN) and the intensity variable consensus ensemble (IVCN), to predict track and intensity, respectively. TVCN and IVCN operate by taking a simple average across the guidance models that are available for a particular forecast cycle and lead time. The set of available guidance models can vary, and there is a minimum number of at least two guidance models required for making a prediction. Note that when averaging over latitude and longitude specifically, care must be taken to correctly handle wrapping around the prime meridian at the 0–360° longitude boundary.

The simple average in TVCN and IVCN is equivalent to a weighted mean with equal weights; however, we found that because WN-C has substantially higher skill than does any one of the individual guidance models, it is beneficial to allow the weights to be unequal and to optimize them based on the validation set. This allows the consensus to optimally combine its guidance to optimize for performance on a particular performance metric.

Specifically, for each variable *k* ∈ {lat, lon, max wind}, given a forecast *c*_1,*kt*_ produced by WN-C and the base consensus prediction* c*_2,*kt*_, we combine them into a single consensus prediction *c*_*kt*_ by computing their weighted average, using respective probability weights *w*_1,*k*_ and *w*_2,*k*_:

$$\begin{array}{c}{c}_{{kt}}={w}_{1,k}{c}_{1,{kt}}+{w}_{2,k}{c}_{2,{kt}},\end{array}$$

subject to *w*_1,*k*_ + w_2,*k*_ = 1 and *w*_1,*k*_, w_2,*k*_ ≥ 0. Note that in our set-up we use different weights for each variable, because we do not expect WN-C to be equally skilful relative to consensus members across track and intensity by the same amount. In addition, we use lead times of up to 3 days when optimizing the weights (see details below), and then use fixed weight across lead times. We made these choices because in early explorations using forecasts from 2021 and 2022 we found that the weights might (a) slightly overfit due to the small size of the dataset and (b) be disproportionately affected by larger errors that naturally occur at the longer lead times.

We parameterize the logits of the weights *w*_*f*__,__*k*_ and *w*_*c*__,__k_ and use the MAE between ${c}_{{kt}}^{{\prime} }$ and the corresponding ground truth as the loss to train them. We minimize the MAE using full-batch gradient descent with the Adam optimizer and a learning rate of 10^−2^ for 10,000 descent steps. Once the weights have been optimized on the validation set, we use them to generate consensus predictions on the test set, and evaluate them in the same way that we evaluate all other deterministic paired forecasts (see ‘Evaluation methodology’ in the main text).

In addition to using IVCN and TVCN from the NHC a-decks as baselines, we also tried building versions of those ensembles by optimizing weights between its individual constituents, and obtained very similar results. We decided to use the official version from NHC to minimize any potential caveats, optimizing the weights of a two-member consensus containing WN-C and either TVCN (for latitude and longitude) or IVCN (for intensity).

WN-C receives 75% of the weight for latitude, 89% for longitude and 42% for intensity. Note that TVCN and IVCN themselves comprise several different guidance models, so the effective weight that WN-C receives relative to these individual models is even larger than that displayed here. This, together with the position and intensity errors in Fig. 5, suggests that WN-C can provide substantial value to a consensus ensemble.

## Online content

Any methods, additional references, Nature Portfolio reporting summaries, source data, extended data, supplementary information, acknowledgements, peer review information; details of author contributions and competing interests; and statements of data and code availability are available at https://doi.org/10.1038/s41586-026-10953-2.

## Supplementary information

Supplementary Information This file includes: Supplementary Text, Supplementary References and Supplementary Display Items. Supplementary Text, Section 1: Extended Methods (architecture and training details, computational requirements, verification metrics, data description, evaluation protocol). Section 2: Extended Results (2025 deterministic evaluation, extended global weather forecasting, extended baseline comparisons, Tracker/co-training ablations, wind-speed probabilities, significance tests, historical progress analysis, geospatial skill decomposition and WN-C Mini evaluation). Supplementary Display Items: 19 Supplementary Figures and 2 Supplementary Tables. These items collectively illustrate deterministic and probabilistic evaluation metrics, baseline performance comparisons, historical rate of progress, model ablations, geospatial error decompositions and evaluations of the lightweight WN-C Mini model.

Peer Review File

## Data Availability

ERA5 reanalysis data are publicly available from the Copernicus Climate Data Store. IBTrACS data are publicly available from the NOAA’s National Centers for Environmental Information. HRES, ENS and HAFS data are available from their respective meteorological agencies. Figures were generated using Matplotlib^74^ and Cartopy^75^, with Natural Earth base maps (https://www.naturalearthdata.com). Operational tropical cyclone forecast data as well as reforecasts from WN-C and GenCast are available at https://deepmind.google.com/science/weatherlab.

## Code availability

The code and neural network weights required for WN-C inference are available at https://www.github.com/google-deepmind/weathernext, together with a colab notebook showing how to run the model and the tracker. We include a 1° version of the model with fewer parameters, WN-C Mini, which is cheaper to run and also outperforms global and regional NWP models (Supplementary Information section 2.9).
