# Supplementary material for: Operational tropical cyclone forecasting with AI
Source: Nature. 2026 Aug 6;657(8132):680–8. doi: 10.1038/s41586-026-10953-2 (PMC13581599; doi:10.1038/s41586-026-10953-2)
Supplement: Supplementary file 1 [file 41586_2026_10953_MOESM1_ESM.pdf]

nature portfolio

https://doi.org/10.1038/s41586-026-10953-2

Supplementary information

Operational tropical cyclone forecasting with AI

In the format provided by the authors and unedited

Supplementary Information: Operational tropical cyclone forecasting with AI

Ferran Alet[1,*], Tom R. Andersson[1,*], Ilan Price[1,*], Stratis Markou[1,*], Andrew El-Kadi[1,*], Dominic Masters[1,*], Amy Li[2], Samier Merchant[3], Natalie Williams[3], Gregory Thornton[1], Ken MacKay[1], Olivia Graham[3], Akib Uddin[3], Ben Gaiarin[1], Devaja Shah[1], Elinor Kruse[1], Wallace Hogsett[4], David Zelinsky[4], John Cangialosi[4], Jonathan Martinez[4,5], James Franklin[5], Mark DeMaria[5], Kate Musgrave[5], Caroline L. Bain[6], Helen Titley[6], Jacklynn Stott[1], Remi Lam[1], Aaron Bell[3], Paul Komarek[1], Matthew Willson[1], Alvaro Sanchez-Gonzalez[1], Peter Battaglia[1]

[1]Google DeepMind [2]University of Waterloo, work done at Google DeepMind
[3]Google Research [4]NOAA/NWS/NCEP National Hurricane Center
[5]Cooperative Institute for Research in the Atmosphere [6]UK Met Office

[*]Equal contribution
Corresponding authors: {ferran,tomandersson,pricei,stratismarkou}@google.com

Contents

1 Extended Methods **2**
1.1 Architecture and training details . . . 2
1.2 Computational requirements . . . 3
1.3 Verification metrics . . . 3
1.4 Data . . . 5
1.5 Evaluation protocol . . . 8

2 Extended Results **9**
2.1 Deterministic metric evaluation for 2025 . . . 9
2.2 Extended global weather forecasting results . . . 10
2.3 Extended comparisons to other baselines . . . 14
2.4 Detailed tracker and co-training ablations. . . . 18
2.5 Extended wind speed probability results . . . 19
2.6 Statistical significance tests . . . 23
2.7 Historical rate of progress in cyclone prediction . . . 25
2.8 Geospatial decomposition of deterministic skill improvements . . . 26
2.9 WeatherNext Cyclones Mini . . . 28

1 Extended Methods

1.1 Architecture and training details

WeatherNext Cyclones (WN-C) uses a very similar architecture to that of GenCast, with some hyperparameter changes and some minor architectural differences.

Conditioning on input physical states. GenCast is a diffusion model, requiring multiple forward passes of the denoiser to generate a single forecast frame in a process of iterative refinement. As such, GenCast conditions on the two prior weather states, $X^{t-1,t-2}$, as well as a noisy version of the targets Z_σ, all concatenated along the channel dimension. WN-C generates a forecast in a single forward pass, and thus, similar to GraphCast[82] it simply conditions on the two prior weather states, $X^{t-1,t-2}$, without taking any noisy targets as inputs.

Shared encoder for conditional layer-norm layers. GenCast uses conditional layer-norm layers to condition on the noise level. First, the noise level is encoded into a vector of sine–cosine Fourier features, then it is passed through a small 2-layer multi-layer perceptron (MLP) to obtain 16-dimensional noise-level encodings before ultimately being used to condition the various layer-norm layers in the architecture. WN-C also uses conditional layer-norm layers but to condition on the noise vector of 32 elements. In this case, the noise vector is directly encoded with a single matrix multiplication to produce another vector of 32 elements, and is then passed as conditioning to the various layer-norm layers. Initially this encoder was added as an implementation detail, but we later found that it seemed to help with optimisation during training.

Capacity. GenCast contained MLPs with hidden and output sizes of 512, 4 attention heads, and 16 transformer layers. WN-C has a higher capacity with hidden and output sizes of 768, 6 attention heads, and 24 transformer layers. The only exception to this is the capacity of the MLPs that embed the edge features for the grid-to-mesh encoder graph and the mesh-to-grid decoder graph. For GenCast, this had hidden and output sizes of 512, while in WN-C these were reduced to 32. As there are $O(10^5)$ edges, but only 4 edge features to embed, we found this had no detrimental effect on performance while yielding substantial savings in compute and memory.

Global features. GenCast (and GraphCast) condition on the sine and cosine of the year's progress to tell the model which time of the year is being modeled. They broadcast this feature, which is constant in space, across the entire grid, also providing it as input to the encoder in addition to other grid features. In WN-C, we additionally broadcast this feature across the entire mesh, concatenating it with the rest of the mesh input features.

Encoder from the grid to mesh. GenCast (and GraphCast) use a graph neural network (GNN) to translate features between the grid and mesh structures by building a bipartite graph with edges pointing from the grid nodes to the mesh nodes. In these, the "edge model" (also often referred as "message function") is a function that conditions on both the features of the sender grid nodes, and the receiver mesh nodes, via gather operations, for every edge of the graph. For WN-C, we found that we could remove this conditioning on the receiver mesh nodes in the message function at no performance cost and substantial compute and memory savings.

Training protocol. WN-C was trained in multiple stages, outlined in Supplementary Table 1.

Open-source model with reduced size. We also train a smaller version of WN-C (WN-C Mini) that has a smaller computational footprint, for quicker experimentation and greater accessibility. We emphasise that the main purpose of this model is to provide a lightweight demonstration, and does not reflect the skill of the larger, higher resolution, model. The architectural differences, training protocol, and evaluation results for this model are detailed in Supplementary Section 2.9.

Supplementary Table 1: Model training phases and hyperparameters. Stages 1 and 2 train only on ERA5 data, whereas stages 3–6 include direct cyclone targets (DCT; see Section 2.2).

	**Stage 1**	**Stage 2**	**Stage 3**	**Stage 4**	**Stage 5**	**Stage 6**
**Training dataset**	ERA5			ERA5, DCT		HRES-fc0, DCT
**Time-step size**	12 hr	6 hr				
**Spatial resolution**	1°		0.25°			
**Optimised params**	All except cyclone output head			Cyclone output head	All	
**AR steps**	1					1–8
**Training samples**	2					
**Batch size**	64					
**Optimiser**	AdamW[83]					
**Weight decay**	0.1					
**LR schedule**	Cosine with linear warmup					
**Peak LR**	8e-4	8e-5				8e-5, 8e-6, [8e-7]× 6
**Warmup steps**	1000					800, 400, [100]× 6
**Total steps**	400k	100k	32k	5k	20k	8k, 4k, [1k]× 6

1.2 Computational requirements

Generating a single 15-day WN-C forecast with our 768 embedding size model at 0.25° takes about 1 minute on a Tensor Processing Unit (TPU) v5p device, and an ensemble of forecasts can be generated in parallel. During real-time inference, we generate a regular size ensemble (50 members) and a large size ensemble (1000 members). For the regular size, we first generate 16 members from each of the four model checkpoints, using 64 TPUv5 devices working in parallel. To make a 50-member ensemble, we select 50 members in a balanced manner, taking 12 members from each of the four model checkpoints and one additional member from the first two checkpoints, for a total of 50. For the large ensemble, we generate 240 additional members from each of the four checkpoints, using 256 TPUv5 devices working in parallel. We collect the resulting 240 * 4 = 960 members together with the original 64, and sub-sample the result down to 1000 in the same balanced procedure. For the 1° WN-C Mini provided in our code release, generating a single 15-day forecast takes about 20-25 seconds on a single TPUv4 device.

1.3 Verification metrics

1.3.1 Mean absolute error (MAE) of ensemble mean

To assess the deterministic skill of the ensemble, we compute the MAE of the ensemble mean against the ground truth. For scalar variables such as maximum wind speed and wind radii, this is simply the arithmetic mean of the absolute differences. For track forecasting, the MAE is defined as the mean geodesic (great-circle) distance between the ensemble mean position (the centroid of the member positions) and the ground truth best track location.

The ensemble mean is computed only with the ensemble members predicting the existence of the cyclone and only defined if at least 30 % of the ensemble members are still making predictions.

1.3.2 CRPS

The CRPS is a strictly proper scoring rule that measures the accuracy of the probabilistic forecast for univariate scalar variables. This property guarantees that the expected score is minimised uniquely when the predicted distribution aligns with the true distribution. Consequently, the metric

incentivises 'honest' forecasting, ensuring that the model is rewarded for accurately capturing both the position and the associated uncertainty.

As detailed in Methods Section 11.3, we utilise the standard decomposition for ensemble forecasts. We apply this metric to intensity (maximum wind speed), minimum sea level pressure, and wind radii to quantify the quality of the marginal distributions produced by the model.

1.3.3 Position energy score

The energy score[84] is a strictly proper scoring rule which generalises CRPS to vectors in $\mathbb{R}^n$, allowing us to use euclidean distance in place of mean absolute error.

$$ES(F, \mathbf{y}) = \mathbb{E}_{\mathbf{x}\sim F} \, ||\mathbf{x} - \mathbf{y}||_2 - \frac{1}{2}\mathbb{E}_{\mathbf{x},\mathbf{x}'\sim F} \, ||\mathbf{x} - \mathbf{x}'||_2 \tag{1}$$

Like for CRPS, we use the fair estimator of the energy score, which accounts for the fact that the effective ensemble size for different predictions differs based on how many ensemble members predict the existence of a cyclone. Note that there is some subtlety with respect to the distribution to which this metric compares the predicted distributions. Specifically, since this metric is only defined when a predetermined non-zero fraction of ensemble members are active, we are not taking an expectation over the ground truth position distribution, but rather over that ground truth distribution conditional on the model predicting the cyclone's existence with a minimum probability, i.e. the 30 % threshold used in defining the ensemble mean (Supplementary Section 1.3.1).

1.3.4 Spread/skill ratio

The spread/skill ratio is a measure of ensemble calibration. We define the "skill" as the root mean square error (RMSE) of the ensemble mean, and the "spread" as the square root of the average variance of the ensemble members around the ensemble mean. A ratio of 1 indicates that the ensemble variance correctly reflects the magnitude of the forecast error on average. Ratios less than 1 indicate on average the ensemble is under-dispersive (overconfident), while ratios greater than 1 indicate over-dispersion (underconfident).

Spread/skill ratio for position forecast. In order to facilitate an unbiased estimate of the spread/skill ratio for predicted cyclone position, we compute the bias-corrected squared position error in km, approximated by squared euclidean distance after projecting onto the tangent plane of the target. The bias-correction is then the variance of the ensemble of predicted positions in the target plane divided by the number of ensemble members in that forecast. "Skill" is computed as the square root of the average of this quantity over the test set. "Spread" is computed as square root of the mean over the test set of the unbiased ensemble variance of predicted position after projection into the tangent plane of the target.

1.3.5 Relative Economic Value

Relative Economic Value (REV)[85], measures the utility of a forecast for a decision-maker who takes protective action based on whether the probability of an adverse event E exceeds a cost-loss ratio threshold $\alpha = C/L$ (see also Supplementary Information Section A.5.6 of Price et al.[86] for an exposition). The value is defined as the reduction in expected expense relative to a baseline of using only climatological frequency, normalised by the value of a perfect forecast. This baseline either always takes protective action (for low C/L) or never does (for high C/L). Following ref.[85], instead of using a single pre-determined threshold $\alpha = C/L$ we compute REV at every possible threshold for the empirical predictive probability of our ensemble, and report the maximum REV over these.

1.3.6 Reliability diagrams

Reliability diagrams provide a visual assessment of the calibration of predicted categorical event probabilities. We bin the predicted probabilities of an event, such as rapid intensification (RI) or $>$ 34kt wind speeds, into width-10 % bins, and plot the observed frequency of that event against the mean predicted probability for each bin. For a perfectly calibrated model, the points lie on the $y = x$ diagonal, such that out of all instances where the event was predicted with 30 % probability, the event occurred 30 % of those times, and so on. Note that the predicted probabilities are not necessarily uniformly distributed within each bin (e.g. it's more common for 24 % of members to predict RI than 28 % because RI is rare); that implies that the empirical x position of each bin is not at the centre bin (the centre of the 20-30 % bin is not 25 %).

1.3.7 Lack of evaluations of RMW

Our model also forecasts the radius of maximum winds (RMW). Internal evaluations show significant improvements for RMW, but this variable has significantly fewer observations in IBTrACS, which limits the statistical significance of conclusions that can be drawn from its verification. We have therefore omitted RMW verification results from our evaluations.

1.4 Data

The following descriptions of ERA5 and HRES-fc0 datasets are largely adapted from Lam et al.[87] and Price et al.[88]. We include some additional details around preprocessing of HRES-fc0 for the purposes of training WN-C.

1.4.1 ERA5

We made use of a subset of ECMWF's Reanalysis v5 (ERA5)[89] archive, which is a large corpus of data that represents the global weather from 1959 to the present, at 1 hour increments, for hundreds of static, surface, and atmospheric variables. The ERA5 archive is based on *reanalysis*, which uses ECMWF's HRES model (cycle 42r1) that was operational for most of 2016, within ECMWF's 4D-Var data assimilation system. ERA5 assimilates 12-hour windows of observations, from 21z-09z and 09z-21z, as well as previous forecasts, into a dense representation of the weather's state, for each historical date and time. ERA5 consists of a deterministic reanalysis computed at computed at a native horizontal resolution of T639, corresponding to a nominal grid spacing of 31 km (or 0.28125°).

Our ERA5 dataset contains a subset of available variables in ECMWF's ERA5 archive (Supplementary Table 2), on 13 pressure levels corresponding to the WeatherBench[90] benchmark. Following common practice, we use pressure as the vertical coordinate: a pressure level (e.g., 500 hPa) represents the isobaric surface at which atmospheric pressure equals that value, with the geopotential variable encoding the relationship between pressure and altitude. The levels used are: 50, 100, 150, 200, 250, 300, 400, 500, 600, 700, 850, 925, 1000 hPa. The range of dates included was 1979-01-01 to 2018-01-15, which were downsampled to 6-hour time intervals (corresponding to 00z, 06z, 12z and 18z each day). The downsampling is performed by subsampling, except for the total precipitation, which is accumulated for the 6 h leading up to the corresponding downsampled time.

Variables with NaNs. ERA5 sea surface temperature (SST) data contains NaNs over land by default. As preprocessing of the SST training and evaluation data, values over land are replaced with the minimum sea surface temperature seen globally in a subset of ERA5.

Type	Variable name	Short name	ECMWF ID	Role
Atmospheric	Geopotential	z	129	Input/Predicted
Atmospheric	Specific humidity	q	133	Input/Predicted
Atmospheric	Temperature	t	130	Input/Predicted
Atmospheric	U component of wind	u	131	Input/Predicted
Atmospheric	V component of wind	v	132	Input/Predicted
Atmospheric	Vertical velocity	w	135	Input/Predicted
Single	2 metre temperature	2t	167	Input/Predicted
Single	10 metre u wind component	10u	165	Input/Predicted
Single	10 metre v wind component	10v	166	Input/Predicted
Single	Mean sea level pressure	msl	151	Input/Predicted
Single	Sea Surface Temperature	sst	34	Input/Predicted
Single	6 hour total precipitation	tp	228	Predicted
Cyclone	Track latitude	LAT	n/a	Predicted
Cyclone	Track longitude	LON	n/a	Predicted
Cyclone	Max sustained wind-speed	USA_WIND	n/a	Predicted
Cyclone	All-provider wind-speed	ALL_WIND†	n/a	Predicted
Cyclone	Minimum sea-level pressure	USA_PRES	n/a	Predicted
Cyclone	Radius of maximum winds	USA_RMW	n/a	Predicted
Cyclone	{NE,NW,SE,SW} 34 kt radii	USA_R34_*	n/a	Predicted
Cyclone	{NE,NW,SE,SW} 50 kt radii	USA_R50_*	n/a	Predicted
Cyclone	{NE,NW,SE,SW} 64 kt radii	USA_R64_*	n/a	Predicted
Static	Geopotential at surface	z	129	Input
Static	Land-sea mask	lsm	172	Input
Static	Grid cell latitude	n/a	n/a	Input
Static	Grid cell longitude	n/a	n/a	Input
Clock	Local time of day	n/a	n/a	Input
Clock	Elapsed year progress	n/a	n/a	Input

Supplementary Table 2: ECMWF analysis and cyclone best-track variables used in our datasets. The "Type" column indicates whether the variable represents a *static* property, a time-varying *single*-level property (e.g., surface variables), a time-varying *atmospheric* (3D) property, a time-varying tabular scalar per *cyclone*, or per *cyclone quadrant* (one for each of North-East, South-East, South-West, and North-West). The "Variable name" and "Short name" columns are ECMWF's labels or cyclone best track's labels. †ALL_WIND is a wind variable that we derive from IBTrACS by combining wind speeds from different providers (see text for description). The "ECMWF Parameter ID" column is a ECMWF's numeric label, and can be used to construct the URL for ECMWF's description of the variable, by appending it as suffix to the following prefix, replacing "ID" with the numeric code: `https://apps.ecmwf.int/codes/grib/param-db/?id=ID`. The "Role" column indicates whether the variable is something our model takes as input and predicts, or only predicts, or only uses as input context. The middle horizontal rules separate atmospheric variables, surface variables, cyclone best-track variables, and input-only variables. The GenCast baseline (but not this version of WN-C) was also trained with 100m u- and v- components of wind, but we found its inclusion has very little impact on other variables in our evaluations (not shown).

1.4.2 HRES-fc0

HRES-fc0 contains the initial (zeroeth) step of the ECMWF HRES forecast at each initialisation time (00z, 06z, 12z, 18z) of the day. The data is similar to ERA5, but for a given forecast time, it is assimilated using the latest ECMWF NWP model, and only with observations from $\pm 3\,\text{h}$ relative to the synoptic time that were available in real-time.

Variables with corresponding NaNs in ERA5. Unlike ERA5, HRES-fc0 does not make use of NaNs to represent land within the sea surface temperature (SST) data. Rather, we found a placeholder value was used. To ensure a consistent representation between the two datasets in training, for all locations with a NaN in the original ERA5 dataset, we imputed the corresponding location in HRES-fc0 with the minimum sea surface temperature as well.

Total precipitation. As an accumulated variable, total precipitation (tp) takes a value of 0 in HRES-fc0, inhibiting its use in a setting where the model is initialised on the dataset. To account for this, WN-C is trained to only output tp, not taking it as input. Further, while ERA5 accumulates tp over the interval leading up to the initialisation time, using HRES tp would require accumulating the variable forwards in time (since the interval prior is another forecast initialisation's trajectory). To mitigate this discrepancy, we continue to use ERA5's tp for both finetuning and evaluation purposes.

1.4.3 Source of cyclone best-track data

We use the International Best Track Archive for Climate Stewardship (IBTrACS[91,92]) version 4r01 as the primary source of tropical cyclone data for training and evaluating WN-C. IBTrACS aggregates best-track data from numerous agencies and Regional Specialised Meteorological Centers (RSMCs) worldwide. IBTrACS includes data interpolated to 3-hourly frequency, but we subsample the data to 6-hourly synoptic times (00, 06, 12, 18 UTC) for which the IBTrACS-contributing operational centres report best-track data, which aligns with our model's timestep and the analysis data. The dataset provides the ground truth data for the cyclone-specific variables outlined in Supplementary Table 2. We use the USA_* columns for all variables (position and scalar properties), which are provided by the U.S. NHC and Joint Typhoon Warning Center and use a 1-minute averaging period for wind speed variables. This choice ensures a globally consistent definition of tropical cyclone intensity, avoiding the conflicting definitions (such as 10-minute wind averaging) used by other agencies.

In addition to the IBTrACS variables in the USA_WIND field, we also make use of wind speeds from other providers, by deriving an ALL_WIND variable (see Supplementary Table 2) that aggregates across data from different providers. For each provider in the set of all providers $A = \{\text{CMA}, \text{NEWDELHI}, \text{REUNION}, \text{BOM}\}\_\text{WIND}$, we fit a univariate linear model f_a that interpolates USA_WIND as a function of the wind speed from each provider $a \in A$. We fit each of these models on data from the period 01/01/1979, to 31/12/2015, for instances where USA_WIND and $a \in A$ are observed for the same cyclone at the same time. We then create the ALL_WIND variable for each cyclone data point i, by computing the value of f_a for each of the providers that are available at that example A_i, clipping the result of each linear model below by zero, and averaging across the providers, i.e. $\frac{1}{|A_i|}\sum_{a\in A_i} \texttt{max}(f_a(w_a), 0)$. We include this derived variable in our training set (Supplementary Table 2) but do not evaluate it.

1.5 Evaluation protocol

1.5.1 Paired evaluations: Storm stage subsetting and homogeneous model comparisons

For our paired evaluations, we adhere to the verification procedure used by the NHC in their annual verification reports[93]:

1. **System Status:** For paired evaluations, we evaluate models only when the observed storm system is classified in IBTrACS as having a tropical or subtropical nature (NATURE equal to TS or SS), excluding disturbances (DS), extratropical cyclones (ET), and unclassified systems (NR/MX). This subsetting is omitted only for the geospatial error decomposition plots shown in Extended Data Figure 10 and Supplementary Figures 16 and 17 and Extended Data Figure 11, which include disturbances and extratropical cyclones to show more datapoints.

2. **Homogenisation:** A forecast lead time is included in the aggregate metrics only if all models in the comparison (e.g., WN-C, ENS, and HAFS) predict an active tropical cyclone for that specific forecast cycle *and* the true system existed in IBTrACS at the valid time.

1.5.2 Justification for comparing ensemble and deterministic models in deterministic cyclone evaluations

In our deterministic evaluations, we compare ensemble means of WN-C, ENS and GenCast against deterministic models like HAFS and HRES (Supplementary Figure 5). Although the best minimiser for the MAE metrics is the ensemble median, ensemble means generally also benefit from noise cancellation and therefore have a systematic advantage over single deterministic forecasts in terms of mean error. However, this comparison reflects standard operational practice: forecasters routinely use ensemble mean guidance from models such as ENS, and the NHC's own official forecast verification[93] evaluates ensemble means alongside deterministic models. Moreover, our probabilistic evaluations (Figure 3) directly compare full ensemble distributions via proper scoring rules (CRPS, energy score).

1.5.3 ENS operational latency advantage

In practice, the ECMWF ENS forecast is not available within the 6h30m window after synoptic time that is required for real-time operational use; forecasters therefore typically access ENS with a 12 h delay rather than 6 h. In our evaluations, we deliberately give ENS the benefit of the shorter 6 h delay—the same latency we assume for WN-C—so that the comparison is as favourable to ENS as possible. This means that the improvements over ENS reported in this manuscript are conservative relative to what would be observed under true operational timing constraints.

1.5.4 Unpaired evaluations: Handling of missing values in IBTrACS quadrant wind radii

For unpaired evaluations, i.e. track and wind speed probability metrics, we include all forecasts of a given model without applying homogenisation and include all storm stages of development, unlike paired evaluations (Supplementary Section 1.5.1).

The IBTrACS quadrant radii data occasionally contain missing values (NaNs) that cannot be inferred to be 0 km based on the maximum sustained wind speed. These require careful handling when generating ground-truth strike masks for evaluation (e.g., for REV; Supplementary Section 1.3.5).

We use a NaN-aware aggregation scheme so that regions of missing data are correctly distinguished from regions with confirmed zero wind extent. For each wind speed threshold s, a climatological maximum radius $\bar{r}_s$ is defined (1500 km for 34 kt, 750 km for 50 kt, and 600 km for 64 kt—slightly above the historical maxima since 1980). When one or more quadrant radii are NaN at a given timestep, the non-NaN quadrants contribute their binary masks as usual, while each NaN quadrant instead contributes a NaN mask over a disc of radius $\bar{r}_s$ in the corresponding quadrant. These are combined using a NaN-aware logical OR operator $\oplus$ defined as:

$$a \oplus b = \begin{cases} 1 & \text{if } a = 1 \text{ or } b = 1, \\ 0 & \text{if } a = 0 \text{ and } b = 0, \\ \text{NaN} & \text{otherwise.} \end{cases}$$

This ensures that: (1) a grid cell with a confirmed positive strike from any non-NaN quadrant is correctly marked; (2) a cell that lies only in NaN quadrants remains NaN, propagating the uncertainty; and (3) a cell outside all discs remains zero.

2 Extended Results

2.1 Deterministic metric evaluation for 2025

On 12th June 2025, we began releasing live, operational WN-C cyclone trajectory forecasts publicly on Weather Lab (https://deepmind.google.com/science/weatherlab), which were used in real-time by forecasters at agencies like the NHC and the UK Met Office. Here we provide an evaluation of WN-C on paired, deterministic metrics for the 2025 season on the North Atlantic and East Pacific basins (Extended Data Figure 1). On September 25th 2025, we released an upgrade of the cyclone tracker (which maps from the neural network's gridded cyclone channels to tabular cyclone trajectories; see Extended Data Figure 2). To assess the impact of this upgrade, we evaluate three variants of WN-C, each using different configurations of the cyclone tracker (but the same underlying neural network, f_θ):

1. WeatherNext Cyclones (v0 tracker): This initial version estimated scalar variables by performing a weighted average based on the existence and scalar variable fields, and did not involve the pruning operation in the advancing step of the tracker.

2. WeatherNext Cyclones: The v1 tracker version presented in this work, which ran operationally since September 25th 2025. It introduced pruning to address tracker jumps, and replaced the weighted average approach with bilinear interpolation to estimate the scalar parameters. This is the version described in Methods Section 10.3.

3. WeatherNext Cyclones (realtime): The model that ran in real time for forecasters, v0 until September 25th 2025 and v1 for the rest of 2025.

The upgrade from the v0 to the v1 tracker brought noticeable improvements in intensity, and very large gains on wind radii. This is because the model predicts wind radii conditioned on the precise centre position, and the v0 tracker's weighted-average extraction inadvertently smoothed these predictions.

Note, unlike our historical evaluations which initialise the tracker using IBTrACS (step 1 in Methods Section 10.3), the real-time tracker is initialised using observed cyclone data from TCVitals, since IBTrACS is not available in real time.

2.2 Extended global weather forecasting results

We compare WN-C to GenCast[86] and ECMWF's ENS[94]. While GenCast was trained and evaluated on ERA5 reanalysis data in ref. 86, it has since been fine-tuned on operationally available HRES-fc0 data, as part of Google's WeatherNext family of operational models where it is called WeatherNext Gen. Here we focus our evaluations on the operational setting, and thus compare performance to the WeatherNext Gen forecasts. Furthermore, the resolution of ENS changed from 0.2° to 0.1° on the 27th of June 2023, as part of the upgrade from Cycle 47r3 to Cycle 48r1[95]. To account for this, all data was regridded to 0.25° using MetView[96] defaults (conservative downsampling). The models are evaluated against the HRES-fc0 ground truth in 2023 (while shorter than the 3 year period used for cyclone evaluations, this suffices as a sample size for analysis variable evaluations).

2.2.1 Marginal forecast distribution evaluations

Extended Data Figure 5 shows WN-C significantly outperforms GenCast on 99.8 % of targets, with an average improvement of 7.3 % and a maximum improvement of 29.6 %. Statistical significance for each scorecard cell is computed using the approach described in Price et al.[86]. Also as in Price et al.[86], we compare WN-C to ENS on 00z and 12z initialisations, a subset of 8 pressure levels, and without vertical velocity, as this is the data made available in the public TIGGE archive[97]. Supplementary Figure 1 shows very large improvements, averaging more than 10 % across all lead times, and up to 27.6 %. On the same subset of data, WN-C outperforms GenCast by 6.5 % on average, and up to 18.8 %.

We compute the lead time hours of advantage for each set of models. On a per-variable-level basis, for a given lead time T, and baseline model admitting CRPS error E, we find the lead time T' for which WN-C admits the same error (by interpolation). We truncate this to a maximum lead time of 10 days, which we confirmed avoids any model error extrapolations. Supplementary Figure 2 and Supplementary Figure 3 demonstrate the lead time improvements WN-C provides over GenCast and ENS, with average advantages of 18.8 h and 26.2 h respectively.

Effect of cyclones co-training on global weather variables. In Alet et al.[98], we described the modelling approach behind WN-C, which we named the *functional generative network* (FGN; Methods Section 11), where the model was trained with analysis variables only (i.e. without IBTrACS output channels)[98]. The content of this preprint is now contained in the present manuscript. The primary differences relative to WN-C is the removal of training Stage 4 (cyclone output head warmup) and the exclusion of direct cyclone targets (DCT) from training Stages 5 and 6 (see Table 1). We verified that this difference does not affect weather forecasting performance: WN-C's skill on standard weather variables is extremely similar to the analysis-only FGN model—marginally positive, but within typical training variance (not shown).

2.2.2 Joint forecast distribution evaluations

In addition to the above results quantifying the skill-improvement of the marginals of the forecast distribution, (the per-location, per-lead-time, per-level forecast distributions), we assess the quality of the correlation structure of the joint forecast distributions via a number of standard proxies, each of which quantifies specific aspects of this joint distribution.

Extended Data Figure 6 shows our joint distribution evaluation comparing WN-C and GenCast. First, we assess the spatial correlations and dependencies in the forecast distribution by performing probabilistic evaluation on spatially pooled predicted fields. We follow the pooling protocol described in 'Spatially pooled CRPS evaluation' in the Methods section in Price et al.[86], evaluating

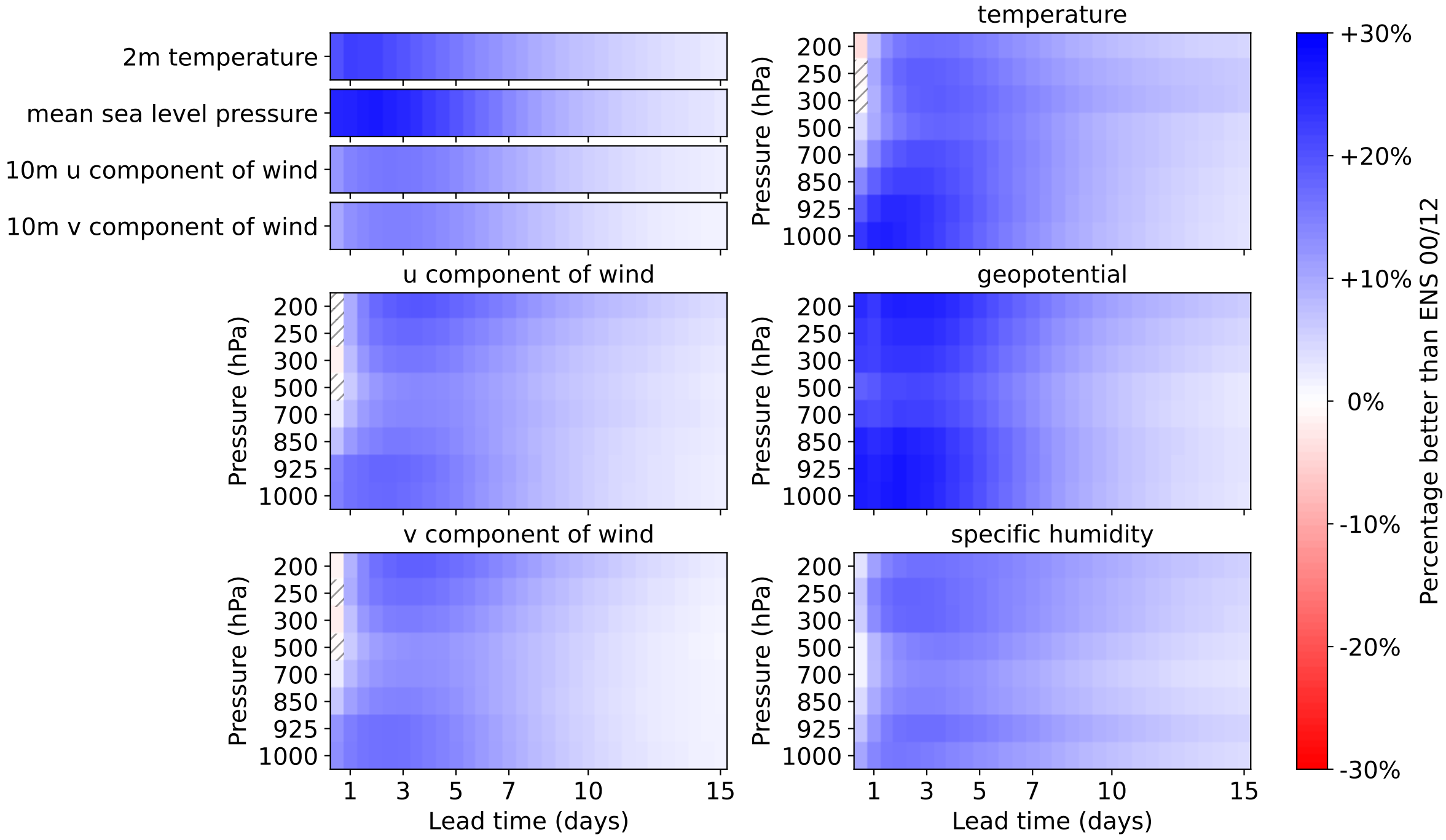

Supplementary Figure 1: **CRPS comparison of WN-C to ENS.** CRPS scorecard comparing WN-C to ENS, on all 00/12 initialisation times in 2023, against HRES-fc0 ground truth. WN-C achieves better CRPS on 99.2 % of targets ($p < 0.05$), with an average improvement of 10.8 % and a maximum improvement of 27.6 %. Hatching indicates scorecard cells where the difference is not statistically significant ($p \geq 0.05$). Notably, while GenCast suffered compared to ENS at short lead times, WN-C is strong across all lead times.

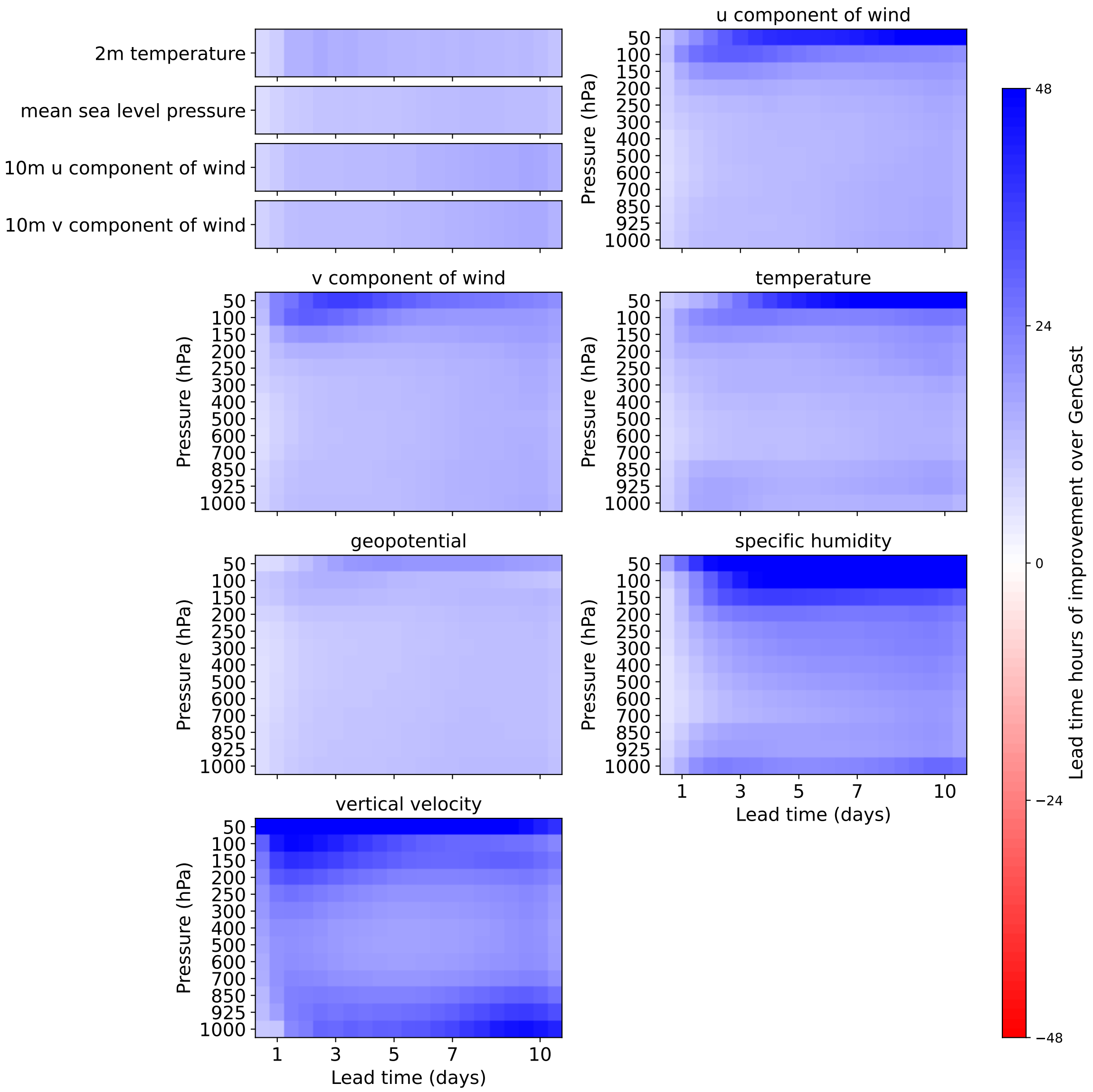

Supplementary Figure 2: **Lead time hours of advantage of WN-C compared to GenCast.** Lead time hours of CRPS advantage scorecard comparing WN-C to the currently operational version of GenCast on all initialisation times in 2023, against HRES-fc0 ground truth. Note the color-bar limits of 48 h (some higher level atmospheric variables still saturate at this threshold) and lead time truncation to 10 days. WN-C has an average advantage of 18.8 h of lead time.

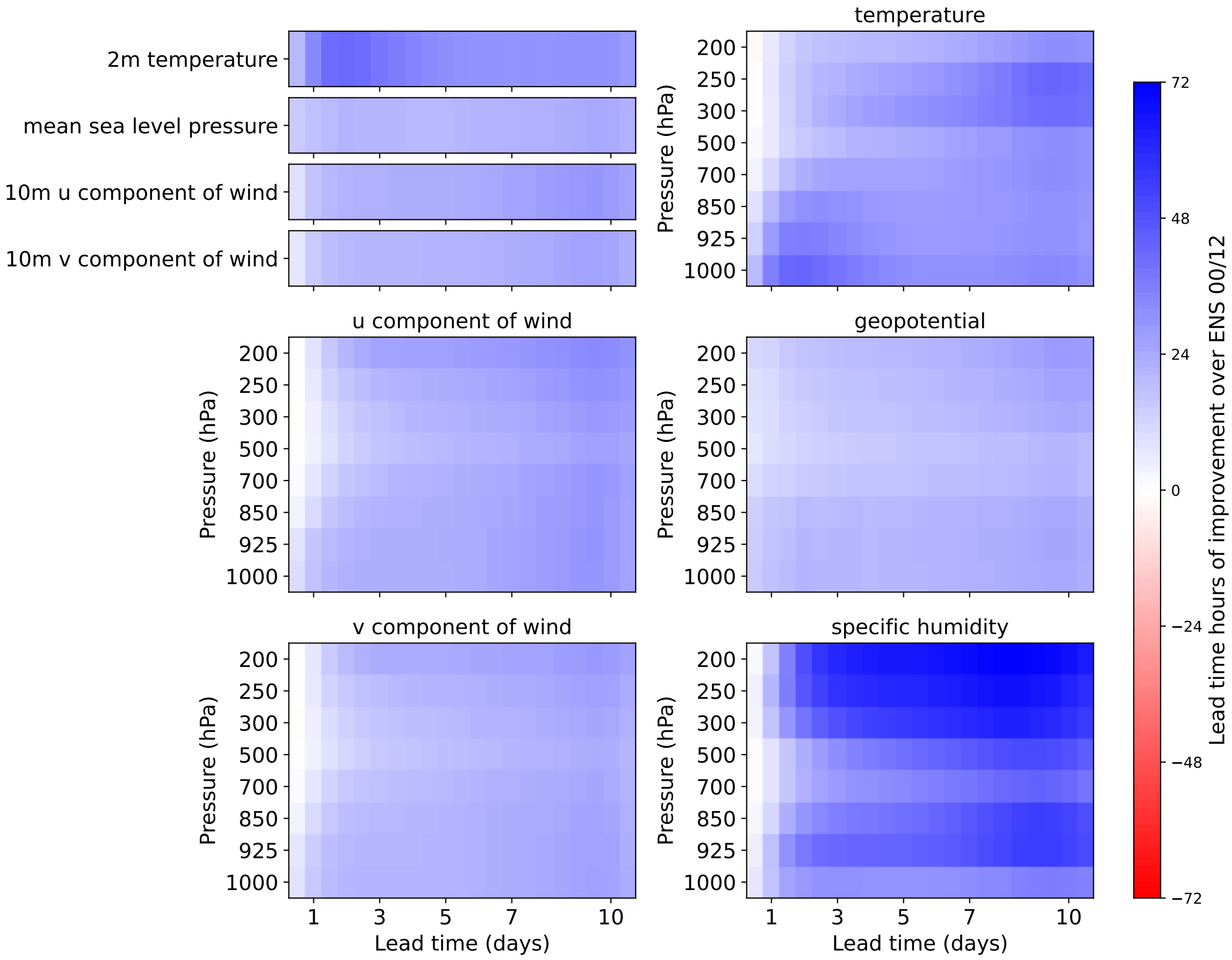

Supplementary Figure 3: **Lead time hours of advantage of WN-C compared to ENS.** Lead time hours of CRPS advantage scorecard comparing WN-C to ENS, on all 00/12 initialisation times in 2023, against HRES-fc0 ground truth. Note the color-bar limits of 72 h (some higher level atmospheric variables still saturate at this threshold) and lead time truncation to 10 days. WN-C has an average advantage of 26.2 h of lead time.

max-pooled and average-pooled CRPS for pool sizes ranging from 120 km to 3828 km. Note that when spatial dependencies exist, minimising the CRPS of the marginal predictions is not sufficient to minimise the CRPS of the pooled predictions. Thus, the pooled CRPS provides a useful metric to assess the spatial structure of forecasts' joint distributions. Second, we evaluate the cross-variable dependencies in the forecasts by computing the CRPS for two derived quantities of interest: 10m wind speed (derived from the 10u and 10v predicted fields), as well as the difference in geopotential at pressure levels 300hPa and 500hPa. Evaluating cross-variable dependencies can be done in different ways: we have chosen these because forecasting wind speeds is crucial across many applications, e.g. transportation, while 300hPa to 500hPa geopotential difference is a critical quantity for tropical cyclones predictions as well as weather more generally. Skill in forecasting these derived quantities relies on correctly modelling the dependencies of their constituent predicted fields. Finally, we report the spherical harmonic power spectra of the forecasts and compare it to those of the ground truth HRES-fc0. Power spectra are a standard, albeit imperfect, measure of physical plausibility. They are an important tool to diagnose blurring, a common but "unphysical" way deterministic ML-based weather models achieve lower RMSE.

Across all pool-size, variable, lead time, level combinations, WN-C achieves better CRPS in average-pooled evaluations in 100 % of cases and in 99 % of cases for max-pooled evaluations (Extended Data Figure 6a,b), showing that WN-C skillfully models the relevant spatial correlations in the forecast distribution. WN-C also obtains approximately 10 % and 15 % better CRPS on 10m wind speed and $z300 - z500$ at short lead times respectively, with this improvement decreasing as lead time increases (Extended Data Figure 6c,d). This shows WN-C is able to capture these across-variable dependencies despite not being directly trained on them. Finally, power spectra of WN-C's forecasts generally match well with those of the ground truth analysis (Extended Data Figure 6e–j). In some variables (e.g. z500), WN-C has a spike at the mesh frequency and some additional high frequency content, more so than GenCast, but orders of magnitude smaller than the dominant powers in the signal.

2.3 Extended comparisons to other baselines

In the main text we compare against representative state-of-the-art models from different categories: global numerical models (ECMWF ENS), regional models (HAFS-A, denoted HAFS) and AI models (GenCast). We note that ENS increased its resolution from 0.2° to 0.1° in June 2023[95]; about three quarters of global cyclones and almost all the North Atlantic and Eastern Pacific test set evaluate the post-upgrade model. We choose these models because they are representative of the best performance on the test years of 2023 and 2024, across the main variables of interest, that is: track error, intensity error and quadrant radius error.

Here, we also compared WN-C against a range of additional models that are widely used in the literature, in particular: ECMWF HRES, GEFS (Supplementary Figure 5), and HAFS-B, HMON, and HWRF (Supplementary Figure 6).

GenCast with the pressure-wind speed relation versus raw TempestExtremes. DeMaria et al.[99] showed that standard global weather forecasting models have very low skill in forecasting cyclone intensity, partly because they are strongly low-biased. This bias arises because surface winds in gridded analyses are too spatially coarse to resolve the true maximum wind speed of a cyclone; a standard tracker such as TempestExtremes therefore inherits this low bias by simply reading off an inherently underestimated wind field. To strengthen the ML baseline, we augmented the TempestExtremes tracker to use the Atkinson–Holliday pressure–wind speed relation[100] and optimised its parameters to obtain a more accurate estimate of maximum wind speed. The rationale

is that maximum wind speed is well correlated with the minimum central pressure, and tuning the relation's parameters helps reduce the systematic bias. Supplementary Figure 4 shows that using the pressure–wind speed relation with optimised parameters significantly improves both the MAE and bias of the intensity forecasts.

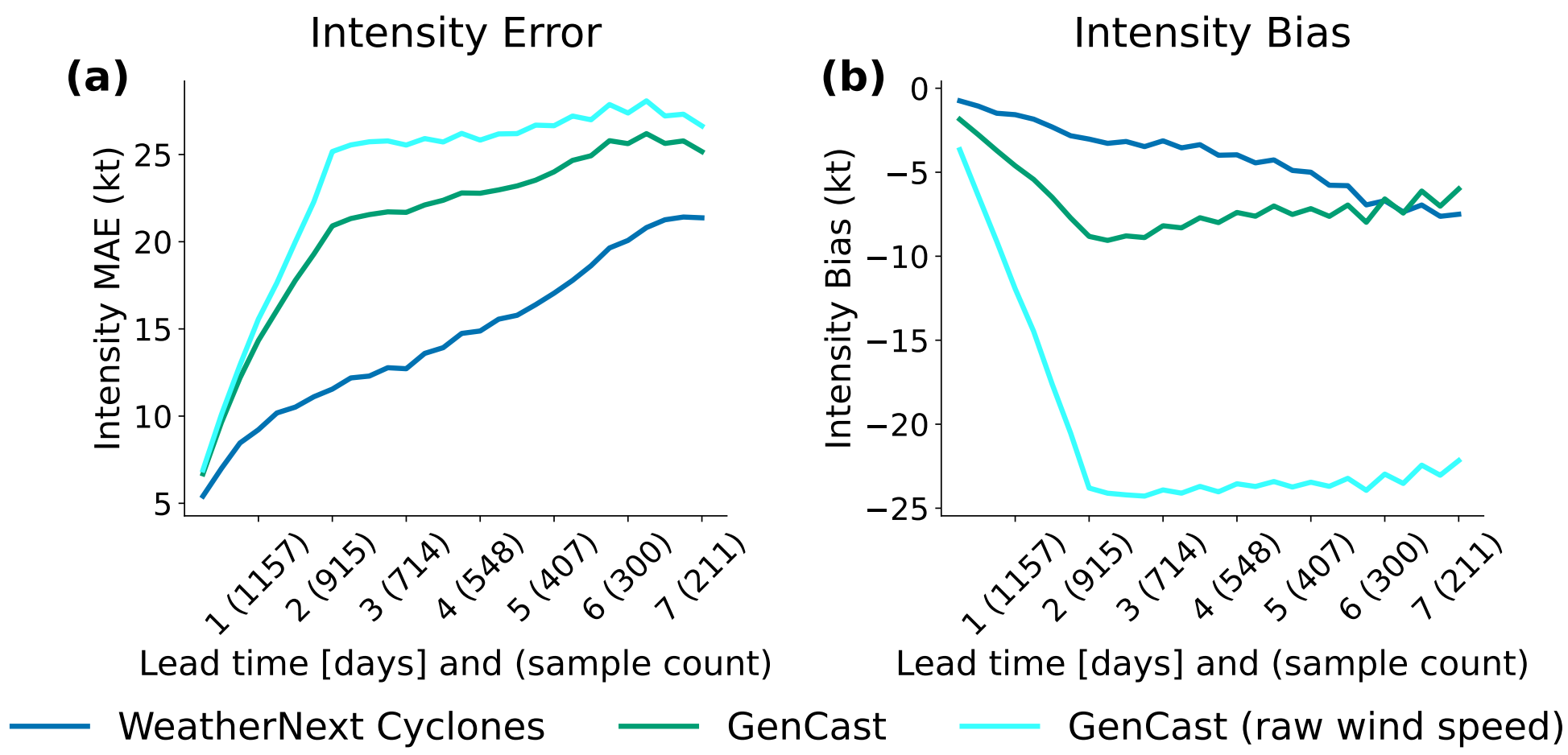

Supplementary Figure 4: **Learning a pressure–wind speed relation improves intensity error and bias for GenCast.** **a**, On intensity error, the pressure-wind speed relation significantly improves the intensity forecasts of GenCast over the raw pressure-wind speed. **b**, The pressure-wind speed relation significantly reduces the bias of the intensity forecast.

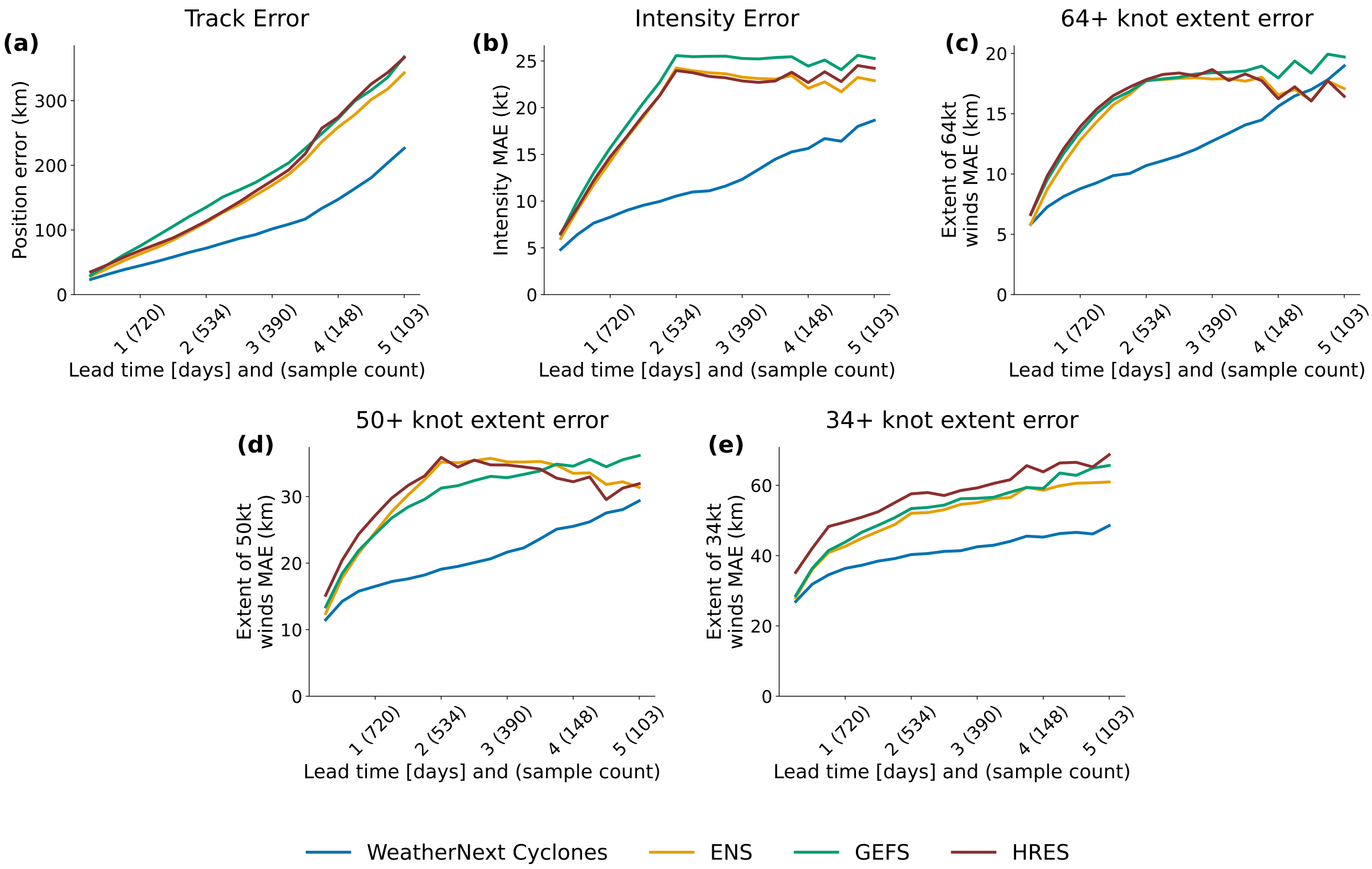

Supplementary Figure 5: **WN-C improves across global baselines in track, intensity and wind radii forecasts.** Deterministic track, intensity and extent error on North Atlantic and East Pacific basin on 2023 and 2024 with a homogeneous comparison of multiple global NWP baselines and WN-C. **a**, On track error, WN-C outperforms all NWP baselines at all lead times. ENS achieves slightly lower track error than GEFS across all lead times, and lower than HRES from 3.5 days. **b**, On intensity error, WN-C outperforms all NWP baselines at almost all lead times. ENS outperforms GEFS across all lead times in this homogeneous evaluation, achieving similar performance to HRES. **c,d,e**, On extent error, WN-C outperforms all of ENS, HRES, and GEFS, which perform roughly on par with one another for 64+ knot winds. GEFS performs slightly better than HRES and ENS on 50+knot extent, and GEFS and ENS marginally outperform HRES on 34+ knot extent.

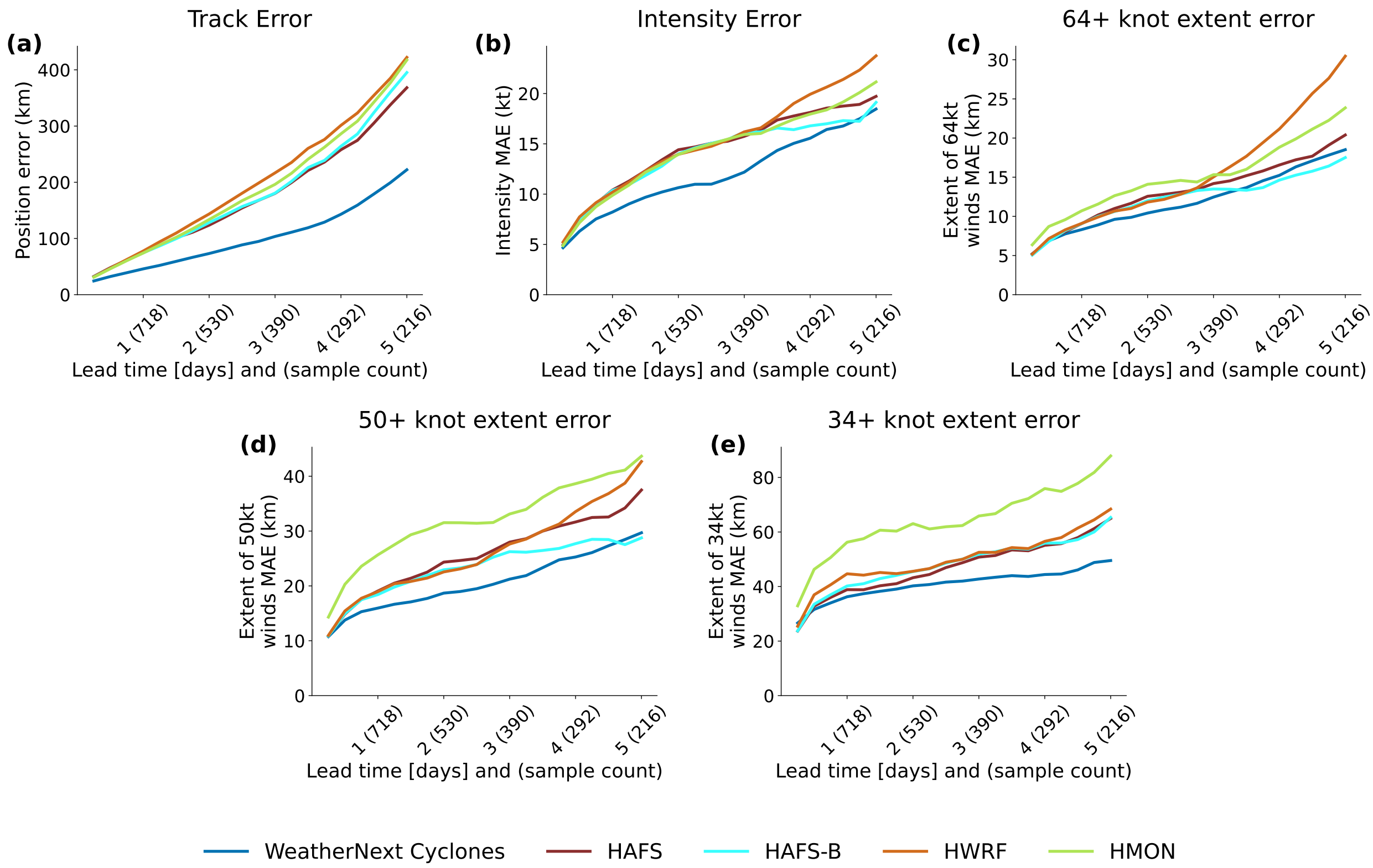

Supplementary Figure 6: **WN-C improves across regional baselines in track, intensity and wind radii forecasts.** Deterministic track, intensity and extent error on North Atlantic and East Pacific basin on 2023 and 2024 with a homogeneous comparison of multiple regional NWP baselines and WN-C. **a**, On track error, WN-C outperforms all NWP baselines at all lead times. HAFS, especially HAFS-A, had better track error than other regional models. **b**, On intensity error, WN-C outperforms all NWP baselines at almost all lead times. HAFS-A, HAFS-B, and HMON all have similar magnitudes of intensity error. **c,d,e**. On extent error, WN-C outperforms all NWP baselines at all lead times. HAFS-A and HAFS-B have similar errors and outperform HMON at all lead times, and HWRF at longer lead times on 64+ knot extent.

2.4 Detailed tracker and co-training ablations.

The improvement of WN-C over a model trained only on analysis data arises from two main sources: 1) the additional cyclone-specific training signal improving the model's hidden representations of cyclones, and 2) the migration to learned output channels and direct tracker extraction algorithm (i.e. 'learned tracker'; see Methods Section 10.3) which generate outputs that are more representative of real IBTrACS data.

While the combined impact of both co-training and the learned tracker relative to the analysis-only baseline is shown in Extended Data Figure 4, we present here two detailed ablations to isolate their individual contributions. First, comparing tracks generated by applying the analysis-based tracker to WN-C against an analysis-only baseline (FGN) shows that co-training with IBTrACS alone yields marginal but statistically significant improvements in track position error at late lead times (Supplementary Figure 7). Second, comparing the direct tracker to the analysis-based tracker shows systematic gains in intensity (Supplementary Figure 8). The direct tracking algorithm also yields small but significant track accuracy gains at early lead times due to the its ability to make sub-grid predictions; given that the grid resolution is 0.25° and overall tracking errors are low, discretisation error constitutes a substantial portion of the baseline tracker's error.

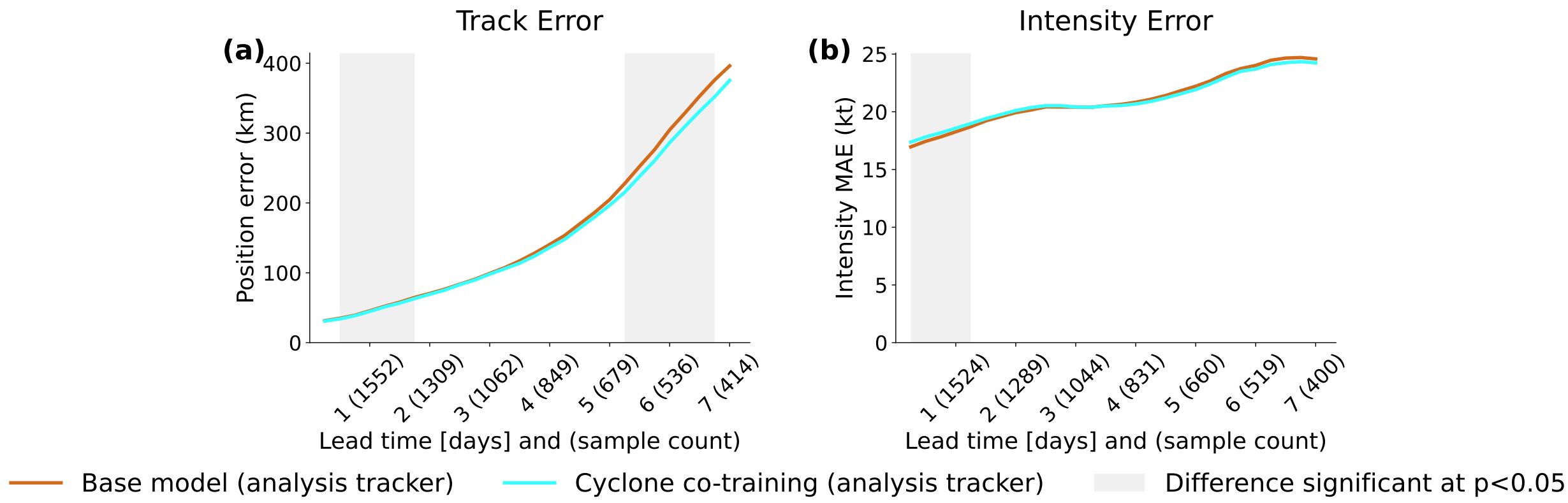

Supplementary Figure 7: **Co-training WN-C on IBTrACS improved track error.** Mean track error (**a**) and intensity MAE (**b**) comparing WN-C to FGN (analysis only), both using the TempestExtremes tracker. Grey shading denotes lead times where the difference is statistically significant at $p < 0.05$. Co-training with IBTrACS results in marginal but statisitically significant improvements in analysis-based position error at lead times of 5.25 days to 7 days, and negligible changes in minimum sea level pressure-based intensity error. Evaluated on 2023 without earlification.

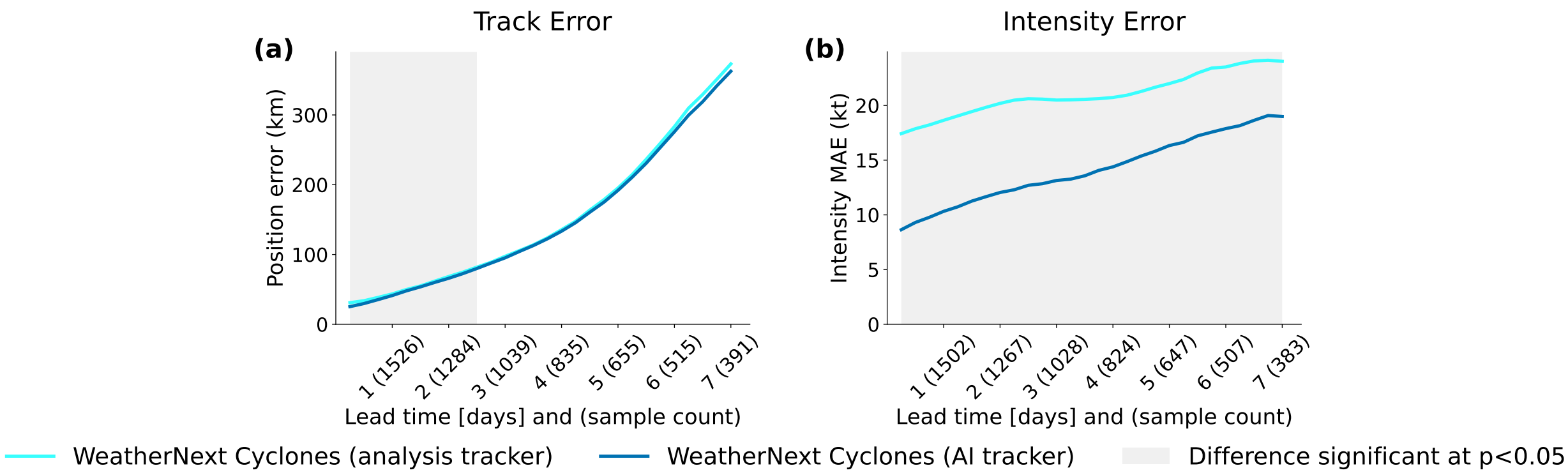

Supplementary Figure 8: **Direct intensity prediction with WN-C significantly improved forecast skill.** Mean track error (**a**) and intensity MAE (**b**) comparing WN-C (direct tracker) to WN-C (TE), both applied to the same neural network co-trained on IBTrACS. Grey shading denotes lead times where the difference is statistically significant at $p < 0.05$. The learned tracker further improves track accuracy and provides consistent intensity gains. Evaluated on 2023 without earlification.

2.5 Extended wind speed probability results

Extended Data Figure 7 and Supplementary Figure 9 show that WN-C provides substantial value compared to ENS on many lead times. Supplementary Figures 10 and 11 show that the track probabilities produced by WN-C are more reliable than those produced by both GenCast and ENS, and similarly, its wind speed probabilities are more reliable than those of ENS.

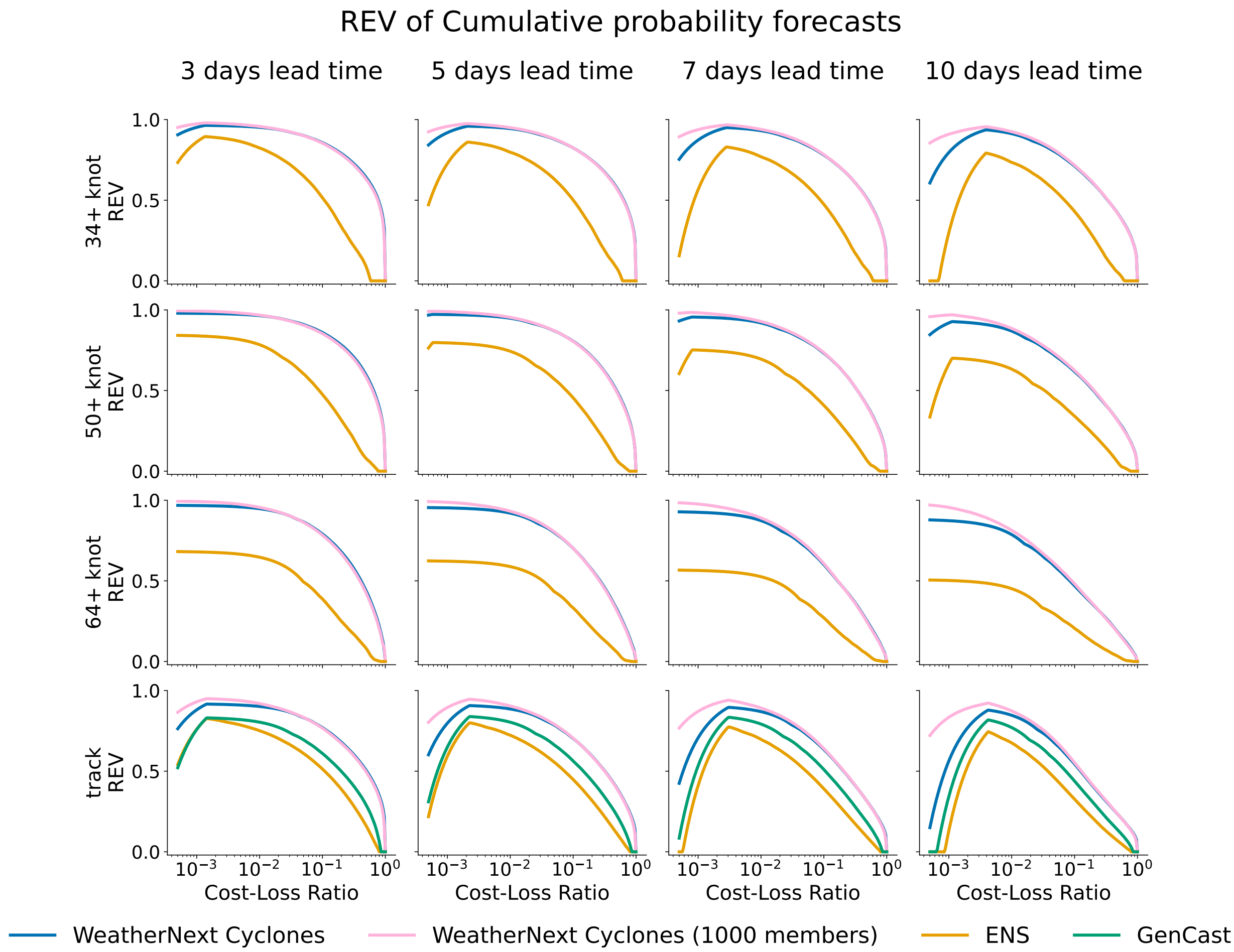

Supplementary Figure 9: **WN-C significantly improves on cumulative probability REV over GenCast and ENS.** Relative economic value (REV) across multiple lead times for track probability and 34+ knot, 50+ knot, and 64+ knot cumulative wind speed probabilities. The 7 day and 10 day lead times show results only for 00 UTC and 12 UTC initialisations because ENS's 06 UTC and 18 UTC initialisations stop at a 6.25 days lead time. GenCast only appears in the bottom row, as it does not predict wind radii.

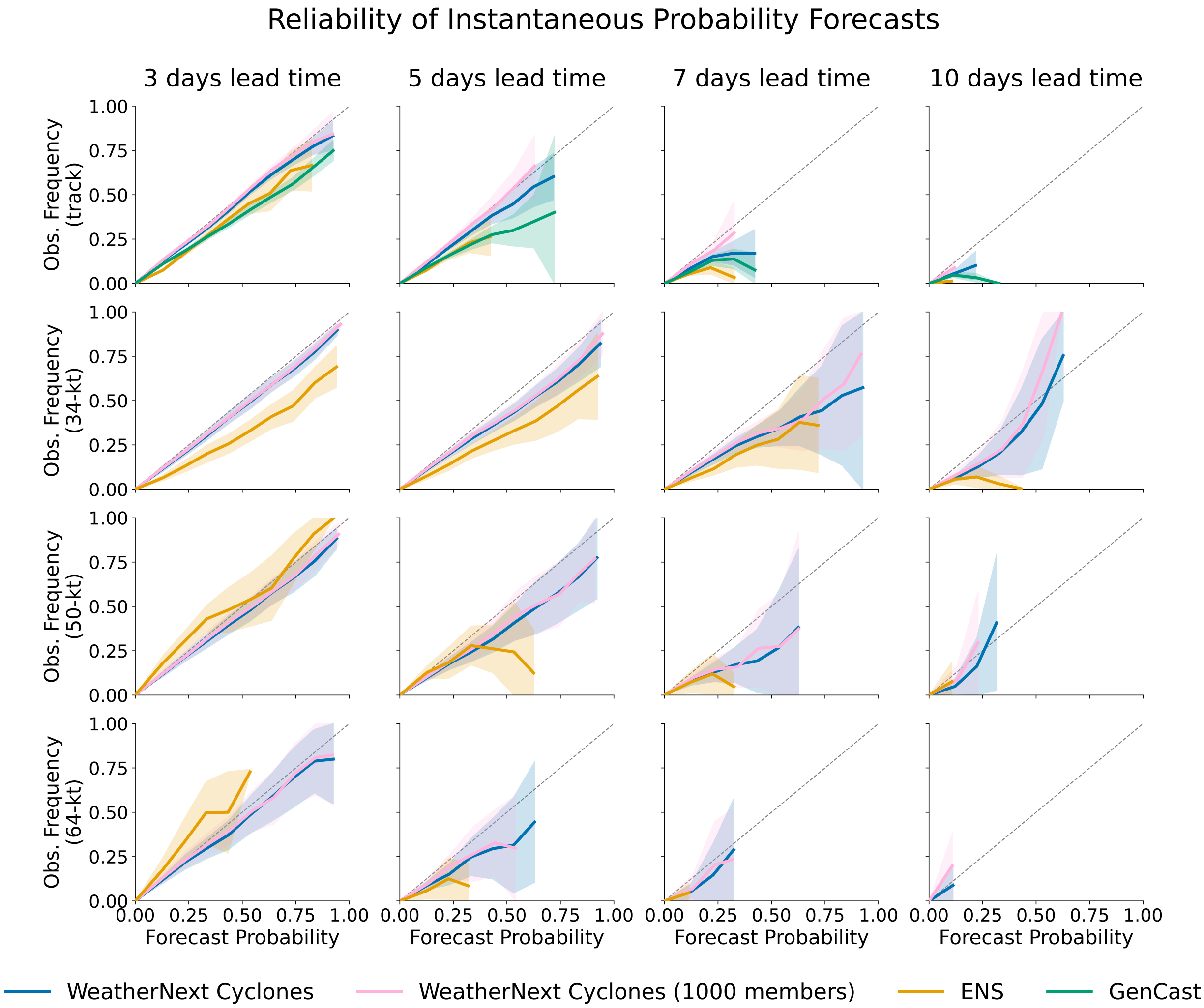

Supplementary Figure 10: **Reliability plots for instantaneous track and wind speed probabilities.** The 7 day and 10 day lead times show results only for 00 UTC and 12 UTC initialisations because ENS's 06 UTC and 18 UTC initialisations stop at a 6.25 d lead time. GenCast only appears in the bottom row, as it does not predict wind radii. Error bars show 95% confidence intervals as determined by the method explained in Supplementary Section 2.6.

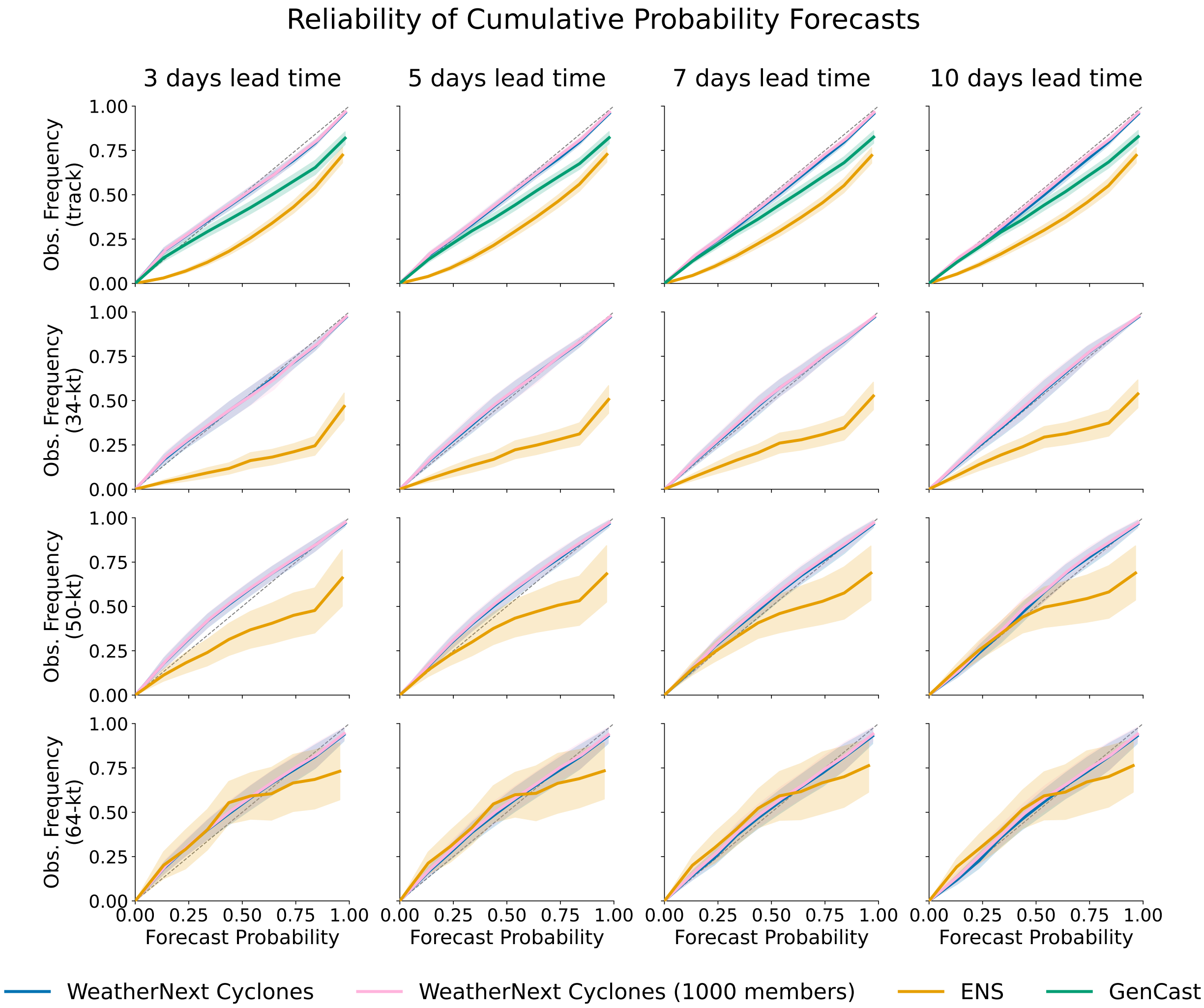

Supplementary Figure 11: **Reliability plots for cumulative track and wind speed probabilities.** The 7 day and 10 day lead times show results only for 00 UTC and 12 UTC initialisations because ENS's 06 UTC and 18 UTC initialisations stop at a 6.25 days lead time. GenCast only appears in the bottom row, as it does not predict wind radii. Error bars show 95% confidence intervals as determined by the method explained in Supplementary Section 2.6.

2.6 Statistical significance tests

To test for significance of differences in our paired-cyclone evaluation metrics, we perform bootstrap tests using 1000 replicates resampled at the level of cyclones, as described in refs. 82 and 86. For unpaired cyclone evaluation metrics, which are each a function of the mean of a multivariate time-series of spatially-aggregated statistics, we account for autocorrelation by performing Heteroskedasticity and Autocorrelation Robust (HAR) inference using the equal-weighted cosine (EWC) covariance estimator with settings recommended by Lazarus et al.[101]. We then apply the multivariate delta method to construct confidence intervals and p-values for a (potentially non-linear) function of the mean. The lead times at which the difference between two models' metrics is statistically significant at $p < 0.05$ are shown in Supplementary Figures 12 to 15.

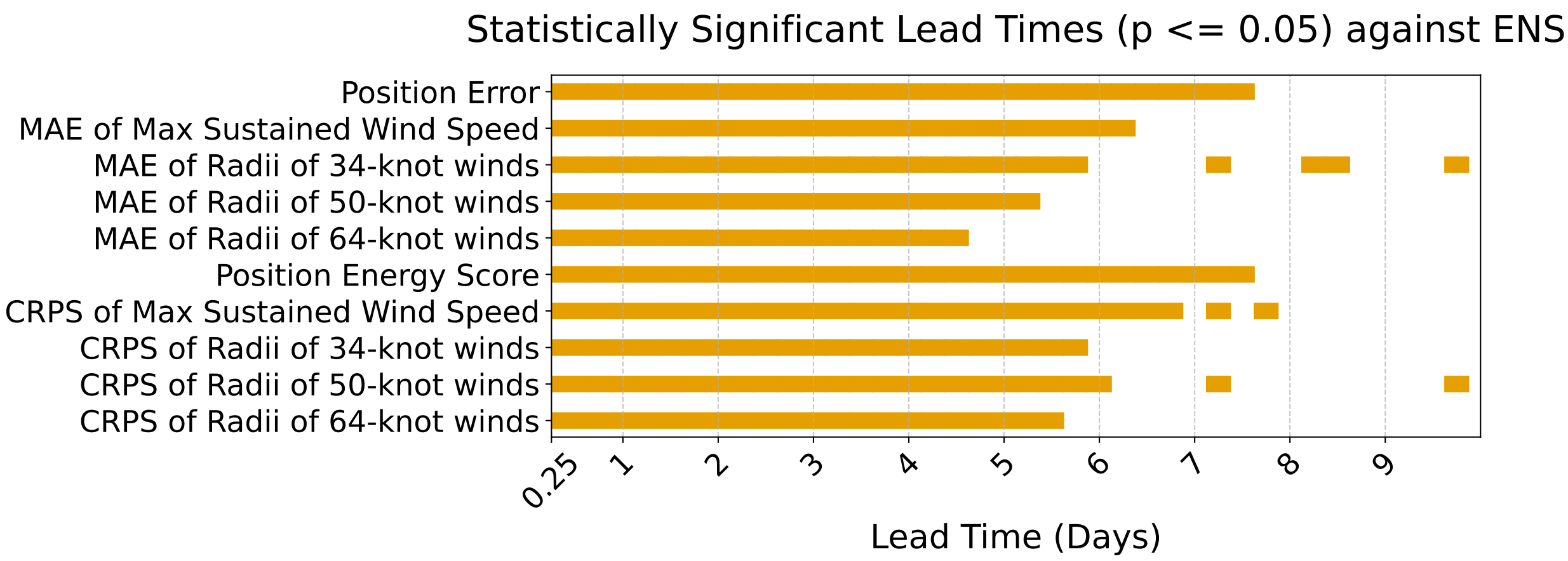

Supplementary Figure 12: **Statistical significance of improvements from WN-C over ENS.** Lead times at which the difference in various paired metrics achieved by WN-C and ENS are statistically significant at $p < 0.05$.

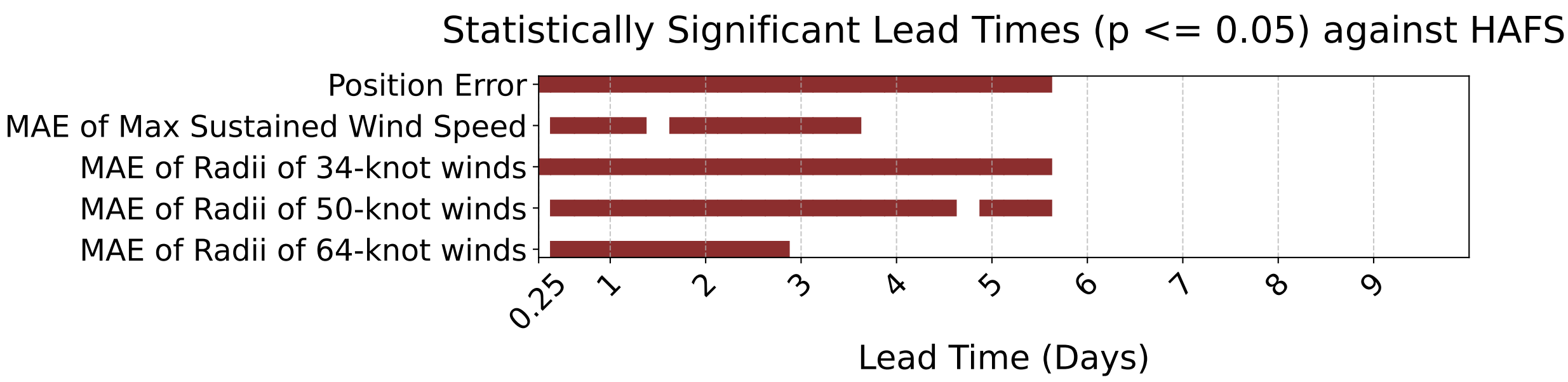

Supplementary Figure 13: **Statistical significance of improvements from WN-C over HAFS.** Lead times at which the difference in various paired metrics achieved by WN-C and HAFS are statistically significant at $p < 0.05$.

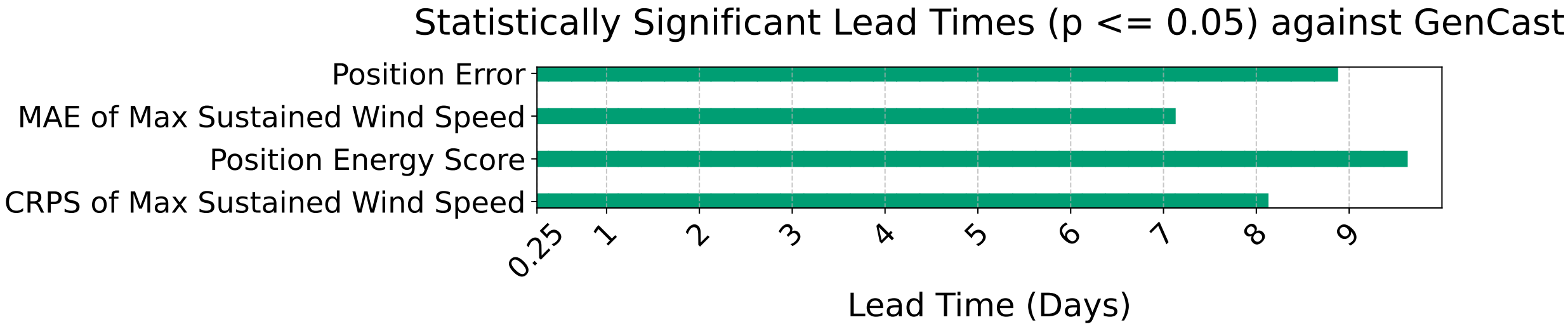

Supplementary Figure 14: **Statistical significance of improvements from WN-C over GenCast.** Lead times at which the difference in various metrics achieved by WN-C and GenCast are statistically significant at $p < 0.05$.

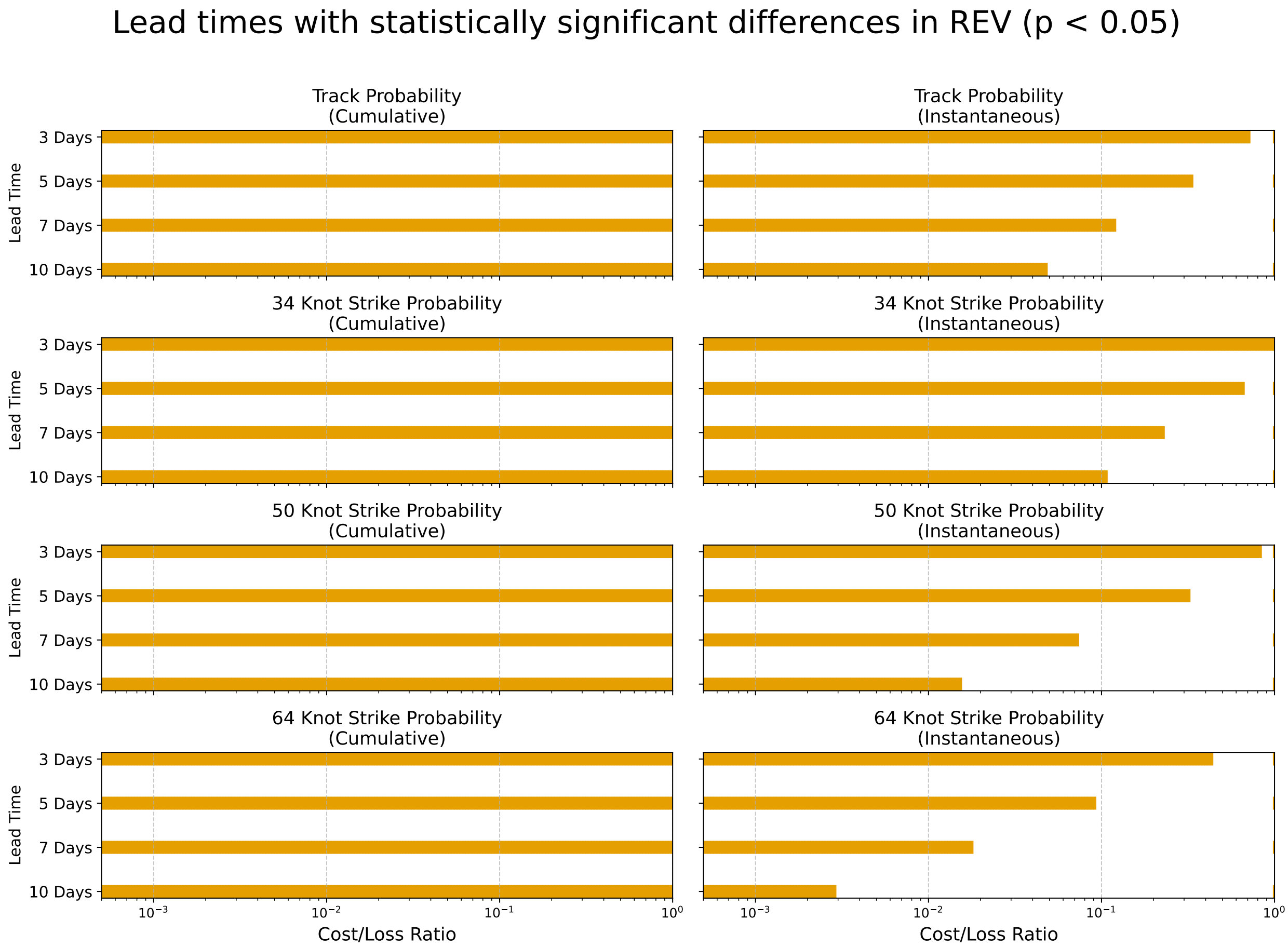

Supplementary Figure 15: **Statistical significance of improvements from WN-C over ENS on track and strike probabilities.** Lead times at which the difference in REV achieved by WN-C and ENS are statistically significant at $p < 0.05$.

2.7 Historical rate of progress in cyclone prediction

This section contextualises the gain in WN-C performance over NWP baselines by comparing it with the historical improvement of those NWP models over time. This allows the rate of progress enabled by WN-C to be quantified in terms of years of traditional physics-based modelling research and development.

2.7.1 Improvements in cyclone track prediction

Global NWP models have improved over time due to advances in high performance computing, the global observing system, and data assimilation. For example, NWP predictions of large-scale atmospheric patterns have gained approximately one day of useful forecast lead time per decade[102]. This large-scale steering flow dictates tropical cyclone movement. The resulting improvement to NWP cyclone track guidance used by human forecasters has been credited with commensurate 'one day per decade' gains in the accuracy of cyclone track forecasts issued by the U.S. National Hurricane Center[103,104] and forecast agencies in the Western North Pacific[105].

To measure the rate of progress of ECMWF ENS's ensemble mean track, we evaluate its ensemble mean position error globally over two multi-year periods centred one decade apart: 2005–2014 and 2015–2024 (Extended Data Figure 8a). Aggregating over cyclones from multiple years accounts for interannual variability in forecast skill. At a 300 km position error threshold, ENS gained about 22 h of lead time with a 65 km position error reduction (Extended Data Figure 8b). A homogeneous evaluation of WN-C and ENS's late ensemble mean position error over 2023–2024 shows that WN-C gains 27 h of lead time and a 100 km position error reduction. WN-C, which benefited from the advances that drove ENS's cyclone track prediction improvements via its ECMWF analysis training data, thus unlocks more than a decade of cyclone track prediction progress relative to ENS.

2.7.2 Improvements in cyclone intensity prediction

While track forecasts have seen steady improvement for decades (Section 2.7.1), intensity forecasting has historically remained a major challenge[106]. Official NHC hurricane intensity forecast errors did not improve substantially between 1970 and 2010 in the Atlantic basin[107]. This trend changed around 2010 with the launch of the Hurricane Forecast Improvement Project (HFIP), which invested heavily in the Hurricane Weather Research and Forecasting (HWRF) system[108], an ocean-coupled, regional, high-resolution hurricane NWP model developed by NOAA, which is credited with driving substantial improvements in NHC official intensity forecasts between 2010 and 2020[104].

HWRF has been superseded by newer hurricane models like HMON[109] and HAFS[110]. However, as the longest continually running regional hurricane model, HWRF provides a record on the historical rate of progress of a single flagship regional hurricane NWP model. We estimate the historical rate of progress of NWP-based intensity prediction using HWRF in the Atlantic and East Pacific basins where it was operationalised. Similar to the evaluation in Section 2.7.1, we compare HWRF's (late) intensity error over two multi-year periods centred one decade apart: 2009–2014 and 2019–2024. HWRF gained approximately 31 h of lead time at a 15 kt error threshold between these two periods (Extended Data Figure 9b). Conducting a homogeneous evaluation over 2023–2024, WN-C's late ensemble mean achieves a lead time gain of 40 h and a 4 kt error reduction relative to the operational HWRF baseline at the same error threshold (Extended Data Figure 9c). WN-C thus provides a step-change in intensity forecast skill exceeding the gains achieved over a decade by the premier regional hurricane model.

2.8 Geospatial decomposition of deterministic skill improvements

To showcase the performance of WN-C across different basins and conditions, we evaluate WN-C's improvements in position and intensity errors over ENS and HAFS as a function of the observed cyclone location, averaging over all initialisation times for which both models predicted the cyclone at that valid time, i.e. a homogeneous evaluation (Supplementary Section 1.5.1). This evaluation does not perform storm stage subsetting (Supplementary Section 1.5.1) to maximize geospatial coverage.

We observe mostly uniform improvements over ENS, for both track and intensity, across basin and observed cyclone, with only a few rare instances where WN-C's track or intensity was much worse than ENS (Extended Data Figure 10 and Supplementary Figure 17). The same is seen for WN-C versus HAFS on position error (Supplementary Figure 16). Comparisons with HAFS on intensity are more nuanced, with more frequent cases where HAFS has a lower intensity error (Extended Data Figure 11). There is no clear dependence of skill difference on ocean basin or proximity to land.

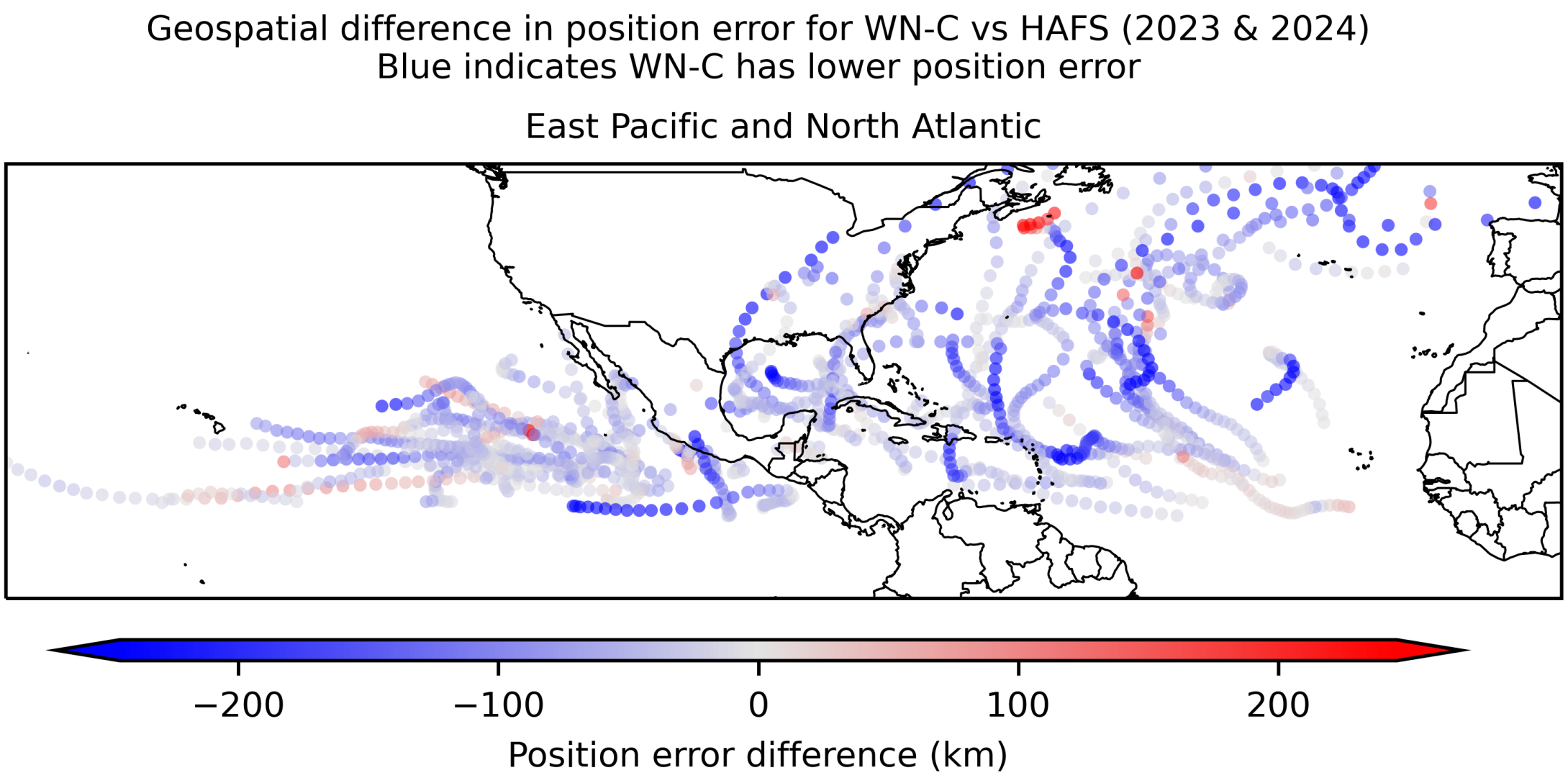

Supplementary Figure 16: **Geospatial illustration of WN-C vs HAFS position error.** Geospatial difference in ensemble mean position error for the interpolated homogeneous comparison, averaged over all lead times. The colour bar saturates at 95 % of the maximum absolute difference. Note that the HAFS data from the NHC a-decks is only available in the East Pacific and North Atlantic. Base map data provided by Natural Earth.

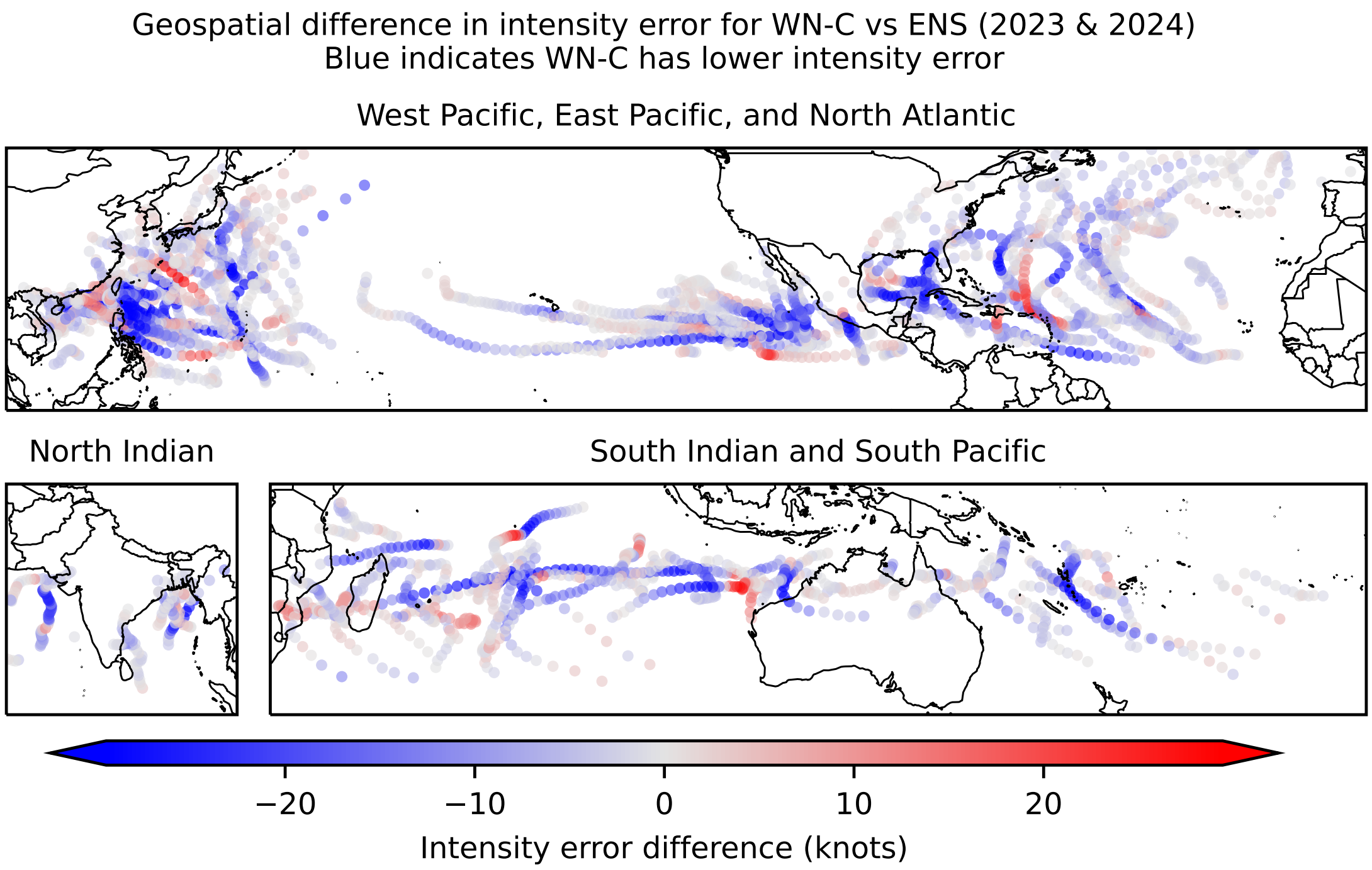

Supplementary Figure 17: **Geospatial illustration of WN-C vs ENS intensity error.** Geospatial difference in ensemble mean intensity error for the homogeneous comparison between WN-C and ENS, averaged over all lead times. Both models use early (interpolated) forecasts. The colour bar saturates at 95 % of the maximum absolute difference. Base map data provided by Natural Earth.

2.9 WeatherNext Cyclones Mini

Alongside the full-size model, we also trained and are open-sourcing a light-weight version, WN-C Mini. Both are available at https://github.com/google-deepmind/weathernext. The smaller version is much cheaper and faster to run, with a 16-layer processor with latent size of 512 and, and operating at 1° resolution. It is also a single model seed instead of four. This is intended for researchers or others who want to run the model themselves, but who have limited computational resources. The training stages were the same as that of the full model, except for the fact that stage 3 was skipped, and stage 4 onwards were performed at 1° resolution.

Though it performs worse than the full-sized WN-C model, WN-C Mini still significantly outperforms prior state-of-the-art across many metrics (Supplementary Figures 18 and 19).

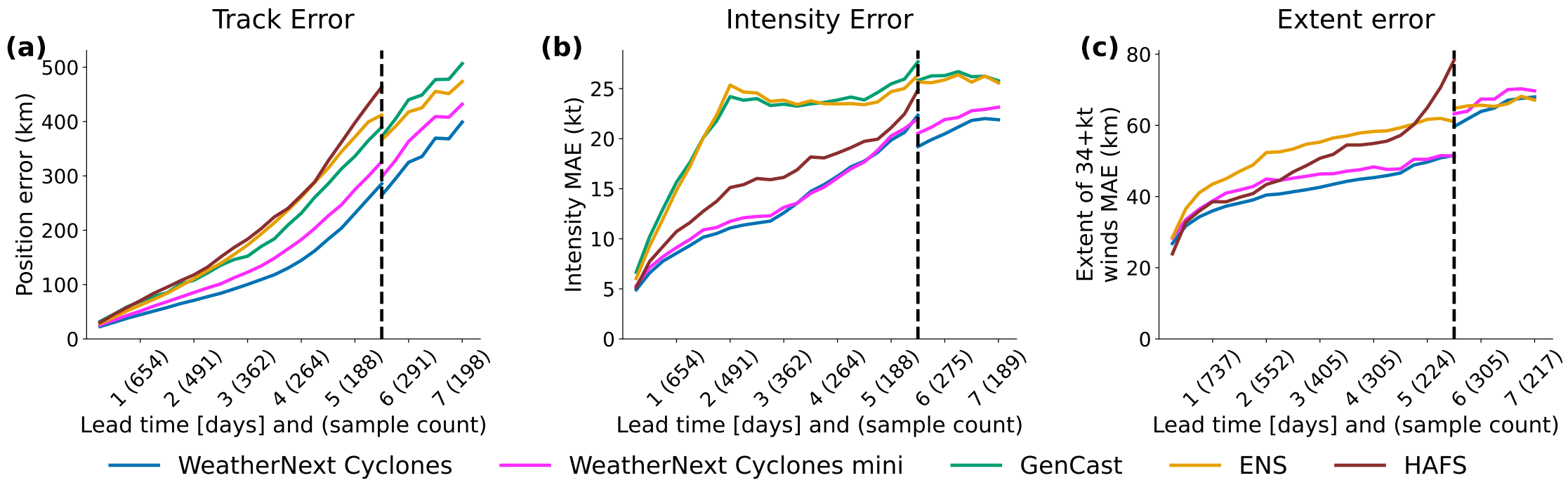

Supplementary Figure 18: **WN-C Mini deterministic paired evaluation.** WN-C Mini performs worse than WN-C but mostly better than prior models on MAE of track, intensity, and extent predictions, despite having an input/output resolution of only 1°.

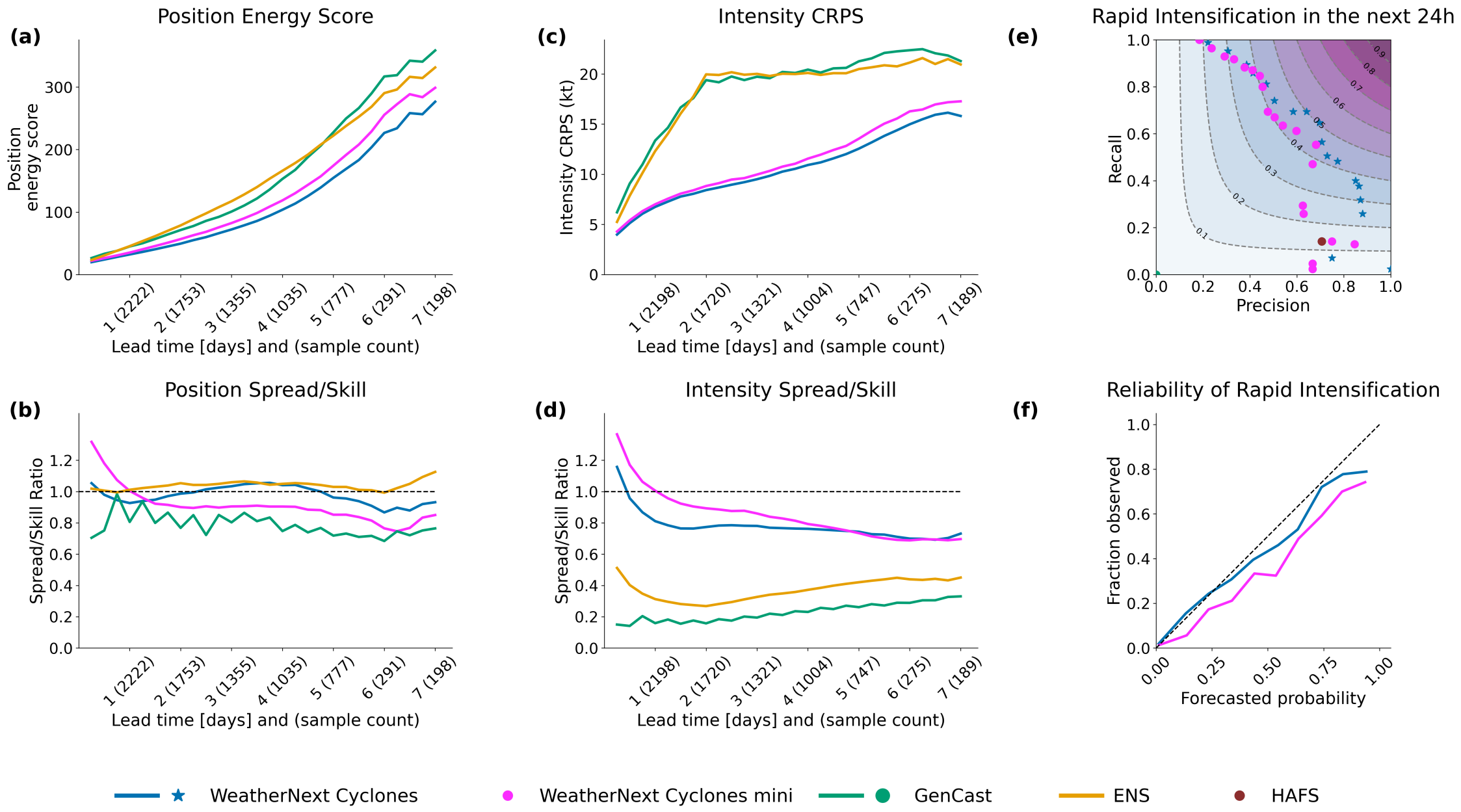

Supplementary Figure 19: **WN-C Mini probabilistic paired evaluation.** WN-C Mini performs worse than WN-C in probabilistic paired evaluations but mostly better than prior models, despite having an input/output resolution of only 1°. Overdispersion at early lead times is explained by there being higher relative uncertainty for earlified forecasts with lower resolution inputs, and this short-leadtime overdispersion also explains the degradation in reliability of Rapid Intensification forecasts shown in panel (f).

Supplementary References

[82] Lam, R. *et al.* Learning skillful medium-range global weather forecasting. *Science* eadi2336 (2023).

[83] Loshchilov, I. & Hutter, F. Decoupled weight decay regularization. In *International Conference on Learning Representations* (2018).

[84] Gneiting, T. & Raftery, A. E. Strictly proper scoring rules, prediction, and estimation. *Journal of the American statistical Association* 359–378 (2007).

[85] Richardson, D. S. Skill and relative economic value of the ECMWF ensemble prediction system. *Quarterly Journal of the Royal Meteorological Society* **126**, 649–667 (2000).

[86] Price, I. *et al.* Probabilistic weather forecasting with machine learning. *Nature* **637**, 84–90 (2025).

[87] Lam, R. *et al.* GraphCast: Learning skillful medium-range global weather forecasting. *arXiv preprint arXiv:2212.12794* (2022).

[88] Price, I. *et al.* GenCast: Diffusion-based ensemble forecasting for medium-range weather. *arXiv preprint arXiv:2312.15796* (2023).

[89] Hersbach, H. *et al.* The ERA5 global reanalysis. *Quarterly Journal of the Royal Meteorological Society* **146**, 1999–2049 (2020).

[90] Rasp, S. *et al.* WeatherBench: a benchmark data set for data-driven weather forecasting. *Journal of Advances in Modeling Earth Systems* **12**, e2020MS002203 (2020).

[91] Knapp, K. R., Kruk, M. C., Levinson, D. H., Diamond, H. J. & Neumann, C. J. The international best track archive for climate stewardship (IBTrACS): Unifying tropical cyclone data. *Bull. Am. Meteorol. Soc.* **91**, 363–376 (2010).

[92] Gahtan, J. *et al.* International best track archive for climate stewardship (ibtracs) project, version 4r01. NOAA National Centers for Environmental Information. doi:10.25921/82ty-9e16 (2024).

[93] Cangialosi, J. P. & Martinez, J. National Hurricane Center forecast verification report. *National Hurricane Center (NHC)* (2024).

[94] ECMWF. *IFS Documentation CY46R1 - Part V: Ensemble Prediction System* (2019). URL https://www.ecmwf.int/node/19309.

[95] ECMWF. *IFS Documentation CY48R1 - Part V: Ensemble Prediction System.* 5 (ECMWF, 2023).

[96] Russell, I. & Kertész, S. Metview (2017). URL https://www.ecmwf.int/node/18136.

[97] Swinbank, R. *et al.* The tigge project and its achievements. *Bulletin of the American Meteorological Society* **97**, 49–67 (2016).

[98] Alet, F. *et al.* Skillful joint probabilistic weather forecasting from marginals. *arXiv preprint arXiv:2506.10772* (2025).

[99] DeMaria, M. *et al.* Evaluation of tropical cyclone track and intensity forecasts from artificial intelligence weather prediction (aiwp) models. *arXiv preprint arXiv:2409.06735* (2024).

[100] Atkinson, G. D. & Holliday, C. R. Tropical cyclone minimum sea level pressure/maximum sustained wind relationship for the western north pacific. *Monthly Weather Review* **105**, 421 – 427 (1977). URL https://journals.ametsoc.org/view/journals/mwre/105/4/1520-0493_1977_105_0421_tcmslp_2_0_co_2.xml.

[101] Lazarus, E., Lewis, D. J., Stock, J. H. & Watson, M. W. HAR inference: Recommendations for practice. *J. Bus. Econ. Stat.* **36**, 541–559 (2018).

[102] Bauer, P., Thorpe, A. & Brunet, G. The quiet revolution of numerical weather prediction. *Nature* **525**, 47–55 (2015).

[103] National Hurricane Center. Verification of the 2024 hurricane season (2024). URL https://www.nhc.noaa.gov/verification/pdfs/Verification_2024.pdf.

[104] Cangialosi, J. P. *et al.* Recent progress in tropical cyclone intensity forecasting at the national hurricane center. *Weather and Forecasting* **35**, 1913–1922 (2020).

[105] Yu, H. *et al.* Are we reaching the limit of tropical cyclone track predictability in the western north pacific? (2022).

[106] Zhang, Z. *et al.* A review of recent advances (2018–2021) on tropical cyclone intensity change from operational perspectives, part 1: Dynamical model guidance. *Tropical Cyclone Research and Review* **12**, 30–49 (2023).

[107] DeMaria, M. Tropical cyclone intensity change predictability estimates using a statistical-dynamical model. In *29th Conference on Hurricanes and Tropical Meteorology* (American Meteorological Society, Tucson, AZ, 2010). URL https://ams.confex.com/ams/29Hurricanes/techprogram/paper_167916.htm. Paper 9C.5.

[108] Alaka, G. J. *et al.* Lifetime performance of the operational hurricane weather research and forecasting model (HWRF) for north atlantic tropical cyclones. *Bull. Am. Meteorol. Soc.* **105**, E932–E961 (2024).

[109] Mehra, A. *et al.* Advancing the state of the art in operational tropical cyclone forecasting at ncep. *Tropical Cyclone Research and Review* **7**, 51–56 (2018).

[110] Alaka, G. J., Zhang, X. & Gopalakrishnan, S. G. High-definition hurricanes: Improving forecasts with storm-following nests. *Bull. Am. Meteorol. Soc.* **103**, E680–E703 (2022).
