# Supplementary material for: Operational tropical cyclone forecasting with AI
Source: Nature. 2026 Aug 6;657(8132):680–8. doi: 10.1038/s41586-026-10953-2 (PMC13581599; doi:10.1038/s41586-026-10953-2)
Supplement: Supplementary file 2 [file 41586_2026_10953_MOESM2_ESM.pdf]

nature portfolio

Peer Review File

Operational tropical cyclone forecasting with AI

Corresponding Author: Dr Ferran Alet

Any redactions in this file are there to maintain patient confidentiality, the confidentiality of unpublished data, or to remove third-party material.

This file contains all reviewer reports in order by version, followed by all author rebuttals in order by version.

Attachments originally included by the reviewers as part of their assessment can be found at the end of this file.

Version 0:

Reviewer comments:

Referee #1

(Remarks to the Author)
Please refer to the attachment for the review comments.

(Remarks on code availability)
The current code provides "high-level" code for the usage of the predictor, the tabular data processing, and the cyclone tracker. While it is helpful for understanding the usage, and the cyclone data processing, it is not enough for a comprehensive understanding of the algorithm, including its model architecture and the training.

Referee #2

(Remarks to the Author)
I co-reviewed this manuscript with one of the reviewers who provided the listed reports.

(Remarks on code availability)
The key algorithms are provided. But the codes cannot be run independently if no information about software/hardware environment and python package dependencies are provided. There are also no enough instructions for installing and running the application. I recommend a Cyclonecast of appropriate size for running can be uploaded. The authors can also upload the Cyclonecast output of atmospheric field X. Then we can run the tracking algorithm by ourselves. I recommend some minimum level of visualization scripts to be provided to show the results.

Referee #3

(Remarks to the Author)
The present manuscript introduces CycloneCast, an AI-based, operational model for forecasting tropical cyclone (TC) intensity, track, and structure. CycloneCast is trained to jointly predict the 0.25°-resolution environment (from ERA5: 6 atmospheric variables on 13 levels and 6 surface variables) and our best record of TC track and intensity (IBTrACS, i.e., tabular data). Interestingly, CycloneCast achieves this by converting IBTrACS into two 0.25° fields: (1) a field indicating the probability the cyclone exists, based on Gaussian blobs centered on the track, and (2) scalars describing TC properties, conditioned on this existence (Section 2.2). Cyclonecst(a large graph neural network-based model, see Section 1.2) is trained to optimize the continuous ranked probability score (CRPS) via a multi-stage training procedure (see Section 12).

According to standard (WeatherBench2-like) evaluation procedures, CycloneCast outperforms the state-of-the-art GenCast in predicting the target global environmental variables (Section B.2). The TC-centric evaluation on IBTrACS uses three baselines (Figures 2–5): ECMWF ENS (considered the best ensemble model we currently have globally for TCs), a debiased Gencst(representative of SOA AI weather models), and NOAA HAFS-A (arguably the top operational baseline for the North Atlantic and East Pacific basins). This evaluation shows significant improvement over existing baselines, especially for intensity and rapid intensification (for which there is currently no equivalent global product), with statistical significance tests reported in Appendix B.

Overall, this is more than just an impressive interdisciplinary collaboration and could fundamentally change the way tropical meteorological forecasts are routinely made, and where resources are invested for further improvement. I've independently evaluated live forecasts of the corresponding FNV model released through WeatherLab as part of research collaborations; live forecasts are significantly harder than standard ML evaluation, and FNV still showed superior probabilistic intensity and rapid intensification forecasts compared with existing models. Given the deadliness and hazard potential of tropical cyclones at the global scale, CycloneCast could very well warrant an eventual Nature publication if other, independent peer reviews concur.
However, given previous advances in AI weather prediction, I am slightly worried about the legacy of this manuscript if published. I therefore mostly focus below on replicability issues and on statements that could limit, rather than encourage, future research in (AI for) tropical meteorology.

I) Major comments

1) General lack of transparency

For the main model, we are missing details that are useful:

(1.1) Exact architecture, exact number of parameters, hardware used, and time for training vs. inference (important for replicability, but also for AI sustainability studies). The manuscript would benefit from a diagram clearly outlining the architecture beyond what is presented in text in Section A.1.
This is especially given the claim around L367 about training efficiency.
Aspects that are already explicit enough could serve as a model for aspects that are less detailed: the CRPS loss is explicit, and Table 1 (p. 29) does a good job of presenting the learning schedule.

(1.2) Some sensitivity/ablation tests would be helpful. For instance, the 120 km ℓ parameter in Eqs. A2 and A3 is central to many of the manuscript's claims/results and seems especially important to test.

(1.3) The geographic validity of each model (e.g., HAFS only works for the NA / maybe EP domain) could be more explicitly stated and discussed. This is a substantial advantage of CycloneCast.

(1.4) Is there a risk of erroneous transfer learning from basins with large sample size and high data quality (North Atlantic) to, e.g., North or South Indian Ocean cases? This could be mentioned more explicitly.

(1.5) The authors mention do not mention why, during training, they chose to use only two members in the ensemble (L169–170) to calculate the CRPS (and not more). Leutbecher et al. (2018) does explicitly mention that at least two members are needed: "Moreover, the ensemble must have at least two members."

(1.6) Related but minor: It would be helpful to have a citation for the fair CRPS, e.g., Ferro et al. (2013).

(1.7) For TC track evaluation (Equation A1): is the energy score calculated only for the position (track), or over the entire set of predicted TC characteristics?
- If the former, is it computed with Euclidean distance in degrees (or some great-circle metric distance)?
- If the latter, was the calculation done over normalized values?
Additionally: if using the fair variant, does one get $1/N$ in front of term 1 and $1/(2 \cdot N \cdot (N-1))$ in front of term 2, or something different? It would be helpful to make it explicit.

(1.8) Importantly: there is no explicit mention of the reliance on US-reported values in IBTrACS. While it is often a necessary choice to avoid conflicting definitions of TC intensity, this choice and its consequences on data quality in non-US basins should be explicitly discussed.

(1.9) Also, there is a major abstract claim of extending forecasts up to 15 days, but little evidence in the text past 5 days. Would it be possible to clarify this aspect?

2) Giving an impression of "solved problem", potentially impeding future research in tropical meteorology

(2.1) Limitations and outlook are glossed over (there is a quick, appreciated nod to "IBTrACS should be maintained"), and this is especially visible in the conclusion section. Especially given the current defunding of some of the public weather enterprise, not explicitly mentioning the work's limitations is dangerous and a potential creativity killer for future research. Especially given the manuscript's potential impact, would it be possible to list potential axes of improvement for future research and operations?
Seeing the results, ideas include:
- Challenges in data assimilation: is the current HRES situation satisfactory, or should we move toward end-to-end observation-to-TC forecasting, or both?
- Global data quality discrepancies: more discussion of issues related to better quality data in the NA/EP basins, as well as limitations related to using the US definition of intensity.
- While wind speed, pressure, track, and structure are discussed, there is still a need to work on precipitation, which is only briefly mentioned in the conclusion.

(2.2) In general, especially given the large interdisciplinary coauthor list, it would be helpful to discuss what could be next for

“AI for tropical meteorology”? This is especially important to avoid giving the impression that the problem is fully solved as it’s unlikely that this work already reaches the intrinsic limit of TC predictability.

(2.3) Is there a plan to release the training/evaluation code for the global tropical meteorology community to make progress? The risk is that this code stays confined within the NHC/DeepMind communities, which would hinder global progress for the regional prediction centers.

3) Clarifications related to tropical cyclogenesis

(3.1) Around L146, the manuscript claims CycloneCast “handles tropical cyclogenesis”, which is potentially impactful but also risky as written: there is limited consensus on an exact definition of TCG (and different communities/datasets define “genesis” differently), so the current phrasing reads stronger than what is explicitly demonstrated.

(3.2) Given the clear potential and the importance of cyclogenesis, I would encourage the authors to add a concise dedicated appendix/figure that (i) states the specific definition(s) of genesis they adopt (with caveats), and (ii) explains how this maps onto the model outputs. For instance, the authors could consider framing the Gaussian-blob “existence probability” field as a proxy for a TC seed, with an explicit tracking/thresholding rule that turns this field into a “genesis event” (L726 could be clearer on this proxy interpretation).

(3.3) Finally, since genesis is discussed (10.3.3) but not evaluated, I would ask for at least one targeted genesis evaluation under the chosen definition (timing/location error and simple event-based metrics would already help), ideally stratified by basin.

4) Lack of details regarding TC structure

(4.1) It is currently unclear how the manuscript converts IBTrACS wind radii by quadrant into a continuous near-surface wind field (e.g., what is shown in Fig. 1). Because this mapping is not unique, would it be possible to state the exact assumptions, parametric form (and any smoothing/interpolation across quadrants) in the appendices/SI?

(4.2) Relatedly, the manuscript appears to rely on a simple albeit dated wind-pressure relationship; given how central intensity/TC structure is to the paper, I would encourage testing more recent wind-pressure relationships such as Chavas, Reed & Knaff (2017).

5) Confusion about whether FGN is new or not

The presentation of FGN reads as though it is being introduced “from scratch” here, which I found potentially misleading given the existence of the Alet et al. (2025) preprint, which predates this submission. I would recommend being explicit about provenance and clearly stating what is new in this paper versus inherited from earlier FGN work. The novelty of the TC-specific learning setup and evaluation appears strong enough on its own; clarifying lineage would by no means take away from this manuscript’s novelty.

6) “Earlification” is slightly obtuse

Since many headline results rely on earlified runs, it would be helpful to have more context/explanations than what is already provided in Section 14: For instance, a schematic showing how TCVitals enters the pipeline and what is modified (track relocation, intensity/size/structure adjustment, etc.). More broadly, I could not easily find prior use of the term “earlification” in the TC forecasting literature, so as written it reads like an informal or new label for a family of established practices; I would strongly encourage anchoring it to the existing terminology and literature if possible.

II) Some minor comments

(i) The authors named their algorithm “CycloneCast”: what about extratropical cyclones?

(ii) Section 2.2: more comments on how the Gaussian-blob technique (e.g., Law and Deng, 2018) addresses the fundamental issue of data imbalance in tropical meteorology would be helpful.

(iii) L895: table labeled as 12.2, but points to Table 1 on p. 29.

References

Alet, F., Price, I., El-Kadi, A., Masters, D., Markou, S., Andersson, T. R., ... & Battaglia, P. (2025). Skillful joint probabilistic weather forecasting from marginals. arXiv preprint arXiv:2506.10772.

Chavas, D. R., Reed, K. A., & Knaff, J. A. (2017). Physical understanding of the tropical cyclone wind-pressure relationship. Nature communications, 8(1), 1360.

Ferro, C. A. T. (2014). Fair scores for ensemble forecasts. Quarterly Journal of the Royal Meteorological Society, 140(683), 1917-1923.

Leutbecher, M. (2019). Ensemble size: How suboptimal is less than infinity?. Quarterly Journal of the Royal Meteorological Society, 145, 107-128.

(Remarks on code availability)

Referee #4

(Remarks to the Author)
I co-reviewed this manuscript with one of the reviewers who provided the listed reports.

(Remarks on code availability)

Referee #5

(Remarks to the Author)
Please see attached review.

(Remarks on code availability)
The code provided with this manuscript is well documented and structured. It is not the end-to-end CycloneCast model, but one could conceivably use the code provided along with the well-documented codebase already publicly available on the GraphCast github page (link provided in comments) to train and run a version of CycloneCast. Therefore, I think the provided code is sufficient.

Version 1:

Reviewer comments:

Referee #1

(Remarks to the Author)
Thank you for your thorough and well-supported responses. I'm satisfied that my concerns have been mostly addressed.

(Remarks on code availability)

Referee #2

(Remarks to the Author)
I co-reviewed this manuscript with one of the reviewers who provided the listed reports.

(Remarks on code availability)
I am pleased with what the authors plan to do. I hope the full release will happen soon.

Referee #3

(Remarks to the Author)
The authors have thoroughly responded to all reviews and addressed most comments in the manuscript. Given the manuscript's expected impact, most of my comments are minor and aimed at making the work's description more specific or addressing leftover review points.

Major comments

Throughout the text: The change in the manuscript's outline has made many references confusing and/or obsolete, with many "Sections" actually referring to the SI (e.g., L188), references to figure panels that have moved (e.g., L1365), etc. To avoid general confusion, I highly recommend going through each reference and preceding it with the appropriate "Methods" or "SI" qualifier. This is major because some figures now seem to be missing.

Figure 2 caption: "Performance is evaluated only when all models make a prediction and the true system is classified as a hurricane, tropical storm, subtropical storm, tropical depression, and subtropical depression": While I understand that this choice was made for consistency with the NHC evaluation guidelines, it will result in a very incomplete picture of a prediction system, highly favoring "cautious" systems with many misses but excellent forecasts for the few systems they predict. Would it be possible to complement this with, e.g., each prediction system's hit rate?

R3 Comment 18 & L129-130 ("we express uncertainty in functional space, an approach we call functional generative networks…") & L608 ("we propose functional generative networks…"): This is one of the reviews that I do not think the authors have properly addressed. The manuscript still makes it seem like it is the first to introduce FGN chronologically.

Where exactly did the authors “clarify the text to avoid the impression that FGN is introduced here for the first time”?

Minor comments

L13: “WeatherNext Cyclones enables up to 1000 member ensembles which better capture…”: On which infrastructure can we expect up to 1000-member ensembles? Just adding “on a XYZ TPU” would be enough to avoid misleading most readers who do not have access to such resources.

L48: “However, these approaches have not achieved state-of-the-art results”: I would be more specific here and complete the sentence: “state-of-the-art results in”, e.g., “combined track and intensity medium-range forecasting” or “real-time tropical cyclone forecasting”.

L80: Because the subsequent formulae use discrete, dimensionless time, it would be helpful to remind the reader that the time coordinate has been normalized using a 6-hour timestep.

L86: “initial atmospheric variables X^0, X^{-1}…”: From IFS initial conditions?

L97-98: “By contrast, a two-step model that 1) predicts X alone, and then … would not have this transfer learning benefit”: Here, one could cite examples that post-process AI weather prediction models, e.g., Bremnes et al. (2024; https://doi.org/10.5194/npg-31-247-2024) or Gomez et al. (2026; https://doi.org/10.1175/AIES-D-25-0073.1) for a tropical cyclone example. I imagine this statement is implicitly justified by the comparison with GenCast in the manuscript? If so, being a bit more explicit could be helpful here.

L137: “making it 8x faster”: I imagine this is “at inference”?

L1170: “with some minimum probability”: Would it be possible to give a number here? Is it an important hyperparameter in the context of WN-C?

R3 Comment 11: I acknowledge the authors’ mention that “tropical cyclone forecasting remains a challenging and fertile field for AI research”, but this does not address the original review, which mentioned the threat to future research in tropical meteorology, much of which does not heavily rely on AI, or does not rely on it at all. Addressing this would not take away from the impressive achievements described in the manuscript.

Very minor comments

R3 Comment 20: Renaming the model “WeatherNext Cyclones” still does not tackle the problem of extratropical cyclones. A quick mention of whether this will be included in the future, or whether it is clearly out of scope and would require a completely different model, would be helpful to anticipate future research.

L1356: “Skill in forecasting these derived quantities relies on correctly modeling the dependencies of their constituent predicted fields”: This is not the most direct way to evaluate these statistically, compared with directly evaluating the dependence structure/copulae. It could be clarified that this is because these derived quantities are physically important quantities.

(Remarks on code availability)
The authors provided part of their code:
- The FGN Predictor class
- Utilities related to tracking, grid handling, and data handling
- A demo colab notebook

I was able to run the demo notebook on Colab without any hurdles, which demonstrates that the corresponding utilities work. Note that it does not include:

- The model's architecture
- The model's weights and biases

which would be important for reproducibility.

Referee #5

(Remarks to the Author)
Please see attached review.

(Remarks on code availability)
I reviewed the code in the first round and found it to be sufficient.

Open Access This Peer Review File is licensed under a Creative Commons Attribution 4.0 International License, which permits use, sharing, adaptation, distribution and reproduction in any medium or format, as long as you give appropriate credit to the original author(s) and the source, provide a link to the Creative Commons license, and indicate if changes were made.
In cases where reviewers are anonymous, credit should be given to 'Anonymous Referee' and the source.
The images or other third party material in this Peer Review File are included in the article's Creative Commons license, unless indicated otherwise in a credit line to the material. If material is not included in the article's Creative Commons license and your intended use is not permitted by statutory regulation or exceeds the permitted use, you will need to obtain permission directly from the copyright holder.
To view a copy of this license, visit https://creativecommons.org/licenses/by/4.0/

Overall comments

The manuscript introduces **CycloneCast**, an AI-based operational ensemble system for tropical cyclones that produces probabilistic forecasts of **track, intensity, and structure** globally out to 15 days. The work is timely and potentially high-impact, and the focus on operational ensemble guidance and scalable large ensembles is valuable. The reported improvements over leading operational baselines (track vs ECMWF ENS; intensity/structure vs HAFS, GenCast; operational consensus benefits) could represent a meaningful advance. That said, I have the following major concerns:

1. The manuscript is currently **hard to assess** because the **writing and structure are poor**. The methods are scattered across the main text and Methods/Appendix, which makes it difficult to understand the exact problem formulation, training objective, uncertainty mechanism, and evaluation protocol.I strongly suggest reorganizing: move the **problem formulation (currently Sec. 2)** and the **evaluation methodology (currently Sec. 3)** into the Methods, then keep the main text focused on the conceptual contributions + key results.

2. Conceptually, the manuscript proposes an ensemble-forecasting paradigm that is distinct from the now-common state-space generative approach (e.g., diffusion-style sampling). Rather than learning structured stochastic perturbations directly in the evolving atmospheric state, CycloneCast primarily generates ensemble diversity through parameter-space stochasticity (noise injected into model parameters or conditioning mechanisms, via FGN) and deep ensembles, trained using a marginal (univariate) fCRPS objective aggregated over many variables, locations, and initialization cases. This design is potentially attractive for operations—especially in terms of sampling efficiency—but it raises a central scientific question: **if the loss effectively assigns a largely uniform weighting across variables and grid points, it is not clear that minimizing marginal fCRPS will produce ensembles with physically correct spatial and multivariate structure of uncertainty. In particular, the system could be well-calibrated pointwise yet still distort circulation-relevant structure (e.g., scale-dependent variance and spectra, multivariate covariance/teleconnections, balance relationships), leading to artifacts such as degraded power spectra or incoherent uncertainty patterns**. Closely related is the concern that parameter-noise–driven ensembles may be more prone to manifold drift in longer rollouts, gradually departing from dynamically plausible climate states even if short-lead marginal scores are good. If the authors wish to frame this as a credible

alternative to generative state-space ensembles (and to position performance relative to baselines such as GenCast, which may benefit from additional techniques like classifier-free guidance), the paper would be much stronger with targeted diagnostics showing that uncertainty is not only marginally calibrated but also structurally realistic and stable (e.g., scale-dependent power/variance spectra, multivariate correlation/covariance structure, balance constraints, and long-rollout stability). Finally, given that the large-scale state X may already be predicted well and cyclone attributes C do not feed back into X, the manuscript should also clarify why joint training of X and C is necessary rather than a strong X-forecast model followed by a separate cyclone downscaling step.

3. The manuscript's "paradigm shift" framing feels overstated, because, as currently formulated, the cyclone component is one-way: **the predicted cyclone attributes (track, intensity, radii) are produced as a downscaling/diagnostic output conditioned on the large-scale state X, but they do not feed back to influence the subsequent evolution of X**. Without a two-way coupling, CycloneCast is best interpreted as a global field forecasting system augmented by a cyclone-aware diagnostic/downscaling module, rather than a fully coupled multiscale model in which cyclone-scale evolution can modify the large-scale circulation. This distinction matters for how the results should be claimed: the approach can still be highly valuable operationally, but the text should avoid implying that cyclone structure is being dynamically resolved within the global circulation model or that the system constitutes an end-to-end multiscale modeling breakthrough. In the same vein, if the base model already predicts X strongly, the paper should more clearly justify why joint training of X and cyclone variables C is necessary beyond convenience, given that C does not influence X in the forecast loop.

4. The work does not include true data assimilation, limiting end-to-end positioning. **The system relies on ECMWF analyses for initialization and introduces "earlification", but it does not perform data assimilation in the standard sense (i.e., conditioning on raw observations with an explicit DA procedure)**. This limits claims that CycloneCast represents a great leap forward toward end-to-end forecast-and-assimilation systems. I suggest reframing: CycloneCast is an operationally efficient AI ensemble forecast model initialized from analyses, rather than a final solution to this problem.

Detailed comments

Abstract

- **L5–6 ("historical database of cyclones")**: Please specify the exact source in the abstract (e.g., IBTrACS) and cite it. "Historical database" is too vague for a core dataset.

- **L7** Is the model driven by real-time updated initial state? Please clarify:
    - Are forecasts updated in real time with each synoptic cycle?
    - What is the update frequency (e.g., every 6 hours)?
    - Is the system initialized from ECMWF analyses operationally, and how does that relate to “timely” forecasts?

- **L11 (“consensus ensemble”)**: Please define what you mean by “consensus ensemble” (e.g., TVCN/IVCN-type consensus, weighted average of models, etc.). Many readers will not know this term. This is defined very late in the Method section, making it hard to interpret in sequential reading.

Section 1 (Introduction)

- **L29 (“trade-off of what?”)**: The manuscript says there is a “trade-off,” but it is not clear what two quantities are being traded off. In operational TC forecasting, the key trade-off is often **resolution vs ensemble size (and cost)**. The text currently reads as if there is a trade-off between large-scale circulation and fine-scale thermodynamic processes, but those are both needed and not a trade-off in itself. Please rewrite this passage to state the actual operational constraint and trade-off clearly.

- **L34 (resolution comparison)**: Please ensure the resolution statements are accurate. ECMWF-ENS is ~9 km, which is finer than the resolution implied by training primarily on ERA5 (0.25°, ~25–28 km). The manuscript should be explicit about:
    - the resolution of X used in training/inference,
    - the resolution of each baseline,
    - how resolution differences might impact intensity/structure skill.

- **L42 (“standard” vs “primitive”)**: Replace “standard” with **“primitive”** (or define what “standard” means). These are “standard” only in the sense that they are the primitive variables of atmospheric dynamics.

- **L44 (missing related literature)**: Please include more emerging literature in the introduction, especially end-to-end/DA-related ML systems:
    - *FuXi Weather*: https://www.nature.com/articles/s41467-025-62024-1
    - Also relevant: generative assimilation and prediction ideas (GAP): https://arxiv.org/abs/2503.03038

- **L54 (how ensemble members are generated here?)**: You mention uncertainty sources (initial state uncertainty, model uncertainty, stochasticity). Please state concretely, in the introduction:

- how CycloneCast generates ensemble members;
- whether variability comes from parameter noise only, deep ensembles only, or both;
- whether any initial condition perturbation is performed (or if not, how initial uncertainty is represented).

- **L58 (cyclone dataset curation and trustworthiness)**: Please provide references and concise description of:

  - which cyclone dataset is used,
  - how it is curated/quality-controlled,
  - uncertainties in wind radii and intensity estimates. This is critical because cyclone structure targets are non-trivial observational products.

- **L53–60 (disconnect with limitations)**: The introduction motivates difficulty from inability to resolve cyclone inner-core structure in global models. However, CycloneCast is still trained on coarse global analyses for X. The writing gives the impression that the model "learns resolved fine-scale structure," but it appears instead to learn cyclone *parameters* conditioned on coarse (X). Please rewrite to avoid implying that the model resolves fine-scale structure in a dynamical sense.

- **L60 ("fine-scale core dynamics")**: I suggest revising to "predicts cyclone intensity/structure parameters" rather than "simulates fine-scale core dynamics."

- **L69 ("best …")**: Please clarify what is meant by "most skillful":

  - most skillful for cyclones or for global fields?
  - relative to which baseline(s)?
  - up to what lead times and in what basins? Avoid broad "best overall" language unless the scope is precisely defined.

Figure 1 and Caption

- **Figure (green arrow)**: Please clarify whether the green arrow represents the **atmospheric state** or the **evolution/rollout** of atmospheric state.
- **Caption grammar**: "**A CycloneCast rollout**" (not "An").
- **Caption structure**: Put the context sentence earlier: "Rollout initialized on Monday Oct 7 … Hurricane Milton … all times UTC" should appear at the beginning.
- **Key confusion**: The sentence "at each forecast step… predicts … 6 h ahead" reads like a 6-hour lead-time forecast only. Please rephrase explicitly:

  - CycloneCast produces iterative 6-hour steps out to 15 days,
  - each step predicts the next 6-hour increment, fed autoregressively.

Section 2

Section title / flow

- **L72 title ("dense and sparse predictions")**: This title is not very clear to the meteorology community. Consider renaming to emphasize joint prediction of gridded fields and cyclone attributes.

- **L74–87 (loose structure)**: The introduction already established key facts. Here you can be more compact and formal: define the task directly as joint probabilistic learning of $p\Big(\{X, C\}^{1:T} \mid X^{\leq 0}\Big)$

- **L89**: "prior state" → "prior states".

- **L90–92 ("standard practice … do not condition on raw observations")**: This is misleading. Operational weather forecasting *does* condition on observations through data assimilation. A more accurate statement is: "forecasts are initialized from analysis states produced by a DA system," rather than implying forecasts do not condition on observations.

- **L95–96 (independence of C_t from C_{t-1})**: As written, it seems C_t depends on $X_{t-2:t-1}$ but not explicitly on C_{t-1}. This could hurt temporal continuity of cyclone attributes. In meteorology this resembles **statistical downscaling/diagnostic modeling**. Please:

    - explain why this conditional independence is assumed,
    - discuss limitations (possible temporal inconsistency),
    - provide evidence that continuity is adequate.

- **L105 ("trained … up to 15 days … 6-hour timestep")**: Please clarify training exactly:

    - Is the training loss computed only at 6h lead time (one-step), or on multi-step rollouts?
    - If multi-step, how is loss aggregated across lead times (uniform average vs weighted)?
    - Your later statements about averaging rollout loss need to be clearly tied back here.

- **L110–115 (coarse analysis limitation)**: You criticize the two-step approach because coarse fields underestimate intensity. But CycloneCast also uses coarse analysis fields for (X). How does CycloneCast actually address this? The current text states the limitation but does not clearly show how your design resolves it.

- **L126 (“centered at a given grid point”)**: More precise wording: a cyclone center can be “within a grid cell,” not “centered at a grid point.”
- **L128 (“12 quadrant-wise wind radii”)**: Please explicitly state 3 thresholds × 4 quadrants = 12.
- **L143–149 (continuity / failure cases)**: Since cyclone information is extracted via existence probability + tracking, please discuss:
  - how temporal continuity is ensured,
  - whether discontinuities occur (e.g., blob splitting/merging, sudden jumps),
  - whether there are failure cases where track/existence becomes inconsistent.

Section 2.3 (Uncertainty / FGN)

- **L154–162 (missing uncertainty source)**: You mention epistemic and initial-state uncertainty. But a third major uncertainty source is **stochasticity** due to coarse representation and unresolved processes, plus chaotic growth with time. Please discuss this explicitly and relate to superensemble ideas (e.g., Krishnamurti, T. N., Chandra M. Kishtawal, Timothy E. LaRow, David R. Bachiochi, Zhan Zhang, C. Eric Williford, Sulochana Gadgil, and Sajani Surendran. "Improved weather and seasonal climate forecasts from multimodel superensemble." Science 285, no. 5433 (1999): 1548-1550., as well as **GenEPS**: https://www.nature.com/articles/s41612-025-01255-x).
- **L163–170 (initial state uncertainty)**: I cannot see how FGN captures or reduces initial-state estimation uncertainty. If the model is conditioned on an analysis state without explicit perturbation of initial states, please clarify what “initial condition uncertainty” means in this context and how it is represented.
- **L166 (distribution over parameters)**: What distribution is used to perturb neural network parameters? Gaussian? How is it parameterized (global noise vector, conditional layer norm, low-rank covariance)? Please provide:
  - explicit definition,
  - rationale for this choice,
  - and any theoretical/empirical support.
- **L167 (marginal fCRPS in high-dimensional fields)**: fCRPS is proper for **univariate** distributions, but you predict a high-dimensional multivariate field. Please explain:
  - how you weight the CRPS losses across initial cases, variables, locations, and levels,
  - whether all variables/locations are equally weighted (your description suggests

they are),
    - and why this does not harm spatial structure (spectra, coherence).

- **L174 ("loss averaged over rollout steps")**: You average fCRPS across rollout steps. Please justify why uniform averaging across lead times is appropriate. Would a decaying factor be more reasonable, especially with only N=2 members per batch during training?

- **L181 (GenCast needs 40 runs)**: Please add a brief explanation for readers unfamiliar with diffusion models: why does GenCast require ~40 denoiser calls per forecast step?

- **L182 (8× faster to sample)**: Please specify the hardware / compute setting for this claim (e.g., GPU type, batch size).

- **L183 ("sparse target")**: Please define "sparse targets" explicitly (cyclone targets only present near cyclone centers).

- **L184–185 (difficulty following the point)**: Please clarify:

    - Why do autoencoders/diffusion frameworks "struggle" with sparse targets in your view?
    - Since cyclone variables do not feed back into (X), one could forecast (X) with a strong generative ensemble model and then run a cyclone diagnostic model afterward. Why is end-to-end joint prediction necessary here, beyond convenience?

Section 3 (Evaluation methodology)

- **L187–192 (paired/unpaired naming)**: The idea of separating formed vs not-yet-formed cyclones is valuable, but I suggest:

    - introducing cyclone genesis as a major challenge earlier in the Introduction,
    - clarifying why these are called "paired" and "unpaired" (not standard terminology).

- **L201 ("performed similarly")**: This is ambiguous. "Performed similarly" should specify:

    - which metric(s),
    - which variable(s),
    - which lead times,
    - and which basins/regions/resolutions.

- **L204–207 (HAFS reference)**: Please add references/citations for the exact HAFS configuration (HAFS-A) and explain the evaluation domain limitation.

- **L208–210 (bias correction for baseline GenCast)**: Why apply a bias correction to the baseline model rather than evaluating raw performance? Please justify clearly and ideally present results with and without the correction.

- **L211 (late models depend on analyses)**: Not all recent data-driven forecasting models depend exclusively on analyses; some incorporate DA or observation-to-forecast pipelines. Please update this statement and cite:

    - FuXi Weather (Nat Commun 2025): https://www.nature.com/articles/s41467-025-62024-1
    - GAP (arXiv 2503.03038): https://arxiv.org/abs/2503.03038

- **L217–218 (earlification placement)**: Earlification appears central for operational evaluation and related to initialization error. It should be described earlier (main text and Methods), not only later, and framed explicitly as a strategy for operational latency.

- **L235–237 (confusing training/evaluation description)**: I am confused:

    - what is the benefit of retraining/finetuning each year,
    - whether you evaluate a given model on the next year only,
    - and what "two years combined" refers to exactly.

- **L247 (Appendix B.1 insufficient)**: Section B.1 does not provide enough detail for the evaluation strategy and significance testing. Please summarize the main statistical testing in the Methods.

- **L249 ("always provide the best guidance")**: This is too strong. Please be specific: do you mean consensus means often outperform any single model on mean track error? Also, CycloneCast is not strictly a single model (it is a deep ensemble). Please clarify terminology.

Section 4 (Global weather forecasting)

- **L258–263**: The text discusses comparison to ENS but the presentation seems uneven (sometimes only GenCast comparisons are highlighted). If ENS comparisons are central, they should be summarized clearly in the main text.
- Many results are pushed to Appendix B.2; operational readers would benefit from a **summary table/figure** in the main text.

Section 5 (Track)

- **Overlap**: The content in L286–287 overlaps with L297–305. The section can be made

much more compact and clear:

  - first present deterministic ensemble-mean track error,
  - then probabilistic score,
  - then calibration (spread/skill), without repeating similar statements.

Section 6 (Intensity and structure)

- **L311**: CycloneCast does not "resolve" cyclone structure dynamically; it is more a statistical downscaling/diagnostic model for cyclone parameters. Please adjust wording.

- **L313–318 (fairness)**: It is not fair to compare CycloneCast ensemble mean directly against a single deterministic NWP member. Please include:

  - comparison against individual CycloneCast members, or
  - compare ensemble means consistently (e.g., against ENS mean), and explain why ensemble-mean comparison is operationally appropriate.

Rapid intensification (RI)

- Your RI evaluation is interesting, but please ensure definitions and interpretation are clear for readers:
  - define RI early (the threshold),
  - explain how ensemble probabilities map to decision thresholds,
  - and present reliability in a straightforward way.

Section 8 (Operational guidance / consensus)

- "Consensus ensemble" should be defined earlier (not first appearing late). Many readers will not know TVCN/IVCN.
- Please clarify what exactly is optimized (weights), on what data (validation), and whether weights vary with lead time or basin.

Section 9 (Conclusion)

- **L432 ("overcome")**: "Overcome" is too strong given:

  - no true assimilation,
  - cyclone variables are largely downscaled,
  - and physical coupling is limited. Please soften this language.

- **L446 (“double-penalty problem”)**: Please briefly explain what this means for non-specialists.
- **L447 (“co-training”)**: If cyclone variables do not influence (X), the manuscript should explain why joint co-training is necessary rather than training separate modules (global forecast + cyclone downscaler).

Methods Section (Specific)

- **Layout**: I find the layout confusing. I recommend moving the current Sec. 2 and Sec. 3 content into the Methods, then clearly presenting: 1) learning task definition,
  2) cyclone downscaling/griddification + tracker,
  3) FGN uncertainty model + deep ensembles,
  4) training objective/weighting and rollout strategy,
  5) evaluation + statistical tools.
- **L665–666**: Please avoid vague phrases like “some wind speed probabilities evaluation.” Be specific: what evaluations, what source, what quantitative basis?
- **L672**: Clarify that this statement applies to the cyclone-variable loss/training, not the large-scale atmospheric field prediction model (FGN).
- **L730 (threshold = 0.3)**: Please justify why 0.3 is chosen (sensitivity test or tuning procedure).
- **L780 (J=4 deep ensemble)**: Please provide an ablation: what happens with fewer than 4 models? Since you call this a trade-off, show that it is a reasonable one.
- **L789 / L803–807 (form of (\mathcal{P}_{\mathcal{M}}(\theta)))**: You later define a Gaussian-like parameterization. Please explain:
  - why this choice,
  - what assumptions it encodes,
  - and whether alternatives were tested.
- **L800 (spatially-dependent perturbations)**: Please clarify what you mean and why they cause high-frequency components that “need to be tamed.”
- **L827–829 (parameter noise vs input noise)**: This is an important discussion point. Generative state-space perturbations (input noise) often preserve structure and spectra better. Please discuss explicitly:
  - what structural advantages you gain/lose,

    - and provide diagnostics (spectra, long-term rollout behavior).

- **Sec. 13 (baseline modification)**: I question whether it is necessary to modify the baseline in this paper. If you keep it, please show results both with and without the modification.

- **L942–943 (define consensus ensemble earlier)**: Please define “consensus ensemble” early in main text.

Recommendation

The contribution is potentially important, but the manuscript needs substantial improvement in (i) clarity and organization, (ii) careful positioning and toned-down claims, and (iii) deeper justification/diagnostics for the chosen uncertainty modeling and the (currently one-way) coupling between cyclone parameters and atmospheric state.

I co-reviewed this manuscript with one of the reviewers. The main contents of my review are included in one of the referee reports. Here are some concerns of my own that I want the authors to answer.

L8: “Structure” is not appropriate. Tropical cyclone structure includes vertical structure and horizontal structure. “Wind circle radius” is a precise word. Also, in L23 and so on.
L29: trade-off is not appropriate; dilemma is more precise.
L120: what is stitching algorithm?
L130-134: what are blobs? I do not quite follow what you want to express. The "standard technique" for computer science may not be comprehensible for readers in the other disciplines.
L135-142: In L125-L129 "A set of grids where each grid represents the value of a cyclone property (e.g. maximum wind speed) if a cyclone were centered at a given grid point." I don't quite understand why you define your target within a circular region of radius 120 km, since you define the cyclone property only on the grid point of cyclone center. I know you explain it in the appendix, but reader might get confused here.
L143-152: How does blob-tracking algorithm work. Why cyclone properties are extracted by bilinear interpolation on scalar fields. You should make the reader more comfortable. Not all readers want to refer to the appendix for details. You should make the readers understand what you are talking about by making a brief introduction of your whole idea here.
L166: Why the neural network weight is sampled from a learned distribution, how does it work? explain it with more details.
L211-229: This paragraph is not necessary. I think it can be thrown to the appendix.
L237: "For each calendar year of evaluation, the model was retrained (with the frozen protocol) on data up to the end of the preceding calendar year." Why?
L308-311: These lines appear here again. These are almost identical to L107-L115, no need.
L339: operational meteorology is not appropriate; TC dynamics is precise.
L358: You may consider a sub title around L358.

Review of "Tropical cyclone forecasting with AI"

This manuscript describes the new CycloneCast AI forecasting model for tropical cyclones. CycloneCast is trained on a combination of global weather analysis data and a database of tropical cyclones (IBTRaCS). It predicts the track, intensity, and basic wind structural metrics of TCs globally. The model is evaluated for the years 2023 and 2024 and is shown to represent a step increase in skill at forecasting these critical metrics compared to traditional NWP models. Moreover, including CycloneCast in the track and intensity consensus ensembles used operationally at NHC greatly improves their skill. Thus, this work represents a novel and urgent contribution to the TC forecasting literature and its impact is certainly appropriate for Nature.

I have one general comment regarding the lack of verification results for the radius of maximum wind (RMW), and several other comments aimed at clarifying the presentation of the results and the explanation of the technique. All of these comments should be straightforward to address, and so I recommend accepting the manuscript for publication after minor revisions.

Recommendation: Minor Revisions

General Comment:

Section 6 does not contain a discussion of the verification results for the radius of maximum winds (RMW). The RMW is an important wind radius for anticipating storm surge impacts from TCs (e.g., Irish et al. 2007, https://doi.org/10.1175/2008JPO3727.1) and the area impacted by the most extreme winds in the eyewall at landfall. Even high-resolution NWP models like HAFS struggle to accurately predict the RMW (Trabing et al. 2024, https://doi.org/10.1029/2024GL109663) and so AI models like CycloneCast offer great promise for addressing this forecasting limitation. That said, the quality of RMW data in IBTRaCS is generally lower than that of other metrics like intensity and track – especially outside of the Atlantic basin and prior to 2021 (when NHC first started post-season quality control of RMW in the Atlantic; see Knaff et al. 2021, https://doi.org/10.1016/j.tcrr.2021.09.002). Because most of the training years for CycloneCast are before 2021, the model was likely trained on RMW estimates of questionable quality. However, the IBTRaCS RMW for Atlantic TCs in the verification years (2023 and 2024) is probably more reliable and could be used for verification. Therefore, I'd still like to see a few sentences added to the manuscript (possible in Section 6) about RMW verification results, or the reasons why such a verification was not conducted.

Minor/Specific Comments:

Lines 5-6: Given the title of the paper and the focus of IBTrACS on storms originating in or near the tropics, I recommend including the word “tropical” before “cyclones” here and elsewhere in the abstract. This avoids giving the impression that CycloneCast is also capable of predicting TC-specific metrics for mid- and high-latitude cyclones (which have different structural characteristics from TCs).

Line 51: Please change “NWPs” to “NWP models” to align with conventional terminology.

Line 60: I believe by “core dynamics” you mean “inner-core dynamics”. Also, the reference to Figure 1 on this line is not necessary and somewhat out of place here (see next comment).

Lines 63-64: It is unusual (at least in the meteorological literature) to reference a figure this early in a paper. It’s fine to tease these statistics in these introductory paragraphs, but I recommend reserving specific references to Figure 2 for Sections 5 and 6. I also recommend moving the positions of Figures 1 and 2 closer to Sections 5 and 6 where they are discussed in more detail.

Figure 1a: You might consider showing the ensemble mean forecast metrics (track and wind radii) instead of values from an individual ensemble member. The ensemble mean tends to have more skill than any one individual member, and showing the ensemble mean avoids having to justify why this particular member was selected for illustration.

Figure 1a: I recommend including the best track of Milton on all four panels so the reader has a better sense of how good the ~66-h landfall forecast is.

Figure 1 caption: A 24-hour time format is used when referring to UTC times, so I believe it should be 1200 UTC (instead of 12am UTC). However, it is unclear if you actually mean 0000 UTC, assuming 12am indeed refers to midnight. Please correct.

Figure 1 caption: Change “An CylconeCast rollout” to “A CycloneCast rollout”.

Figure 2 caption: “Tropical storm” should also be included in the list “…a hurricane, a tropical depression, or a subtropical depression”. It’s also unclear why subtropical depressions are included in the verification.

Figure 2: Please clarify somewhere in the caption that the yellow line represents the ENS *ensemble mean*.

Figure 2 caption: Please note in the caption that GenCast does not appear in panel (c) because it does not predict wind field radii.

Figure 2 caption: Please note in the caption why the sample counts in (c) are lower than in the other panels. I recognize it's because the number of storms with 64-kt wind radii is a subset of the total number of storms used for verification, but it would help to clarify that point.

Line 158: The use of "ensemble" as a verb is unusual; I recommend revising to something like "...combine their predictions into an ensemble."

Line 161: Change "they grow" to "it grows"

Line 201: When you say "It" here, I assume you are referring to the ENS ensemble mean, but that should be specified.

Line 207: This is the first time the reader is seeing "HWRF" and "HMON". It would be worth spelling out these acronyms and briefly explaining that these are the legacy deterministic TC forecasting models used operationally.

Line 231: I would not classify IBTrACS as a reanalysis; I recommend replacing "reanalysis" with simply "dataset" to avoid confusion.

Line 289: I recommend just citing Fig. 1d in this sentence to make it clear that you are referring to the narrow region of Hurricane-force wind probabilities; the tropical storm-force wind probabilities encompass the entire state of FL and, as such, do not obviously reflect a tightly clustered ensemble. If it's possible to include all the ensemble cyclone tracks in Fig 1, that would provide clearer evidence of a tightly clustered ensemble near the landfall position.

Line 291: The end of the sentence ending on this line is awkward; perhaps a better way to say this is "...a generalisation of CRPS that is better suited for positions than scalars."

Lines 304-305: This last sentence is confusing because the previous sentence states that the calibration of CycloneCast is similar to that of ENS, while this sentence implies that the calibration of CycloneCast is better than ENS. Please rewrite or remove this sentence.

Lines 324-325: CRPS does not need to be spelled out again here.

Lines 340-341: I do not think this statement is correct; RI predictions can be (and are) evaluated at any forecast lead time, not just within the first 24 h. Please clarify and/or adjust this sentence.

Lines 342-343 and Fig. 3e: The "recall-precision" terminology used here is non-traditional in the atmospheric sciences. I believe the equivalent (and more commonly used) terms for recall and precision are Probability of Detection (POD) and Success Rate, respectively. See Roebber (2009, https://doi.org/10.1175/2008WAF2222159.1).

Figure 3e: The different blue stars in this panel are not adequately explained in the text or caption. I assume they represent different required thresholds on the number of ensemble members predicting RI to be considered an RI prediction. If that is indeed the case, then as a matter of presentation clarity I recommend using fewer stars (thresholds) and labelling them accordingly in Fig. 3e so the reader knows which stars in the diagram correspond to the strictest vs. most lenient RI classifications from CycloneCast.

Figure 3 caption: The caption should explicitly state that the shading in panel (e) is the critical success ratio, as that is not clear from the text.

Lines 346-348: It's difficult to see how CycloneCast improves upon the deterministic high-resolution NWP models at RI prediction based on Fig. 3e. I assume it's because most of the blue stars (including many with the highest thresholds of members predicting RI) are in regions of the diagram corresponding to higher CSI values than the deterministic models, but that should be stated more clearly in the text.

Line 396: Please expand the last part of this sentence for added clarity, e.g., "…jumps from 0.5 in the 50-member ensemble to 0.7 in the 1000-member ensemble."

Figures 5a,b: The gray and blue lines in these panels are difficult to differentiate. I recommend re-creating these panels using more contrasting colors (grays and reds, for example).

Figure 5 caption: In the last sentence of the caption, it's more accurate to say that IVCN+CycloneCast outperforms IVCN across all lead times up to 5 days (not 6 days).

Lines 864-867: Please clarify in this description which datasets are used during the overlap years of 2016-2018.

Line 904: Remove repeated instance of "number of" on this line.

Line 919: Please include a citation for the Tempest Extremes tool, e.g., Ullrich et al. (2021, https://doi.org/10.5194/gmd-14-5023-2021).

Line 1019: Please correct grammar issue: "of the of year"

Line 1082: Please change "computing" to "compute".

Figures B11-B13 captions: The last sentence in each of these figure captions has an odd structure; consider revising to: "GenCast predicts track probabilities, but not wind radii, which is why it only appears on the bottom row"

Figure B14 caption: Remove boldface after first sentence.

Lines 1276-1277: This is unclear to me. Do you mean to say the performance of a model at forecasting intensity at the beginning of a TC's lifecycle is consistently related to its future performance for that same TC? If so, that is not obviously apparent to me from Figs. B17-18.

Section B.8: I'd be more curious to know if CycloneCast tracks tend to be better (than HAFS or ENS) at specific intensities. One way to examine this would be to see if there are strong correlations between relative improvements in track and intensity. It also appears from Figs. B17-18 that CycloneCast positions tend to improve the most upon HAFS and ENS positions around the times where there are substantial changes in cyclone heading (e.g., during TC recurvature). This may be worth mentioning briefly in this section.

I co-reviewed this manuscript with one of the reviewers. The main contents of my review are included in one of the referee reports. Here are some concerns of my own that I want the authors to answer.

L28-L30: need reference here.

L37-39: Why NOAA-HAFS's track prediction is not accurate since it is a storm-following model which captures both large-scale background flow and fine-scale TC dynamics? In L59-61 you state WN-C's ensemble mean is significantly more accurate than the specialised HAFS model on intensity and quadrant wind radii, but not on tracks. This is not consistent with the arguments that HAFS's track error is large. You should rephrase the statements here.

L93-98: I think this paragraph should appear in section 2.2, following L115. In equation (1), X's prediction is completely irrelevant to C. I do not see any advantage of jointly predicting X and C in enabling sharing latent representations learned from gridded data X and tabular data C. But in section 2.2, I think the end-to-end forecasting provides extra information on TC tracks and intensity for AI-based forecasting model of gridded data X to learn extra knowledge, which prove the statements here in L93-98.

L122-L126: I do not quite follow what you want to express here. You seem want to argue that CRPS is not a good objective to optimize, but you still use CRPS loss. I don't understand the "marginal" and "joint" here.

L251: You should briefly explain why GenCast should be debiased here.

2nd Review of "Operational tropical cyclone forecasting with AI"

The authors have adequately addressed my main concerns. However, I encountered some new issues in the revised manuscript related to ambiguous references to figures, sections and tables, which may have resulted from the reorganization of the manuscript. I also have some minor clarification and grammatical comments. Otherwise, I believe the manuscript is very close to being suitable for publication.

Figure and Section Reference Issues: There are several references throughout the revised manuscript to sections, tables, or figures that are ambiguous because the authors do not specify if these items are in the main text, the supplementary material, or the extended data sections. I attempted to compile an exhaustive list of problematic references below, but the authors should carefully examine each reference in the manuscript to make sure it is accurate and unambiguously refers to sections/figures in the main vs. supporting/extended text. Generally speaking, references to main text figures/tables/sections do not need qualifiers while references to figures/tables/sections in the supplementary material or extended results do (e.g. Supplementary Section 2.1, Extended Data Figure 3, etc.)

- Lines 145, 153: indicate that these are references to supplementary Section 2.4.
- Line 164: indicate that this is supplementary Section 1.3.
- Line 172: indicate that this is supplementary Section 2.2.
- Line 188: indicate that this is supplementary Section 2.3.
- Line 203: indicate that this is supplementary Figure 1.
- Line 204: indicate that this is supplementary Section 2.9.
- Line 214: This number "4" in parentheses needs a qualifying word before it ("Section 4"? "Figure 4"?)
- Line 221: indicate that this is supplementary Section 1.2.3
- Line 228: indicate that this is supplementary Section 1.2.4
- Line 304: indicate that this is supplementary Section 1.2.5
- Line 306: I believe the figure references on this line are to Extended Data Figure 7 and Supplementary Figures 15, 16, and 17. Please adjust figure references accordingly.
- Line 322: indicate that this is supplementary Section 2.1
- Line 544: indicate that this is supplementary Table 1
- Line 793: indicate that this is supplementary Section 1.3
- Lines 809, Table 2 caption: Please add the word "Section" before "2.2"
- Line 813: This reversed range of figures ("Extended Data Figures 13 to 4") seems incorrect. Please double check the figure numbers/range.

Other comments:

Line 53: IBTrACS should be spelled out the first time it is used.

Line 126: The use of "wind pressure" is unusual. Do you mean a local minimum in "wind and pressure"?

Line 137: I recommend adjusting to something like "…making it 8x faster and substantially easier to model the sparse cyclone targets" to address awkward grammar.

Line 155: Please define HRES-fc0 the first time it appears.

Line 239: Please add the word "a" before "cyclone's"

Fig. 3e/Lines 276-277: this sentence seems like it would be more appropriate in the caption than in the main text.

Fig. 3e caption: The statement about the "off-trend performance on the bottom-right" is helpful for interpreting the different stars, but I still felt a clearer statement is needed here. I think I'm getting hung up on what exactly is meant by "off-trend performance". Perhaps a clearer way of saying this is: "the stars in the lower right (upper left) correspond to higher (lower) probability thresholds for RI."

Line 343: To be consistent with the following sentence, change "and a mean" to "with an average".

Line 360: I recommend rewording to "from June 2025 to present".

Line 535: Change "entires" to "entries"

Line 540: Change "for quadrants" to "four quadrants"

Line 543: Move "IBTrACS-derived" to be before the word "gridded".

Line 689-696: A lot of this material has already been covered elsewhere in the manuscript and can probably be removed or shortened considerably.

Line 703-704, 707: It's unusual to use "ensemble" as a verb like this. I recommend changing to "combine their predictions into an ensemble".

Line 722: Please spell out the acronym "RL" if this is the first time it appears.

Lines 789-790: please be cognizant of how many times this CRPS definition ("a strictly proper scoring rule…") is repeated throughout the manuscript. You probably only need to repeat this statement once or twice to reduce the word count.

1 Common Responses

1.1 Code Availability and Open-Sourcing

This topic was raised by the *editor* and by *Referees #1, #2, #3, and #5*.

We are taking a two-step approach to code availability. For the review process, we now provide the full cyclone data generation and tracker code, along with the already provided code snippets describing the FGN framework, and the public neural network code(`https://github.com/google-deepmind/graphcast`). Upon publication, we will open-source the full inference code and a minimal training pipeline, adhering to the high standard of transparency we set with GraphCast and GenCast. We have also significantly increased the level of detail of the model, tracker, and evaluation in the supplements.

We believe this approach is justified for several reasons:

- **Proven reliability:** Unlike previous AI weather papers evaluated solely on reanalysis data, WN-C has been running live on Google DeepMind WeatherLab and has directly influenced real-time NHC predictions. The revised manuscript includes a 2025 operational season analysis demonstrating this. Furthermore, reviewers can query the deployed model via a public Vertex AI API (`https://developers.google.com/weathernext/guides/access-vmg`) to obtain field predictions for arbitrary inputs and independently verify performance claims.
- **Technical constraints:** The training infrastructure is substantially larger than preceding systems and is TPU-dependent, making fully independent replication by reviewers impractical.
- **Full content:** The core network code is functionally nearly identical to our already open-sourced GenCast model. However, we have an internal re-implementation that we think is more extensible. Open-sourcing it is taking significant amount of time, that we want to parallelise with the reviewing process. However, because it is a re-implementation rather than a new neural network, the novelty for reviewers to assess lies primarily in the cyclone-specific components (data representation and tracker), which we now provide. For this, we have included a demo of the cyclone data gridding process and the operation of the tracker in our response email.
- **Commitment:** Following an internal review process, we commit to open-sourcing both the code and the model weights upon acceptance, to make the work open to the community.

1.2 Model name change

At the time of review, our model was called CycloneCast. In our resubmission, it has been renamed to WeatherNext Cyclones (WN-C), which we use in our responses. This will make it easier to identify its provenance (the FGN model contribution of this work is behind WeatherNext 2), and makes it clearer that this model is both a (state-of-the-art) global weather prediction model and a cyclone model.

1.3 2025 evaluations

Although not explicitly requested by the reviewers, we have included 2025 results to strengthen the operational angle highlighted by the *editor*.

Since IBTrACS is provisional for 2025, this limits the extent of the evaluation that can be conducted at this time. One particular limitation is that the storm classification field in IBTrACS (e.g. tropical storm / depression, subtropical storm / depression, hurricane) is not finalised outside the NHC basins, so we cannot apply the storm stage subsetting protocol that the NHC uses for verification. In addition, the `USA_*` fields of IBTrACS, i.e. the fields are not yet finalised and are less reliable outside the US basins at the time of writing. We have therefore restricted our additional 2025 evaluation to paired deterministic evaluations for Atlantic and East Pacific hurricanes. For this reason, we're keeping this in the appendix rather than modifying the main text. We leave it to the *editor* to decide whether our abstract and introduction should say 2023–2024 or 2023–2025.

1.4 IBTrACS NaN treatment in evaluations

Wind radii in IBTrACS are often NaN. In our first submission we simply skipped them, as is conventional in machine learning. This was the reason wind radii counts were substantially lower than for maximum wind speed and track errors, as pointed out by *Referee #3*. Many, but not all, of the wind radii in IBTrACS reported as NaNs can be imputed to 0, when the maximum wind speed does not exceed the corresponding wind speed threshold. For instance, if wind speed is 54 kt, we know all 34 kt and 50 kt wind radii must be zero. Some forecasters use NaN to report 0, but not all NaNs are due to this convention, e.g. some nans simply denote missing data.

We trained the model with this imputation, but we're undecided on what to it for the evaluation. After extensive deliberation, we believe there are two reasonable ways of doing the evaluation, that we present here.

1. **Unconditional evaluation**: we always evaluate wind radii, regardless of maximum wind speed. We impute as many wind radii as possible to 0, and remove the rest from the evaluation. This gives us higher counts than the first submission, as now a fraction of them are imputed to be 0.

2. **Conditional evaluation**: we only evaluate wind radii when maximum wind speed exceeds the threshold, both for the ground truth and for all models. This evaluates models on the question "What do you expect the x kt wind radii to be if the wind speed is above x kt" The conditioning happens at the ensemble member level for probabilistic models, i.e. only members with wind speeds above the threshold count towards the conditional mean computation.

For the unpaired REV plots our suggested approach is to impute the imputable NaNs to 0 and create a NaN mask to skip evaluation for the non-imputable NaNs. There are no missing track centers, a handful of rare misses in wind speed, and about 1–2% of wind radii missing. For those, in the paired evaluations we simply ignore the time step. For unpaired evaluations we exclude a disc around the track center from the evaluation if the ground truth is missing and not imputable, to avoid incorrectly penalising / rewarding a model if the ground truth is missing.

As shown in fig. 1 the core main message remains, with WN-C outperforming baselines in unpaired and in all except one day for paired. Before seeing evaluation results we decided to switch to conditional evaluation and 34 kt. Doing the conditional evaluation is the same as the NHC protocol, but reduces the number of samples further. Using 34 kt instead of 64 kt increases the number of samples due to the more lenient conditioning and the fact that sometimes 34 kt are reported and 64 kt aren't. However, we show all options here for visibility and in case reviewers think another plot is more suitable.

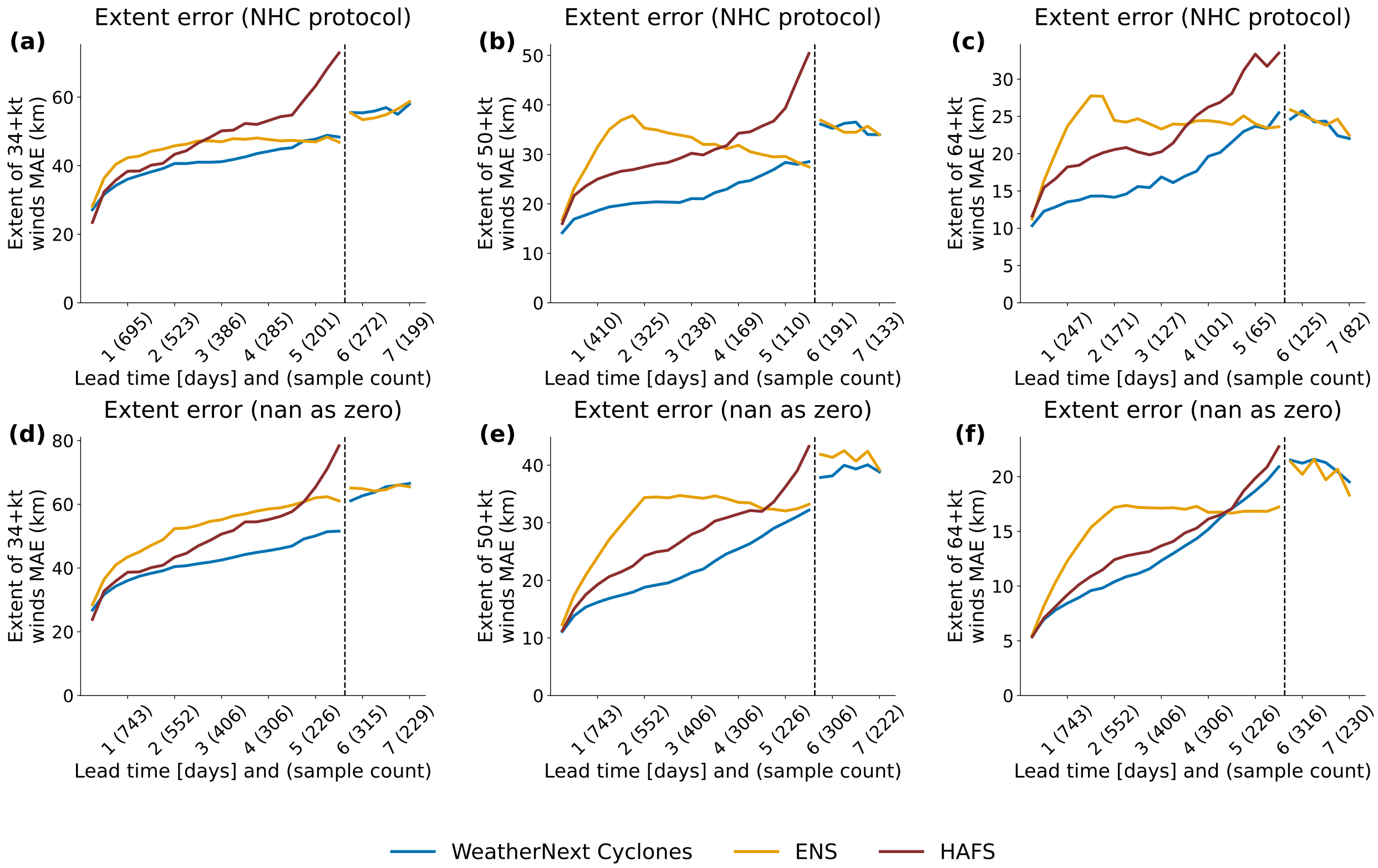

Figure 1: Deterministic evaluation of quadrant radii for all quadrant thresholds across both evaluation protocols for handling NaN quadrants.

3 Response to Referee #1

Note: Referee #1's comments were provided as a PDF attachment.

R1: ***Overall comments***

The manuscript introduces CycloneCast, an AI-based operational ensemble system for tropical cyclones that produces probabilistic forecasts of track, intensity, and structure globally out to 15 days. The work is timely and potentially high-impact, and the focus on operational ensemble guidance and scalable large ensembles is valuable. The reported improvements over leading operational baselines (track vs ECMWF ENS; intensity/structure vs HAFS, GenCast; operational consensus benefits) could represent a meaningful advance. That said, I have the following major concerns:

We thank the reviewer for their careful reading and for recognising our operational ensemble system, WeatherNext Cyclones, as 'timely', 'potentially high-impact', and 'valuable'. We have substantially revised the manuscript to address the concerns raised, focusing on clarity, precise terminology, and providing deeper justifications for our methodological choices.

R1 Comment 1: *The manuscript is currently hard to assess because the writing and structure are poor. The methods are scattered across the main text and Methods/Appendix, which makes it difficult to understand the exact problem formulation, training objective, uncertainty mechanism, and evaluation protocol. I strongly suggest reorganizing: move the problem formulation (currently Sec. 2) and the evaluation methodology (currently Sec. 3) into the Methods, then keep the main text focused on the conceptual contributions + key results.*

We agree with this structural critique of the initial submission. To improve clarity and comply strictly with Nature's 4,500-word limit and formatting guidelines, we have significantly restructured the manuscript. The detailed problem formulation and evaluation methodology have been moved to the Methods section. We have retained only concise, high-level summaries of these concepts in the main text to ensure it remains focused entirely on core results and conceptual contributions. In particular, one of our key algorithmic contributions is the griddification algorithm, allowing the gridded AI weather model to train on sparse, tabular cyclone trajectory data, which is now outlined more clearly in Section 2.2 (but with specific details delegated to the Methods in Section 10.1).

R1 Comment 2: *Conceptually, the manuscript proposes an ensemble-forecasting paradigm that is distinct from the now-common state-space generative approach (e.g., diffusion-style sampling). Rather than learning structured stochastic perturbations directly in the evolving atmospheric state, CycloneCast primarily generates ensemble diversity through parameter-space stochasticity (noise injected into model parameters or conditioning mechanisms, via FGN) and deep ensembles, trained using a marginal (univariate) fCRPS objective aggregated over many variables, locations, and initialization cases. This design is potentially attractive for operations—especially in terms of sampling efficiency—but it raises a central scientific question: if the loss effectively assigns a largely uniform weighting across variables and grid points, it is not clear that minimizing marginal fCRPS will produce ensembles with physically correct spatial and multivariate structure of uncertainty. In particular, the system could be well-calibrated pointwise yet still distort circulation-relevant structure (e.g., scale-dependent variance and spectra, multivariate covariance/teleconnections, balance relationships), leading to artifacts such as degraded power spectra or incoherent uncertainty patterns. Closely related is the concern that parameter-noise–driven ensembles may be more prone to manifold drift in longer rollouts, gradually departing from dynamically plausible climate states even if short-lead marginal scores are good. If the authors wish to frame this as a credible alternative to generative state-space ensembles (and to position performance relative to baselines such as GenCast, which may benefit from additional techniques like classifier free guidance), the paper would be*

much stronger with targeted diagnostics showing that uncertainty is not only marginally calibrated but also structurally realistic and stable (e.g., scale-dependent power/variance spectra, multivariate correlation/covariance structure, balance constraints, and long-rollout stability).
We thank the reviewer for inquiring about how WN-C, trained only on the fCRPS of marginals, performs on the multivariate, joint distributions in the ensemble forecast. In response, we have added several further evaluations to the supplementary material (pointed to from the main text), evaluating various aspect of the joint forecast distribution. We added Figure 6, evaluating (1) average and max pooled versions of the ensemble weather field forecasts versus GenCast, (2) the CRPS for two derived quantities, 10m wind speed and the difference in geopotential at pressure levels 300hPa and 500hPa, versus GenCast (3) the averaged power spectra of a number of variables at different lead times. WN-C consistently outperforms GenCast across spatial pooling scales and derived variables, and its spherical harmonic power spectra closely match those of analysis state data for key variables. We have also added a note about the existence of minor artefacts in the forecasts which constitute "violations" of the true joint spatial structure. However, the key take-home is that spatial structure and correlations across variables are not significantly sacrificed by optimising the CRPS of the marginals.

Of relevance to joint structure in the high-dimensional weather distribution is Figure 14a, which evaluates the position error of a physics-based tracker (TempestExtremes) run on WN-C's atmospheric state rollouts. The position error of the physics-based tracker closely matches that of the AI tracker run on the IBTrACS output channels (referred to as E and S in the revised manuscript). This requires spatiotemporal and inter-variable coherency in the form of persistent mean sea level pressure minima and upper-level warm cores in WN-C's atmospheric state trajectories, further suggesting WN-C captures complex, joint structure in its forecast distribution.

While this is a surprising result, it is outside the remit of this work to conduct a full investigation into *why* training on marginal fCRPS loss is sufficient to generalise reliably to statistics of the joint distribution.

Regarding the stability of long rollouts, while we view this as an important consideration in AI weather model development, the focus of our paper is on medium-range weather forecasting, and so we leave such investigations to future work. We note, however, that we found WN-C to be consistently stable across the 15 day forecast horizon evaluated in our manuscript.

Finally, regarding, classifier-free guidance (CFG), there are indeed many ways one could try to improve GenCast, CFG among them. However, it is an open question whether CFG would actually improve forecast skill, since it trades off distributional coverage/diversity for sample quality, but the former is obviously crucial in a probabilistic forecasting task. Furthermore, the computational efficiency of WN-C's one-step sample generation process (unlike the multi-step diffusion process) is an enabler of larger ensemble sizes (e.g. 1000 members, as evaluted in our paper) and model sizes.

R1 Comment 3: *Finally, given that the large scale state X may already be predicted well and cyclone attributes C do not feed back into X, the manuscript should also clarify why joint training of X and C is necessary rather than a strong X-forecast model followed by a separate cyclone downscaling step.*
We respond to this in R1 Comment 4.

R1 Comment 4: *The manuscript's "paradigm shift" framing feels overstated, because, as currently formulated, the cyclone component is one-way: the predicted cyclone attributes (track, intensity, radii) are produced as a downscaling/diagnostic output conditioned on the large-scale state X, but they do not feed back to influence the subsequent evolution of X. Without a two-way coupling, CycloneCast is best interpreted as a global field forecasting system augmented by a cyclone-aware*

diagnostic/downscaling module, rather than a fully coupled multiscale model in which cyclone-scale evolution can modify the large-scale circulation. This distinction matters for how the results should be claimed: the approach can still be highly valuable operationally, but the text should avoid implying that cyclone structure is being dynamically resolved within the global circulation model or that the system constitutes an end-to-end multiscale modeling breakthrough. In the same vein, if the base model already predicts X strongly, the paper should more clearly justify why joint training of X and cyclone variables C is necessary beyond convenience, given that C does not influence X in the forecast loop.

The reviewer is correct in the assessment that C does not influence X during inference; however, joint training is critical. The cyclone record is comparatively small (fewer than 5,000 historical storms, which are rare in both space and time), whereas the 0.25° atmospheric analysis spans terabytes of gridded data with ∼1 million pixels per timestep. A pure diagnostic model ($X \to C$) would be limited to these ∼5,000 labelled cases, whereas co-training lets the model learn transferable atmospheric representations from the abundant weather data that directly benefit the data-scarce cyclone prediction task ($X \to C$ transfer learning). In turn, the training signal from the specialised best-track cyclone dataset can help the model learn better representations for *atmospheric state forecasting* when cyclones are active (transfer learning $C \to X$). We have now made this argument explicit in Section 2.1. We also verified that cyclone co-training does not degrade atmospheric state prediction skill (Section 2.3).

In response to the reviewer's question we have added two ablation studies. First, co-training with IBTrACS channels substantially reduces intensity error compared with a decoupled approach that relies on a physics-based tracker (C14). Second, co-training significantly reduces position error even when running a physics-based tracker purely on the model's atmospheric state rollouts, i.e. without using the cyclone channels at all (C13). We cite these new results in the main text.

These improvements demonstrate that the joint training process induces a meaningful coupling between X and C, despite C not feeding back into X during the autoregressive rollout. This is an important result, and we thank the reviewer for prompting this investigation.

R1 Comment 5: *The work does not include true data assimilation, limiting end-to-end positioning. The system relies on ECMWF analyses for initialization and introduces "earlification", but it does not perform data assimilation in the standard sense (i.e., conditioning on raw observations with an explicit DA procedure). This limits claims that CycloneCast represents a great leap forward toward end-to-end forecast-and-assimilation systems. I suggest reframing: CycloneCast is an operationally efficient AI ensemble forecast model initialized from analyses, rather than a final solution to this problem.*

We agree that WN-C is not fully end-to-end in the sense of removing all dependence on NWP outputs, and it was not out intention to suggest otherwise. The manuscript now explicitly states that the model is initialised with analysis states from a data assimilation process, and all mentions of end-to-end modelling are in the context of cyclone trajectory prediction. We make clear that interpolation ("earlification") is used to address operational latency and mimic the approach of operational weather centres like NHC, without claiming that it constitutes full DA. We hope the resubmitted manuscript aligns more closely with the framing of an 'operationally efficient AI forecast model', while still highlighting the key modelling innovations in our work (which do constitute a form of end-to-end learning).

R1 Comment 6: ***Detailed comments***
Abstract

L5–6 ("historical database of cyclones"): Please specify the exact source in the abstract (e.g., IBTrACS) and cite it. "Historical database" is too vague for a core dataset.
Thank you for raising this. We now make reference to IBTrACS and cite it in the abstract.

R1 Comment 7: *L7 Is the model driven by real-time updated initial state? Please clarify:*

- *Are forecasts updated in real time with each synoptic cycle?*
- *What is the update frequency (e.g., every 6 hours)?*
- *Is the system initialized from ECMWF analyses operationally, and how does that relate to "timely" forecasts?*

WN-C is initialised from ECMWF operational analysis states and, in operation, produces forecasts at each synoptic cycle (∼every 6 hours). This is described in the Fig. 1 caption ("initialised operationally with atmospheric state initial conditions from ECMWF"), in the main text (Section 2.1: "we condition on initial analysis states $X^{\leq 0}$ from a preceding data assimilation process"), and in the evaluation methodology (Section 3: "forecasts initialised 6 h prior, corrected with the latest real-time cyclone observations"). By "timely" we refer to the fact that each WN-C forecast is generated in ∼2 minutes on a single TPU, enabling rapid delivery of updated guidance.

R1 Comment 8: *L11 ("consensus ensemble"): Please define what you mean by "consensus ensemble" (e.g., TVCN/IVCN-type consensus, weighted average of models, etc.). Many readers will not know this term. This is defined very late in the Method section, making it hard to interpret in sequential reading.*
Thank you for raising this. We have modified this to say "weighted average consensus ensemble" to clarify this point while keeping the abstract statement concise.

R1 Comment 9: ***Section 1 (Introduction)***
L29 ("trade-off of what?"): The manuscript says there is a "trade-off," but it is not clear what two quantities are being traded off. In operational TC forecasting, the key trade-off is often resolution vs ensemble size (and cost). The text currently reads as if there is a trade-off between large-scale circulation and fine-scale thermodynamic processes, but those are both needed and not a trade-off in itself. Please rewrite this passage to state the actual operational constraint and trade-off clearly.
We agree and have rewritten the introduction to clarify this. We now explicitly state that for physics-based NWP models, achieving the high spatial resolution required to capture intensity changes "trades off against domain and ensemble size" due to severe computational costs.

R1 Comment 10: *L34 (resolution comparison): Please ensure the resolution statements are accurate. ECMWF-ENS is ∼9 km, which is finer than the resolution implied by training primarily on ERA5 (0.25°, ∼25–28 km). The manuscript should be explicit about:*

- *the resolution of X used in training/inference,*
- *the resolution of each baseline,*
- *how resolution differences might impact intensity/structure skill.*

We have revised the introduction to accurately state the resolutions of the baseline models (e.g., ENS at 9 km and HAFS with a 2 km inner nest). Crucially, we have clarified that *physics-based* NWP models require these high resolutions to *explicitly* simulate inner core dynamics. This properly

frames the physical constraints of NWP baselines and avoids implying that our AI model physically resolves the core, motivating our use of AI to *implicitly* infer fine-scale cyclone attributes from the large-scale atmospheric state (see next comment).

R1 Comment 11: *L42 ("standard" vs "primitive"): Replace "standard" with "primitive" (or define what "standard" means). These are "standard" only in the sense that they are the primitive variables of atmospheric dynamics.*
Thank you, we have now amended this.

R1 Comment 12: *L44 (missing related literature): Please include more emerging literature in the introduction, especially end-to-end/DA-related ML systems:*

- *FuXi Weather:* `https://www.nature.com/articles/s41467-025-62024-1`
- *Also relevant: generative assimilation and prediction ideas (GAP):* `https://arxiv.org/abs/2503.03038`

Thank you for your suggestion, we have now cited both of these works.

R1 Comment 13: *L54 (how ensemble members are generated here?): You mention uncertainty sources (initial state uncertainty, model uncertainty, stochasticity). Please state concretely, in the introduction:*

- *how CycloneCast generates ensemble members;*
- *whether variability comes from parameter noise only, deep ensembles only, or both;*
- *whether any initial condition perturbation is performed (or if not, how initial uncertainty is represented).*

We have now made up-front reference to the mechanisms of randomness in the model (deep ensembles and parameter noise). We also made reference to the fact that we treat initial conditions as exactly known, i.e. we do not add any initial perturbations (in section 2). We feel that going into much more detail up front could make the introduction too long and might throw off readers, and are leaning towards keeping the introduction concise. We believe this is a good compromise and hope it addresses your concern.

R1 Comment 14: *L58 (cyclone dataset curation and trustworthiness): Please provide references and concise description of:*

- *which cyclone dataset is used,*
- *how it is curated/quality-controlled,*
- *uncertainties in wind radii and intensity estimates.*

This is critical because cyclone structure targets are non-trivial observational products.
We are using the IBTrACS dataset, which is considered to be the most complete historical record available. We have added explicit reference to IBTrACS in the text and a citation. IBTrACS consists of a collection of historical hurricane data from a range of different RSMCs that have already been carefully quality controlled by several RSMCs around the world. We defer to IBTrACS documentation `https://www.ncei.noaa.gov/sites/default/files/2025-04/IBTrACS_`

`version4r01_Technical_Details.pdf` which give details on the uncertainty in the radii and intensity estimates.

R1 Comment 15: *L53–60 (disconnect with limitations): The introduction motivates difficulty from inability to resolve cyclone inner-core structure in global models. However, CycloneCast is still trained on coarse global analyses for X. The writing gives the impression that the model "learns resolved fine-scale structure," but it appears instead to learn cyclone parameters conditioned on coarse X. Please rewrite to avoid implying that the model resolves fine-scale structure in a dynamical sense.*
The revised text uses "infer" rather than "resolve/simulate," consistent with the model's data-driven approach (see section 2). We have updated the introduction section in line with your suggestions.

R1 Comment 16: *L60 ("fine-scale core dynamics"): I suggest revising to "predicts cyclone intensity/structure parameters" rather than "simulates fine-scale core dynamics."*
We have now removed this sentence in a rework of this section.

R1 Comment 17: *L69 ("best . . . "): Please clarify what is meant by "most skillful":*

- *most skillful for cyclones or for global fields?*
- *relative to which baseline(s)?*
- *up to what lead times and in what basins?*

Avoid broad "best overall" language unless the scope is precisely defined.
We have kept "most skillful" as we believe it is justified: WN-C is comprehensively better across track, intensity, structure, and rapid intensification metrics, against all evaluated baselines including both NWP and AI systems, across lead times and ocean basins. The supplementary evaluations—including geospatial breakdowns by basin—further support this claim. We have ensured the main text references these comprehensive evaluations.

R1 Comment 18: ***Figure 1 and Caption***

- *Figure (green arrow): Please clarify whether the green arrow represents the atmospheric state or the evolution/rollout of atmospheric state.*
- *Caption grammar: "A CycloneCast rollout" (not "An").*
- *Caption structure: Put the context sentence earlier: "Rollout initialized on Monday Oct 7 . . . Hurricane Milton . . . all times UTC" should appear at the beginning. Key confusion: The sentence "at each forecast step. . . predicts . . . 6 h ahead" reads like a 6-hour lead-time forecast only. Please rephrase explicitly:*
  - *CycloneCast produces iterative 6-hour steps out to 15 days,*
  - *each step predicts the next 6-hour increment, fed autoregressively.*

Done. We have revised the Figure 1 caption to address all of these points. The context sentence is now at the beginning, the grammatical typo is fixed, and the text now explicitly states that the model produces "iterative 6 h forecasts... out to 15 days, by autoregressively feeding its atmospheric state prediction as input to the next model step (green arrows)."

R1 Comment 19: ***Section 2***
Section title / flow
L72 title ("dense and sparse predictions"): This title is not very clear to the meteorology community. Consider renaming to emphasize joint prediction of gridded fields and cyclone attributes.
We have changed the title to "An ensemble weather and cyclone model"

R1 Comment 20: *L74–87 (loose structure): The introduction already established key facts. Here you can be more compact and formal: define the task directly as joint probabilistic learning of* $p(\{X, C\}^{1:T} \mid X^{\leq 0})$
We have amended the section in line with your suggestions making it tighter and putting the conditional modelling task more up-front.

R1 Comment 21: *L89: "prior state" → "prior states".*
Note that we have removed the phrase "prior state" in the most recent version of the manuscript.

R1 Comment 22: *L90–92 ("standard practice . . . do not condition on raw observations"): This is misleading. Operational weather forecasting does condition on observations through data assimilation. A more accurate statement is: "forecasts are initialized from analysis states produced by a DA system," rather than implying forecasts do not condition on observations.*
Thank you for the suggestion. The point we were trying to get across is that the *forecasting step* that is initialised with the output of DA does not itself condition on the raw observations, e.g. satellite radiance. However we agree that our phrasing could mislead the reader so we have amended the text to mention DA and remove the comment on raw data by: "Following standard operational practice, we condition on initial analysis states $X^{\leq 0}$ from a preceding data assimilation process."

R1 Comment 23: *L95–96 (independence of C_t from C_{t-1}): As written, it seems C_t depends on $X_{t-2:t-1}$, but not explicitly on C_{t-1}. This could hurt temporal continuity of cyclone attributes. In meteorology this resembles statistical downscaling/diagnostic modeling. Please:*

- *explain why this conditional independence is assumed,*
- *discuss limitations (possible temporal inconsistency),*
- *provide evidence that continuity is adequate.*

The conditional independence assumption $p(C^t \mid X^{t-2:t-1})$ rather than $p(C^t \mid X^{t-2:t-1}, C^{t-1})$ is a deliberate design choice. Our results demonstrate that a state-of-the-art model can be trained under this assumption, suggesting that sufficient information about C^t is contained within $X^{t-2:t-1}$. There is also a practical motivation: at inference time, the model is initialised with real-time TCVitals data, which has different characteristics and error profiles from the IBTrACS training targets. Conditioning the network on C^{t-1} would expose it to this distribution mismatch, potentially degrading robustness. Additionally, the smooth evolution of the autoregressive atmospheric state and the spatial proximity constraints in the tracking algorithm both encourage temporal coherence in the predicted cyclone attributes.

Regarding evidence, two of our evaluations address temporal continuity indirectly:

1. **Rapid intensification**: this metric evaluates skill at classifying ≥30 kt intensity changes within 24 h. Temporal discontinuities in the predicted intensity would produce many false positives, which we do not observe—WN-C achieves state-of-the-art RI skill.

2. **Cumulative wind probabilities**: these are computed by interpolating between 6-hourly timesteps to ensure all areas swept by the cyclone are captured. If tracks had poor temporal consistency, the large jumps between timesteps would cause the interpolate swaths to expand unrealistically, inflating the affected area. We do not observe this.

While we cannot rule out minor inconsistencies, forecasters have not flagged temporal discontinuities since WN-C was deployed operationally on WeatherLab. Exploring the potential benefits of conditioning on C^{t-1} remains an interesting direction for future work.

R1 Comment 24: *L105 ("trained ... up to 15 days ... 6-hour timestep"): Please clarify training exactly:*

- *Is the training loss computed only at 6h lead time (one-step), or on multi-step rollouts?*
- *If multi-step, how is loss aggregated across lead times (uniform average vs weighted)?*

Your later statements about averaging rollout loss need to be clearly tied back here.
We have now modified the sentence (positioned right after equation 1 now), to clarify and to explicitly link to the training protocol.

R1 Comment 25: *L110–115 (coarse analysis limitation): You criticize the two-step approach because coarse fields underestimate intensity. But CycloneCast also uses coarse analysis fields for X. How does CycloneCast actually address this? The current text states the limitation but does not clearly show how your design resolves it.*
Thank you for raising this. The fundamental limitation is that existing models predict surface wind (10U and 10V components) and then the tracker (e.g. Tempest Extremes) simply reads off the maximum wind speed. This process inherits the low-bias of the surface winds. WN-Cgets around this issue by directly learning to predict the cyclone variables from X, so these cyclone predictions are not constrained by the low-bias of the surface wind: instead, the model can learn to make accurate inferences about the cyclone parameters based on the rich signal provided by all the atmospheric variables in X. As evidenced by the performance of the model, this is a much better approach than simply reading off the surface winds. We appreciate your suggestion for improvement and have changed the phrasing to "However, coarse analysis data systematically underestimate cyclone intensity, and training on atmospheric states alone provides no direct learning signal for cyclone-specific features" to highlight that training to predict the cyclone variables from the atmospheric variables is a key feature of the approach for this reason.

R1 Comment 26: *L126 ("centered at a given grid point"): More precise wording: a cyclone center can be "within a grid cell," not "centered at a grid point."*
We have rewritten this part and this phrase no longer appears.

R1 Comment 27: *L128 ("12 quadrant-wise wind radii"): Please explicitly state 3 thresholds $\times$ 4 quadrants = 12.*
This has now been removed while shortening the section, similarly to the comment above.

R1 Comment 28: *L143–149 (continuity / failure cases): Since cyclone information is extracted via existence probability + tracking, please discuss:*

- *how temporal continuity is ensured,*
- *whether discontinuities occur (e.g., blob splitting/merging, sudden jumps),*

- *whether there are failure cases where track/existence becomes inconsistent.*

We respond to the temporal continuity question in R1 Comment 23. Regarding blobs merging, we have not seen this happen, and indeed this does not happen in the griddified IBTrACS training data other than rare cases of the Fujiwhara effect. Regarding track/existence becoming inconsistent, our tracking algorithm described in Section 10.3 is designed carefully to follow the Gaussian kernel-like features in WN-C's existence probability channel (E) by applying lower bounds on the probability mass within a fixed distance of the lat/lon, so we are confident that the lat/lon of the tracker differing from E is not a concern.

R1 Comment 29: ***Section 2.3 (Uncertainty / FGN)***
L154–162 (missing uncertainty source): You mention epistemic and initial-state uncertainty. But a third major uncertainty source is stochasticity due to coarse representation and unresolved processes, plus chaotic growth with time. Please discuss this explicitly and relate to superensemble ideas (e.g., Krishnamurti, T. N., Chandra M. Kishtawal, Timothy E. LaRow, David R. Bachiochi, Zhan Zhang, C. Eric Williford, Sulochana Gadgil, and Sajani Surendran. "Improved weather and seasonal climate forecasts from multimodel superensemble." Science 285, no. 5433 (1999): 1548-1550., as well as GenEPS: `https://www.nature.com/articles/s41612-025-01255-x`).
We agree and have expanded the aleatoric uncertainty subsection in the Methods to explicitly name these sources: the coarse 0.25° representation leaving sub-grid processes unresolved, and chaotic error amplification growing over lead time. We now cite Buizza (1999) and Palmer (2001) for stochastic parametrisation in NWP, Krishnamurti et al. (1999) for multi-model superensemble approaches, and Brecht et al. (2025) for the recent GenEPS system. The text draws an explicit analogy between our learned parameter perturbations and NWP stochastic physics.

R1 Comment 30: *L163–170 (initial state uncertainty): I cannot see how FGN captures or reduces initial state estimation uncertainty. If the model is conditioned on an analysis state without explicit perturbation of initial states, please clarify what "initial condition uncertainty" means in this context and how it is represented.*
We agree with the reviewer and have corrected the text. FGN captures epistemic (model) uncertainty and aleatoric (stochastic dynamics / unresolved scale) uncertainty. Because we do not explicitly perturb the input analysis states, we do not sample over initial condition uncertainty. The text has been revised to make this more clear.

R1 Comment 31: *L166 (distribution over parameters): What distribution is used to perturb neural network parameters? Gaussian? How is it parameterized (global noise vector, conditional layer norm, low-rank covariance)? Please provide:*

- *explicit definition,*
- *rationale for this choice,*
- *and any theoretical/empirical support.*

We have added a parenthetical clarification in the main text: the learned distribution is implemented via a low-dimensional (32-d) Gaussian noise vector injected through conditional normalisation layers. The full details and theoretical justification (Alet et al., 2022) are in the Methods.

R1 Comment 32: *L167 (marginal fCRPS in high-dimensional fields): fCRPS is proper for univariate distributions, but you predict a high-dimensional multivariate field. Please explain:*

- *how you weight the CRPS losses across initial cases, variables, locations, and levels,*
- *whether all variables/locations are equally weighted (your description suggests they are),*
- *and why this does not harm spatial structure (spectra, coherence).*

We have added a table in the Supplementary Material listing all per-variable loss weights. The weights are *not* uniform: weather variable weights follow GraphCast (Lam et al., 2023), with geopotential halved to 0.5; cyclone existence, wind speed, and pressure variables are upweighted to 10 to compensate for spatial sparsity. The Methods text now references this table directly.

Regarding spatial structure, see R1 Comment 2.

R1 Comment 33: *L174 ("loss averaged over rollout steps"): You average fCRPS across rollout steps. Please justify why uniform averaging across lead times is appropriate. Would a decaying factor be more reasonable, especially with only N=2 members per batch during training?*
While a time-decaying factor could theoretically prioritise early-step accuracy, we found that uniform weighting encourages the model to maintain stable, non-divergent dynamics across the entire autoregressive window (15 days. It is important to note two things: 1) we do most of the training at 1 step, ensuring the model is good before going for auto-regressive fine-tuning. 2) we want the model to generalize to 15 days but only train it on 3 days. By not decaying the factor, our loss depends more on the longer days and generalizes a bit better to the lead times we do not train on.

R1 Comment 34: *L181 (GenCast needs 40 runs): Please add a brief explanation for readers unfamiliar with diffusion models: why does GenCast require ∼40 denoiser calls per forecast step?*
Done. We added a brief explanation noting that GenCast is a diffusion model, which requires an iterative reverse-diffusion process to gradually denoise the state into a coherent forecast at each timestep.

R1 Comment 35: *L182 (8× faster to sample): Please specify the hardware / compute setting for this claim (e.g., GPU type, batch size).* We have added a "Computational resources" paragraph to the Methods section, specifying: training takes approximately 4 wall-clock days per model seed, using a combined total of 560 TPUv5p and TPUv6e days of compute. For inference, generating a single 15-day forecast trajectory takes just under 1 minute on a single TPU v5p, and every ensemble member can be generated in parallel. The 8× speedup relative to GenCast is a direct consequence of requiring only a single forward pass per forecast step, compared with the ∼40 denoiser calls of GenCast's iterative diffusion process. Batch size and other details can be found in methods as well.

R1 Comment 36: *L183 ("sparse target"): Please define "sparse targets" explicitly (cyclone targets only present near cyclone centers).*
Done. We have explicitly defined sparsity in tabular cyclone data as variables that are only defined locally around active cyclone centers, leaving the rest of the global grid masked.

R1 Comment 37: *L184–185 (difficulty following the point): Please clarify:*

- *Why do autoencoders/diffusion frameworks "struggle" with sparse targets in your view?*
- *Since cyclone variables do not feed back into X, one could forecast X with a strong generative ensemble model and then run a cyclone diagnostic model afterward. Why is end-to-end joint prediction necessary here, beyond convenience?*

FGN makes it very easy to train on processes of conditional sparsity. In our case, we can easily ask the model what the cyclone intensity would be if a cyclone were to be in a given position without leaking the position into the model.

Conversely, in the encoder part of an auto-encoder it would be harder to feed the intensity of the cyclone at a particular location without simultaneously leaking the given location. Similarly, for diffusion, we cannot use NaNs for places away from cyclones as they have to be ingested as inputs. If we use a placeholder value for NaNs outside the range of possible values, then the denoiser would easily catch up on it, immediately detecting the location of the cyclone. Although possible, it would probably require heavy tuning or architectural modifications.

Regarding joint training, please see R1 Comment 4.

R1 Comment 38: ***Section 3 (Evaluation methodology)***
L187–192 (paired/unpaired naming): The idea of separating formed vs not-yet-formed cyclones is valuable, but I suggest:

- *introducing cyclone genesis as a major challenge earlier in the Introduction,*
- *clarifying why these are called "paired" and "unpaired" (not standard terminology).*

Done. We have introduced cyclogenesis earlier as a major forecasting challenge. We also clarified our terminology in the Evaluation section: "paired" refers to matching predicted tracks strictly to existing, observed best tracks (standard operational verification), while "unpaired" evaluates all forecasted tracks globally to capture genesis, dissipations, and false alarms.

R1 Comment 39: *L201 ("performed similarly"): This is ambiguous. "Performed similarly" should specify:*

- *which metric(s),*
- *which variable(s),*
- *which lead times,*
- *and which basins/regions/resolutions.*

Thank you for flagging this. We have removed this "performed similarly" claim for ECMWF ENS, and for HAFS/HWRF/HMON we have detailed the specific variables with a reference the Extended Results to back up the claim.

R1 Comment 40: *L204–207 (HAFS reference): Please add references/citations for the exact HAFS configuration (HAFS-A) and explain the evaluation domain limitation.*
Done. We have updated the text in the Evaluation Methodology to explicitly describe the operational HAFS-A configuration. We now detail that it employs a 6 km regional parent domain with a 2 km storm-following moving nest to explicitly resolve core dynamics, and we explicitly state that its forecasts are used exclusively in the North Atlantic and East Pacific basins because that is where the regional model is operationally run.

R1 Comment 41: *L208–210 (bias correction for baseline GenCast): Why apply a bias correction to the baseline model rather than evaluating raw performance? Please justify clearly and ideally present results with and without the correction.*
We applied the bias correction to GenCast to provide the strongest possible AI baseline for intensity. Without this correction, raw GenCast severely underestimates intensity, making the comparison

trivially easy for WN-C. By correcting it using a physical pressure-wind relation, we isolate the true value of our co-training approach. For transparency, we have added the raw (unmodified) GenCast intensity results to the Supplementary Information alongside the debiased version. See figure entitled "Debiasing with a learned pressure–wind speed relation strongly improves the MAE and bias of GenCast intensity forecasts."

R1 Comment 42: *L211 (late models depend on analyses): Not all recent data-driven forecasting models depend exclusively on analyses; some incorporate DA or observation-to-forecast pipelines. Please update this statement and cite:*

- *FuXi Weather (Nat Commun 2025):* `https://www.nature.com/articles/s41467-025-62024-1`
- *GAP (arXiv 2503.03038):* `https://arxiv.org/abs/2503.03038`

We acknowledge that recent work incorporates direct observations (e.g., FuXi Weather, GAP). We have softened this statement to clarify that we are specifically referring to models initialised purely from reanalysis or analysis states (like GraphCast and GenCast). Note that we have also cited FuXi and GAP earlier in the introduction, as per your earlier suggestions.

R1 Comment 43: *L217–218 (earlification placement): Earlification appears central for operational evaluation and related to initialization error. It should be described earlier (main text and Methods), not only later, and framed explicitly as a strategy for operational latency.*
Done. We have moved the conceptual explanation of earlification (correcting late forecasts with real-time TCVitals) earlier in the text and explicitly framed it as a necessary strategy for bridging operational latency, and added a schematic of the process in Extended Data Fig. 3.

R1 Comment 44: *L235–237 (confusing training/evaluation description): I am confused:*

- *what is the benefit of retraining/finetuning each year,*
- *whether you evaluate a given model on the next year only,*
- *and what "two years combined" refers to exactly.*

Thank you for raising this question. *Referee #2* also raised a similar question. The reason for this is to simulate the way that the model would be trained and deployed in the real world. For example, to test model performance on the 2023 season, we train it on data up to and including 2022 (without any data from 2023 onwards), and then test it on 2023. Subsequently, to test the model on the 2024 season, we train it on data up to and including 2023, i.e. one extra year of data that would have been available after the 2023 season is complete. In this manner, we ensure that we leverage as much training data as possible for each test year, while keeping the training and test sets disjoint in a causal manner. Nothing else changes about the model training or protocol from one year to the next. "Two years combined" simply refers to pooling the test metrics from the out-of-sample periods to increase statistical power.

R1 Comment 45: *L247 (Appendix B.1 insufficient): Section B.1 does not provide enough detail for the evaluation strategy and significance testing. Please summarize the main statistical testing in the Methods.*
We have now deferred the outline of the statistical testing methodology to the section entitled "Statistical significance" in the appendix, and the results of the statistical tests to the SI figures. Please let us know if this does not satisfy your concern.

R1 Comment 46: *L249 ("always provide the best guidance"): This is too strong. Please be specific: do you mean consensus means often outperform any single model on mean track error? Also, CycloneCast is not strictly a single model (it is a deep ensemble). Please clarify terminology.*
Thank you for raising this. We have softened the language used here and added more details to clarify the terminology. While our model is a deep ensemble, in the context of consensus ensembles we consider its deterministic ensemble mean, in a similar way to how consensus models often contain the ENS or GEFS ensemble mean as a single consensus member. We have clarified that we consider WN-Cas a single guidance, analogously to ENS and GEFS.

R1 Comment 47: ***Section 4 (Global weather forecasting)***
L258–263: The text discusses comparison to ENS but the presentation seems uneven (sometimes only GenCast comparisons are highlighted). If ENS comparisons are central, they should be summarized clearly in the main text.
Perhaps we are misunderstanding this point, but the original paragraph did mention ENS explicitly: "it also significantly outperforms ENS on 99.2 (p ¡ 0.05) of all evaluated variables, levels, and lead times out to 15 days..." In general, we have aimed for comparisons to ENS and GenCast to be similar except for ENS being used also for quadrant radii.

R1 Comment 48: *Many results are pushed to Appendix B.2; operational readers would benefit from a summary table/figure in the main text.*
While we agree a summary figure would be helpful, due to Nature's strict limits on main-text display items, we have retained the detailed scorecards in the Extended Data / Supplementary Information. We ensured the main text clearly and quantitatively summarizes the exact lead-time improvements over ENS and GenCast.

R1 Comment 49: ***Section 5 (Track)***
Overlap: The content in L286–287 overlaps with L297–305. The section can be made much more compact and clear:

- *first present deterministic ensemble-mean track error,*
- *then probabilistic score,*
- *then calibration (spread/skill), without repeating similar statements.*

We appreciate your comment. However, we would like to point out that position energy score and spread-skill capture different notions of uncertainty and calibration, and even though they might appear repeated content, they are not. Spread-skill is computed by calculating the average spread and the average skill and dividing one by the other. It is therefore a "global" assessment of how closely the average spread of a model matches its average skill. By contrast, position energy score (PES) is a different notion of uncertainty that can be computed on a per-forecast basis: for each probabilistic forecast, PES is computed by taking the difference between the average position error and the average positional spread of the ensemble members (so it is computing the difference between a skill-like term and a spread-like term as well). Therefore these two notions are different and complementary: one is measuring situational calibration and the other is measuring global calibration, and they help give a more complete picture of the model's performance. On account of this, we are leaning towards keeping the current structure. That being said, we have slightly edited the text to reduce repetition, while keeping the parts distinct.

R1 Comment 50: ***Section 6 (Intensity and structure)***
L311: CycloneCast does not "resolve" cyclone structure dynamically; it is more a statistical downscaling/diagnostic model for cyclone parameters. Please adjust wording.
We have now replaced this with alternative phrasing: "coarse analysis data that does not accurately match cyclone's true intensity." Though we do note that the original statement was saying that the *coarse analysis data* do not resolve the cyclone intensity, i.e. it was a statement about the data not about WN-C. However, we do think that the alternative wording improves the sentence. Thank you for the suggestion.

R1 Comment 51: *L313–318 (fairness): It is not fair to compare CycloneCast ensemble mean directly against a single deterministic NWP member. Please include:*

- *comparison against individual CycloneCast members, or*
- *compare ensemble means consistently (e.g., against ENS mean), and explain why ensemble-mean comparison is operationally appropriate.*

This is explicitly addressed in the Supplementary Material ("Ensemble mean comparisons"). We show both individual member and ensemble-mean comparisons, explaining why comparing the 50-member ensemble mean against deterministic models is appropriate: the ensemble mean is the natural point forecast from any ensemble system, and HRES/HWRF are already optimised as single-trajectory forecasters.

R1 Comment 52: ***Rapid intensification (RI)***
Your RI evaluation is interesting, but please ensure definitions and interpretation are clear for readers:

- *define RI early (the threshold),*
- *explain how ensemble probabilities map to decision thresholds,*
- *and present reliability in a straightforward way.*

RI is now defined with its threshold ($\geq$30 kt in 24 h) at its first mention in the Evaluation Methodology. The ensemble-to-decision mapping is now explicit: we state the decision threshold as the minimum fraction of members predicting RI. The reliability diagram description is unchanged.

R1 Comment 53: ***Section 8 (Operational guidance / consensus)***
"Consensus ensemble" should be defined earlier (not first appearing late). Many readers will not know TVCN/IVCN.
Thank you – we now make reference to consensus models earlier, in the evaluation methodology (Section 3).

R1 Comment 54: *Please clarify what exactly is optimized (weights), on what data (validation), and whether weights vary with lead time or basin.*
Thank you – we have now added clarifying details in this section.

R1 Comment 55: ***Section 9 (Conclusion)***
L432 ("overcome"): "Overcome" is too strong given:

- *no true assimilation,*

- *cyclone variables are largely downscaled,*
- *and physical coupling is limited.*

Please soften this language.
Thank you for the constructive criticism. We agree that this language could be softened and have changed the opening line to “This paper introduced WN-C, an AI system that addresses a long-standing challenge in tropical cyclone forecasting.” We have also changed a later usage of “overcome”: ”while co-training on extreme and analysis weather data effectively bypasses the data scarcity of extreme events”.

R1 Comment 56: *L446 (“double-penalty problem”): Please briefly explain what this means for non-specialists.*
Thank you for your suggestion. You’re right, perhaps non-specialists might be unfamiliar with this term. Since this problem manifests as spatial smoothing in the forecasts, a concept that non-specialists are unlikely to be confused by, we have changed this wording to “we avoid the spatial smoothing problems plaguing deterministic models.” We have opted for this because we think it’s likely a better approach than to introduce a new concept in the conclusion. We hope this addresses your point.

R1 Comment 57: *L447 (“co-training”): If cyclone variables do not influence X, the manuscript should explain why joint co-training is necessary rather than training separate modules (global forecast + cyclone downscaler).*
Thank you for raising this subtle point! There are two answers to this: First having a single model that tackles both tasks is a simpler approach than having two modules, which makes the model easier to maintain and iterate on (by contrast, iterating on the model by adding more modules can make the approach difficult to scale, version control and so on). We have made a modification to the text to reflect this argument for simplicitly. Second, we have also found that co-training on cyclone variables slightly improves performance on forecasting cyclone tracks, *even without using the cyclone variables C at inference + tracking time.* Specifically, we have added an ablation figure entitled “Ablation: effect of IBTrACS co-training” which shows that co-training a model on atmospheric and cyclone variables and then running tracking on it with Tempest Extremes improves performance over a baseline that is not co-trained on cyclone variables. In essence, C does not affect X directly through the probabilistic rollout, but it *can affect performance via training.* We hope this additional ablation and modifications help address your points.

R1 Comment 58: ***Methods Section (Specific)***
Layout: I find the layout confusing. I recommend moving the current Sec. 2 and Sec. 3 content into the Methods, then clearly presenting:

1. *learning task definition,*
2. *cyclone downscaling/griddification + tracker,*
3. *FGN uncertainty model + deep ensembles,*
4. *training objective/weighting and rollout strategy,*
5. *evaluation + statistical tools.*

We appreciate this suggestion. Following the editor's guidance on Nature's format (main text ≤4,500 words with a separate Methods section), we have restructured the paper: significant portions of the evaluation methodology and problem formulation detail have been moved into the Methods, and the main-text evaluation section has been compressed substantially (from ∼855 to ∼335 words). We retain a concise overview of the problem formulation in the main text to make the paper self-contained for a broad readership, as recommended by the editor.

R1 Comment 59: *L665–666: Please avoid vague phrases like "some wind speed probabilities evaluation." Be specific: what evaluations, what source, what quantitative basis?*
Thank you for the suggestion. We have now removed this phrasing entirely in the most recent version of the manuscript, after updating this section.

R1 Comment 60: *L672: Clarify that this statement applies to the cyclone-variable loss/training, not the large-scale atmospheric field prediction model (FGN).*
In our updated version, we have removed the phrase "These fields serve as prediction targets for the model" that we believe you are referring to, which we believe should address any potential confusion here.

R1 Comment 61: *L730 (threshold = 0.3): Please justify why 0.3 is chosen (sensitivity test or tuning procedure).*
We heuristically set this parameter, based on the fact that the ground existence kernels are unit-mode Gaussians, so this heuristically selected threshold quickly rules out candidates with low existence probabilities. We have amended the text to add details on this.

R1 Comment 62: *L780 (J=4 deep ensemble): Please provide an ablation: what happens with fewer than 4 models? Since you call this a trade-off, show that it is a reasonable one.*
The $J = 4$ deep ensemble operates at the level of full model retraining, not sample generation. Each ensemble member is an independently trained copy of the full model, so J directly multiplies the total training cost. At training time, each member uses only $N = 2$ stochastic samples to compute an unbiased CRPS gradient (see our response to Comment 1.5). Training with $N = 2$ rather than $N = 4$ samples per member allows us to average over twice as many distinct input examples per batch for the same TPU budget, which we found more beneficial than reducing gradient variance with more samples on the same input. We have informally tested $J > 4$ and observed no significant gains, though we have not explored this axis exhaustively enough to include a formal ablation in the paper. We have clarified the trade-off in the Methods.

R1 Comment 63: *L789 / L803–807 (form of $\mathcal{P}_{\mathcal{M}}(\theta)$): You later define a Gaussian-like parameterization. Please explain:*

- *why this choice,*
- *what assumptions it encodes,*
- *and whether alternatives were tested.*

The Gaussian is the most general and commonly used distribution for continuous noise injection, and is a natural choice for a reparameterization-based approach. Importantly, while the noise vector $z \sim \mathcal{N}(0, I)$ is i.i.d. Gaussian in 32 dimensions, this does *not* imply Gaussian-distributed model outputs. The noise is injected into the network via conditional layer-norm layers, which apply it through highly nonlinear transformations with extensive parameter sharing across spatial

locations and variables. The resulting output perturbations are therefore complex, structured, and non-Gaussian. We did not test alternative noise distributions (e.g., uniform), as the Gaussian parameterization worked well and is standard practice.

R1 Comment 64: *L800 (spatially-dependent perturbations): Please clarify what you mean and why they cause high-frequency components that "need to be tamed."*
By this, we mean that the cited approaches use perturbations on the input introduce high frequency artifacts that hurt performance and need to be mitigated by specialised techniques that add complexity to the training protocol. We have changed this section to better explain this point.

R1 Comment 65: *L827–829 (parameter noise vs input noise): This is an important discussion point. Generative state-space perturbations (input noise) often preserve structure and spectra better. Please discuss explicitly:*

- *what structural advantages you gain/lose,*
- *and provide diagnostics (spectra, long-term rollout behavior).*

See R1 Comment 2.
We have informally observed that injecting i.i.d. noise directly at the input or output level tends to produce high-spatial-frequency artifacts in the generated fields. Other groups have encountered similar issues and had to introduce additional loss terms or architectural modifications to mitigate them—for example, NeuralGCM applies spectral filtering, and Lang et al. penalise ensemble variance and apply a convolutional filter to suppress the resulting high-frequency artifacts. By injecting noise in parameter space via conditional layer normalisation, the perturbations are spatially smooth by construction, since the same noise vector modulates the entire field through learned, shared network weights. We emphasise that our results demonstrate that parameter-space noise works well for this application; we do not claim that input-space noise cannot be made to work with appropriate additional engineering.

R1 Comment 66: *Sec. 13 (baseline modification): I question whether it is necessary to modify the baseline in this paper. If you keep it, please show results both with and without the modification.*
We have conducted an additional evaluation before and after our modification to justify this point, see "Extended comparisons to other baselines" in the appendix. These results show a clear advantage of incorporating the pressure-windpseed relation on GenCast. In particular, this significantly helps with the commonly observed effect of low-biased intensity forecasts that has been observed in the literature, e.g. by DeMaria et al; (2025).

R1 Comment 67: *L942–943 (define consensus ensemble earlier): Please define "consensus ensemble" early in main text.*
Thank you, we now define consensus ensembles earlier in the text, in section 3 "Evaluation methodology."

R1 Comment 68: ***Recommendation***
The contribution is potentially important, but the manuscript needs substantial improvement in (i) clarity and organization, (ii) careful positioning and toned-down claims, and (iii) deeper justification/diagnostics for the chosen uncertainty modeling and the (currently one-way) coupling between cyclone parameters and atmospheric state.

We thank the reviewer for this assessment. By comprehensively reorganizing the manuscript, toning down the coupling claims, and providing deep diagnostics into the FGN and co-training mechanisms, we believe we have fully addressed these concerns and significantly strengthened the paper.

R1 Comment 69: ***Remarks on code availability:*** *The current code provides "high-level" code for the usage of the predictor, the tabular data processing, and the cyclone tracker. While it is helpful for understanding the usage, and the cyclone data processing, it is not enough for a comprehensive understanding of the algorithm, including its model architecture and the training.*
See Section 1.1 for our unified response on code availability and open-sourcing.

4 Response to Referee #2

R2 Comment 1: *I co-reviewed this manuscript with one of the reviewers. The main contents of my review are included in one of the referee reports. Here are some concerns of my own that I want the authors to answer.*
Thank you for your time and effort in reviewing our manuscript, and for your detailed feedback. We respond to each of your queries in detail below.

R2 Comment 2: *L8: "Structure" is not appropriate. Tropical cyclone structure includes vertical structure and horizontal structure. "Wind circle radius" is a precise word. Also, in L23 and so on.*
Thank you, we have now changed the term in the abstract to "size" because we think this is likely appropriate for the general Nature audience, without misusing terminology. We have also changed "structure" to "wind radii" in several instances in the main text following your suggestion.

R2 Comment 3: *L29: trade-off is not appropriate; dilemma is more precise.*
We agree. We have revised the text to refer to the overarching physical dichotomy as a "scale dilemma," reserving the term "trade off" specifically for the computational constraints between resolution and ensemble size.

R2 Comment 4: *L120: what is stitching algorithm?*
By "stitching algorithm" we are referring to an algorithm which, connects a sequence of nodes representing cyclone centers at different lead times, across time. Note that this is existing terminology which we did not introduce ourselves, e.g. see the Tempest Extremes documentation page at `https://climate.ucdavis.edu/tempestextremes.php`, which makes reference to its StitchNodes and StitchBlobs routines. Note however that we have removed reference to stitching in the main text. We now make reference to it in the methods section with an appropriate citation to Tempest Extremes.

R2 Comment 5: *L130-134: what are blobs? I do not quite follow what you want to express. The "standard technique" for computer science may not be comprehensible for readers in the other disciplines.*
We have now changed the term "blob" to "kernel" throughout. We originally used the term "blob" to refer to distinct areas of high probability of existence (in either the gridded data or the model predictions). We agree that the term "blob" would likely be unclear to the general audience, hence why we have replaced it by "kernel".

R2 Comment 6: *L135-142: In L125-L129 "A set of grids where each grid represents the value of a cyclone property (e.g. maximum wind speed) if a cyclone were centered at a given grid point." I don't quite understand why you define your target within a circular region of radius 120 km, since you define the cyclone property only on the grid point of cyclone center. I know you explain it in the appendix, but reader might get confused here.*
Thank you for raising this. We have now changed this section significantly to shorten it and simplify it, deferring these details to the appendix (section 10).

R2 Comment 7: *L143-152: How does blob-tracking algorithm work. Why cyclone properties are extracted by bilinear interpolation on scalar fields. You should make the reader more comfortable. Not all readers want to refer to the appendix for details. You should make the readers understand what you are talking about by making a brief introduction of your whole idea here.*

Similarly to the point above, we have modified this section to shorten and simplify it. We agree with you that some readers are likely not going to be comfortable if we mention some details by name without explaining further, e.g. just saying “bilinear interpolation” without further explanation. We have tried to roughly follow your advice of explaining the highl level idea in the main text, but deferring the details to the appendix so that we don’t confuse the reader or make them feel uncomfortable (see section 10). We think this is a good trade-off between readability and accuracy, and hope that our modification addresses your concern.

R2 Comment 8: *L166: Why the neural network weight is sampled from a learned distribution, how does it work? explain it with more details.*

Thank you for raising this point. We have now included more details in the supplementary section, on the way the noise vector is sampled and injected into the network (effectively acting as sampling from a distribution of low dimensional perturbation around a learned mean). In principle, we could directly sample all of the weights, but leveraging conditional normalization works well and is efficient to implement.

Together with the *deep ensemble*, we can see this as sampling from a multinomial distribution of these low-dimensional distributions. Note that this is equivalent to what in ML is called a ”hyper-network” that receives i.i.d. gaussian noise as an extra input. We think the view of learning a low-dimensional parameter distribution combines better with the deep ensemble (which also samples weights) and is analogous to stochastic parameterizations in the weather literature.

R2 Comment 9: *L211-229: This paragraph is not necessary. I think it can be thrown to the appendix.*

Thank you for the suggestion. We agree that this was probably a bit too much information for the main text. Since this is an important part of the evaluation, particularly for ensuring operational realism, we are leaning towards keeping part of this in the main text to make the reader aware, and have deferred details to the section “Early forecast construction via interpolation” in the appendix, including a visualisation in our extended data. Thank you for suggesting this.

R2 Comment 10: *L237: “For each calendar year of evaluation, the model was retrained (with the frozen protocol) on data up to the end of the preceding calendar year.” Why?*

Thank you for raising this question. The reason for this is to simulate the way that the model would be trained and deployed in the real world. For example, to test model performance on the 2023 season, we train it on data up to and including 2022 (without any data from 2023 onwards), and then test it on 2023. Subsequently, to test the model on the 2024 season, we train it on data up to and including 2023, i.e. one extra year of data that would have been available after the 2023 season is complete. In this manner, we ensure that we leverage as much training data as possible for each test year, while keeping the training and test sets disjoint in a causal manner. Nothing else changes about the model training or protocol from one year to the next.

R2 Comment 11: *L308-311: These lines appear here again. These are almost identical to L107-L115, no need.*

Thank you for your suggestion. In this instance, we feel it is beneficial to include the second reference despite the slight repetition in content: we think it is important to take the opportunity to reference relevant work in the field (Zhang et al., 2023) and (DeMaria et al., 2025) at the relevant places in the intensity and structure forecasting section, but also to remind the reader the fundamental challenge that we are resolving with cyclone co-training.

R2 Comment 12: *L339: operational meteorology is not appropriate; TC dynamics is precise.*

Thank you for raising this. We have edited this section and the term “operational meteorology” no longer appears.

R2 Comment 13: *L358: You may consider a sub title around L358.*
Thank you, the sub-title here is "Wind probabilities and large ensembles” but this was separated from the corresponding text by figure 4. We have now changed it so that the title appears next to the relevant text. We hope this makes the subtitle clearer.

R2 Comment 14: ***Remarks on code availability:*** *The key algorithms are provided. But the codes cannot be run independently if no information about software/hardware environment and python package dependencies are provided. There are also no enough instructions for installing and running the application. I recommend a Cyclonecast of appropriate size for running can be uploaded. The authors can also upload the Cyclonecast output of atmospheric field X. Then we can run the tracking algorithm by ourselves. I recommend some minimum level of visualization scripts to be provided to show the results.*
See Section 1.1 for our unified response on code availability and open-sourcing.

5 Response to Referee #3

R3 Comment 1: ***Referee #3 (Remarks to the Author):***

The present manuscript introduces CycloneCast, an AI-based, operational model for forecasting tropical cyclone (TC) intensity, track, and structure. CycloneCast is trained to jointly predict the $0.25°$*-resolution environment (from ERA5: 6 atmospheric variables on 13 levels and 6 surface variables) and our best record of TC track and intensity (IBTrACS, i.e., tabular data). Interestingly, CycloneCast achieves this by converting IBTrACS into two* $0.25°$ *fields: (1) a field indicating the probability the cyclone exists, based on Gaussian blobs centered on the track, and (2) scalars describing TC properties, conditioned on this existence (Section 2.2). Cyclonecst(a large graph neural network-based model, see Section 1.2) is trained to optimize the continuous ranked probability score (CRPS) via a multi-stage training procedure (see Section 12).*

According to standard (WeatherBench2-like) evaluation procedures, CycloneCast outperforms the state-of-the-art GenCast in predicting the target global environmental variables (Section B.2). The TC-centric evaluation on IBTrACS uses three baselines (Figures 2–5): ECMWF ENS (considered the best ensemble model we currently have globally for TCs), a debiased Gencst(representative of SOA AI weather models), and NOAA HAFS-A (arguably the top operational baseline for the North Atlantic and East Pacific basins). This evaluation shows significant improvement over existing baselines, especially for intensity and rapid intensification (for which there is currently no equivalent global product), with statistical significance tests reported in Appendix B.

Overall, this is more than just an impressive interdisciplinary collaboration and could fundamentally change the way tropical meteorological forecasts are routinely made, and where resources are invested for further improvement. I've independently evaluated live forecasts of the corresponding FNV model released through WeatherLab as part of research collaborations; live forecasts are significantly harder than standard ML evaluation, and FNV still showed superior probabilistic intensity and rapid intensification forecasts compared with existing models. Given the deadliness and hazard potential of tropical cyclones at the global scale, CycloneCast could very well warrant an eventual Nature publication if other, independent peer reviews concur.

However, given previous advances in AI weather prediction, I am slightly worried about the legacy of this manuscript if published. I therefore mostly focus below on replicability issues and on statements that could limit, rather than encourage, future research in (AI for) tropical meteorology.

We thank Referee #3 for their thorough and constructive review. We particularly appreciate their recognition that this work "could fundamentally change the way tropical meteorological forecasts are routinely made" and their thoughtful suggestions for strengthening the manuscript. We address their concerns on replicability in detail below.

R3 Comment 2: ***I) Major comments***

1) General lack of transparency

(1.1) Exact architecture, exact number of parameters, hardware used, and time for training vs. inference (important for replicability, but also for AI sustainability studies). The manuscript would benefit from a diagram clearly outlining the architecture beyond what is presented in text in Section A.1. This is especially given the claim around L367 about training efficiency. Aspects that are already explicit enough could serve as a model for aspects that are less detailed: the CRPS loss is explicit, and Table 1 (p. 29) does a good job of presenting the learning schedule.

We have added a detailed architecture description in the Methods (Section 12.1), specifying: ∼180M parameters per model seed (4 seeds), latent dimension 768, 24 graph-transformer layers, 6 attention heads. We have also added a "Computational resources" paragraph to the Methods section. Training takes approximately 4 wall-clock days per model seed, using a combined total of 560 TPUv5p

and TPUv6e days of compute per seed (4 seeds total). A single 15-day forecast trajectory takes just under 1 minute on a single TPU v5p, with each ensemble member generated in parallel.

R3 Comment 3: *(1.2) Some sensitivity/ablation tests would be helpful. For instance, the 120 km ℓ parameter in Eqs. A2 and A3 is central to many of the manuscript's claims/results and seems especially important to test.*

The 120 km parameter was chosen because it is (a) large enough to contain multiple 0.25° grid cells in diameter for robust gradient signal, (b) small enough to avoid overlap between simultaneously active cyclones (the minimum inter-cyclone distance in the historical record exceeds 240 km), and (c) inspired by operational ECMWF and NHC wind probability verification disc sizes. Unfortunately, tweaking it requires expensive retraining. We have added two ablations in a similar vein:

- Our 2025 evaluations also contain the version of the tracker than ran live from June to September, showing similar tracks but degraded scalar prediction.
- We ran the Tempest Extremes physics-based tracker on the non-cyclone fields, showing roughly similar track performance and poorer intensity.

R3 Comment 4: *(1.3) The geographic validity of each model (e.g., HAFS only works for the NA / maybe EP domain) could be more explicitly stated and discussed. This is a substantial advantage of CycloneCast.*
Done.

R3 Comment 5: *(1.4) Is there a risk of erroneous transfer learning from basins with large sample size and high data quality (North Atlantic) to, e.g., North or South Indian Ocean cases? This could be mentioned more explicitly.*
This is a valid concern. WN-C is trained globally on all IBTrACS basins simultaneously, so the model could in principle learn biases from data-quality discrepancies between basins. However, the atmospheric training data (ERA5/HRES-fc0) is globally uniform in quality, and cyclone-specific training uses IBTrACS data which, while North Atlantic/East Pacific data is of highest quality, also includes WMO-standard reports from all basins. For our evaluation, the 2023–2024 test years cover all basins, where IBTrACS data quality is sufficient for reliable verification globally. The 2025 test year is restricted to the Atlantic and East Pacific basins, since non-NHC basins have not been finalized.
More importantly to answer your question, our per-basin evaluations (Section 2.9) show consistent improvements across all basins in 2023–2024, suggesting the model generalises well.

R3 Comment 6: *(1.5) The authors mention do not mention why, during training, they chose to use only two members in the ensemble (L169–170) to calculate the CRPS (and not more). Leutbecher et al. (2018) does explicitly mention that at least two members are needed: "Moreover, the ensemble must have at least two members."*
As the reviewer points out, using $N = 2$ samples is the minimum required to compute an unbiased estimate of the fair CRPS (which requires at least 2 samples for the inter-sample term). Crucially, $N = 2$ provides an unbiased gradient of the true CRPS loss with respect to model parameters, so training with more members would not change the expected gradient direction—only reduce its variance at the cost of reduced initialization dates in the same batch. At inference time, we generate 50 or more ensemble members to fully characterise forecast uncertainty. We have added a citation to Leutbecher (2019) and clarified this in the Methods (Section 11.3).

R3 Comment 7: *(1.6) Related but minor: It would be helpful to have a citation for the fair CRPS, e.g., Ferro et al. (2013).*
Done.

R3 Comment 8: *(1.7) For TC track evaluation (Equation A1): is the energy score calculated only for the position (track), or over the entire set of predicted TC characteristics?*

- *If the former, is it computed with Euclidean distance in degrees (or some great-circle metric distance)?*
- *If the latter, was the calculation done over normalized values?*

Additionally: if using the fair variant, does one get $1/N$ in front of term 1 and $1/(2 \cdot N \cdot (N-1))$ in front of term 2, or something different? It would be helpful to make it explicit. The Position Energy Score is applied only to cyclone track positions (latitude, longitude). It uses *euclidean* distance on the sphere, we we are not sure whether an equivalent geodesic version exists. We use the fair variant of the energy score, computed analogously to the fair CRPS, to account for finite ensemble size. We have clarified all of these points in the Methods and supplementary information (Section 1.2.3Cyclones/main.pdf).

R3 Comment 9: *(1.8) Importantly: there is no explicit mention of the reliance on US-reported values in IBTrACS. While it is often a necessary choice to avoid conflicting definitions of TC intensity, this choice and its consequences on data quality in non-US basins should be explicitly discussed.*
We thank the reviewer for bringing it up, as multiple agencies have asked us about this as well. We have added a clarification in the methods.

R3 Comment 10: *(1.9) Also, there is a major abstract claim of extending forecasts up to 15 days, but little evidence in the text past 5 days. Would it be possible to clarify this aspect?*

Our public weather and cyclone forecasts extend up to 15 days. For every evaluation, we use the maximum possible lead time that gives confident results:

- For paired cyclone evaluations, we are limited by the small number of cyclones lasting more than 7 days. We are also limited by the 5.5 days of HAFS-A lead time, so we do a break in the plot and continue without it.
- For unpaired evaluations, we are limited to 10 days by the length of ENS cyclone tracks in 00/12 initializations.
- For general weather forecasting, ENS lasts up to 15 days so we evaluate all 15 days.

R3 Comment 11: ***2) Giving an impression of "solved problem", potentially impeding future research in tropical meteorology***

(2.1) Limitations and outlook are glossed over (there is a quick, appreciated nod to "IBTrACS should be maintained"), and this is especially visible in the conclusion section. Especially given the current defunding of some of the public weather enterprise, not explicitly mentioning the work's limitations is dangerous and a potential creativity killer for future research. Especially given the manuscript's potential impact, would it be possible to list potential axes of improvement for future research and operations? Seeing the results, ideas include:

- *Challenges in data assimilation: is the current HRES situation satisfactory, or should we move toward end-to-end observation-to-TC forecasting, or both?*
- *Global data quality discrepancies: more discussion of issues related to better quality data in the NA/EP basins, as well as limitations related to using the US definition of intensity.*
- *While wind speed, pressure, track, and structure are discussed, there is still a need to work on precipitation, which is only briefly mentioned in the conclusion.*

(2.2) In general, especially given the large interdisciplinary coauthor list, it would be helpful to discuss what could be next for "AI for tropical meteorology"? This is especially important to avoid giving the impression that the problem is fully solved as it's unlikely that this work already reaches the intrinsic limit of TC predictability.
We have expanded the conclusion to discuss a few axes for future improvement. We have also softened "overcome" and similar language throughout to avoid giving the impression that TC forecasting is a solved problem.

R3 Comment 12: *(2.3) Is there a plan to release the training/evaluation code for the global tropical meteorology community to make progress? The risk is that this code stays confined within the NHC/DeepMind communities, which would hinder global progress for the regional prediction centers.*
We have made a common answer at section 1.1 regarding what code we are sharing when and the approach we took.

R3 Comment 13: ***3) Clarifications related to tropical cyclogenesis***
(3.1) Around L146, the manuscript claims CycloneCast "handles tropical cyclogenesis", which is potentially impactful but also risky as written: there is limited consensus on an exact definition of TCG (and different communities/datasets define "genesis" differently), so the current phrasing reads stronger than what is explicitly demonstrated.
Please note that we have now removed this sentence altogether while streamlining our text. We have confined mentions of "cyclogenesis" to the section "Cyclone tracking algorithm" where we use the term in a precise sense, to refer to the cyclogenesis step of the tracker, whose operation is defined more precisely. We hope this addresses your concern.

R3 Comment 14: *(3.2) Given the clear potential and the importance of cyclogenesis, I would encourage the authors to add a concise dedicated appendix/figure that (i) states the specific definition(s) of genesis they adopt (with caveats), and (ii) explains how this maps onto the model outputs. For instance, the authors could consider framing the Gaussian-blob "existence probability" field as a proxy for a TC seed, with an explicit tracking/thresholding rule that turns this field into a "genesis event" (L726 could be clearer on this proxy interpretation).*
Please see our response to the above comment: we have confined our usage of the term "cyclogenesis" to the section "Cyclone tracking algorithm" where we are referring to it in a precise sense. We have also added a remark in this section that makes the framing of the existence Gaussians as a proxy for cyclone activity and genesis. We hope this addresses your concerns here.

R3 Comment 15: *(3.3) Finally, since genesis is discussed (10.3.3) but not evaluated, I would ask for at least one targeted genesis evaluation under the chosen definition (timing/location error and simple event-based metrics would already help), ideally stratified by basin.*
We expect our model to do much better than ENS at cyclogenesis, as it is tuned to reproduce IBTrACS cyclogenesis criteria. In contrast, ENS is overly sensitive (which has its own advantages).

We actively chose to not evaluate cyclogenesis for two reasons: (1) It is indirectly captured by unpaired evaluations, as the model needs to predict the emergence of future cyclones to capture track and strike probabilities. (2) The exact moment and place of cyclogenesis is a particularly noisy part of the data. For instance, anecdotally, (non-co-author) NHC forecasters have asked us to not rely on their live assessment of cyclone existence in future versions of the model, to be able to provide an independent assessment of whether a cyclone has formed.

R3 Comment 16: ***4) Lack of details regarding TC structure***

(4.1) It is currently unclear how the manuscript converts IBTrACS wind radii by quadrant into a continuous near-surface wind field (e.g., what is shown in Fig. 1). Because this mapping is not unique, would it be possible to state the exact assumptions, parametric form (and any smoothing/interpolation across quadrants) in the appendices/SI?

Thank you for suggesting this. We have now fleshed out this procedure mathematically in "Griddification of cyclone best-track data" in the appendix. In a nutshell, we do not actually create a grid like the one shown in Fig. 1 (which is only intended as a visualisation of what the model predicts in physical space). Instead, we create a separate grid (i.e. a separate channel) for each of the twelve quadrant variables (aggregating all cyclones active at the given valid time in the given grid). This grid consists mostly of NaN entries except for a few (i.e. sparse) non-NaN entries close to the cyclone center. These are the values that the model is trained to predict. We hope that the mathematical formulation added in this section precisely clarifies the gridding process.

R3 Comment 17: *(4.2) Relatedly, the manuscript appears to rely on a simple albeit dated wind-pressure relationship; given how central intensity/TC structure is to the paper, I would encourage testing more recent wind-pressure relationships such as Chavas, Reed & Knaff (2017).*

We unfortunately do not have bandwidth to apply a new rule, as it would have also required retraining the parameters and redoing some maps that happen in real-time. We also do not expect more modern relationships to bring GenCast or ENS to be better than HAFS on intensity, and thus change the message of the progress in a material way. We note that this wind-pressure relationship was made to strengthen a baseline beyond its original accuracy, and have added a figure showing its positive impact (Supplementary Figure 10).

R3 Comment 18: ***5) Confusion about whether FGN is new or not***

The presentation of FGN reads as though it is being introduced "from scratch" here, which I found potentially misleading given the existence of the Alet et al. (2025) preprint, which predates this submission. I would recommend being explicit about provenance and clearly stating what is new in this paper versus inherited from earlier FGN work. The novelty of the TC-specific learning setup and evaluation appears strong enough on its own; clarifying lineage would by no means take away from this manuscript's novelty.

This is a reasonable question—we had flagged the relationship to the Alet et al. preprint in our letter to the editor, though this was not visible to reviewers. The content of that preprint has been folded into the present manuscript, which subsumes and substantially extends it. The preprint was not submitted to any venue and will not be submitted separately; its author contributions are part of this paper. We have clarified the text to avoid the impression that FGN is introduced here for the first time. We also verified that cyclones co-training does not degrade weather skill: performance on standard weather variables is extremely similar to a weather-only model trained with identical architecture and schedule—marginally positive, but within variance (Section 2.3). We now also use that model checkpoint as a baseline to show that cyclone co-training tracks and significantly improves intensities.

R3 Comment 19: ***6) "Earlification" is slightly obtuse***

Since many headline results rely on earlified runs, it would be helpful to have more context/explanations than what is already provided in Section 14: For instance, a schematic showing how TCVitals enters the pipeline and what is modified (track relocation, intensity/size/structure adjustment, etc.). More broadly, I could not easily find prior use of the term "earlification" in the TC forecasting literature, so as written it reads like an informal or new label for a family of established practices; I would strongly encourage anchoring it to the existing terminology and literature if possible.

We have expanded the earlification discussion in the Methods (Section 14), anchoring it to the standard operational practice of using "late" model forecasts from a previous forecast cycle and correcting them with real-time observations. This is the standard procedure used at NHC and other operational centers when a model's analysis inputs arrive after the forecast cycle deadline. We have added a schematic illustrating the timeline of synoptic time, real time, and the correction procedure (Extended Data Fig. 3). Based on internal discussions and reviewer feedback, we have also changed the terminology to "interpolation", which is more standard in the literature.

R3 Comment 20: ***II) Some minor comments***

(i) The authors named their algorithm "CycloneCast": what about extratropical cyclones?

We have renamed the model to WeatherNext Cyclones, to convey its lineage and future effects and better convey that it is also a (state-of-the-art) medium-range weather model. We know the exact definition of cyclone is a bit imprecise, but think it is a reasonable description of the model.

R3 Comment 21: *(ii) Section 2.2: more comments on how the Gaussian-blob technique (e.g., Law and Deng, 2018) addresses the fundamental issue of data imbalance in tropical meteorology would be helpful.*

The Gaussian-blob (renamed to Gaussian-kernel now) does not per se address data imbalance, the co-training with weather does. Predicting a gaussian kernel encourages the model to predict whether a cyclone is there (0 or part of the blob) as well as its exact location (getting the center of the gaussian off introduces an error in the kernels proportional to the error).

R3 Comment 22: *(iii) L895: table labeled as 12.2, but points to Table 1 on p. 29.*

Fixed, thank you for pointing it out.

6 Response to Referee #4

R4 Comment 1: *I co-reviewed this manuscript with one of the reviewers who provided the listed reports.*
We thank Referee #4 for their review.

7 Response to Referee #5

Note: Referee #5's comments were provided as a PDF attachment.

R5 Comment 1: ***Review of "Tropical cyclone forecasting with AI"***

This manuscript describes the new CycloneCast AI forecasting model for tropical cyclones. CycloneCast is trained on a combination of global weather analysis data and a database of tropical cyclones (IBTRaCS). It predicts the track, intensity, and basic wind structural metrics of TCs globally. The model is evaluated for the years 2023 and 2024 and is shown to represent a step increase in skill at forecasting these critical metrics compared to traditional NWP models. Moreover, including CycloneCast in the track and intensity consensus ensembles used operationally at NHC greatly improves their skill. Thus, this work represents a novel and urgent contribution to the TC forecasting literature and its impact is certainly appropriate for Nature.

I have one general comment regarding the lack of verification results for the radius of maximum wind (RMW), and several other comments aimed at clarifying the presentation of the results and the explanation of the technique. All of these comments should be straightforward to address, and so I recommend accepting the manuscript for publication after minor revisions.

Recommendation: *Minor Revisions*

We are glad to hear your positive outlook on our work. We have strived to address your questions below in detail, including an additional verification analysis for RMW.

R5 Comment 2: ***General Comment:***

Section 6 does not contain a discussion of the verification results for the radius of maximum winds (RMW). The RMW is an important wind radius for anticipating storm surge impacts from TCs (e.g., Irish et al. 2007, `https://doi.org/10.1175/2008JPO3727.1`) and the area impacted by the most extreme winds in the eyewall at landfall. Even high-resolution NWP models like HAFS struggle to accurately predict the RMW (Trabing et al. 2024, `https://doi.org/10.1029/2024GL109663`) and so AI models like CycloneCast offer great promise for addressing this forecasting limitation. That said, the quality of RMW data in IBTRaCS is generally lower than that of other metrics like intensity and track – especially outside of the Atlantic basin and prior to 2021 (when NHC first started post-season quality control of RMW in the Atlantic; see Knaff et al. 2021, `https://doi.org/10.1016/j.tcrr.2021.09.002`). Because most of the training years for CycloneCast are before 2021, the model was likely trained on RMW estimates of questionable quality. However, the IBTRaCS RMW for Atlantic TCs in the verification years (2023 and 2024) is probably more reliable and could be used for verification. Therefore, I'd still like to see a few sentences added to the manuscript (possible in Section 6) about RMW verification results, or the reasons why such a verification was not conducted.

Thank you for raising this point. The primary reasons that we did not include an RMW verification are that: (a) there are significantly fewer counts of RMW than quadrant radii in the target data (which negatively impacts the statistical significance of the conclusions that can be drawn); (b) as you describe we have some reservations about the accuracy of the RMW field; (c) our core baseline for wind structure, HAFS, does not provide estimates of RMW for its earlified version (with ATCF code HFAI; note that it does provide RMWs for its late version). For these reasons, we decided not to include RMW in our verifications in the main paper. However, for completeness and transparency, we are including here a homogeneous paired evaluation in fig. 2 for RMW, against HAFS and ENS. As mentioned before, because the early / interpolated version of HAFS (code HFAI) does not include RMW, here we have taken late HAFS and applied to it the same earlification protocol that we applied to WN-C. This is why the error of HAFS cuts off earlier

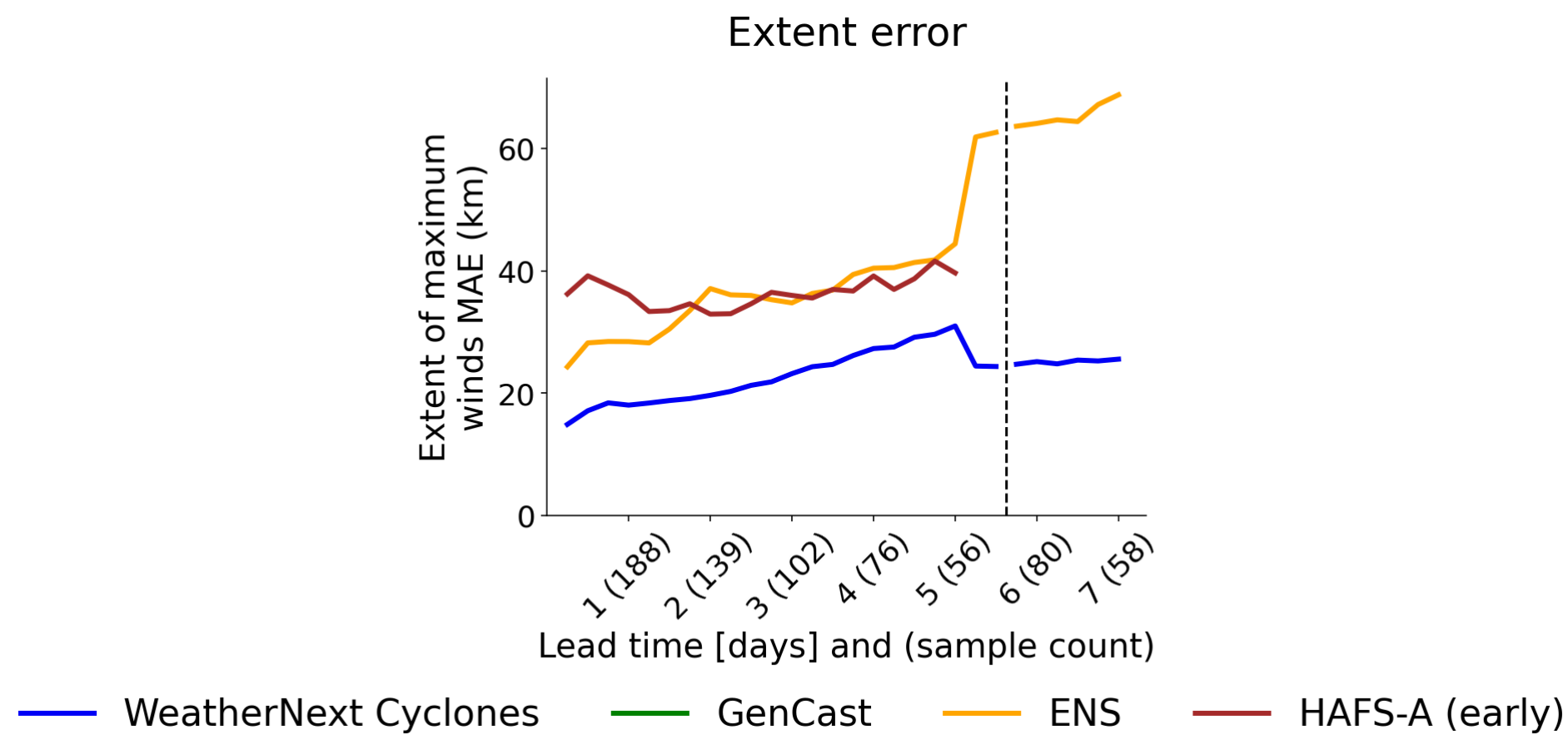

Figure 2: Additional verification results for radius of maximum winds (RMW) for 2023-2024. Here we are using the same evaluation protocol as the experiments in Figure 2 (main text). Note that here the HAFS baseline is not the official early / interpolated version of HAFS as issued in the NHC a-decks (because that does not contain RMW). Instead, we have taken the late HAFS baseline and earlified it using the same method we use for WN-C. These results demonstrate that WN-Coutperforms HAFS and ENS across all lead times for RMW, but due to concerns about the quantity and reliability of RMW data we have not included this verification in our main text.

than it does in Figure 2 (main text) in the main text. These results demonstrate that WN-Coutperforms HAFS and ENS also on RMW, however due to the aforementioned concerns, we are not comfortable adding this verification to our manuscript. We have added a comment in the Supplementary Information (Section 1.2) to explain this.

R5 Comment 3: ***Minor/Specific Comments:***
Lines 5-6: Given the title of the paper and the focus of IBTrACS on storms originating in or near the tropics, I recommend including the word "tropical" before "cyclones" here and elsewhere in the abstract. This avoids giving the impression that CycloneCast is also capable of predicting TC-specific metrics for mid- and high-latitude cyclones (which have different structural characteristics from TCs).
Thank you, we have now done this.

R5 Comment 4: *Line 51: Please change "NWPs" to "NWP models" to align with conventional terminology.*
Thank you, we have now done this.

R5 Comment 5: *Line 60: I believe by "core dynamics" you mean "inner-core dynamics". Also, the reference to Figure 1 on this line is not necessary and somewhat out of place here (see next comment).*
Thank you, we have removed this sentence altogether in the most recent revision of the manuscript.

R5 Comment 6: *Lines 63-64: It is unusual (at least in the meteorological literature) to reference a figure this early in a paper. It's fine to tease these statistics in these introductory paragraphs, but I recommend reserving specific references to Figure 2 for Sections 5 and 6. I also recommend moving the positions of Figures 1 and 2 closer to Sections 5 and 6 where they are discussed in more detail.*

Thank you for this suggestion. We agree that this may be somewhat unusual. We have now removed most of these statistics, and instead kept a more qualititative textual overview of the results.

R5 Comment 7: *Figure 1a: You might consider showing the ensemble mean forecast metrics (track and wind radii) instead of values from an individual ensemble member. The ensemble mean tends to have more skill than any one individual member, and showing the ensemble mean avoids having to justify why this particular member was selected for illustration.*
While we agree that the ensemble mean provides the most skillful deterministic guidance (which we evaluate extensively in Sections 5 and 6), we have opted to keep the individual ensemble member in Figure 1a to accurately reflect the model's generative process. The purpose of Figure 1a is to illustrate the direct output of a single WN-C ensemble member rollout. Computing the ensemble mean of gridded fields and cyclone trajectories results in a statistically smoothed, unphysical state that does not represent an actual output of the network at any given timestep. We have, however, updated the Figure 1 caption to explicitly clarify that panel (a) shows a single sampled trajectory that was chosen for illustration purposes, to prevent any confusion regarding forecast skill.

R5 Comment 8: *Figure 1a: I recommend including the best track of Milton on all four panels so the reader has a better sense of how good the ∼66-h landfall forecast is.*
While we appreciate the suggestion, we have chosen not to overlay the best track in this specific figure. The primary purpose of Figure 1 is to serve as a visual schematic of the model's pipeline and output data structures (gridded vs. tabular, global fields vs. wind probabilities), rather than to evaluate track accuracy on a single combination of storm and forecast initialisation. We perform rigorous, quantitative evaluations of track accuracy for two years of forecasts and hundreds of IBTrACS storms in subsequent figures (e.g., Figure 2) and Figure 3.

R5 Comment 9: *Figure 1 caption: A 24-hour time format is used when referring to UTC times, so I believe it should be 1200 UTC (instead of 12am UTC). However, it is unclear if you actually mean 0000 UTC, assuming 12am indeed refers to midnight. Please correct.*
Thank you for flagging the unclear time format in the caption. We have updated the caption to '00:00 UTC' (i.e. midnight).

R5 Comment 10: *Figure 1 caption: Change "An CylconeCast rollout" to "A CycloneCast rollout".*
We have now modified the figure caption, and this is no longer an issue.

R5 Comment 11: *Figure 2 caption: "Tropical storm" should also be included in the list "...a hurricane, a tropical depression, or a subtropical depression". It's also unclear why subtropical depressions are included in the verification.*
Thank you for spotting this, we have now added it. The reason why we include subtropical depressions is to follow the NHC verification protocol (see `https://www.nhc.noaa.gov/verification/pdfs/Verification_2024.pdf`), which keeps exactly these storm stages of development and drops all the rest (e.g. extratropical) from the evaluation. In particular it maintains subtropical depressions and subtropical storms in the evaluation.

R5 Comment 12: *Figure 2: Please clarify somewhere in the caption that the yellow line represents the ENS ensemble mean.*
Done, thank you for raising this.

R5 Comment 13: *Figure 2 caption: Please note in the caption that GenCast does not appear in panel (c) because it does not predict wind field radii.*

Now done, thank you for raising this.

R5 Comment 14: *Figure 2 caption: Please note in the caption why the sample counts in (c) are lower than in the other panels. I recognize it's because the number of storms with 64-kt wind radii is a subset of the total number of storms used for verification, but it would help to clarify that point.*
This is correct, the reason is because only a small subset of the storms have 64-kt wind radii resulting in lower counts. As part of a wider revision, we are suggesting to replace this with a 34kt wind panel instead, because 34kt are the most commonly occurring quadrant radii. Because of this, the counts are now significantly increased so we believe the caption does not need to be modified. However if we receive feedback to switch back to 64kt winds, we will add an appropriate clarification. Thank you.

R5 Comment 15: *Line 158: The use of "ensemble" as a verb is unusual; I recommend revising to something like "... combine their predictions into an ensemble."*
Thank you, we have done this now.

R5 Comment 16: *Line 161: Change "they grow" to "it grows"*
Thank you, we have now amended this.

R5 Comment 17: *Line 201: When you say "It" here, I assume you are referring to the ENS ensemble mean, but that should be specified.*
We have cut this sentence altogether now as part of an effort to reduce the overall word count of the manuscript.

R5 Comment 18: *Line 207: This is the first time the reader is seeing "HWRF" and "HMON". It would be worth spelling out these acronyms and briefly explaining that these are the legacy deterministic TC forecasting models used operationally.*
We have now done this. Thank you.

R5 Comment 19: *Line 231: I would not classify IBTrACS as a reanalysis; I recommend replacing "reanalysis" with simply "dataset" to avoid confusion.*
We removed the term "reanalysis" – thank you.

R5 Comment 20: *Line 289: I recommend just citing Fig. 1d in this sentence to make it clear that you are referring to the narrow region of Hurricane-force wind probabilities; the tropical storm force wind probabilities encompass the entire state of FL and, as such, do not obviously reflect a tightly clustered ensemble. If it's possible to include all the ensemble cyclone tracks in Fig 1, that would provide clearer evidence of a tightly clustered ensemble near the landfall position.*
We have now modified the reference to point just to Figure 1d. We prefer this over adding all ensemble tracks because that would make the figure too busy in our view and reduce clarity. Thank you for this suggestion.

R5 Comment 21: *Line 291: The end of the sentence ending on this line is awkward; perhaps a better way to say this is "... a generalisation of CRPS that is better suited for positions than scalars."*
We have now removed this sentence altogether while reducing word count. Thank you for flagging this.

R5 Comment 22: *Lines 304-305: This last sentence is confusing because the previous sentence states that the calibration of CycloneCast is similar to that of ENS, while this sentence implies that the calibration of CycloneCast is better than ENS. Please rewrite or remove this sentence.*
We have reworded this sentence, to help clarify this point according to your suggestion.

R5 Comment 23: *Lines 324-325: CRPS does not need to be spelled out again here.*
We have now fixed this spelling-out here and elsewhere in the paper.

R5 Comment 24: *Lines 340-341: I do not think this statement is correct; RI predictions can be (and are) evaluated at any forecast lead time, not just within the first 24 h. Please clarify and/or adjust this sentence.*
While there exist verification studies in the literature that evaluate RI at lead times beyond 24h, we follow the NHC verification protocol and constrain ourselves to the first 24h. For reference see `https://www.nhc.noaa.gov/verification/pdfs/Verification_2024.pdf`. In addition, we have slightly altered the wording as well to make sure it does not sound we are suggesting that evaluating RI beyond lead times of 24h is non-standard.

R5 Comment 25: *Lines 342-343 and Fig. 3e: The "recall-precision" terminology used here is non-traditional in the atmospheric sciences. I believe the equivalent (and more commonly used) terms for recall and precision are Probability of Detection (POD) and Success Rate, respectively. See Roebber (2009, `https://doi.org/10.1175/2008WAF2222159.1`).*
Thank you, we have added these terms to the text as alternative terminology to bring all audiences on the same page.

R5 Comment 26: *Figure 3e: The different blue stars in this panel are not adequately explained in the text or caption. I assume they represent different required thresholds on the number of ensemble members predicting RI to be considered an RI prediction. If that is indeed the case, then as a matter of presentation clarity I recommend using fewer stars (thresholds) and labelling them accordingly in Fig. 3e so the reader knows which stars in the diagram correspond to the strictest vs. most lenient RI classifications from CycloneCast.*
Thank you for the suggestion. Yes, this is correct: each star corresponds to a different probability threshold at which we choose to clasify a forecast as an RI event or not. Here is an explanation in more detail: Each sample from WN-Ccorresponds to a possible scenario in which RI may or may not occur. Given an ensemble of such forecasts, we can compute the probability that the model places on RI as the fraction of ensemble members for which RI occurs. Finally, given this probability, a forecaster (or decision maker) needs to decide whether or not to issue an RI forecast (or take mitigating actions), which is a binary decision, so the probability must be thresholded at some value. Different threshold values determine different trade-offs between false positives and false negatives.

We are inclined to not reduce the number of stars (probability thresholds) because we want to be transparent about our results: we do not want to cherry-pick specific values, but instead we want to show the full trade-off achieved by the model. We want to help reduce ambiguity and possible confusion, so we have amended the text accordingly to explain the stars better, and have tried to do this concisely to satisfy the word count constraints of the journal. We highly value your feedback here and hope our preference to not change the figure is not mistaken for anything except striving to remain transparent about the model performance across all probability thresholds.

R5 Comment 27: *Figure 3 caption: The caption should explicitly state that the shading in panel (e) is the critical success ratio, as that is not clear from the text.*

We have now fixed this – thank you.

R5 Comment 28: *Lines 346-348: It's difficult to see how CycloneCast improves upon the deterministic high resolution NWP models at RI prediction based on Fig. 3e. I assume it's because most of the blue stars (including many with the highest thresholds of members predicting RI) are in regions of the diagram corresponding to higher CSI values than the deterministic models, but that should be stated more clearly in the text.*
Thank you for raising this, we have now modified the text to: "The performance curve for WN-Cimproves on those of leading operational models, as it often achieves a better precision for the same level of recall. This indicates a better trade-off between correct detections and false positives in the Atlantic and East Pacific" We hope that this addresses your concern here.

R5 Comment 29: *Line 396: Please expand the last part of this sentence for added clarity, e.g., "...jumps from 0.5 in the 50-member ensemble to 0.7 in the 1000-member ensemble."*
Thank you for the suggestion. While adjusting the length of our submission we simplified this section to shorten it, and it now makes a relatively simpler comparison: "For instance, at a 0.001 cost/loss ratio, 1,000 members at 7 days provide more REV than 50 members at 5 days."

R5 Comment 30: *Figures 5a,b: The gray and blue lines in these panels are difficult to differentiate. I recommend re-creating these panels using more contrasting colors (grays and reds, for example).*
Thank you for the suggestion, we have now updated this figure to use blues and reds instead. For consistency, we would like to keep blue for WN-Csince this is what we use in other parts of the paper. Thank you for the suggestion.

R5 Comment 31: *Figure 5 caption: In the last sentence of the caption, it's more accurate to say that IVCN+CycloneCast outperforms IVCN across all lead times up to 5 days (not 6 days).*
We have now modified the caption accordingly, to say "outperforms IVCN at lead times up to 5."

R5 Comment 32: *Lines 864-867: Please clarify in this description which datasets are used during the overlap years of 2016-2018.*
We have added a detailed layout of our training strategy in "Table 2: Model training phases and hyperparameters" which should help clarify this point: During pre-training stages 1 through 5, we train the model on ERA5 data (up to 2018) and then, during finetuning stage 6 we train on HRES-fc0 (from 2016 onwards), so effectively, during the overlapping period, we train on both data sources (but at different finetuning stages).

R5 Comment 33: *Line 904: Remove repeated instance of "number of" on this line.*
Now fixed, thank you.

R5 Comment 34: *Line 919: Please include a citation for the Tempest Extremes tool, e.g., Ullrich et al. (2021, `https://doi.org/10.5194/gmd-14-5023-2021`).*
Done, thank you for raising this.

R5 Comment 35: *Line 1019: Please correct grammar issue: "of the of year"*
Fixed this to "of the year's progress." Thank you.

R5 Comment 36: *Line 1082: Please change "computing" to "compute".*
Now fixed, thank you.

R5 Comment 37: *Figures B11-B13 captions: The last sentence in each of these figure captions has an odd structure; consider revising to: "GenCast predicts track probabilities, but not wind radii, which is why it only appears on the bottom row"*
Now fixed, thank you.

R5 Comment 38: *Figure B14 caption: Remove boldface after first sentence.*
Now fixed, thank you.

R5 Comment 39: *Lines 1276-1277: This is unclear to me. Do you mean to say the performance of a model at forecasting intensity at the beginning of a TC's lifecycle is consistently related to its future performance for that same TC? If so, that is not obviously apparent to me from Figs. B17-18.*
We agree with you and have removed this sentence.What we originally meant to say is that in the geospatial decomposition figures, a track that appears blue (red) at early lead times also appears blue (red) at subsequent lead times (at least qualitatively). Therefore, if this holds across all lead times a forecaster could compare the errors of two models at the beginning of a cyclone's lifetime to adjust their predictions for subsequent forecast rounds. Even though it is qualitatively plausible that this approach could work, we have not conducted a verification of this method, so we have now removed this statement.

R5 Comment 40: *Section B.8: I'd be more curious to know if CycloneCast tracks tend to be better (than HAFS or ENS) at specific intensities. One way to examine this would be to see if there are strong correlations between relative improvements in track and intensity. It also appears from Figs. B17-18 that CycloneCast positions tend to improve the most upon HAFS and ENS positions around the times where there are substantial changes in cyclone heading (e.g., during TC recurvature). This may be worth mentioning briefly in this section.*
We thank the reviewer for this suggestion to probe the relationship between our track and intensity forecasts. To fully address this, we first investigate WN-C's ensemble mean error statistics in isolation, and then examine its relative improvement over the HAFS baseline across specific storm intensities. As for the paired evals in the manuscript, we subsetted to cases where the ground truth was a hurricane, a subtropical or tropical storm, or a subtropical or tropical depression.

First, examining WN-C's absolute performance, we computed the Pearson correlation (r) between the model's track errors and intensity errors. As shown in Figure 3 below, we found low correlation magnitudes across lead times of 1 day, 3 days, 5 days, and 7 days ($r \in [-0.12, 0.10]$). At 3 days and 5 days, the correlation is significant but only weakly positive ($r = 0.07$ and $r = 0.10$, respectively). This demonstrates that WN-C's capacity to accurately forecast a cyclone's intensity is not tightly coupled to its track accuracy.
Next, when plotting WN-C's absolute intensity error against the *actual* ground-truth intensity of the cyclone, we observe a highly significant, moderate positive correlation ($r \approx 0.38$ to 0.50 across the lead times tested). This aligns with standard meteorological expectations: absolute intensity errors scale with the intensity of the cyclone, as capturing the extreme peaks of major hurricanes remains inherently more challenging than predicting the intensity of weaker systems.
Finally, we address the core of your question: does WN-C's advantage over HAFS depend on the specific intensity of the storm? To answer this, we computed the relative intensity error difference (WN-C - HAFS, where a negative value means WN-C has lower ensemble mean intensity error) and correlated it against the actual ground-truth intensity.

We find no statistically significant correlation at any lead time ($p > 0.05$, $r \approx 0$). This is an encouraging result: it demonstrates that WN-C's performance advantage over HAFS is uniform

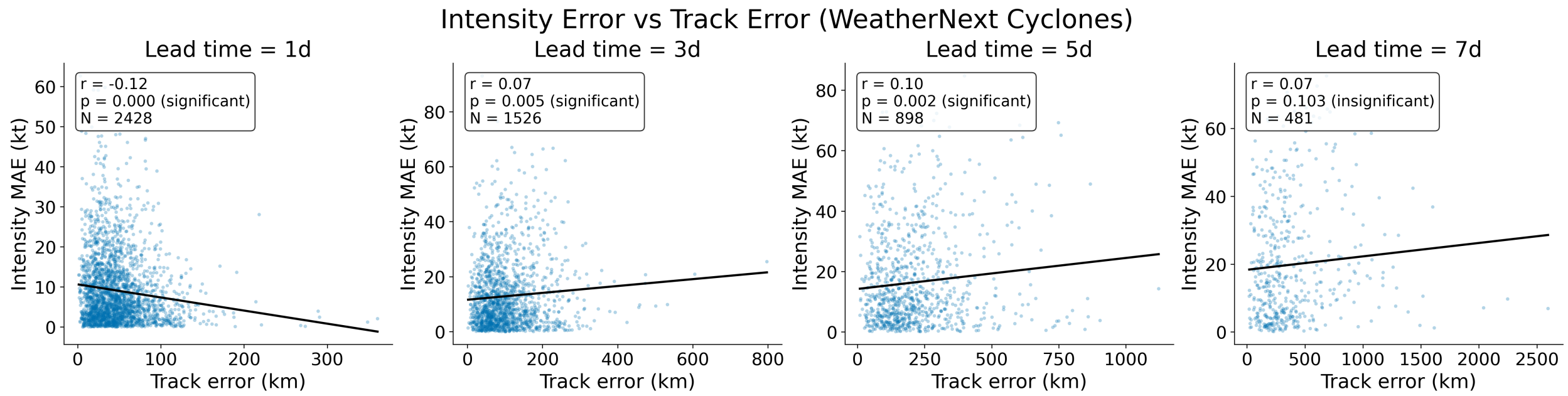

Figure 3: Pearson correlation (r) between WN-C track error and intensity absolute error at 1, 3, 5, and 7 days lead time. N denotes the number of forecast samples.

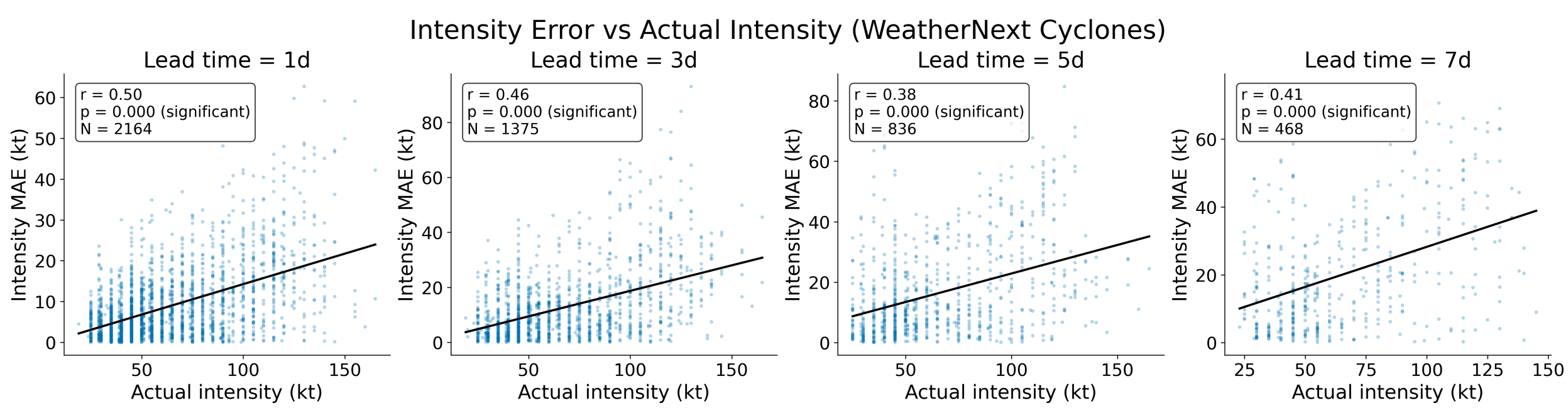

Figure 4: Pearson correlation (r) between WN-C intensity absolute error and the actual ground-truth intensity at 1, 3, 5, and 7 days lead time.

across the entire spectrum of storm intensities. The model does not solely gain its advantage on weak tropical depressions; it consistently outperforms HAFS regardless of whether the system is weak or a major hurricane.

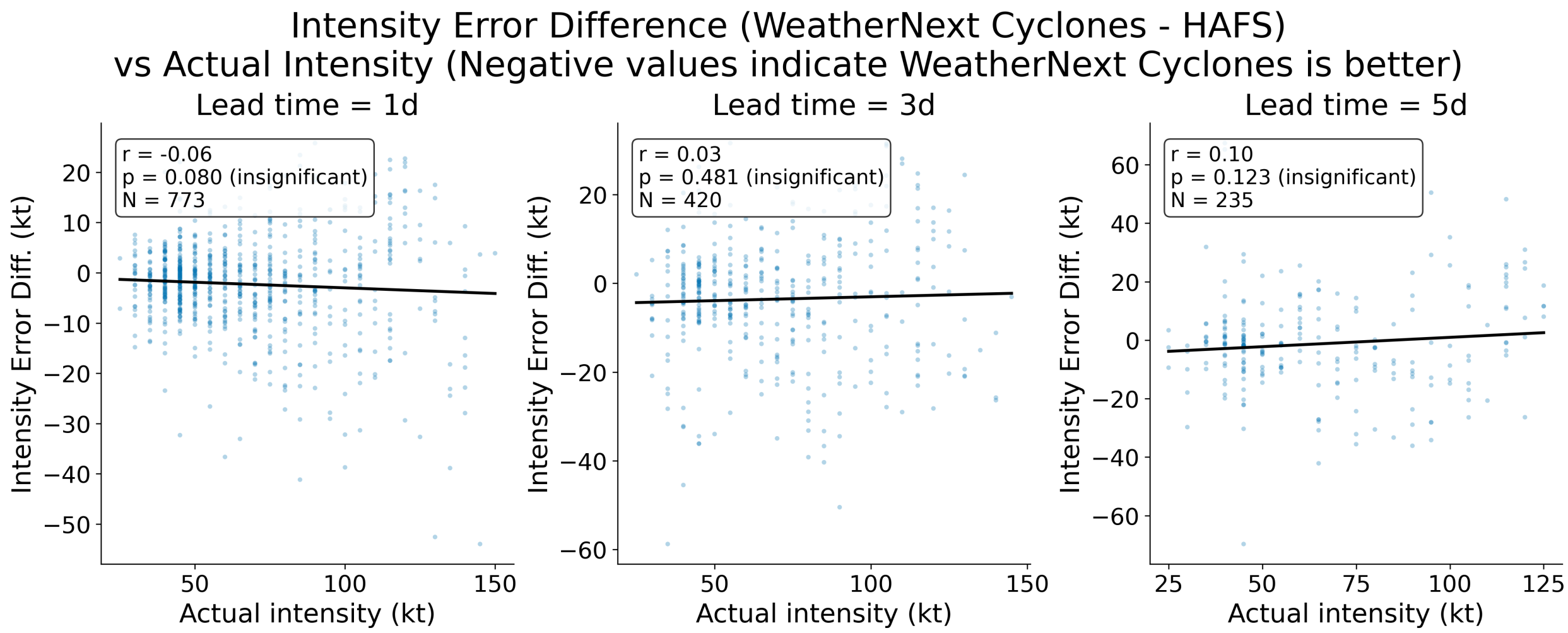

Figure 5: Pearson correlation (r) between the relative intensity error difference (WN-C - HAFS) and the actual ground-truth intensity of the cyclone at 1, 3, and 5 days lead time. Values below 0 indicate WN-C outperformed HAFS.

We hope the reviewer finds these results interesting, which may also be of interest to operational forecasters, because it suggests WN-C's ensemble mean intensity guidance can be relied upon over HAFS roughly equally across storm intensities. We decided against adding these figures or results to the manuscript to keep our resubmission to a reasonable size, but we can add it in some capacity if the reviewer or editor prefer.

R5 Comment 41: *[Code availability:] The code provided with this manuscript is well documented and structured. It is not the end-to-end CycloneCast model, but one could conceivably use the code provided along with the well-documented codebase already publicly available on the GraphCast github page to train and run a version of CycloneCast. Therefore, I think the provided code is sufficient.* We thank Referee #5 for this positive assessment. See also Section 1.1 for our unified response on code availability and open-sourcing.

2 Response to Referee #2

We thank Referee #2 for their careful re-reading of the revised manuscript and for the additional comments below.

R2 Comment 1: *L28–L30: Need reference here.*
Done.

R2 Comment 2: *L37–39: Why is NOAA-HAFS's track prediction not accurate since it is a storm-following model which captures both large-scale background flow and fine-scale TC dynamics? You should rephrase the statements here.*

Also: In L59–61 you state WN-C's ensemble mean is significantly more accurate than the specialised HAFS model on intensity and quadrant wind radii, but not on tracks. This is not consistent with the arguments that HAFS's track error is large.
Thank you for raising this. We would like to clarify that we did not claim HAFS was inaccurate at track forecasting. Rather, that the most skilful track models are *global* ensemble systems (ENS, GenCast), not regional models; as shown in Figure 1. This is why we only mention HAFS for intensity and wind radii, where it was previously state-of-the-art.. HAFS's track performance is sub-optimal relative to these global models for several reasons, discussed with NHC forecasters: (i) inter-mesh interactions can cause the regional model to lose part of the global synoptic context that drives large-scale steering currents; (ii) some subgrid physics parameters are tuned primarily for intensity rather than track; and (iii) as a single-trajectory deterministic model, HAFS cannot reduce variance through ensemble averaging, which contributes to higher mean track error. Its high resolution makes ensemble modeling too expensive.

We have rephrased the relevant passage to avoid any ambiguity.

R2 Comment 3: *L93–98: I think this paragraph should appear in section 2.2, following L115. In equation (1), X's prediction is completely irrelevant to C.*
Good idea; we have moved this paragraph to the next section as suggested.

R2 Comment 4: *L122–L126: I do not quite follow what you want to express here. You seem to want to argue that CRPS is not a good objective to optimize, but you still use CRPS loss. I don't understand the "marginal" and "joint" here.*
Thank you for flagging the confusion. We have slightly modified the sentence to make it clearer. By "marginal" we mean the distribution of each variable at each grid point independently; by "joint" we mean the full multivariate distribution including spatial and cross-variable correlations. Our point is that while we *train* using the marginal fCRPS (which is computationally tractable and provides clean per-variable gradients), we need the resulting ensembles to also capture realistic *joint* structure. The justification for how marginal training achieves this is in the following paragraph ("We enable the learning of joints. . . ") and in the new supplementary evaluations of spatial pooling, derived variables, and power spectra.

R2 Comment 5: *L251: You should briefly explain why GenCast should be debiased here.*
Agreed. We have added a brief explanation noting that GenCast's raw intensity forecasts are systematically low-biased because the model is trained on coarse 0.25° analysis data that cannot resolve a cyclone's peak winds, and that the pressure–wind speed relation corrects this known limitation to provide the strongest possible AI baseline for intensity.

3 Response to Referee #3

We thank Referee #3 for their continued engagement with the manuscript and their thoughtful follow-up comments.

R3 Comment 1: *L13: "WeatherNext Cyclones enables up to 1000 member ensembles which better capture...": On which infrastructure can we expect up to 1000-member ensembles? Just adding "on a XYZ TPU" would be enough to avoid misleading most readers who do not have access to such resources.*
Thank you for this suggestion. We have added a clarification to the abstract and a detailed "Computational resources" paragraph in the Methods section, specifying that each 15-day forecast trajectory takes just under 1 minute on a single TPU v5p, with all ensemble members generated in parallel. Including writing to disk, 1000-member ensemble thus completes in ∼20 minutes on 256 TPU v5p. We have also added a mini-appendix evaluating a lightweight 1-degree variant of the model, which is designed to run on more widely available hardware, demonstrating the scalability of the approach.

R3 Comment 2: *To avoid general confusion, I highly recommend going through each reference and preceding it with the appropriate "Methods" or "SI" qualifier. The change in the manuscript's outline has made many references confusing and/or obsolete, with many "Sections" actually referring to the SI, references to figure panels that have moved, etc.*
Thank you for flagging this. We have now updated all references throughout the manuscript to include explicit "Extended Data", "Supplementary", or "Methods" qualifiers as appropriate. We have also resolved cases where figure panel references had become outdated due to restructuring.

R3 Comment 3: *Follow-up on R3 Comment 18 (L129–130, L608): This is one of the reviews that I do not think the authors have properly addressed. The manuscript still makes it seem like it is the first to introduce FGN chronologically. Where exactly did the authors "clarify the text to avoid the impression that FGN is introduced here for the first time"?*
In our original revision we added the following passage to the Supplementary Material: "The weather-only version of the model architecture described in this paper was previously presented in our preprint (Alet et al., 2025), whose content is now contained in the present manuscript. The primary difference to WN-C is the removal of Stage 4 (cyclone output head warmup) and the exclusion of direct cyclone targets (DCT) from Stages 5 and 6 (see Table 2). We verified that this difference does not affect weather forecasting performance: WN-C's skill on standard weather variables is extremely similar to the weather-only model—marginally positive, but within variance." This preprint has not been submitted to any other archival venue, since we decided to expand the model further and apply it to cyclones before sending it for publication. We view this as similar to how the GenCast paper had a simpler pre-print months before getting submitted to Nature.

R3 Comment 4: *Follow-up on R3 Comment 11: I acknowledge the authors' mention that "tropical cyclone forecasting remains a challenging and fertile field for AI research", but this does not address the original review, which mentioned the threat to future research in tropical meteorology, much of which does not heavily rely on AI, or does not rely on it at all. Addressing this would not take away from the impressive achievements described in the manuscript.*
We appreciate the reviewer's concern and share their commitment to ensuring that this work supports, rather than displaces, the broader tropical meteorology community. It is difficult for us to make categorical statements about future non-AI research directions in a peer-reviewed manuscript

without stronger justification than we can currently provide. In presentations at conferences (including AMS Tropical), we have informally discussed how AI and physics-based approaches can coexist through better (re)analysis, physical insights, and improved evaluations. In the manuscript, we have highlighted specific limitations of our work that represent clear opportunities for continued research, including from non-AI perspectives.

R3 Comment 5: *Follow-up on R3 Comment 20: Renaming the model "WeatherNext Cyclones" still does not tackle the problem of extratropical cyclones. A quick mention of whether this will be included in the future, or whether it is clearly out of scope and would require a completely different model, would be helpful to anticipate future research.*
Thank you for pressing this point. We have made two changes: (1) In the evaluation methodology, we added: "Note that IBTrACS captures *tropical* cyclones, and thus only contains extratropical cyclones if they are part of an extratropical transition." (2) In the conclusion, we added "tropical" to the final sentence: "This framework is not limited to tropical cyclones; . . . " to make clear that the current work focuses on tropical cyclones while the underlying approach is extensible.

R3 Comment 6: *L80: Because the subsequent formulae use discrete, dimensionless time, it would be helpful to remind the reader that the time coordinate has been normalised using a 6-hour timestep.*
Done. We have added a parenthetical clarification after "Following standard operational practice" to remind the reader of the 6-hour timestep.

R3 Comment 7: *L86: "initial atmospheric variables $X^0, X^{-1} \ldots$": From IFS initial conditions?*
We use HRES-fc0, the zero-hour forecast from the ECMWF high-resolution model, which serves as an operational analysis. We have clarified this in the ground truth section of the evaluation methodology.

R3 Comment 8: *L48: "However, these approaches have not achieved state-of-the-art results": I would be more specific here and complete the sentence, e.g., "state-of-the-art results in" combined track and intensity medium-range forecasting, or real-time tropical cyclone forecasting.*
Thank you for the suggestion. We have added "in operational tropical cyclone forecasting" to complete the sentence.

R3 Comment 9: *L97–98: "By contrast, a two-step model that 1) predicts X alone, and then . . . would not have this transfer learning benefit": Here, one could cite examples that post-process AI weather prediction models, e.g., Bremnes et al. (2024) or Gomez et al. (2026) for a tropical cyclone example. I imagine this statement is implicitly justified by the comparison with GenCast in the manuscript? If so, being a bit more explicit could be helpful here.*
Yes, this is justified both by the comparison with GenCast (which is a strong AI weather model without cyclone co-training) and by the ablation study in Extended Data Figure 4, which demonstrates the benefit of co-training. We have also added the suggested citations. Thank you.

R3 Comment 10: *L137: "making it 8× faster": I imagine this is "at inference"?*
Yes, the 8× speedup refers to inference. We have clarified by adding "at inference" to the sentence.

R3 Comment 11: *Figure 2 caption: "Performance is evaluated only when all models make a prediction and the true system is classified as a hurricane, tropical storm, subtropical storm, tropical depression, and subtropical depression": While I understand that this choice was made for consistency with the NHC evaluation guidelines, it will result in a very incomplete picture of a prediction system, highly favouring "cautious" systems with many misses but excellent forecasts for the few*

systems they predict. Would it be possible to complement this with, e.g., each prediction system's hit rate?
Thank you for this thoughtful comment. You are right that if we evaluated solely on the paired metric with NHC storm classifications, the analysis could in principle favour overly cautious systems.

However, our unpaired evaluation using the REV metric (see main Figure 4, Extended Data Figure 7, and Supplementary Figures 14–15) does not subset storms based on their classification and includes all storm systems active in the IBTrACS database. Moreover, it is not a homogeneous evaluation, so it captures the performance of individual models against the ground truth. If a prediction system is overly cautious, it will produce too many false negatives and its REV performance will deteriorate compared to a well-calibrated model.

In addition, a separate hit-rate evaluation for the models in Figure 2 could unfairly impact the regional baselines' performance. Regional models such as HAFS may not always be run for all storms in real time due to computational and operational constraints, and a missing forecast would be interpreted as a miss.

We believe our current evaluation protocol (paired + unpaired) accurately measures performance on core cyclone forecasting metrics and also captures hit-miss events via the REV analysis.

R3 Comment 12: *L1170: "with some minimum probability": Would it be possible to give a number here? Is it an important hyperparameter in the context of WN-C?*
Thank you for raising this. Here we are referring to the 30% probability threshold used to define the ensemble mean, explained in the subsection "Mean absolute error (MAE) of ensemble mean." We have clarified this in the main text with: "with a minimum probability, i.e. the 30% threshold used in defining the ensemble mean (see Section 1.2.1)."

R3 Comment 13: *L1356: "Skill in forecasting these derived quantities relies on correctly modelling the dependencies of their constituent predicted fields": This is not the most direct way to evaluate these statistically, compared with directly evaluating the dependence structure/copulae. It could be clarified that this is because these derived quantities are physically important quantities.*
Thank you for this comment. We have amended the text to read: "Evaluating cross-variable dependencies can be done in different ways: we have chosen these because forecasting wind speeds is crucial across many applications (e.g., transportation), while the 300 hPa–500 hPa geopotential difference is a critical quantity for tropical cyclone prediction as well as weather forecasting more generally."

4 Response to Referee #5

We thank Referee #5 for their thorough re-reading of the revised manuscript and for the detailed list of corrections below, which have further improved the clarity of the paper.

R5 Comment 1: *There are several references throughout the revised manuscript to sections, tables, or figures that are ambiguous because the authors do not specify if these items are in the main text, the supplementary material, or the extended data sections. I attempted to compile an exhaustive list of problematic references below, but the authors should carefully examine each reference in the manuscript to make sure it is accurate and unambiguously refers to sections/figures in the main vs. supporting/extended text. Generally speaking, references to main text figures/tables/sections do not need qualifiers while references to figures/tables/sections in the supplementary material or extended results do (e.g. Supplementary Section 2.1, Extended Data Figure 3, etc.)*
Thank you for carefully compiling this list. We have addressed each instance below, adding explicit qualifiers (e.g., "Supplementary Section", "Extended Data Figure") throughout the manuscript. We have also done a full pass to catch any additional cases.

R5 Comment 2: *Lines 145, 153: Indicate that these are references to Supplementary Section 2.4.* Done.

R5 Comment 3: *Line 164: Indicate that this is Supplementary Section 1.3.* Done.

R5 Comment 4: *Line 172: Indicate that this is Supplementary Section 2.2.* Done.

R5 Comment 5: *Line 188: Indicate that this is Supplementary Section 2.3.* Done.

R5 Comment 6: *Line 203: Indicate that this is Supplementary Figure 1.* Done. This now correctly refers to Extended Data Figure 1.

R5 Comment 7: *Line 204: Indicate that this is Supplementary Section 2.9.* Done.

R5 Comment 8: *Line 214: This number "4" in parentheses needs a qualifying word before it ("Section 4"? "Figure 4"?).* Done. This now correctly refers to Extended Data Figure 4.

R5 Comment 9: *Line 221: Indicate that this is Supplementary Section 1.2.3.* Done.

R5 Comment 10: *Line 228: Indicate that this is Supplementary Section 1.2.4.* Done.

R5 Comment 11: *Line 304: Indicate that this is Supplementary Section 1.2.5.* Done.

R5 Comment 12: *Line 306: I believe the figure references on this line are to Extended Data Figure 7 and Supplementary Figures 15, 16, and 17. Please adjust figure references accordingly.* Done. This now correctly refers to Figure 4, Extended Data Figure 7, and Supplementary Figure 13. The next sentence also now correctly refers to the wind speed probability reliability figures (Supplementary Figures 14 and 15).

R5 Comment 13: *Line 322: Indicate that this is Supplementary Section 2.1.* Done.

R5 Comment 14: *Line 544: Indicate that this is Supplementary Table 1.* Done.

R5 Comment 15: *Line 793: Indicate that this is Supplementary Section 1.3.* Done.

R5 Comment 16: *Lines 809, Table 2 caption: Please add the word "Section" before "2.2".* Done; both references to Section 2.2 have been specified.

R5 Comment 17: *Line 813: This reversed range of figures ("Extended Data Figures 13 to 4") seems incorrect. Please double-check the figure numbers/range.* Done. We resolved duplicate figure definitions by keeping them in the Extended Data section (where they are highlighted) and removing them from the Supplementary Information. This resolves `cleveref` numbering confusion and ensures correct ranges.

R5: Line-level corrections and wording suggestions

R5 Comment 18: *Line 126: The use of "wind pressure" is unusual. Do you mean a local minimum in "wind and pressure"?* Thank you; we have changed "wind pressure" to "surface pressure."

R5 Comment 19: *Line 137: I recommend adjusting to something like "...making it 8× faster and substantially easier to model the sparse cyclone targets" to address awkward grammar.* Done. We have adopted the suggested rewording.

R5 Comment 20: *Line 155: Please define HRES-fc0 the first time it appears.* Done. We have added a parenthetical definition at first use: "the initial step of the ECMWF HRES forecast, serving as an operational analysis."

R5 Comment 21: *Line 239: Please add the word "a" before "cyclone's."* Done.

R5 Comment 22: *Fig. 3e / Lines 276–277: This sentence seems like it would be more appropriate in the caption than in the main text.* Agreed. We have moved the sentence about stars corresponding to probability thresholds into the Figure 3e caption.

R5 Comment 23: *Fig. 3e caption: The statement about the "off-trend performance on the bottom-right" is helpful for interpreting the different stars, but I still felt a clearer statement is needed here. Perhaps a clearer way of saying this is: "the stars in the lower right (upper left) correspond to higher (lower) probability thresholds for RI."* Thank you for the clearer phrasing. We have adopted the reviewer's suggested wording, replacing the "off-trend performance" statement with an explicit description of the threshold-to-position mapping.

R5 Comment 24: *Line 343: To be consistent with the following sentence, change "and a mean" to "with an average."* Done.

R5 Comment 25: *Line 360: I recommend rewording to "from June 2025 to present."* Done. Changed "since June 2025" to "from June 2025."

R5 Comment 26: *Line 535: Change "entires" to "entries."* Done.

R5 Comment 27: *Line 540: Change "for quadrants" to "four quadrants."* Done.

R5 Comment 28: *Line 543: Move "IBTrACS-derived" to be before the word "gridded."* Done.

R5 Comment 29: *Lines 689–696: A lot of this material has already been covered elsewhere in the manuscript and can probably be removed or shortened considerably.* Done. We have shortened the description of the physical consistency constraints by replacing the itemised list with a single compact paragraph.

R5 Comment 30: *Lines 703–704, 707: It's unusual to use "ensemble" as a verb like this. I recommend changing to "combine their predictions into an ensemble."* Done. Changed "ensemble their predictions" to "combine their predictions into an ensemble" in both instances.

R5 Comment 31: *Line 722: Please spell out the acronym "RL" if this is the first time it appears.* Done. Spelled out as "reinforcement learning (RL)."

R5 Comment 32: *Lines 789–790: Please be cognizant of how many times this CRPS definition ("a strictly proper scoring rule...") is repeated throughout the manuscript. You probably only need to repeat this statement once or twice to reduce the word count.* Done. We removed three redundant definitions of CRPS as a "strictly proper scoring rule" in the Methods section, keeping only the first occurrence in the main text and the definitions in the Supplementary Material.

R5 Comment 33: *L53: IBTrACS should be spelled out the first time it is used.* We have added the full form (International Best Track Archive for Climate Stewardship) in the ground truth section of the evaluation methodology, together with the ECMWF acronym. We believe it reads better there, as the first substantive discussion of the dataset. Thank you for the suggestion.
